# Grouping Pentylenetetrazol-Induced Epileptic Rats According to Memory Impairment and MicroRNA Expression Profiles in the Hippocampus

**DOI:** 10.1371/journal.pone.0126123

**Published:** 2015-05-11

**Authors:** Xixia Liu, Yuan Wu, Qi Huang, Donghua Zou, Weihan Qin, Zhen Chen

**Affiliations:** Department of Neurology, The First Affiliated Hospital of Guangxi Medical University, Nanning, Guangxi, China; Huazhong University of Science and Technology, CHINA

## Abstract

Previous studies have demonstrated a close relationship between abnormal regulation of microRNA (miRNA) and various types of diseases, including epilepsy and other neurological disorders of memory. However, the role of miRNA in the memory impairment observed in epilepsy remains unknown. In this study, a model of temporal lobe epilepsy (TLE) was induced via pentylenetetrazol (PTZ) kindling in Sprague-Dawley rats. First, the TLE rats were subjected to Morris water maze to identify those with memory impairment (TLE-MI) compared with TLE control rats (TLE-C), which presented normal memory. Both groups were analyzed to detect dysregulated miRNAs in the hippocampus; four up-regulated miRNAs (miR-34c, miR-374, miR-181a, and miR-let-7c-1) and seven down-regulated miRNAs (miR-1188, miR-770-5p, miR-127-5p, miR-375, miR-331, miR-873-5p, and miR-328a) were found. Some of the dysregulated miRNAs (miR-34c, miR-1188a, miR-328a, and miR-331) were confirmed using qRT-PCR, and their blood expression patterns were identical to those of their counterparts in the rat hippocampus. The targets of these dysregulated miRNAs and other potentially enriched biological signaling pathways were analyzed using bioinformatics. Following these results, the MAPK, apoptosis and hippocampal signaling pathways might be involved in the molecular mechanisms underlying the memory disorders of TLE.

## Introduction

Cognitive deficits represent a serious neuropsychological obstacle in people suffering from temporal lobe epilepsy (TLE)[1]. Memory disorders, which are the most notably neural behavioral impairments manifested by TLE[2], currently attract a great deal of attention because their underlying molecular mechanisms remain unknown[3]. Seizures[4], medication[5], circuit reorganization[6] and abnormal forms of interictal activity[7] have been proposed to contribute to cognitive impairment. Evidence has supported the hypothesis that memory and learning are facilitated by the hippocampal cortex[8]. Additionally, neuroimaging studies reveal higher cognitive deficits after epilepsy surgery, whereas the hippocampal formation remains normal[9]. Previous lesion studies have demonstrated an important effect of entorhinal lesions on the hippocampi of TLE animals suffering from indirect excitotoxins (such as aminooxyacetic acid or gamma-aminobutyric acid) with memory impairment[10]. However, a recent study reported no neuronal cell loss but decreased expression of synaptophysin, SNAP-25, and Syt 1 in the hippocampi of KA-treated rats[11].

Currently, TLE-related cognitive disorders have been characterized by experimental models and memory and learning deficits were compared after preconditioning[12,13]. However, standardized neuropsychological tests to analyze memory impairment in epileptic rat models remain elusive. The Morris water maze (MWM) test is considered the primary method used in animal behavior research to analyze learning and memory[14,15]. PTZ-induced epileptic is the most popular animal model used by scholar to study seizure and its cognitive deficits[16,17]. Studies have showed learning and memory disorders caused by PTZ kindling, with a bad show in the test of MWM[18]. We therefore screened PTZ kindling-induced TLE rats using the MWM test to identify the TLE rats suffering from memory impairment among the rats with normal cognitive function.

MicroRNAs (miRNAs) are small regulatory RNA molecules (approximately 22 nt) and regulate gene expression negatively, playing a critical role in normal neuronal development[19]. miRNAs control various biological processes and involved in several neurological diseases, such as ischemic tolerance[20], Alzheimer’s disease[21] and Parkinson’s disease[22], as well as in the response to electroshock therapy[23]. Recently, increasing evidence has supported the involvement of changes in miRNA expression in the molecular mechanism of epileptic tolerance[24–29]. Furthermore, multiple lines of evidence support the role of miRNAs in memory formation mechanisms and in activity-induced gene expression. Synaptic plasticity and memory function were constrained in the presence of increased levels of miRNAs in rodent brains[30–31] and similar effects might occur in AD brains[32]. Wang et al[33] demonstrated impaired acquisition of trace fear memory in mice following reduced miR-132 expression in the hippocampus. In addition, a therapeutic approach was used to improve memory performance in MS patients after miR-124 inhibition in hippocampal neurons[34].

However, the effects of miRNAs on memory deficits in epilepsy alone have not been reported. Therefore, we profiled miRNA expression in the hippocampus of a PTZ-kindled rat with TLE-associated memory deficits and characterized the effects of specific miRNAs. We then also demonstrated potential correlation in the expression of miRNAs in hippocampal temporal lobe tissue and in peripheral blood. The results of this study may help explain the mechanisms of memory disorders and enable novel functional genomic analyses in temporal lobe epilepsy.

## Materials and Methods

### Animals and kindling induction

A total of 70 experimental healthy male Sprague-Dawley (SD) rats, which were 6–7 weeks old and weighed 220–250 g, were obtained from the Animal Experiment Center of Guangxi Medical University, China. Animals were raised in groups of five every cage under certain conditions (22–26°C, 50–60% humidity and a 12:12 light-dark cycle, with lights on at 8:00 AM). The rats were given free access to food and water. Animal care and sacrifice were conducted according to methods approved by the Guangxi Medical University Animal Experimentation Committee. All experiments were performed in accordance with the National Institutes of Health Guide for the Care and Use of Laboratory Animals.

All chemicals used in this research were of analytical grade. Pentylenetetrazol (PTZ) was purchased from Sigma-Aldrich (St. Louis, MO, USA) and freshly dissolved in physiological saline before administering injections. The rats were divided into a normal control group (n = 10) and a PTZ model group (n = 60) using a random number table.

TLE preconditioning was performed by a single intraperitoneal injection of PTZ (60 mg/kg) on the first day, followed by repeated injections of PTZ (35 mg/kg) on alternating days between 8:00 and 10:00 AM starting on the 3rd day, with a total of 14 injections. The control group rats received 0.9% physiological saline (10 ml/kg) i.p. every other 48 hours as sham preconditioning, with 15 injections total. The rats were weighed daily, and the dosage was adjusted according to body weight. The animals were monitored for 30 min after each PTZ treatment. Seizures were rated according to the following scale: 0, no change in behavior; stage 1, chewing; stage 2, gazing and head nodding; stage 3, unilateral forelimb clonus, twitching, and scratching; stage 4, rearing with bilateral forelimb clonus; stage 5, widespread muscle spasms, rearing with bilateral forelimb clonus and falling back[35]. Kindling was defined as ≥4 consecutive stage 2 seizures, or two seizures that were stage 4 or higher.

### Determination of memory dysfunction using the MWM test

The MWM apparatus (Panlab, Spain) consisted of a circular pool (120 cm in diameter, 50 cm in height), a round colorless platform (11 cm in diameter) and a camera installed above the center of the pool. The circular pool was filled with water (22±1°C) and consisted four quadrants. The platform was placed in a designated target quadrant 2 cm below surface. The camera was connected to a computer for synchronous image capture using a tracking system. The reference items around the pool remained unchanged, allowing the rats to use these items for spatial orientation, and the location of the platform remained in a constant position during 5 consecutive days of experiments. The data were acquired using SLY-WMS MWM system v. 2.1 software, and the experimenters were blind to the rat grouping assignments. The MWM test consisted of a place navigation test and a spatial probe test. The memory performance of all successfully established CEP rats was evaluated 24 h after the last PTZ administration.

The acquisition test was lasted for five consecutive training days, with four sessions per day. During each training session, the rats were placed into the water maze from four different starting positions and had 120 s to locate the hidden platform. The software system automatically began recording at the moment each rat entered the water until the rat found the platform (i.e., the escape latency period). A latency of 120 s was recorded when the rat failed to find the platform within 120 s, at which point, the rat was manually guided to the platform and allowed to stay on the platform for 20 s. The escape latency intervals were observed and recorded for each rat during each training session on the fifth day of testing. The hidden platform was removed after the fifth training session on day 5. Each rat was placed into the water facing the wall of the maze from four different starting positions and was given 100 s in the water per trial. The number of crossings that each rat made across the former location of the platform within 100 s over the four trials was recorded and averaged to evaluate the spatial memory of each rat.

The memory-impairment evaluation system for epileptic rats was established as follows. Two scores were obtained as a reflection of normal memory function according to data collected from the 10 rats in the normal group during two MWM sessions (<15 s escape latency for the place navigation test on day 5 and >20 crossings in 400 s for the spatial probe test). Then, 6 rats were selected from the 17 TLE animals because their data overlapped the scores of the normal rats; these 6 rats formed the TLE control group (TLE-C). The memory impairment in TLE (TLE-MI) group included the 10 rats with the worst performance in the MWM experiment (>50 s escape latency for the place navigation test on day 5 and <10 crossings in 400 s for the spatial probe test). Then, 6 rats each were randomly selected from the TLE-C group and from the TLE with memory impairment group (TLE-MI), and microRNA expression patterns in these rats were investigated using microRNA array and differential analyses.

### Tissue dissection and miRNA microarray analysis

Following the behavioral tests, rats from both groups (n = 6 for each group) were anesthetized with 10% chloral hydrate (3.5 ml/kg, i.p.), and 2–3 ml of blood was collected into cryogenic vials with EDTA using cardiocentesis. The rats were then sacrificed, and the hippocampus were rapidly harvested and removed to cryogenic vials with RNAlater (RNA Stabilization Solution, Sigma-Aldrich). The blood and hippocampal tissue samples were stored at -80°C until further testing.

miRNA microarray analysis was performed on total hippocampal RNA from TLE rats with memory disorders and from TLE rats with normal memory performance (n = 6 for each group). Total RNA was harvested using TRIzol reagent (Invitrogen) and a miRNeasy mini kit (Qiagen) according to the manufacturers’ instructions. RNA quality and quantity were measured using a NanoDrop spectrophotometer (ND-1000, NanoDrop Technologies), and RNA integrity was determined using gel electrophoresis. After measuring the quantity of total hippocampal RNA, the total hippocampal RNA samples from both groups of TLE rats were labeled using a miRCURY Hy3/Hy5 Power labeling kit (Exiqon, Vedbaek, Denmark) and then hybridized on a miRCURY LNA Array (v.18.0, Exiqon) using a hybridization system (Nimblegen Systems, Inc., Madison, WI, USA). Following several washing steps using a wash buffer kit (Exiqon), the slides were scanned using an Axon GenePix 4000B microarray scanner (Axon Instruments, Foster City, CA, USA).

GenePix Pro 6.0 software (Axon) was used for grid alignment and data extraction. Replicated miRNAs were averaged, and miRNAs whose intensities were ≥30 in all samples were chosen to calculate the normalization factor. After performing median normalization, significant differentially expressed miRNAs were identified using volcano plot filtering between the two experimental groups. The thresholds we used to identify up- or down-regulated miRNAs were fold change ≥1.5 and p-value ≤0.05. Finally, hierarchical clustering was performed using MEV software (v4.6, TIGR) to identify distinguishable miRNA expression profiling among the samples.

### miRNA Target Gene Prediction and Functional Analysis

Potential target genes of the differentially expressed miRNAs were predicted from data in the MicroCosm, miRanda, and miRDB databases using our proprietary database, and the final targets were integrated from these three public databases. Using this identified target gene set, the significant Gene Ontology (GO) classifications and Kyoto Encyclopedia Genes and Genomes (KEGG) pathways over-represented among these target genes from two of three databases were provided to obtain useful information regarding the functions of the targets. The Gene Ontology project, which covers the Biological Process, Cellular Component and Molecular Function domains, provides a controlled vocabulary to describe gene and gene product attributes in any organism. Pathway analysis is a functional analysis that maps genes to KEGG pathways. The p-value reflects the significance of GO term enrichment in the DE genes and the significance of the pathways correlated to the conditions (a p-value≤0.05 is recommended).

### Quantitative real-time PCR validation of the initial results

To validate the initial results detected by miRNA microarray in this study, 4 miRNAs were selected for an additional qRT-PCR analysis from hippocampus and peripheral blood samples. Total RNA was isolated using TRIzol reagent (Invitrogen Life Technologies, USA), and the RNA quality and quantity were measured using a NanoDrop ND-1000 spectrophotometer. Target cDNAs were amplified using RT-PCR (16°C for 30 min, 42°C for 40 min, and 85°C for 5 min) with a Gene Amp PCR System 9700 (Applied Biosystems). Each RT reaction mixture contained 5 μl Master Mix, 0.5 μl miR-RT primers F (10 μM), 0.5 μl miR-RT primers R (10 μM), and RNase-free H_2_O to a total volume of 8 μl, then 2 μl of the corresponding template cDNA was added. The RT-PCR reactions for U6 and for other miRNAs were performed using a ViiA 7 Real-time PCR System (Applied Biosystems) under the following conditions: 95°C for 10 min, followed by 40 PCR cycles (95°C for 10 s and then 60°C for 60 s), then the fluorescence intensity was measured. The real-time PCR reactions were performed for target miRNAs and for the internal reference (U6), with each sample analyzed in triplicate. The relative expression level for each miRNA was calculated using the comparative cycle threshold (CT) method (2^−ΔΔCT^).

### Statistical analysis

All data in this study are presented as the mean±SD. The Mann-Whitney U test was used for nonparametric inter-group comparisons between two groups. The escape latencies on the 5th day and the crossing times on the 6^th^ day in the MWM test were analysed by variance (ANOVA). All statistical analyses were performed using SPSS 17.0 software (SPSS, Inc., Chicago, IL, USA), and a two-tailed p-value of 0.05 was considered to indicate a significant difference.

## Results

### Animal kindling by PTZ

After the first kindling in response to PTZ (60 mg/kg) (i.p.), followed by 14 repeated kindling stimulations with 35 mg of PTZ/kg (i.p.) on every 48 hours, TLE models were successfully established in 52 of the 60 rats in the PTZ group, and memory behavior was evaluated using the MWM, which was performed 24 h after the last PTZ administration. Ten normal rats were also evaluated.

### Establishment of evaluation and grouping according to memory impairment in TLE rats using the MWM test

**Fig 1:**
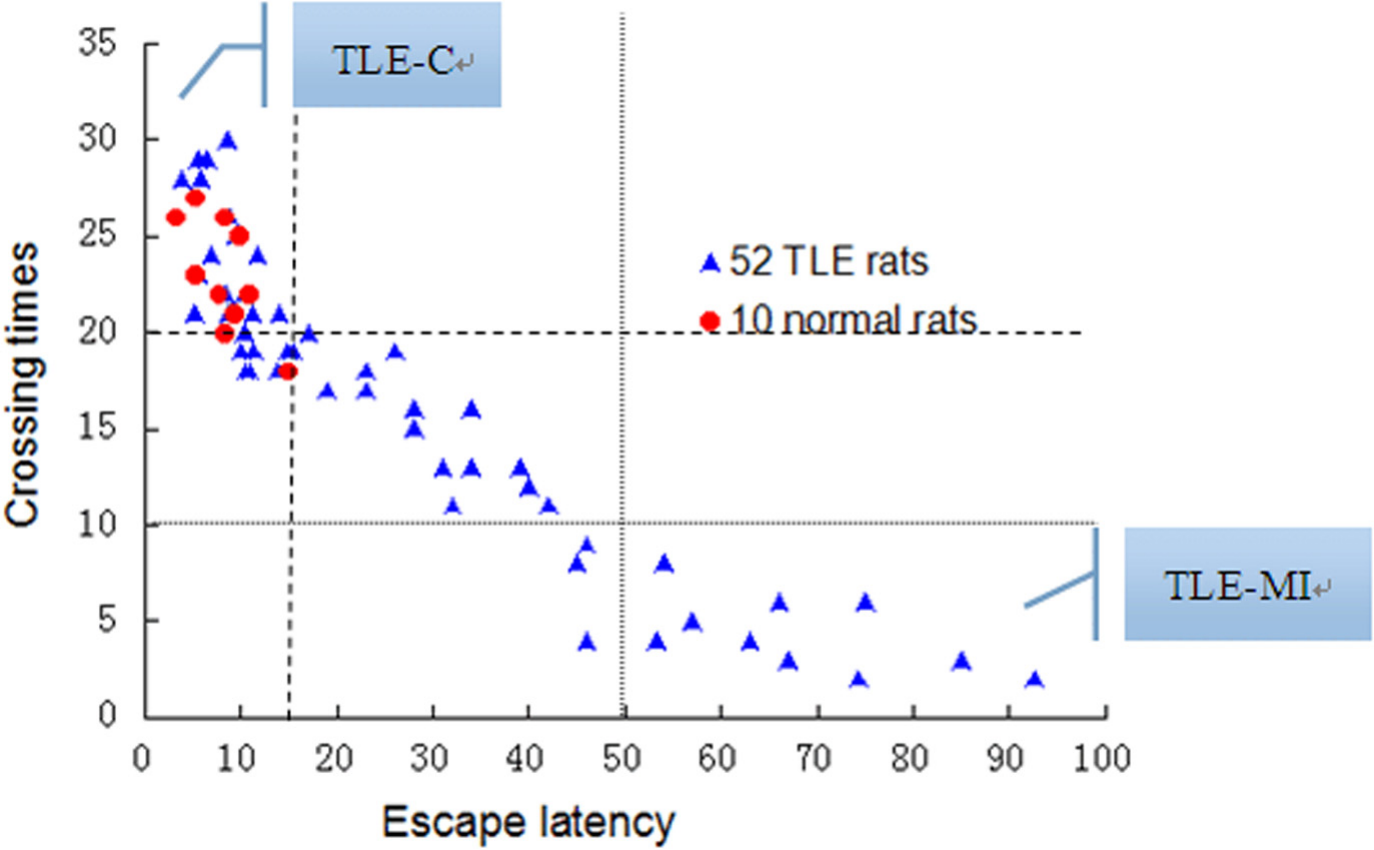
Scatterplot for all rats in the Morris water maze test and the grouping method according to memory impairment in epileptic rats. The red dots mark the 10 normal rats, and the blue dots mark the 52 TLE rats. In total, 17 TLE rats obtained overlapping scores of <15 s for the escape latency and >20 crossings in 400 s; these 17 rats were classified as the TLE control group with normal memory (TLE-C), and 10 TLE rats were classified as TLE rats with memory impairment (TLE-MI) due to their overlapping scores of >50 s for the escape latency and <10 crossings in 400 s.

In total, 17 TLE rats obtained scores of <15 s for escape latency on day 5 and >20 crossings in 400 s; these scores overlapped with the scores calculated for the 10 rats that underwent normal sham preconditioning and had normal memory function. Additionally, 10 rats with the worst performance in the MWM experiments obtained scores of >50 s for the escape latency on day 5 and <10 crossings in 400 s. These rats were classified as exhibiting memory impairment according to the grouping method applied to the 52 TLE rats, representing an incidence of 19.23% (10/52) memory impairment. The evaluation and grouping of memory-impaired TEL rats using the MWM test are shown in Fig 1.

### miRNA microarray analysis

The 7th generation Exiqon microRNA arrays used in this study contained 3100 Tm-normalized LNA-enhanced capture probes, annotated in the miRBase 18.0 sequence database and offering great reproducibility, with 99% correlation between arrays and a dynamic range that exceeds 5 orders of magnitude. The miRNA microarray yielded 11 significantly dysregulated miRNAs(Table 1). Among the 11 significantly dysregulated miRNAs, 4 miRNAs were up-regulated (miR-34c, miR-374, miR-181a, and miR-let-7c-1), and 7 miRNAs were down-regulated (miR-1188, miR-770-5p, miR-127-5p, miR-375, miR-331, miR-873-5p, and miR-328a) (differentially expressed miRNAs were defined by a fold-change >1.5, up or down-regulated; p <0.05).

**Table 1:** The 11 significantly dysregulated miRNAs in the hippocampus of memory-impaired TLE rats.

ID	microRNA	Fold change	Regulation	P-value
42767	rno-miR-34c-3p	1.524517816	up	9.056E-04
148025	rno-miR-374-3p	1.703549498	up	1.515E-02
148340	rno-miR-181a-2-3p	1.52586174	up	1.167E-02
42668	rno-let-7c-1-3p	1.517090485	up	2.813E-02
148417	rno-miR-1188-3p	0.663742737	down	1.321E-02
42817	rno-miR-770-5p	0.587089906	down	3.482E-03
42692	rno-miR-127-5p	0.52301628	down	2.390E-03
46918	rno-miR-375-3p	0.630027837	down	4.320E-02
42887	rno-miR-331-3p	0.601332071	down	3.012E-02
17917	rno-miR-873-5p	0.635312844	down	4.125E-02
145640	rno-miR-328a-3p	0.597376333	down	1.286E-03

**Fig 2:**
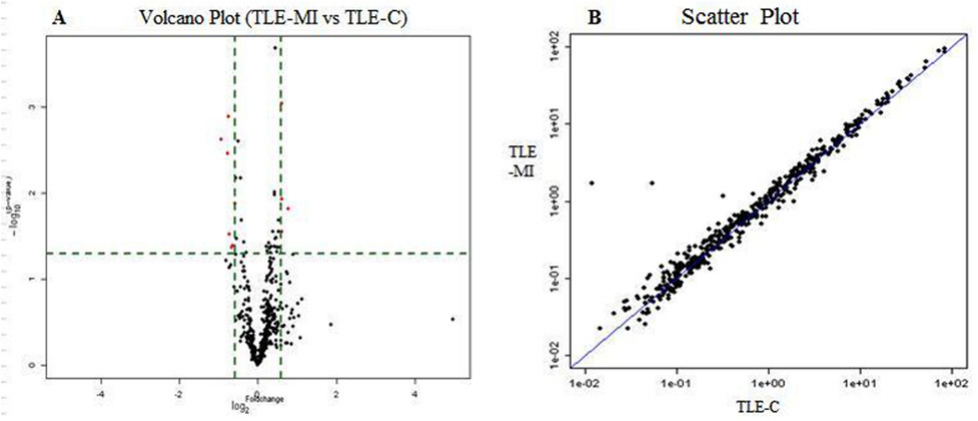
Volcano plot (A) and scatter plot (B) of the miRNA microarray analysis. (A) Volcano plots are a useful tool for visualizing differential expression patterns between two conditions. The vertical lines correspond to 1.5-fold up- and down-regulation, and the horizontal line represents a p-value of 0.05. Thus, the red point in the plot represents the differentially expressed miRNAs that reached significance. (B) The scatter plot is a useful visualization for assessing the variation (or reproducibility) between chips. The axes of the scatter plot are the normalized signal values of the samples (the ratio scale).

**Fig 3:**
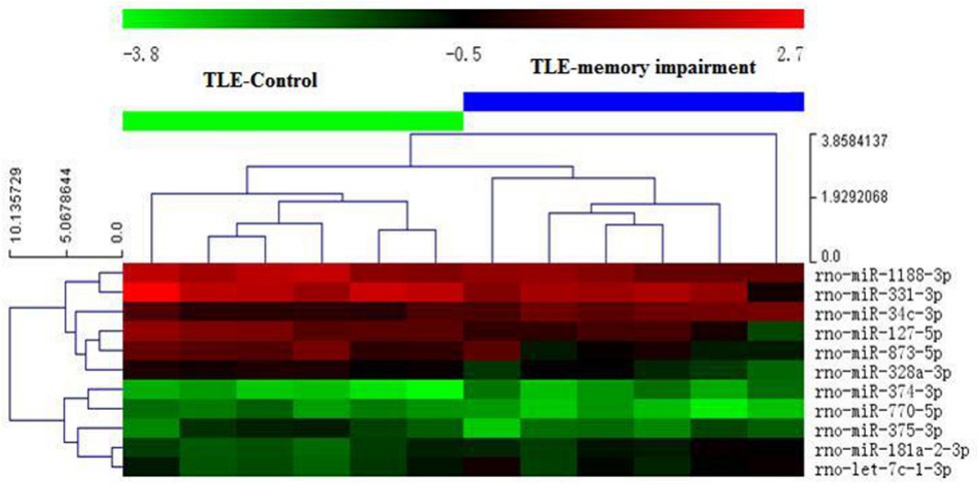
Hierarchical clustering for differentially expressed miRNAs (pass volcano plot) in TLE-MI compared with TLE-C animals. Left, the miRNA clustering tree; top, the sample clustering tree. Red indicates high relative expression, and green indicates low relative expression.

A scatter plot was generated to assess the quality of the miRNA data after filtering, and a volcano plot was generated to visualize the differential expression between two different conditions, as shown in Fig 2. The cluster and heatmaps of all detected rat miRNAs were plotted to better demonstrate the dysregulated miRNAs between the control and memory-impaired TLE rats, as shown in Fig 3. All differentially expressed miRNAs in the normalized primary microarray data are shown in Supporting Information S1 Table.

### Quantitative real-time PCR results

**Fig 4:**
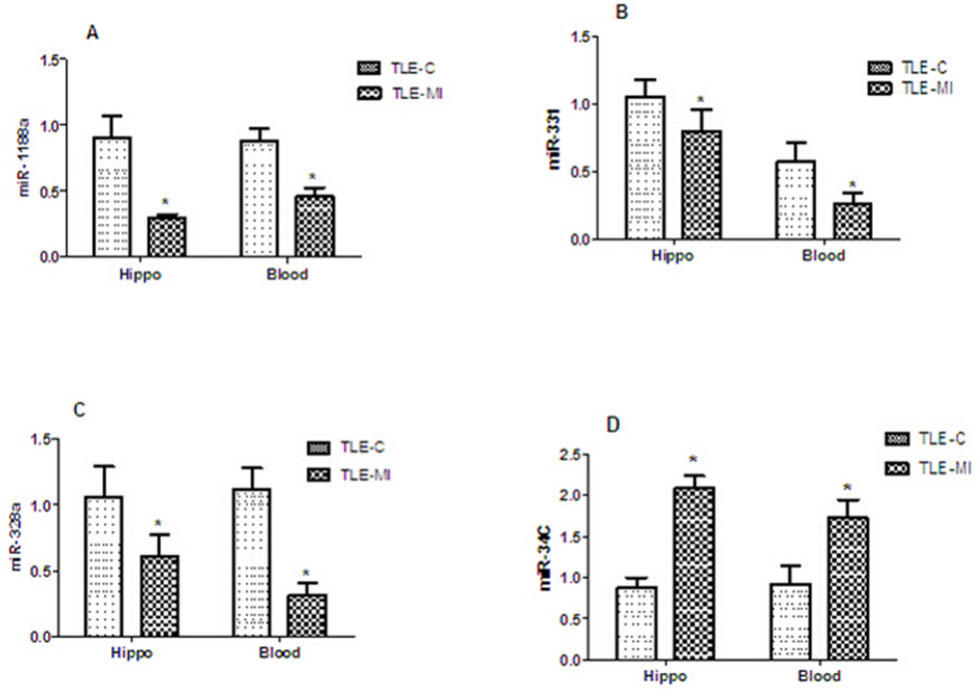
qRT-PCR validation of four differentially expressed miRNAs in the hippocampus (Hippo) and in the peripheral blood (Blood). (A) miR-1188a expression. (B) miR-331 expression. (C) miR-375 expression. Hippocampus and blood expression levels of miR-1188a, miR-331 and miR-375 decreased in memory-impaired rats (TLE-MI) compared with control rats (TEL-C). (D) miR-34c expression. Hippocampus and blood expression levels of miR-34c increased in TLE-MI rats compared with those in TEL-C rats (the values are presented as the mean±SEM, *p<0.05; n = 6 per group).

The dysregulated miRNAs (miR-34c, miR-1188a, miR-328a, and miR-331) were confirmed using qRT-PCR and the results were consistent with the microarray analysis. According to the PCR results, the miR-34c expression levels were up-regulated, whereas the miR-1188a, miR-328a and miR-331 expression levels were down-regulated (*p<0.05) (Fig 4 A–4 C, left), and miR-1188a presented the most pronounced changes in expression in the hippocampus, which is consistent with the microarray findings.

Furthermore, miRNAs from peripheral blood of the TLE-MI and TLE-C rats were also analyzed using qRT-PCR. As observed in our data, the peripheral blood expression levels of miR-1188a, miR-328, and miR-331 decreased in TLE-MI rats (Fig 4 A–4 C, right), whereas the expression level of miR-34c increased (Fig 4 D, right) in TLE-MI rats, suggesting that the expression pattern trends were identical to those of their counterparts in the hippocampus. The quantitative real-time PCR results for peripheral blood and hippocampal tissue are shown in Fig 4.

### Bioinformatics analysis

**Fig 5:**
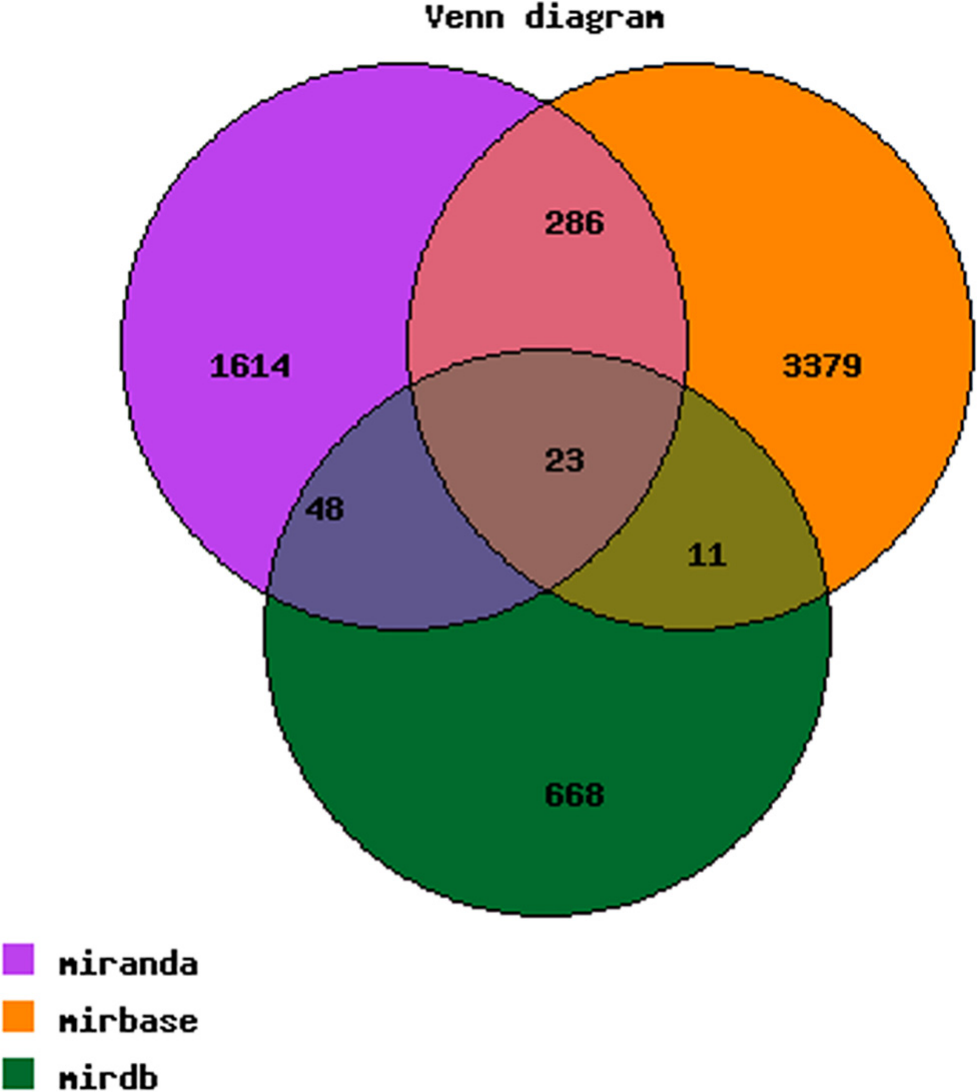
The Arraystar proprietary miRNA target database.

Based on the data from the miRanda, MicroCosm, and miRDB databases within our proprietary database, 1971, 3699, and 750 predicted target genes of the 11 dysregulated miRNAs were identified in the miRanda, MicroCosm and miRDB databases, respectively, with 345 miRNA target genes integrated from two of these three databases and 23 target genes integrated from all three databases (Table 2, Fig 5). All target genes of the differentially expressed miRNAs in the three datebases are shown in Supporting Information S2 Table. Five GO terms were significantly over-represented in the dysregulated miRNA targets under the domains Biological Process, Cellular Component and Molecular Function, as shown in Table 3. The detailed pathway analysis data for the top 10 pathways associated with the 11 dysregulated miRNA targets, including the pathway title, the gene numbers in the pathway and the p-value, are shown in Table 4. The signaling pathways enriched in the TLE-MI rats were consistent with the molecular portrait of latent epilepsy mechanisms and memory dysfunctions presented in recent research[24,30].

**Table 2:** The 23 target genes of significantly dysregulated miRNAs by integrating from all 3 databases.

miRNA_name	Genesymbol	miranda	mirbase	mirdb	num_statistcs
rno-miR-328a-3p	Arid4b	1	1	1	3
rno-miR-328a-3p	Nr3c1	1	1	1	3
rno-miR-328a-3p	Slc2a1	1	1	1	3
rno-miR-331-3p	Dgat1	1	1	1	3
rno-miR-331-3p	Eif2s1	1	1	1	3
rno-miR-331-3p	Inppl1	1	1	1	3
rno-miR-331-3p	Itm2c	1	1	1	3
rno-miR-331-3p	Ptp4a1	1	1	1	3
rno-miR-34c-3p	Kif22	1	1	1	3
rno-miR-34c-3p	Lias	1	1	1	3
rno-miR-34c-3p	Sfrp4	1	1	1	3
rno-miR-375-3p	Tor1aip2	1	1	1	3
rno-miR-873-5p	Cx3cr1	1	1	1	3
rno-miR-873-5p	Donson	1	1	1	3
rno-miR-873-5p	Hr	1	1	1	3
rno-miR-873-5p	Hyal2	1	1	1	3
rno-miR-873-5p	MGC114246	1	1	1	3
rno-miR-873-5p	Nuak2	1	1	1	3
rno-miR-873-5p	Plcl1	1	1	1	3
rno-miR-873-5p	Ptgs1	1	1	1	3
rno-miR-873-5p	Reg3a	1	1	1	3
rno-miR-873-5p	Slc25a3	1	1	1	3
rno-miR-873-5p	Sms	1	1	1	3

**Table 3:** The top 5 GO terms significantly over-represented in three separate domains.

Term	DE Count	Fold Enrichment	P-value	Ontology
cell	2426	1.146237796	2.18E-62	Cellular component
cell part	2424	1.146550867	2.59E-62	Cellular component
cytoplasm	1688	1.252563455	2.47E-47	Cellular component
intracellular part	2080	1.175778541	2.93E-45	Cellular component
membrane-bound organelle	1653	1.232473425	5.63E-40	Cellular component
localization	848	1.390424136	4.86E-33	Biological process
transport	700	1.459650585	5.16E-33	Biological process
establishment of localization	705	1.444730511	9.62E-32	Biological process
system development	768	1.397938001	5.78E-30	Biological process
multicellular organismal development	832	1.355437261	4.75E-28	Biological process
protein binding	1133	1.441278341	2.12E-58	Molecular function
binding	1902	1.203459813	1.16E-52	Molecular function
enzyme binding	294	1.593995386	2.38E-18	Molecular function
small molecule binding	494	1.3207609	4.30E-13	Molecular function
transmembrane transporter activity	221	1.536676019	3.41E-12	Molecular function

**Table 4:** The top 10 pathways of the 11 dysregulated miRNA targets.

Pathway ID	*Rattus norvegicus* (rat) pathway	Genes in pathway	Fisher P-value
rno04010	MAPK signaling pathway	81	1.44E-07
rno04728	Dopaminergic synapse	48	4.49E-07
rno05031	Amphetamine addiction	29	6.98E-07
rno04668	TNF signaling pathway	40	1.22E-06
rno04261	Adrenergic signaling in cardiomyocytes	49	1.01E-05
rno04020	Calcium signaling pathway	57	1.98E-05
rno05030	Cocaine addiction	21	3.12E-05
rno04114	Oocyte meiosis	38	3.20E-05
rno04390	Hippo signaling pathway	48	6.01E-05
rno05200	Pathways in cancer	86	6.14E-05

## Discussion

This study is the first attempt to establish criteria for memory impairment in TLE rats using the MWM test. Approximately 40% of patients with epilepsy suffer from cognitive and behavioral deficits[36]. Additionally, patients with epilepsy rank cognitive dysfunction as the worst problem that they experienced[37]. Memory deficits in TLE are thought to be caused by clinical or subclinical seizure activity, structural abnormalities, adverse effects of anticonvulsant medication, psychological mechanisms[38]or other underlying brain pathologies, such as mitochondrial dysfunction or inflammatory effects of seizure activity[39]. However, complaints regarding memory decline commonly do not match test performance because no standard neuropsychological tests to detect the memory performance associated with epilepsy have been established for either patients or animal models[40].

Evidence that deficits are more severe in patients with chronic TLE compared with newly diagnosed patients has been reported[41], with a demonstrated high frequency of tonic-clonic seizures and a long chronic course acting as the strongest predictors of cognitive decline and of the severity of memory impairments in these patients. We therefore improved the PTZ kindling model with a single intraperitoneal (i.p.) injection of PTZ (60 mg/kg) to trigger seizures, followed by repeated kindling (35 mg/kg) on alternating days (14 injections total) to mimic human epileptic seizures. This newly developed TLE rat model was not influenced by anticonvulsant medication, by a different form of convulsion or by social factors and induced mild cognitive impairment associated with epilepsy for further study. The MWM was used in our study to detect the visuospatial memory functions and to form groups of TLE rats according to their performance in the place navigation test and in the spatial probe test. As indicated in the results (Fig 1), most of the TLE rats (17/52) exhibited good memory with performance that was consistent with that of normal rats; only 19.23% (10/52) of the TLE rats who exhibited poor performance on the MWM tests were classified as TLE-MI rats, and the remaining 25 TLE rats were excluded.

Our study found that distinct patterns of miRNA expression were observed in the TLE rats with memory disorders compared with those rats whose memory was not affected by TLE treatment. miRNAs are predicted to mediate protein-coding genes and to regulate protein levels via binding to complementary sequences in the 3′-untranslated region (UTR) of their target mRNAs in mammals[42]. miRNAs play an important role in normal brain function and in the development of the central nervous system (CNS) by inducing post-transcriptional gene silencing[19]. Additionally, dysregulation of miRNAs may play a role in neurological and neurodegenerative diseases, including Alzheimer’s disease, ischemic tolerance and epileptic brain injury[20–23]. Recently, several studies demonstrated that miRNAs might be involved in memory by controlling synapse function and plasticity in vertebrates[16] and might offer a highly effective means of protein expression by responding to neuronal activity during memory formation[43]. The underlying molecular mechanisms of TLE remain unknown, and an aberrant miRNA expression pattern most likely underlies this pathological condition. Because the PTZ rat model remains one of the established classical animal models for cognitive function research in epilepsy and TaqMan RT-PCR miRNA assays are recognized as sensitive and accurate method, we demonstrated in the present study that the miRNA expression pattern in the rat hippocampus was disturbed during memory disorders. Several miRNAs were significantly up-regulated or down-regulated (P<0.05) more than 1.5-fold in the hippocampus and peripheral blood in TLE rats with memory disorders.

For many neurological diseases, the efficacy and outcome of treatment depend on early detection. In our study, the results provide a partial mechanism for specific miRNA-induced mRNA expression profiles in the brain and demonstrate the potential of blood miRNAs to serve as biomarkers in the absence of direct access to diseased tissue for clinical diagnosis and prognosis in patients with memory dysfunction, as well as other CNS malignancies, neurological, and psychiatric diseases. Some of the up-regulated (miR-34c, miR-374, miR-181a, and miR-let-7c-1) and down-regulated (miR-1188, miR-770-5p, miR-127-5p, miR-375, miR-331, miR-873-5p, and miR-328a) miRNAs we detected using miRNA microarray were suggested to be closely connected with memory function. For example, miR-34c has been proven to play an important role in cellular proliferation and apoptosis by regulating the expression of several genes; this transcript also shows increased expression levels in both PBMC and plasma in AD patients compared with those of age-matched normal controls[44]. The expression of miR-328, which is a translational regulator of ß-amyloid (Aß) precursor protein (APP)-converting enzyme (BACE), was found to be decreased in the hippocampi of aging APPSwe/PS1 mice[45]. Let-7c miRNA was reported to play an important role in the regulation of androgen signaling by down-regulating androgen receptor expression[46]. Inhibiting miR-181a was proved to be correlated with the protection of 10 μmol/L propofol against GD stress in astrocytes by up-regulating Bcl-2 protein expression[47].

**Fig 6:**
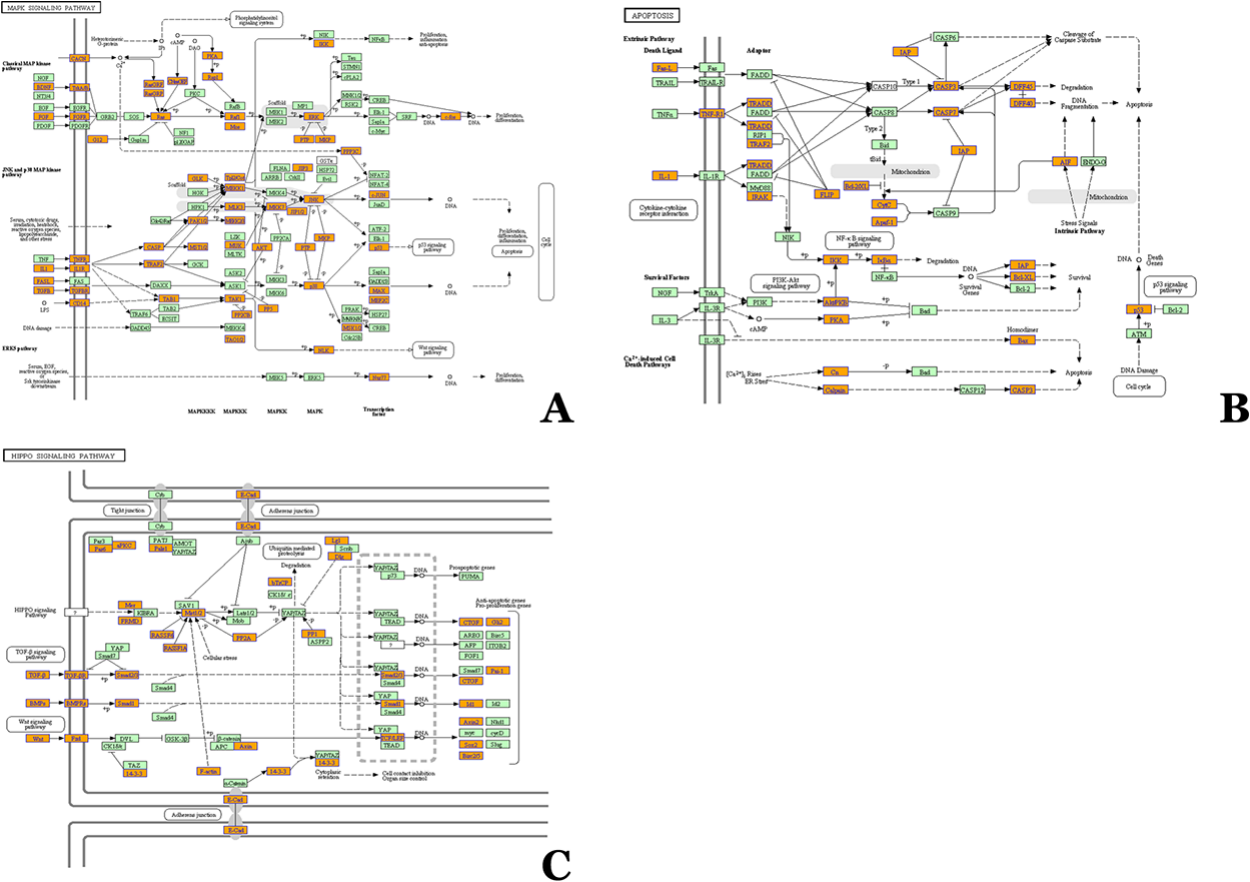
The pathways enriched in the MAPK signaling pathway (A), apoptosis pathway (B) and hippocampal signaling pathway (C).

Reliable miRNA target information remains limited, and integrating miRNA targets from the miRDB, MicroCosm and miRanda databases appears to be a more reliable and relevant approach compared with using information from only one database. According to the KEGG pathway analysis regarding over-represented target genes, we found a large pool of pathways enriched in the MAPK signaling pathway, apoptosis pathway and hippocampal signaling pathway (Fig 6), which are important components in the pathogenesis of several neurological and neurodegenerative diseases, and the detailed internal mechanism requires further study.

## Supporting Information

S1 Table All differentially expressed miRNAs in normalized primary microarray data. (PDF)

S2 Table All target genes of the differentially expressed miRNAs in the three datebases. (PDF)
