# Supplementary material for: Grouping Pentylenetetrazol-Induced Epileptic Rats According to Memory Impairment and MicroRNA Expression Profiles in the Hippocampus
Source: PLoS One. 2015 May 11;10(5):e0126123. doi: 10.1371/journal.pone.0126123 (PMC4427457; doi:10.1371/journal.pone.0126123)
Supplement: S1 Table — (PDF) [file pone.0126123.s001.pdf]

S1 Table. All differentially expressed miRNAs in normalized primary microarray data.

ID	microRNA	Fold change	P-value	Regulation
148152	rno-miR-218b	2.064785	0.266603	up
11113	rno-miR-448-3p	1.676536	0.553317	up
46381	rno-miR-1298	1.815159	0.571066	up
148611	rno-miR-3576	1.503087	0.114742	up
148025	rno-miR-374-3p	1.703549	0.015151	up
17835	rno-miR-450a-5p	1.583394	0.347427	up
10977	rno-miR-183-5p	3.625202	0.336695	up
145705	rno-miR-431	1.755072	0.288	up
148089	rno-miR-208a-3p	1.858046	0.356687	up
148066	rno-miR-216b-3p	1.752258	0.109169	up
148294	rno-miR-217-3p	1.647148	0.081971	up
42568	rno-miR-880-3p	1.846928	0.051663	up
148436	rno-miR-3584-5p	1.813024	0.252382	up
10975	rno-miR-182	31.10161	0.294697	up
19601	rno-miR-211-5p	2.103366	0.476187	up
148497	rno-miR-3573-5p	1.683704	0.086854	up
11005	rno-miR-204-5p	1.634496	0.559808	up
146194	rno-miR-628	1.835271	0.228178	up
148050	rno-miR-3558-3p	1.552541	0.220467	up
46219	rno-miR-1306-5p	1.506754	0.515999	up
29153	rno-miR-34b-5p	1.502375	0.596251	up
148455	rno-miR-741-3p	2.036752	0.194266	up
13147	rno-miR-96-5p	144.5358	0.389977	up
11210	rno-miR-215	1.524653	0.356663	up
148340	rno-miR-181a-2-3p	1.525862	0.011671	up

148334	rno-miR-122-3p	1.647741	0.209159	up
14294	rno-miR-1-3p	2.159723	0.171166	up
42472	rno-miR-190b-5p	1.510302	0.187434	up
148093	rno-miR-1912-3p	1.56448	0.351209	up
148512	rno-miR-187-5p	1.880856	0.281963	up
148211	rno-miR-3590-3p	1.697956	0.423052	up
42668	rno-let-7c-1-3p	1.51709	0.028132	up
146078	rno-miR-632	0.655834	0.124672	down
145970	rno-miR-129-2-3p	0.603946	0.073393	down
148417	rno-miR-1188-3p	0.663743	0.013208	down
42817	rno-miR-770-5p	0.58709	0.003483	down
148645	rno-miR-129-5p	0.624192	0.068552	down
148314	rno-miR-873-3p	0.566322	0.061542	down
42692	rno-miR-127-5p	0.523016	0.00239	down
46918	rno-miR-375-3p	0.630028	0.043204	down
42887	rno-miR-331-3p	0.601332	0.030116	down
17917	rno-miR-873-5p	0.635313	0.041253	down
145640	rno-miR-328a-3p	0.597376	0.001286	down
