# Supplementary material for: Grouping Pentylenetetrazol-Induced Epileptic Rats According to Memory Impairment and MicroRNA Expression Profiles in the Hippocampus
Source: PLoS One. 2015 May 11;10(5):e0126123. doi: 10.1371/journal.pone.0126123 (PMC4427457; doi:10.1371/journal.pone.0126123)
Supplement: S2 Table — (PDF) [file pone.0126123.s002.pdf]

S2 Table. All target genes of the differentially expressed miRNAs in the three datebases.

mirna_name	genesymbol	miranda	mirbase	mirdb	num_statistcs
rno-miR-328a-3p	Arid4b	1	1	1	3
rno-miR-328a-3p	Nr3c1	1	1	1	3
rno-miR-328a-3p	Slc2a1	1	1	1	3
rno-miR-331-3p	Dgat1	1	1	1	3
rno-miR-331-3p	Eif2s1	1	1	1	3
rno-miR-331-3p	Inppl1	1	1	1	3
rno-miR-331-3p	Itm2c	1	1	1	3
rno-miR-331-3p	Ptp4a1	1	1	1	3
rno-miR-34c-3p	Kif22	1	1	1	3
rno-miR-34c-3p	Lias	1	1	1	3
rno-miR-34c-3p	Sfrp4	1	1	1	3
rno-miR-375-3p	Tor1aip2	1	1	1	3
rno-miR-873-5p	Cx3cr1	1	1	1	3
rno-miR-873-5p	Donson	1	1	1	3
rno-miR-873-5p	Hr	1	1	1	3
rno-miR-873-5p	Hyal2	1	1	1	3
rno-miR-873-5p	MGC114246	1	1	1	3
rno-miR-873-5p	Nuak2	1	1	1	3
rno-miR-873-5p	Plcl1	1	1	1	3
rno-miR-873-5p	Ptgs1	1	1	1	3
rno-miR-873-5p	Reg3a	1	1	1	3
rno-miR-873-5p	Slc25a3	1	1	1	3
rno-miR-873-5p	Sms	1	1	1	3
rno-miR-328a-3p	Ablim2	1	1	0	2
rno-miR-328a-3p	Aldoal1	1	1	0	2
rno-miR-328a-3p	Aldoc	1	1	0	2
rno-miR-328a-3p	Apln	1	0	1	2
rno-miR-328a-3p	Arfgef2	1	1	0	2
rno-miR-328a-3p	Arfip1	1	1	0	2
rno-miR-328a-3p	Arhgap17	1	1	0	2
rno-miR-328a-3p	Atp6v1e1	1	1	0	2
rno-miR-328a-3p	Bcl2l2	1	0	1	2
rno-miR-328a-3p	Bles03	1	1	0	2
rno-miR-328a-3p	Cacna1h	1	1	0	2
rno-miR-328a-3p	Cav3	1	1	0	2
rno-miR-328a-3p	Cd37	1	1	0	2
rno-miR-328a-3p	Cdipt	1	1	0	2
rno-miR-328a-3p	Cntn2	1	1	0	2
rno-miR-328a-3p	Cpt1a	1	0	1	2
rno-miR-328a-3p	Dnah1	1	1	0	2
rno-miR-328a-3p	Ebp	1	1	0	2
rno-miR-328a-3p	Fez1	1	1	0	2
rno-miR-328a-3p	Fgf9	1	1	0	2
rno-miR-328a-3p	Gabrd	1	1	0	2
rno-miR-328a-3p	Gnb2	1	1	0	2
rno-miR-328a-3p	Grpel1	1	1	0	2
rno-miR-328a-3p	Hfe2	1	1	0	2

rno-miR-328a-3p	Hist1h4b	1	0	1	2
rno-miR-328a-3p	Id4	0	1	1	2
rno-miR-328a-3p	Ino80c	1	0	1	2
rno-miR-328a-3p	Inpp4b	1	0	1	2
rno-miR-328a-3p	Kcnj9	1	1	0	2
rno-miR-328a-3p	Klhdc3	1	1	0	2
rno-miR-328a-3p	Lgr4	1	0	1	2
rno-miR-328a-3p	LOC499886	1	1	0	2
rno-miR-328a-3p	Mapre1	1	1	0	2
rno-miR-328a-3p	Mcf2l	1	1	0	2
rno-miR-328a-3p	Men1	1	1	0	2
rno-miR-328a-3p	Mtmr3	1	1	0	2
rno-miR-328a-3p	Napsa	1	1	0	2
rno-miR-328a-3p	Nr1i3	1	1	0	2
rno-miR-328a-3p	Nr2f6	1	1	0	2
rno-miR-328a-3p	Nucb1	1	1	0	2
rno-miR-328a-3p	Pax8	1	1	0	2
rno-miR-328a-3p	Pcsk9	1	1	0	2
rno-miR-328a-3p	Pde2a	1	1	0	2
rno-miR-328a-3p	Pdgfrl	1	1	0	2
rno-miR-328a-3p	Phc2	0	1	1	2
rno-miR-328a-3p	Phf23	0	1	1	2
rno-miR-328a-3p	Plce1	1	1	0	2
rno-miR-328a-3p	Prpf19	1	1	0	2
rno-miR-328a-3p	Ptpn9	1	1	0	2
rno-miR-328a-3p	RGD1305215	1	1	0	2
rno-miR-328a-3p	RGD1311946	1	1	0	2
rno-miR-328a-3p	Rgs14	1	1	0	2
rno-miR-328a-3p	Rtkn	1	1	0	2
rno-miR-328a-3p	S100a3	1	1	0	2
rno-miR-328a-3p	Shisa5	1	0	1	2
rno-miR-328a-3p	Slc6a3	1	1	0	2
rno-miR-328a-3p	Tfip11	1	1	0	2
rno-miR-328a-3p	Tp53	1	1	0	2
rno-miR-328a-3p	Trim39	1	0	1	2
rno-miR-328a-3p	Unc13d	1	1	0	2
rno-miR-328a-3p	Ywhaz	1	0	1	2
rno-miR-331-3p	Ap1b1	1	1	0	2
rno-miR-331-3p	Aqp3	1	1	0	2
rno-miR-331-3p	Arf5	1	1	0	2
rno-miR-331-3p	Arhgef1	1	1	0	2
rno-miR-331-3p	Asb6	1	1	0	2
rno-miR-331-3p	Bzw2	1	1	0	2
rno-miR-331-3p	Cabc1	1	1	0	2
rno-miR-331-3p	Car11	1	1	0	2
rno-miR-331-3p	Cask	1	1	0	2
rno-miR-331-3p	Cdc42ep2	1	1	0	2
rno-miR-331-3p	Celsr3	1	1	0	2
rno-miR-331-3p	Cyb561d2	1	1	0	2
rno-miR-331-3p	Dnajc7	1	1	0	2
rno-miR-331-3p	Dusp1	1	1	0	2

rno-miR-331-3p	Egr3	1	1	0	2
rno-miR-331-3p	Ell3	1	1	0	2
rno-miR-331-3p	Fam101b	1	0	1	2
rno-miR-331-3p	Fbxo17	1	1	0	2
rno-miR-331-3p	Fut7	1	1	0	2
rno-miR-331-3p	Fxyd6	1	1	0	2
rno-miR-331-3p	Gjb4	1	1	0	2
rno-miR-331-3p	Gli1	0	1	1	2
rno-miR-331-3p	Gls2	1	1	0	2
rno-miR-331-3p	Gnb2	1	1	0	2
rno-miR-331-3p	Gne	1	1	0	2
rno-miR-331-3p	Gss	1	1	0	2
rno-miR-331-3p	Id1	1	1	0	2
rno-miR-331-3p	Ier3	1	1	0	2
rno-miR-331-3p	Ikbkb	1	1	0	2
rno-miR-331-3p	Il1b	1	1	0	2
rno-miR-331-3p	Impa2	1	1	0	2
rno-miR-331-3p	Lgals7	1	1	0	2
rno-miR-331-3p	LOC314140	0	1	1	2
rno-miR-331-3p	LOC361914	1	1	0	2
rno-miR-331-3p	LOC498368	1	1	0	2
rno-miR-331-3p	Mafk	1	0	1	2
rno-miR-331-3p	Mapkap1	1	1	0	2
rno-miR-331-3p	Msln	1	1	0	2
rno-miR-331-3p	Mxd3	1	1	0	2
rno-miR-331-3p	Nme3	1	1	0	2
rno-miR-331-3p	Panx2	1	1	0	2
rno-miR-331-3p	Phf1	1	1	0	2
rno-miR-331-3p	Phgdh	1	1	0	2
rno-miR-331-3p	Plek	1	1	0	2
rno-miR-331-3p	Polr2c	1	1	0	2
rno-miR-331-3p	Pon2	1	0	1	2
rno-miR-331-3p	Rangap1	1	1	0	2
rno-miR-331-3p	Rara	1	1	0	2
rno-miR-331-3p	Rbp2	1	1	0	2
rno-miR-331-3p	Rcor2	1	1	0	2
rno-miR-331-3p	Reep4	1	1	0	2
rno-miR-331-3p	RGD1307315	1	1	0	2
rno-miR-331-3p	RGD1311899	1	1	0	2
rno-miR-331-3p	Ric8b	1	1	0	2
rno-miR-331-3p	Rims3	1	1	0	2
rno-miR-331-3p	Rpusd4	1	1	0	2
rno-miR-331-3p	Samm50	1	1	0	2
rno-miR-331-3p	Smad1	1	0	1	2
rno-miR-331-3p	Snx24	1	1	0	2
rno-miR-331-3p	Tap2	1	1	0	2
rno-miR-331-3p	Tcp11	1	1	0	2
rno-miR-331-3p	Th	1	1	0	2
rno-miR-331-3p	Ube2n	1	1	0	2
rno-miR-331-3p	Uhmk1	1	1	0	2
rno-miR-331-3p	Usf1	1	1	0	2

rno-miR-331-3p	Vcp	1	1	0	2
rno-miR-331-3p	Vgf	1	1	0	2
rno-miR-331-3p	Vps16	1	1	0	2
rno-miR-331-3p	Vps45	1	1	0	2
rno-miR-331-3p	Ypel4	1	1	0	2
rno-miR-331-3p	Zap70	1	1	0	2
rno-miR-331-3p	Zfp513	0	1	1	2
rno-miR-34c-3p	Actrt2	1	1	0	2
rno-miR-34c-3p	Ahrr	1	0	1	2
rno-miR-34c-3p	Arhgap15	1	0	1	2
rno-miR-34c-3p	Aurkb	1	1	0	2
rno-miR-34c-3p	C6	1	1	0	2
rno-miR-34c-3p	Camk4	1	1	0	2
rno-miR-34c-3p	Cask	1	1	0	2
rno-miR-34c-3p	Casq2	1	1	0	2
rno-miR-34c-3p	Cckbr	1	1	0	2
rno-miR-34c-3p	Cd47	1	1	0	2
rno-miR-34c-3p	Chm	1	1	0	2
rno-miR-34c-3p	Cln8	1	0	1	2
rno-miR-34c-3p	Cth	1	0	1	2
rno-miR-34c-3p	Cyp4f5	1	1	0	2
rno-miR-34c-3p	Ddn	1	1	0	2
rno-miR-34c-3p	Eif4e	1	0	1	2
rno-miR-34c-3p	Fkbp1a	1	1	0	2
rno-miR-34c-3p	Ggh	1	1	0	2
rno-miR-34c-3p	Gja4	1	1	0	2
rno-miR-34c-3p	Gpm6a	1	0	1	2
rno-miR-34c-3p	Gtf2b	1	1	0	2
rno-miR-34c-3p	Hars2l	1	0	1	2
rno-miR-34c-3p	Hfe2	1	1	0	2
rno-miR-34c-3p	Insig1	1	1	0	2
rno-miR-34c-3p	Kazald1	1	1	0	2
rno-miR-34c-3p	Klf11	1	0	1	2
rno-miR-34c-3p	Lamp3	1	1	0	2
rno-miR-34c-3p	Ly6b	1	1	0	2
rno-miR-34c-3p	Marcksl1	1	1	0	2
rno-miR-34c-3p	MGC108823	1	1	0	2
rno-miR-34c-3p	MGC114520	1	0	1	2
rno-miR-34c-3p	Ostn	1	1	0	2
rno-miR-34c-3p	Paics	1	1	0	2
rno-miR-34c-3p	Panx3	1	0	1	2
rno-miR-34c-3p	Pir	1	1	0	2
rno-miR-34c-3p	Prom1	1	1	0	2
rno-miR-34c-3p	Prss16	1	1	0	2
rno-miR-34c-3p	RGD1306991	1	1	0	2
rno-miR-34c-3p	Rnf12	1	1	0	2
rno-miR-34c-3p	Serpina5	1	1	0	2
rno-miR-34c-3p	Serpinf2	1	1	0	2
rno-miR-34c-3p	Slc7a3	1	1	0	2
rno-miR-34c-3p	Smcp	1	1	0	2
rno-miR-34c-3p	Smgc	1	1	0	2

rno-miR-34c-3p	Sord	1	1	0	2
rno-miR-34c-3p	Taldo1	1	1	0	2
rno-miR-34c-3p	Tmem27	1	1	0	2
rno-miR-34c-3p	Tmem33	1	1	0	2
rno-miR-34c-3p	Wrnip1	1	1	0	2
rno-miR-375-3p	Aadat	1	1	0	2
rno-miR-375-3p	Adra1d	1	1	0	2
rno-miR-375-3p	Ahr	1	1	0	2
rno-miR-375-3p	Arf1	1	1	0	2
rno-miR-375-3p	Atp2b1	1	1	0	2
rno-miR-375-3p	Bmp3	1	1	0	2
rno-miR-375-3p	C1qbp	1	1	0	2
rno-miR-375-3p	Copb1	1	1	0	2
rno-miR-375-3p	Ctgf	1	1	0	2
rno-miR-375-3p	Elavl2	1	1	0	2
rno-miR-375-3p	Elavl4	0	1	1	2
rno-miR-375-3p	Esrrg	1	1	0	2
rno-miR-375-3p	Fmo4	1	1	0	2
rno-miR-375-3p	Foxe1	1	1	0	2
rno-miR-375-3p	Gpc2	1	1	0	2
rno-miR-375-3p	Grik5	1	1	0	2
rno-miR-375-3p	Hdmcp	1	1	0	2
rno-miR-375-3p	LOC317471	1	1	0	2
rno-miR-375-3p	LOC494499	1	1	0	2
rno-miR-375-3p	LOC500445	1	1	0	2
rno-miR-375-3p	Naaa	1	0	1	2
rno-miR-375-3p	Nfe2l2	1	1	0	2
rno-miR-375-3p	Notch1	0	1	1	2
rno-miR-375-3p	Nox4	1	1	0	2
rno-miR-375-3p	Nudt9	1	1	0	2
rno-miR-375-3p	Pacsin2	1	1	0	2
rno-miR-375-3p	Pdha1	1	1	0	2
rno-miR-375-3p	Plekha3	1	1	0	2
rno-miR-375-3p	Ppp2r2a	0	1	1	2
rno-miR-375-3p	Psen1	1	1	0	2
rno-miR-375-3p	RGD1307325	1	1	0	2
rno-miR-375-3p	RGD1311463	1	1	0	2
rno-miR-375-3p	Sdha	1	1	0	2
rno-miR-375-3p	Slco1c1	1	1	0	2
rno-miR-375-3p	tGap1	1	1	0	2
rno-miR-375-3p	Tmem19	1	1	0	2
rno-miR-375-3p	Tmem97	1	1	0	2
rno-miR-375-3p	Uxs1	1	1	0	2
rno-miR-375-3p	Ybx1	1	1	0	2
rno-miR-873-5p	Adam15	1	1	0	2
rno-miR-873-5p	Adcy3	1	0	1	2
rno-miR-873-5p	Adrm1	1	1	0	2
rno-miR-873-5p	Aph1a	1	1	0	2
rno-miR-873-5p	Atp6v0c	1	1	0	2
rno-miR-873-5p	Bat3	1	1	0	2
rno-miR-873-5p	Begain	1	0	1	2

rno-miR-873-5p	Cacnb2	1	0	1	2
rno-miR-873-5p	Capn8	1	1	0	2
rno-miR-873-5p	Capza2	1	0	1	2
rno-miR-873-5p	Ccl7	1	0	1	2
rno-miR-873-5p	Cd151	1	1	0	2
rno-miR-873-5p	Cdca7	1	0	1	2
rno-miR-873-5p	Cdh1	1	0	1	2
rno-miR-873-5p	Cebpb	1	1	0	2
rno-miR-873-5p	Chpt1	1	1	0	2
rno-miR-873-5p	Cml1	1	1	0	2
rno-miR-873-5p	Copb1	1	1	0	2
rno-miR-873-5p	Cxxc5	1	1	0	2
rno-miR-873-5p	Cyb5d2	1	1	0	2
rno-miR-873-5p	Dapk2	1	1	0	2
rno-miR-873-5p	Dbil5	1	1	0	2
rno-miR-873-5p	Dhrs4	1	1	0	2
rno-miR-873-5p	Doxl2	1	1	0	2
rno-miR-873-5p	Dsn1	1	0	1	2
rno-miR-873-5p	Eltd1	1	1	0	2
rno-miR-873-5p	Erap1	1	0	1	2
rno-miR-873-5p	Fkbp14	1	1	0	2
rno-miR-873-5p	Folr1	1	1	0	2
rno-miR-873-5p	G6pc3	1	1	0	2
rno-miR-873-5p	Gdf10	1	1	0	2
rno-miR-873-5p	Ghrhr	1	1	0	2
rno-miR-873-5p	Glyat	1	1	0	2
rno-miR-873-5p	Gng10	1	1	0	2
rno-miR-873-5p	Gnpat	1	1	0	2
rno-miR-873-5p	Gtf2e2	0	1	1	2
rno-miR-873-5p	Gyltl1b	1	1	0	2
rno-miR-873-5p	Hk2	1	1	0	2
rno-miR-873-5p	Hmgn2	1	1	0	2
rno-miR-873-5p	Hnrpm	1	1	0	2
rno-miR-873-5p	Hs3st2	1	1	0	2
rno-miR-873-5p	Igfbp2	1	1	0	2
rno-miR-873-5p	Il1rl2	1	1	0	2
rno-miR-873-5p	Kcnk6	1	1	0	2
rno-miR-873-5p	Lcmt1	1	1	0	2
rno-miR-873-5p	Lgmn	1	1	0	2
rno-miR-873-5p	Lim2	1	1	0	2
rno-miR-873-5p	LOC360479	1	1	0	2
rno-miR-873-5p	LOC500331	1	1	0	2
rno-miR-873-5p	LOC500354	1	1	0	2
rno-miR-873-5p	Lta	1	1	0	2
rno-miR-873-5p	Lzic	1	0	1	2
rno-miR-873-5p	Masp1	1	0	1	2
rno-miR-873-5p	Matr3	1	0	1	2
rno-miR-873-5p	Max	1	1	0	2
rno-miR-873-5p	Mefv	1	1	0	2
rno-miR-873-5p	Mmp10	1	1	0	2
rno-miR-873-5p	Mras	1	0	1	2

rno-miR-873-5p	Mrfap1	1	0	1	2
rno-miR-873-5p	Mterfd2	1	1	0	2
rno-miR-873-5p	Mtr	1	1	0	2
rno-miR-873-5p	Mxd3	1	1	0	2
rno-miR-873-5p	Nap1l3	1	1	0	2
rno-miR-873-5p	Nedd8	1	1	0	2
rno-miR-873-5p	Neurod1	1	1	0	2
rno-miR-873-5p	Ngfrap1	1	1	0	2
rno-miR-873-5p	Nme2	1	1	0	2
rno-miR-873-5p	Nono	1	0	1	2
rno-miR-873-5p	Nsf	1	1	0	2
rno-miR-873-5p	Opn4	1	1	0	2
rno-miR-873-5p	Osgepl1	1	1	0	2
rno-miR-873-5p	Pacsin3	1	1	0	2
rno-miR-873-5p	Pak2	1	1	0	2
rno-miR-873-5p	Pcgf6	1	1	0	2
rno-miR-873-5p	Pcyox1	1	1	0	2
rno-miR-873-5p	Pfkl	1	1	0	2
rno-miR-873-5p	Pgm1	1	1	0	2
rno-miR-873-5p	Podxl	1	0	1	2
rno-miR-873-5p	Polr3c	1	1	0	2
rno-miR-873-5p	Ppp2r1a	1	1	0	2
rno-miR-873-5p	Prss16	1	1	0	2
rno-miR-873-5p	Ptprr	1	1	0	2
rno-miR-873-5p	Pvalb	1	1	0	2
rno-miR-873-5p	Pvrl2	1	1	0	2
rno-miR-873-5p	Rad50	1	1	0	2
rno-miR-873-5p	Rbp2	1	1	0	2
rno-miR-873-5p	Rcor2	1	1	0	2
rno-miR-873-5p	Rdh11	1	1	0	2
rno-miR-873-5p	RGD1304719	1	1	0	2
rno-miR-873-5p	RGD1305014	1	1	0	2
rno-miR-873-5p	RGD1308470	1	0	1	2
rno-miR-873-5p	RGD1311463	1	1	0	2
rno-miR-873-5p	Rnase1	1	1	0	2
rno-miR-873-5p	Rnf41	1	1	0	2
rno-miR-873-5p	Rom1	1	1	0	2
rno-miR-873-5p	Rpp25	1	1	0	2
rno-miR-873-5p	Rpsa	1	1	0	2
rno-miR-873-5p	Scd1	1	1	0	2
rno-miR-873-5p	Sdc4	1	1	0	2
rno-miR-873-5p	Serpinh1	1	1	0	2
rno-miR-873-5p	Shank2	1	0	1	2
rno-miR-873-5p	Shisa5	1	0	1	2
rno-miR-873-5p	Slc10a1	1	1	0	2
rno-miR-873-5p	Slc12a3	1	1	0	2
rno-miR-873-5p	Slc16a13	1	1	0	2
rno-miR-873-5p	Slc39a6	1	0	1	2
rno-miR-873-5p	Slc4a1	1	1	0	2
rno-miR-873-5p	Slc6a7	1	1	0	2
rno-miR-873-5p	Slc7a13	1	1	0	2

rno-miR-873-5p	Sod3	1	1	0	2
rno-miR-873-5p	ST7	1	1	0	2
rno-miR-873-5p	Surf6	1	0	1	2
rno-miR-873-5p	Sv2c	1	1	0	2
rno-miR-873-5p	Tacc2	0	1	1	2
rno-miR-873-5p	Tax1bp1	1	1	0	2
rno-miR-873-5p	Tbrg1	1	1	0	2
rno-miR-873-5p	Tmem168	1	0	1	2
rno-miR-873-5p	Tnp2	1	1	0	2
rno-miR-873-5p	Trim39	1	1	0	2
rno-miR-873-5p	Tusc3	1	1	0	2
rno-miR-873-5p	Vps52	1	1	0	2
rno-miR-873-5p	Vtcn1	1	0	1	2
rno-miR-873-5p	Wfdc2	1	1	0	2
rno-miR-873-5p	Ywhae	1	1	0	2
rno-let-7c-1-3p	Actc1	0	0	1	1
rno-let-7c-1-3p	Adam10	0	0	1	1
rno-let-7c-1-3p	Adam19	0	0	1	1
rno-let-7c-1-3p	Adamtsl3	0	0	1	1
rno-let-7c-1-3p	Adnp	0	0	1	1
rno-let-7c-1-3p	Ahctf1	0	0	1	1
rno-let-7c-1-3p	Alx1	0	0	1	1
rno-let-7c-1-3p	Ampd3	0	0	1	1
rno-let-7c-1-3p	Ankmy2	0	0	1	1
rno-let-7c-1-3p	Ankrd12	0	0	1	1
rno-let-7c-1-3p	Ap2b1	0	0	1	1
rno-let-7c-1-3p	Atad2	0	0	1	1
rno-let-7c-1-3p	Atp1b1	0	0	1	1
rno-let-7c-1-3p	Atp6v1g1	0	0	1	1
rno-let-7c-1-3p	B4galt4	0	0	1	1
rno-let-7c-1-3p	B4galt6	0	0	1	1
rno-let-7c-1-3p	Brix1	0	0	1	1
rno-let-7c-1-3p	Btbd7	0	0	1	1
rno-let-7c-1-3p	Btg1	0	0	1	1
rno-let-7c-1-3p	Calm2	0	0	1	1
rno-let-7c-1-3p	Casc4	0	0	1	1
rno-let-7c-1-3p	Ccdc165	0	0	1	1
rno-let-7c-1-3p	Ccne2	0	0	1	1
rno-let-7c-1-3p	Ccng2	0	0	1	1
rno-let-7c-1-3p	Cd5l	0	0	1	1
rno-let-7c-1-3p	Cdh11	0	0	1	1
rno-let-7c-1-3p	Cdk17	0	0	1	1
rno-let-7c-1-3p	Cdkn1c	0	0	1	1
rno-let-7c-1-3p	Chd1	0	0	1	1
rno-let-7c-1-3p	Cisd2	0	0	1	1
rno-let-7c-1-3p	Cited2	0	0	1	1
rno-let-7c-1-3p	Cldn23	0	0	1	1
rno-let-7c-1-3p	Clk2	0	0	1	1
rno-let-7c-1-3p	Cnot6l	0	0	1	1
rno-let-7c-1-3p	Col11a1	0	0	1	1
rno-let-7c-1-3p	Col13a1	0	0	1	1

rno-let-7c-1-3p	Colec12	0	0	1	1
rno-let-7c-1-3p	Crmp1	0	0	1	1
rno-let-7c-1-3p	Csmd1	0	0	1	1
rno-let-7c-1-3p	Ctgf	0	0	1	1
rno-let-7c-1-3p	Cul3	0	0	1	1
rno-let-7c-1-3p	Cxcr4	0	0	1	1
rno-let-7c-1-3p	Cyfip2	0	0	1	1
rno-let-7c-1-3p	Dbn1	0	0	1	1
rno-let-7c-1-3p	Dclk2	0	0	1	1
rno-let-7c-1-3p	Dcx	0	0	1	1
rno-let-7c-1-3p	Ddx21	0	0	1	1
rno-let-7c-1-3p	Dek	0	0	1	1
rno-let-7c-1-3p	Derl1	0	0	1	1
rno-let-7c-1-3p	Dmd	0	0	1	1
rno-let-7c-1-3p	Dmrta1	0	0	1	1
rno-let-7c-1-3p	Dnajb12	0	0	1	1
rno-let-7c-1-3p	Dpp4	0	0	1	1
rno-let-7c-1-3p	Dscaml1	0	0	1	1
rno-let-7c-1-3p	Ebf2	0	0	1	1
rno-let-7c-1-3p	Efcab1	0	0	1	1
rno-let-7c-1-3p	Ei24	0	0	1	1
rno-let-7c-1-3p	Eif4b	0	0	1	1
rno-let-7c-1-3p	Eif4enif1	0	0	1	1
rno-let-7c-1-3p	Elavl2	0	0	1	1
rno-let-7c-1-3p	Ep300	0	0	1	1
rno-let-7c-1-3p	Epb41l3	0	0	1	1
rno-let-7c-1-3p	Evi2a	0	0	1	1
rno-let-7c-1-3p	Fam102b	0	0	1	1
rno-let-7c-1-3p	Fam20b	0	0	1	1
rno-let-7c-1-3p	Fam49b	0	0	1	1
rno-let-7c-1-3p	Fam98a	0	0	1	1
rno-let-7c-1-3p	Fbxl3	0	0	1	1
rno-let-7c-1-3p	Fbxo11	0	0	1	1
rno-let-7c-1-3p	Fbxo21	0	0	1	1
rno-let-7c-1-3p	Fbxw11	0	0	1	1
rno-let-7c-1-3p	Fermt2	0	0	1	1
rno-let-7c-1-3p	Foxc1	0	0	1	1
rno-let-7c-1-3p	Frmd6	0	0	1	1
rno-let-7c-1-3p	G3bp1	0	0	1	1
rno-let-7c-1-3p	Galnt7	0	0	1	1
rno-let-7c-1-3p	Gclc	0	0	1	1
rno-let-7c-1-3p	Gja1	0	0	1	1
rno-let-7c-1-3p	Gli2	0	0	1	1
rno-let-7c-1-3p	Gnai3	0	0	1	1
rno-let-7c-1-3p	Golga7	0	0	1	1
rno-let-7c-1-3p	Gphn	0	0	1	1
rno-let-7c-1-3p	Gpm6a	0	0	1	1
rno-let-7c-1-3p	Grb10	0	0	1	1
rno-let-7c-1-3p	Grhl2	0	0	1	1
rno-let-7c-1-3p	Grid2	0	0	1	1
rno-let-7c-1-3p	Hdgfrp3	0	0	1	1

rno-let-7c-1-3p	Herc1	0	0	1	1
rno-let-7c-1-3p	Hivep2	0	0	1	1
rno-let-7c-1-3p	Hmgb3	0	0	1	1
rno-let-7c-1-3p	Hmgcr	0	0	1	1
rno-let-7c-1-3p	Hmgcs1	0	0	1	1
rno-let-7c-1-3p	Hmgxb4	0	0	1	1
rno-let-7c-1-3p	Hnrnpu	0	0	1	1
rno-let-7c-1-3p	Hrb2	0	0	1	1
rno-let-7c-1-3p	Hrk	0	0	1	1
rno-let-7c-1-3p	Hspd1	0	0	1	1
rno-let-7c-1-3p	Id4	0	0	1	1
rno-let-7c-1-3p	Igsf3	0	0	1	1
rno-let-7c-1-3p	Ino80	0	0	1	1
rno-let-7c-1-3p	Ints10	0	0	1	1
rno-let-7c-1-3p	Ipo7	0	0	1	1
rno-let-7c-1-3p	Irx3	0	0	1	1
rno-let-7c-1-3p	Iws1	0	0	1	1
rno-let-7c-1-3p	Jazf1	0	0	1	1
rno-let-7c-1-3p	Jmjd1c	0	0	1	1
rno-let-7c-1-3p	Kcnc2	0	0	1	1
rno-let-7c-1-3p	Kdelr1	0	0	1	1
rno-let-7c-1-3p	Klf4	0	0	1	1
rno-let-7c-1-3p	Klhl14	0	0	1	1
rno-let-7c-1-3p	Klhl4	0	0	1	1
rno-let-7c-1-3p	Klhl5	0	0	1	1
rno-let-7c-1-3p	Klhl8	0	0	1	1
rno-let-7c-1-3p	Krit1	0	0	1	1
rno-let-7c-1-3p	Leprel1	0	0	1	1
rno-let-7c-1-3p	LOC10036060	0	0	1	1
rno-let-7c-1-3p	LOC10036098	0	0	1	1
rno-let-7c-1-3p	LOC10036125	0	0	1	1
rno-let-7c-1-3p	LOC10036285	0	0	1	1
rno-let-7c-1-3p	LOC10036471	0	0	1	1
rno-let-7c-1-3p	LOC10036607	0	0	1	1
rno-let-7c-1-3p	LOC294446	0	0	1	1
rno-let-7c-1-3p	LOC499124	0	0	1	1
rno-let-7c-1-3p	LOC688041	0	0	1	1
rno-let-7c-1-3p	LOC688133	0	0	1	1
rno-let-7c-1-3p	Lpar4	0	0	1	1
rno-let-7c-1-3p	Lrp12	0	0	1	1
rno-let-7c-1-3p	Lrp5	0	0	1	1
rno-let-7c-1-3p	Lrp6	0	0	1	1
rno-let-7c-1-3p	Lrrc4	0	0	1	1
rno-let-7c-1-3p	Lrrtm3	0	0	1	1
rno-let-7c-1-3p	Lzts2	0	0	1	1
rno-let-7c-1-3p	M6pr	0	0	1	1
rno-let-7c-1-3p	Map4k3	0	0	1	1
rno-let-7c-1-3p	March6	0	0	1	1
rno-let-7c-1-3p	Marcks	0	0	1	1
rno-let-7c-1-3p	Mef2c	0	0	1	1
rno-let-7c-1-3p	Meis2	0	0	1	1

rno-let-7c-1-3p	Memo1	0	0	1	1
rno-let-7c-1-3p	Mier1	0	0	1	1
rno-let-7c-1-3p	Mll4	0	0	1	1
rno-let-7c-1-3p	Mll5	0	0	1	1
rno-let-7c-1-3p	Mnt	0	0	1	1
rno-let-7c-1-3p	Mospd2	0	0	1	1
rno-let-7c-1-3p	Mpp5	0	0	1	1
rno-let-7c-1-3p	Msl2	0	0	1	1
rno-let-7c-1-3p	Mybl1	0	0	1	1
rno-let-7c-1-3p	Nampt	0	0	1	1
rno-let-7c-1-3p	Nap1l4	0	0	1	1
rno-let-7c-1-3p	Ncoa6	0	0	1	1
rno-let-7c-1-3p	Nedd4l	0	0	1	1
rno-let-7c-1-3p	Neo1	0	0	1	1
rno-let-7c-1-3p	Neurod1	0	0	1	1
rno-let-7c-1-3p	Neurod6	0	0	1	1
rno-let-7c-1-3p	Nfkbia	0	0	1	1
rno-let-7c-1-3p	Nfkbiz	0	0	1	1
rno-let-7c-1-3p	Nlk	0	0	1	1
rno-let-7c-1-3p	Npl	0	0	1	1
rno-let-7c-1-3p	Nr3c1	0	0	1	1
rno-let-7c-1-3p	Nr4a3	0	0	1	1
rno-let-7c-1-3p	Nrip1	0	0	1	1
rno-let-7c-1-3p	Nudt4	0	0	1	1
rno-let-7c-1-3p	Nup153	0	0	1	1
rno-let-7c-1-3p	Otud1	0	0	1	1
rno-let-7c-1-3p	P2ry10	0	0	1	1
rno-let-7c-1-3p	Pan3	0	0	1	1
rno-let-7c-1-3p	Pard6b	0	0	1	1
rno-let-7c-1-3p	Pdia3	0	0	1	1
rno-let-7c-1-3p	Pdia5	0	0	1	1
rno-let-7c-1-3p	Pex13	0	0	1	1
rno-let-7c-1-3p	Phf2	0	0	1	1
rno-let-7c-1-3p	Phtf2	0	0	1	1
rno-let-7c-1-3p	Pias3	0	0	1	1
rno-let-7c-1-3p	Pim3	0	0	1	1
rno-let-7c-1-3p	Plk1	0	0	1	1
rno-let-7c-1-3p	Pls3	0	0	1	1
rno-let-7c-1-3p	Pot1a	0	0	1	1
rno-let-7c-1-3p	Ppapdc2	0	0	1	1
rno-let-7c-1-3p	Ppip5k1	0	0	1	1
rno-let-7c-1-3p	Ppp1ca	0	0	1	1
rno-let-7c-1-3p	Ppp1r10	0	0	1	1
rno-let-7c-1-3p	Ppp1r8	0	0	1	1
rno-let-7c-1-3p	Ppp3r1	0	0	1	1
rno-let-7c-1-3p	Prkx	0	0	1	1
rno-let-7c-1-3p	Ptk2	0	0	1	1
rno-let-7c-1-3p	Ptprc	0	0	1	1
rno-let-7c-1-3p	Pum1	0	0	1	1
rno-let-7c-1-3p	Pum2	0	0	1	1
rno-let-7c-1-3p	R3hdm1	0	0	1	1

rno-let-7c-1-3p	Rab10	0	0	1	1
rno-let-7c-1-3p	Rab35	0	0	1	1
rno-let-7c-1-3p	Rab40b	0	0	1	1
rno-let-7c-1-3p	Rapgef2	0	0	1	1
rno-let-7c-1-3p	Rbbp5	0	0	1	1
rno-let-7c-1-3p	Rbbp6	0	0	1	1
rno-let-7c-1-3p	Rbm16	0	0	1	1
rno-let-7c-1-3p	Rdx	0	0	1	1
rno-let-7c-1-3p	Rev1	0	0	1	1
rno-let-7c-1-3p	RGD1307597	0	0	1	1
rno-let-7c-1-3p	RGD1310553	0	0	1	1
rno-let-7c-1-3p	RGD1310862	0	0	1	1
rno-let-7c-1-3p	RGD1563508	0	0	1	1
rno-let-7c-1-3p	Rif1	0	0	1	1
rno-let-7c-1-3p	Rlf	0	0	1	1
rno-let-7c-1-3p	Rnf139	0	0	1	1
rno-let-7c-1-3p	Rnps1	0	0	1	1
rno-let-7c-1-3p	Rsl24d1	0	0	1	1
rno-let-7c-1-3p	Runx2	0	0	1	1
rno-let-7c-1-3p	S1pr3	0	0	1	1
rno-let-7c-1-3p	Scamp5	0	0	1	1
rno-let-7c-1-3p	Scyl1	0	0	1	1
rno-let-7c-1-3p	Sdc2	0	0	1	1
rno-let-7c-1-3p	Sec11a	0	0	1	1
rno-let-7c-1-3p	Sec63	0	0	1	1
rno-let-7c-1-3p	Sema3c	0	0	1	1
rno-let-7c-1-3p	Sept2	0	0	1	1
rno-let-7c-1-3p	Sept7	0	0	1	1
rno-let-7c-1-3p	Sertad4	0	0	1	1
rno-let-7c-1-3p	Sf3b3	0	0	1	1
rno-let-7c-1-3p	Six1	0	0	1	1
rno-let-7c-1-3p	Slain2	0	0	1	1
rno-let-7c-1-3p	Slc25a12	0	0	1	1
rno-let-7c-1-3p	Slc2a2	0	0	1	1
rno-let-7c-1-3p	Slc30a7	0	0	1	1
rno-let-7c-1-3p	Slc6a6	0	0	1	1
rno-let-7c-1-3p	Slc7a11	0	0	1	1
rno-let-7c-1-3p	Slc7a6	0	0	1	1
rno-let-7c-1-3p	Slfn3	0	0	1	1
rno-let-7c-1-3p	Slit2	0	0	1	1
rno-let-7c-1-3p	Smarcd2	0	0	1	1
rno-let-7c-1-3p	Smc6	0	0	1	1
rno-let-7c-1-3p	Smpx	0	0	1	1
rno-let-7c-1-3p	Snrpb2	0	0	1	1
rno-let-7c-1-3p	Snrpd1	0	0	1	1
rno-let-7c-1-3p	Snx25	0	0	1	1
rno-let-7c-1-3p	Socs4	0	0	1	1
rno-let-7c-1-3p	Sox2	0	0	1	1
rno-let-7c-1-3p	Sox8	0	0	1	1
rno-let-7c-1-3p	Sp1	0	0	1	1
rno-let-7c-1-3p	Sp5	0	0	1	1

rno-let-7c-1-3p	Spg20	0	0	1	1
rno-let-7c-1-3p	Spry2	0	0	1	1
rno-let-7c-1-3p	Srebf1	0	0	1	1
rno-let-7c-1-3p	ST7	0	0	1	1
rno-let-7c-1-3p	Stag1	0	0	1	1
rno-let-7c-1-3p	Stat5a	0	0	1	1
rno-let-7c-1-3p	Stk39	0	0	1	1
rno-let-7c-1-3p	Tacstd2	0	0	1	1
rno-let-7c-1-3p	Taf5	0	0	1	1
rno-let-7c-1-3p	Tbc1d15	0	0	1	1
rno-let-7c-1-3p	Tbx18	0	0	1	1
rno-let-7c-1-3p	Tcf21	0	0	1	1
rno-let-7c-1-3p	Tcf7l2	0	0	1	1
rno-let-7c-1-3p	Tcte1	0	0	1	1
rno-let-7c-1-3p	Tctn1	0	0	1	1
rno-let-7c-1-3p	Tdp2	0	0	1	1
rno-let-7c-1-3p	Tlk1	0	0	1	1
rno-let-7c-1-3p	Tmco3	0	0	1	1
rno-let-7c-1-3p	Tmem117	0	0	1	1
rno-let-7c-1-3p	Tmem2	0	0	1	1
rno-let-7c-1-3p	Tmem47	0	0	1	1
rno-let-7c-1-3p	Tnfrsf26	0	0	1	1
rno-let-7c-1-3p	Tp63	0	0	1	1
rno-let-7c-1-3p	Tra2b	0	0	1	1
rno-let-7c-1-3p	Trim2	0	0	1	1
rno-let-7c-1-3p	Trrap	0	0	1	1
rno-let-7c-1-3p	Tsc22d1	0	0	1	1
rno-let-7c-1-3p	Tsc22d2	0	0	1	1
rno-let-7c-1-3p	Tshz1	0	0	1	1
rno-let-7c-1-3p	U2surp	0	0	1	1
rno-let-7c-1-3p	Uba3	0	0	1	1
rno-let-7c-1-3p	Ubqln2	0	0	1	1
rno-let-7c-1-3p	Uhmk1	0	0	1	1
rno-let-7c-1-3p	Ulk1	0	0	1	1
rno-let-7c-1-3p	Usmg5	0	0	1	1
rno-let-7c-1-3p	Usp29	0	0	1	1
rno-let-7c-1-3p	Wdr37	0	0	1	1
rno-let-7c-1-3p	Wdr47	0	0	1	1
rno-let-7c-1-3p	Ywhab	0	0	1	1
rno-let-7c-1-3p	Zdhhc20	0	0	1	1
rno-let-7c-1-3p	Zfand5	0	0	1	1
rno-let-7c-1-3p	Zfp192	0	0	1	1
rno-let-7c-1-3p	Zfp384	0	0	1	1
rno-let-7c-1-3p	Zfp638	0	0	1	1
rno-let-7c-1-3p	Zfr	0	0	1	1
rno-let-7c-1-3p	Zic5	0	0	1	1
rno-miR-1188-3p	Abcf2	0	0	1	1
rno-miR-1188-3p	Capzb	0	0	1	1
rno-miR-1188-3p	Ctcf	0	0	1	1
rno-miR-1188-3p	Flad1	0	0	1	1
rno-miR-1188-3p	Fndc3a	0	0	1	1

rno-miR-1188-3p	Grm2	0	0	1	1
rno-miR-1188-3p	Hic2	0	0	1	1
rno-miR-1188-3p	Hmgcs1	0	0	1	1
rno-miR-1188-3p	Hoxb6	0	0	1	1
rno-miR-1188-3p	Hoxb7	0	0	1	1
rno-miR-1188-3p	Larp1	0	0	1	1
rno-miR-1188-3p	Lipt2	0	0	1	1
rno-miR-1188-3p	Nxf1	0	0	1	1
rno-miR-1188-3p	Pfkl	0	0	1	1
rno-miR-1188-3p	Ppp3r1	0	0	1	1
rno-miR-1188-3p	Ptch2	0	0	1	1
rno-miR-1188-3p	Satb1	0	0	1	1
rno-miR-1188-3p	Secisbp2	0	0	1	1
rno-miR-1188-3p	Tceb3	0	0	1	1
rno-miR-1188-3p	Tdg	0	0	1	1
rno-miR-1188-3p	Thrap3	0	0	1	1
rno-miR-1188-3p	Tmem30a	0	0	1	1
rno-miR-1188-3p	Traf2	0	0	1	1
rno-miR-1188-3p	Ubash3b	0	0	1	1
rno-miR-1188-3p	Ube2i	0	0	1	1
rno-miR-1188-3p	Vav3	0	0	1	1
rno-miR-1188-3p	Vegfb	0	0	1	1
rno-miR-1188-3p	Zfp418	0	0	1	1
rno-miR-127-5p	Adamts19	0	0	1	1
rno-miR-127-5p	Adamts9	0	0	1	1
rno-miR-127-5p	Adcy7	0	0	1	1
rno-miR-127-5p	Anxa5	0	0	1	1
rno-miR-127-5p	Aox3	0	0	1	1
rno-miR-127-5p	Arih1	0	0	1	1
rno-miR-127-5p	Arl5b	0	0	1	1
rno-miR-127-5p	B2m	0	0	1	1
rno-miR-127-5p	Casc4	0	0	1	1
rno-miR-127-5p	Cggbp1	0	0	1	1
rno-miR-127-5p	Cnksr2	0	0	1	1
rno-miR-127-5p	Dcun1d1	0	0	1	1
rno-miR-127-5p	Dcun1d4	0	0	1	1
rno-miR-127-5p	Ddx3x	0	0	1	1
rno-miR-127-5p	Dnajb13	0	0	1	1
rno-miR-127-5p	Dph3	0	0	1	1
rno-miR-127-5p	Dpt	0	0	1	1
rno-miR-127-5p	Drd1a	0	0	1	1
rno-miR-127-5p	Elovl6	0	0	1	1
rno-miR-127-5p	Eny2	0	0	1	1
rno-miR-127-5p	Erc1	0	0	1	1
rno-miR-127-5p	Fam126b	0	0	1	1
rno-miR-127-5p	Fundc1	0	0	1	1
rno-miR-127-5p	Galnt11	0	0	1	1
rno-miR-127-5p	Gatad1	0	0	1	1
rno-miR-127-5p	Glcci1	0	0	1	1
rno-miR-127-5p	Glce	0	0	1	1
rno-miR-127-5p	Gls2	0	0	1	1

rno-miR-127-5p	Grp	0	0	1	1
rno-miR-127-5p	Gtdc1	0	0	1	1
rno-miR-127-5p	Hmgb2	0	0	1	1
rno-miR-127-5p	Hnrnpr	0	0	1	1
rno-miR-127-5p	Il7	0	0	1	1
rno-miR-127-5p	Itpr1	0	0	1	1
rno-miR-127-5p	Itpr2	0	0	1	1
rno-miR-127-5p	LOC10036165	0	0	1	1
rno-miR-127-5p	LOC10036574	0	0	1	1
rno-miR-127-5p	Lsm6	0	0	1	1
rno-miR-127-5p	Map7d2	0	0	1	1
rno-miR-127-5p	Mmp13	0	0	1	1
rno-miR-127-5p	Mtpap	0	0	1	1
rno-miR-127-5p	Mybl1	0	0	1	1
rno-miR-127-5p	N4bp2l2	0	0	1	1
rno-miR-127-5p	Naa30	0	0	1	1
rno-miR-127-5p	Ncam2	0	0	1	1
rno-miR-127-5p	Nefm	0	0	1	1
rno-miR-127-5p	Neurod1	0	0	1	1
rno-miR-127-5p	Nrp1	0	0	1	1
rno-miR-127-5p	Oat	0	0	1	1
rno-miR-127-5p	Orc5	0	0	1	1
rno-miR-127-5p	Pah	0	0	1	1
rno-miR-127-5p	Phf5a	0	0	1	1
rno-miR-127-5p	Ppp1cc	0	0	1	1
rno-miR-127-5p	Psmd3	0	0	1	1
rno-miR-127-5p	Rapgef2	0	0	1	1
rno-miR-127-5p	RGD1308601	0	0	1	1
rno-miR-127-5p	RGD1564519	0	0	1	1
rno-miR-127-5p	RGD1566036	0	0	1	1
rno-miR-127-5p	Rit2	0	0	1	1
rno-miR-127-5p	Rmi1	0	0	1	1
rno-miR-127-5p	Rras2	0	0	1	1
rno-miR-127-5p	Siva1	0	0	1	1
rno-miR-127-5p	Slc25a14	0	0	1	1
rno-miR-127-5p	Slc25a17	0	0	1	1
rno-miR-127-5p	Smarcd1	0	0	1	1
rno-miR-127-5p	Stk17b	0	0	1	1
rno-miR-127-5p	Stx18	0	0	1	1
rno-miR-127-5p	Tmem69	0	0	1	1
rno-miR-127-5p	Ube4a	0	0	1	1
rno-miR-127-5p	Ugp2	0	0	1	1
rno-miR-127-5p	Vash2	0	0	1	1
rno-miR-127-5p	Wif1	0	0	1	1
rno-miR-127-5p	Wnt8a	0	0	1	1
rno-miR-127-5p	Ypel5	0	0	1	1
rno-miR-127-5p	Ywhaq	0	0	1	1
rno-miR-127-5p	Zbtb20	0	0	1	1
rno-miR-127-5p	Zfpm2	0	0	1	1
rno-miR-181a-2-3p	Adss	0	0	1	1
rno-miR-181a-2-3p	Aldh1l2	0	0	1	1

rno-miR-181a-2-3p	Amotl2	0	0	1	1
rno-miR-181a-2-3p	Arhgap25	0	0	1	1
rno-miR-181a-2-3p	Ccdc43	0	0	1	1
rno-miR-181a-2-3p	Cox15	0	0	1	1
rno-miR-181a-2-3p	Cpox	0	0	1	1
rno-miR-181a-2-3p	Ctnnd1	0	0	1	1
rno-miR-181a-2-3p	Dock1	0	0	1	1
rno-miR-181a-2-3p	Elovl2	0	0	1	1
rno-miR-181a-2-3p	Emp1	0	0	1	1
rno-miR-181a-2-3p	Epb41l1	0	0	1	1
rno-miR-181a-2-3p	Hnrnpk	0	0	1	1
rno-miR-181a-2-3p	Il1rn	0	0	1	1
rno-miR-181a-2-3p	Itfg1	0	0	1	1
rno-miR-181a-2-3p	Kat7	0	0	1	1
rno-miR-181a-2-3p	LOC10036271	0	0	1	1
rno-miR-181a-2-3p	LOC679811	0	0	1	1
rno-miR-181a-2-3p	LOC680222	0	0	1	1
rno-miR-181a-2-3p	LOC688972	0	0	1	1
rno-miR-181a-2-3p	Lonrf2	0	0	1	1
rno-miR-181a-2-3p	Lrrc52	0	0	1	1
rno-miR-181a-2-3p	Mal2	0	0	1	1
rno-miR-181a-2-3p	Map3k7	0	0	1	1
rno-miR-181a-2-3p	Mki67	0	0	1	1
rno-miR-181a-2-3p	Mob4	0	0	1	1
rno-miR-181a-2-3p	Nup160	0	0	1	1
rno-miR-181a-2-3p	Pdcd10	0	0	1	1
rno-miR-181a-2-3p	Pou2af1	0	0	1	1
rno-miR-181a-2-3p	Ppp1r3c	0	0	1	1
rno-miR-181a-2-3p	Ppp2r2d	0	0	1	1
rno-miR-181a-2-3p	Prap1	0	0	1	1
rno-miR-181a-2-3p	Psme4	0	0	1	1
rno-miR-181a-2-3p	Rab22a	0	0	1	1
rno-miR-181a-2-3p	Rapgef2	0	0	1	1
rno-miR-181a-2-3p	Rbak	0	0	1	1
rno-miR-181a-2-3p	RGD1305225	0	0	1	1
rno-miR-181a-2-3p	RGD1306502	0	0	1	1
rno-miR-181a-2-3p	RGD1309747	0	0	1	1
rno-miR-181a-2-3p	Rtp3	0	0	1	1
rno-miR-181a-2-3p	Rwdd2b	0	0	1	1
rno-miR-181a-2-3p	Slc10a2	0	0	1	1
rno-miR-181a-2-3p	Sp3	0	0	1	1
rno-miR-181a-2-3p	Srsf6	0	0	1	1
rno-miR-181a-2-3p	St3gal4	0	0	1	1
rno-miR-181a-2-3p	Tbrg4	0	0	1	1
rno-miR-181a-2-3p	Tm4sf4	0	0	1	1
rno-miR-181a-2-3p	Tmem50a	0	0	1	1
rno-miR-181a-2-3p	Tmem87a	0	0	1	1
rno-miR-181a-2-3p	Tnpo2	0	0	1	1
rno-miR-181a-2-3p	Tnrc6b	0	0	1	1
rno-miR-181a-2-3p	Tra2b	0	0	1	1
rno-miR-181a-2-3p	Tril	0	0	1	1

rno-miR-181a-2-3p	Tsnax	0	0	1	1
rno-miR-181a-2-3p	Vom2r18	0	0	1	1
rno-miR-181a-2-3p	Zfp532	0	0	1	1
rno-miR-181a-2-3p	Zfp608	0	0	1	1
rno-miR-328a-3p	2900046G09R	0	1	0	1
rno-miR-328a-3p	4921506J03Ri	0	1	0	1
rno-miR-328a-3p	8430427H17R	0	1	0	1
rno-miR-328a-3p	9030205A07R	0	1	0	1
rno-miR-328a-3p	A830039N20R	0	1	0	1
rno-miR-328a-3p	Aanat	1	0	0	1
rno-miR-328a-3p	Aarsl_predicte	0	1	0	1
rno-miR-328a-3p	Abcb1	0	1	0	1
rno-miR-328a-3p	Abcb8	0	1	0	1
rno-miR-328a-3p	Abcg1	1	0	0	1
rno-miR-328a-3p	Abcg5	0	1	0	1
rno-miR-328a-3p	Abpa	0	1	0	1
rno-miR-328a-3p	Abtb1	0	1	0	1
rno-miR-328a-3p	Acads	0	1	0	1
rno-miR-328a-3p	Accn4	0	1	0	1
rno-miR-328a-3p	Acrbp	0	1	0	1
rno-miR-328a-3p	Acss2_predict	0	1	0	1
rno-miR-328a-3p	Adam7	1	0	0	1
rno-miR-328a-3p	Adamts14_pre	0	1	0	1
rno-miR-328a-3p	Add2	0	1	0	1
rno-miR-328a-3p	Adh6	1	0	0	1
rno-miR-328a-3p	Admr	0	1	0	1
rno-miR-328a-3p	Adsl_predicted	0	1	0	1
rno-miR-328a-3p	Aes	0	1	0	1
rno-miR-328a-3p	Agc1	0	1	0	1
rno-miR-328a-3p	Agpat4	0	1	0	1
rno-miR-328a-3p	Agtr2	1	0	0	1
rno-miR-328a-3p	Agtrap	1	0	0	1
rno-miR-328a-3p	Agxt	0	1	0	1
rno-miR-328a-3p	Ahr	1	0	0	1
rno-miR-328a-3p	AI507597	0	1	0	1
rno-miR-328a-3p	Aipl1	0	1	0	1
rno-miR-328a-3p	Aire_predicted	0	1	0	1
rno-miR-328a-3p	Akna_predicte	0	1	0	1
rno-miR-328a-3p	Akp5	0	1	0	1
rno-miR-328a-3p	Aktip	1	0	0	1
rno-miR-328a-3p	Alpi	1	0	0	1
rno-miR-328a-3p	Amd1	1	0	0	1
rno-miR-328a-3p	Angptl4	1	0	0	1
rno-miR-328a-3p	Ankfn1	0	1	0	1
rno-miR-328a-3p	Ankrd11_predi	0	1	0	1
rno-miR-328a-3p	Ankrd13d_pre	0	1	0	1
rno-miR-328a-3p	Ankrd46	1	0	0	1
rno-miR-328a-3p	Anxa8	0	1	0	1
rno-miR-328a-3p	Ap1b1	0	1	0	1
rno-miR-328a-3p	Apbb1	0	1	0	1
rno-miR-328a-3p	Aph1a	0	1	0	1

rno-miR-328a-3p	Apoc1	0	1	0	1
rno-miR-328a-3p	Apol9a	1	0	0	1
rno-miR-328a-3p	Aqp3	1	0	0	1
rno-miR-328a-3p	Aqp5	0	1	0	1
rno-miR-328a-3p	Aqp8	1	0	0	1
rno-miR-328a-3p	Araf	1	0	0	1
rno-miR-328a-3p	Arf5	0	1	0	1
rno-miR-328a-3p	Arfgap1	1	0	0	1
rno-miR-328a-3p	Arhgef18_pred	0	1	0	1
rno-miR-328a-3p	Arhgef2	1	0	0	1
rno-miR-328a-3p	Arhgef3_predic	0	1	0	1
rno-miR-328a-3p	Arhgef7	0	1	0	1
rno-miR-328a-3p	Arhgef9	1	0	0	1
rno-miR-328a-3p	Arid5a	0	1	0	1
rno-miR-328a-3p	Arl1	0	1	0	1
rno-miR-328a-3p	Arl11	0	1	0	1
rno-miR-328a-3p	Arl6ip4	0	1	0	1
rno-miR-328a-3p	Arl6ip6	1	0	0	1
rno-miR-328a-3p	Art3	0	1	0	1
rno-miR-328a-3p	As3mt	1	0	0	1
rno-miR-328a-3p	Asgr2	1	0	0	1
rno-miR-328a-3p	Atf5	1	0	0	1
rno-miR-328a-3p	Atg9a	1	0	0	1
rno-miR-328a-3p	Atp11a_predic	0	1	0	1
rno-miR-328a-3p	Atp1b3	1	0	0	1
rno-miR-328a-3p	Atp5f1	0	1	0	1
rno-miR-328a-3p	Atpif1	1	0	0	1
rno-miR-328a-3p	Axin1	1	0	0	1
rno-miR-328a-3p	B3galt1	0	0	1	1
rno-miR-328a-3p	B3galt7_predic	0	1	0	1
rno-miR-328a-3p	Bace1	0	1	0	1
rno-miR-328a-3p	Bai2_predicted	0	1	0	1
rno-miR-328a-3p	Baiap3	0	1	0	1
rno-miR-328a-3p	Barhl1	0	1	0	1
rno-miR-328a-3p	Bat5	0	1	0	1
rno-miR-328a-3p	BC021523	0	1	0	1
rno-miR-328a-3p	BC060632	0	1	0	1
rno-miR-328a-3p	Bckdha	0	1	0	1
rno-miR-328a-3p	Blvra	0	1	0	1
rno-miR-328a-3p	Bnc1	0	1	0	1
rno-miR-328a-3p	Bnipl	0	1	0	1
rno-miR-328a-3p	Bola1_predicte	0	1	0	1
rno-miR-328a-3p	BORG5_RAT	0	1	0	1
rno-miR-328a-3p	Brms1	1	0	0	1
rno-miR-328a-3p	Bsg	0	1	0	1
rno-miR-328a-3p	Bxdc2	1	0	0	1
rno-miR-328a-3p	Bzw2	1	0	0	1
rno-miR-328a-3p	Cabp7	1	0	0	1
rno-miR-328a-3p	Cacna1d_v1	0	1	0	1
rno-miR-328a-3p	Cacna2d1	0	1	0	1
rno-miR-328a-3p	Cacng8	0	1	0	1

rno-miR-328a-3p	Camkk1	1	0	0	1
rno-miR-328a-3p	Camkv	0	1	0	1
rno-miR-328a-3p	Capn9	0	1	0	1
rno-miR-328a-3p	Cc2d1a	1	0	0	1
rno-miR-328a-3p	Ccl22	1	0	0	1
rno-miR-328a-3p	Ccm2	0	1	0	1
rno-miR-328a-3p	Ccrk	1	0	0	1
rno-miR-328a-3p	Cd164	1	0	0	1
rno-miR-328a-3p	Cd276	1	0	0	1
rno-miR-328a-3p	Cd300lf	1	0	0	1
rno-miR-328a-3p	Cd40	1	0	0	1
rno-miR-328a-3p	Cd47	1	0	0	1
rno-miR-328a-3p	Cd6	1	0	0	1
rno-miR-328a-3p	Cd8b	1	0	0	1
rno-miR-328a-3p	Cdc37	0	1	0	1
rno-miR-328a-3p	Cdc91l1	0	1	0	1
rno-miR-328a-3p	Cdca7	1	0	0	1
rno-miR-328a-3p	Cdca8	0	1	0	1
rno-miR-328a-3p	Cdh15	0	1	0	1
rno-miR-328a-3p	Cdkl3	0	1	0	1
rno-miR-328a-3p	Celsr2	0	1	0	1
rno-miR-328a-3p	Celsr3	1	0	0	1
rno-miR-328a-3p	Cenpb_predict	0	1	0	1
rno-miR-328a-3p	Centg1	0	1	0	1
rno-miR-328a-3p	Ces3	0	1	0	1
rno-miR-328a-3p	Chdh	1	0	0	1
rno-miR-328a-3p	Chrna4	0	1	0	1
rno-miR-328a-3p	Chrnb2	1	0	0	1
rno-miR-328a-3p	Chst7	1	0	0	1
rno-miR-328a-3p	Cib1	0	1	0	1
rno-miR-328a-3p	Cish	0	1	0	1
rno-miR-328a-3p	Clasp2	0	1	0	1
rno-miR-328a-3p	Clcn4-2	1	0	0	1
rno-miR-328a-3p	Clic1	0	1	0	1
rno-miR-328a-3p	Clns1a	1	0	0	1
rno-miR-328a-3p	Clptm1_predic	0	1	0	1
rno-miR-328a-3p	Cnnm4_predic	0	1	0	1
rno-miR-328a-3p	Cog4_predicte	0	1	0	1
rno-miR-328a-3p	Cog7	0	1	0	1
rno-miR-328a-3p	Col23a1	0	1	0	1
rno-miR-328a-3p	Commd9	1	0	0	1
rno-miR-328a-3p	Cox15	1	0	0	1
rno-miR-328a-3p	Cplx1	0	1	0	1
rno-miR-328a-3p	Cpne5_predict	0	1	0	1
rno-miR-328a-3p	Cpsf1	0	1	0	1
rno-miR-328a-3p	Cpsf4	1	0	0	1
rno-miR-328a-3p	Crabp2	0	1	0	1
rno-miR-328a-3p	Crebl2	1	0	0	1
rno-miR-328a-3p	Crhr2	0	1	0	1
rno-miR-328a-3p	Crtc2	0	1	0	1
rno-miR-328a-3p	Cryl1	1	0	0	1

rno-miR-328a-3p	Csen	0	1	0	1
rno-miR-328a-3p	Csnk1g1	0	1	0	1
rno-miR-328a-3p	Csnk1g2	0	1	0	1
rno-miR-328a-3p	Cugbp2	1	0	0	1
rno-miR-328a-3p	Cx3cl1	1	0	0	1
rno-miR-328a-3p	Cxxc5	1	0	0	1
rno-miR-328a-3p	Cyb5b	1	0	0	1
rno-miR-328a-3p	Cyb5r1	1	0	0	1
rno-miR-328a-3p	Cyc1_predicte	0	1	0	1
rno-miR-328a-3p	Cygb	1	0	0	1
rno-miR-328a-3p	Cyp11b1	1	0	0	1
rno-miR-328a-3p	Cyp11b2	1	0	0	1
rno-miR-328a-3p	Cyp2a1	0	1	0	1
rno-miR-328a-3p	Cyp4f1	1	0	0	1
rno-miR-328a-3p	Cyp4f2	0	1	0	1
rno-miR-328a-3p	Cyp51	1	0	0	1
rno-miR-328a-3p	D10Ertd641e	0	1	0	1
rno-miR-328a-3p	Dao1	0	1	0	1
rno-miR-328a-3p	Dbi	1	0	0	1
rno-miR-328a-3p	Dbil5	0	1	0	1
rno-miR-328a-3p	Dbp	0	1	0	1
rno-miR-328a-3p	Dclk1	1	0	0	1
rno-miR-328a-3p	Ddit4	0	1	0	1
rno-miR-328a-3p	Defb42	0	1	0	1
rno-miR-328a-3p	Degs2	0	1	0	1
rno-miR-328a-3p	Des<i><sup>n	0	1	0	1
rno-miR-328a-3p	Dffb	0	1	0	1
rno-miR-328a-3p	Dhrs9	0	1	0	1
rno-miR-328a-3p	Dkk3	1	0	0	1
rno-miR-328a-3p	Dlgh4	0	1	0	1
rno-miR-328a-3p	Dmbt1	0	1	0	1
rno-miR-328a-3p	Dmn	0	1	0	1
rno-miR-328a-3p	Dnajb13	0	1	0	1
rno-miR-328a-3p	Dnajc5	0	1	0	1
rno-miR-328a-3p	Dnali1	1	0	0	1
rno-miR-328a-3p	Dnd1	0	1	0	1
rno-miR-328a-3p	Dnm1	0	1	0	1
rno-miR-328a-3p	Dnttip1	1	0	0	1
rno-miR-328a-3p	Dock3_predict	0	1	0	1
rno-miR-328a-3p	Dok1	0	1	0	1
rno-miR-328a-3p	Dok2_predicte	0	1	0	1
rno-miR-328a-3p	Dot1l_predicte	0	1	0	1
rno-miR-328a-3p	Dpagt1	1	0	0	1
rno-miR-328a-3p	Dpt_predicted	0	1	0	1
rno-miR-328a-3p	Drd1a	1	0	0	1
rno-miR-328a-3p	Dusp1	1	0	0	1
rno-miR-328a-3p	Dusp6	1	0	0	1
rno-miR-328a-3p	Dusp9	1	0	0	1
rno-miR-328a-3p	Dym_predicted	0	1	0	1
rno-miR-328a-3p	Dync1i1	1	0	0	1
rno-miR-328a-3p	Dyrk1a	1	0	0	1

rno-miR-328a-3p	Dzip1l	0	1	0	1
rno-miR-328a-3p	E430025E21R	0	1	0	1
rno-miR-328a-3p	Ebf1	1	0	0	1
rno-miR-328a-3p	Eif3s9	0	1	0	1
rno-miR-328a-3p	Eif4ebp3	0	1	0	1
rno-miR-328a-3p	Elf2	1	0	0	1
rno-miR-328a-3p	Elk4_predicted	0	1	0	1
rno-miR-328a-3p	En2	0	0	1	1
rno-miR-328a-3p	ENSMUSG000	0	1	0	1
rno-miR-328a-3p	Entpd2	0	1	0	1
rno-miR-328a-3p	Erabp	0	1	0	1
rno-miR-328a-3p	Erap1	1	0	0	1
rno-miR-328a-3p	Ermp1	1	0	0	1
rno-miR-328a-3p	Es22	1	0	0	1
rno-miR-328a-3p	Esco1	0	0	1	1
rno-miR-328a-3p	Espnl	0	1	0	1
rno-miR-328a-3p	Esrra	0	1	0	1
rno-miR-328a-3p	Etv4_predicted	0	1	0	1
rno-miR-328a-3p	Evpl_predicted	0	1	0	1
rno-miR-328a-3p	Exoc4	0	1	0	1
rno-miR-328a-3p	Exosc2_predic	0	1	0	1
rno-miR-328a-3p	Fabp2	0	1	0	1
rno-miR-328a-3p	Fam120b	0	0	1	1
rno-miR-328a-3p	Fam133b	1	0	0	1
rno-miR-328a-3p	Fam82a2	1	0	0	1
rno-miR-328a-3p	Fancc	0	1	0	1
rno-miR-328a-3p	Fbxo44	0	1	0	1
rno-miR-328a-3p	Fbxw4_predict	0	1	0	1
rno-miR-328a-3p	Fermt2	1	0	0	1
rno-miR-328a-3p	Fgf16	1	0	0	1
rno-miR-328a-3p	Fgf22	0	1	0	1
rno-miR-328a-3p	Fgl2	1	0	0	1
rno-miR-328a-3p	Fkbp10	1	0	0	1
rno-miR-328a-3p	Fkhl18	0	1	0	1
rno-miR-328a-3p	Fln29	0	1	0	1
rno-miR-328a-3p	Foxh1_predicte	0	1	0	1
rno-miR-328a-3p	Foxo4	0	0	1	1
rno-miR-328a-3p	Fshb	0	1	0	1
rno-miR-328a-3p	Ftcd	0	1	0	1
rno-miR-328a-3p	Fut1	1	0	0	1
rno-miR-328a-3p	Fut2	1	0	0	1
rno-miR-328a-3p	Fv1	1	0	0	1
rno-miR-328a-3p	G4	0	1	0	1
rno-miR-328a-3p	Gabbr1	1	0	0	1
rno-miR-328a-3p	Gal3st3	1	0	0	1
rno-miR-328a-3p	Galm	1	0	0	1
rno-miR-328a-3p	Gart	0	1	0	1
rno-miR-328a-3p	Gas6	0	1	0	1
rno-miR-328a-3p	Gata2	1	0	0	1
rno-miR-328a-3p	Gck	1	0	0	1
rno-miR-328a-3p	Gdpd3_predict	0	1	0	1

rno-miR-328a-3p	Geft	1	0	0	1
rno-miR-328a-3p	Ggt6	0	1	0	1
rno-miR-328a-3p	Ghsr<i><sup>	0	1	0	1
rno-miR-328a-3p	Gipc1	1	0	0	1
rno-miR-328a-3p	Gm2a	1	0	0	1
rno-miR-328a-3p	Gm672	0	1	0	1
rno-miR-328a-3p	Gm867	0	1	0	1
rno-miR-328a-3p	Gm963_predic	0	1	0	1
rno-miR-328a-3p	Gm98	0	1	0	1
rno-miR-328a-3p	Gmeb2	1	0	0	1
rno-miR-328a-3p	Gna11	0	1	0	1
rno-miR-328a-3p	Gng3	0	1	0	1
rno-miR-328a-3p	Gng7	0	1	0	1
rno-miR-328a-3p	Gp9	0	1	0	1
rno-miR-328a-3p	Gpd1	1	0	0	1
rno-miR-328a-3p	Gpm6a	1	0	0	1
rno-miR-328a-3p	Gpr182	1	0	0	1
rno-miR-328a-3p	Gpr27	0	1	0	1
rno-miR-328a-3p	Gpr2_predicte	0	1	0	1
rno-miR-328a-3p	Gpr41_predict	0	1	0	1
rno-miR-328a-3p	Gpr58	0	1	0	1
rno-miR-328a-3p	Gpr89_predict	0	1	0	1
rno-miR-328a-3p	Gpr97_predict	0	1	0	1
rno-miR-328a-3p	Gprc5a	0	0	1	1
rno-miR-328a-3p	Gprc5c	0	1	0	1
rno-miR-328a-3p	Gpt	1	0	0	1
rno-miR-328a-3p	Gpt1	0	1	0	1
rno-miR-328a-3p	Gpt2_predicte	0	1	0	1
rno-miR-328a-3p	Grem1	1	0	0	1
rno-miR-328a-3p	Grik4	0	1	0	1
rno-miR-328a-3p	Grik5	0	1	0	1
rno-miR-328a-3p	Grin2c	0	1	0	1
rno-miR-328a-3p	Gsbs	1	0	0	1
rno-miR-328a-3p	Gsta5	1	0	0	1
rno-miR-328a-3p	Gtdc1	1	0	0	1
rno-miR-328a-3p	Gtpbp5	1	0	0	1
rno-miR-328a-3p	Gypc	1	0	0	1
rno-miR-328a-3p	H2afx	0	1	0	1
rno-miR-328a-3p	Hand1	1	0	0	1
rno-miR-328a-3p	Has2	1	0	0	1
rno-miR-328a-3p	Hdac1	1	0	0	1
rno-miR-328a-3p	Hdgfrp3	0	1	0	1
rno-miR-328a-3p	Hes6	1	0	0	1
rno-miR-328a-3p	Hmx1_predicte	0	1	0	1
rno-miR-328a-3p	Hnrpul1_predic	0	1	0	1
rno-miR-328a-3p	Hsd17b7	1	0	0	1
rno-miR-328a-3p	Hsd3b7	0	1	0	1
rno-miR-328a-3p	Htatip	0	1	0	1
rno-miR-328a-3p	Ibrdc2_predict	0	1	0	1
rno-miR-328a-3p	Ica1	0	1	0	1
rno-miR-328a-3p	Ifit2	1	0	0	1

rno-miR-328a-3p	Igf2bp1	0	1	0	1
rno-miR-328a-3p	Igsf4c_predicte	0	1	0	1
rno-miR-328a-3p	Il17d	0	1	0	1
rno-miR-328a-3p	Il22ra2	1	0	0	1
rno-miR-328a-3p	Il9r	1	0	0	1
rno-miR-328a-3p	Ilk	0	1	0	1
rno-miR-328a-3p	Impa1	1	0	0	1
rno-miR-328a-3p	Inhbb	0	1	0	1
rno-miR-328a-3p	Inpp4a	1	0	0	1
rno-miR-328a-3p	Inpp5j	1	0	0	1
rno-miR-328a-3p	Inppl1	1	0	0	1
rno-miR-328a-3p	Ipmk	1	0	0	1
rno-miR-328a-3p	Iqce_predicted	0	1	0	1
rno-miR-328a-3p	Irf1	1	0	0	1
rno-miR-328a-3p	Irxl1_predicted	0	1	0	1
rno-miR-328a-3p	Itga3_predicte	0	1	0	1
rno-miR-328a-3p	Itga7	0	1	0	1
rno-miR-328a-3p	Itgal	0	1	0	1
rno-miR-328a-3p	Itih4	0	1	0	1
rno-miR-328a-3p	Itpa	0	1	0	1
rno-miR-328a-3p	Itsn1	0	1	0	1
rno-miR-328a-3p	Jmjd3	0	1	0	1
rno-miR-328a-3p	Jph2	0	1	0	1
rno-miR-328a-3p	Kars	0	1	0	1
rno-miR-328a-3p	Kb40	0	1	0	1
rno-miR-328a-3p	Kcna5	0	1	0	1
rno-miR-328a-3p	Kcna7_predict	0	1	0	1
rno-miR-328a-3p	Kcnd3	0	1	0	1
rno-miR-328a-3p	Kcnh6	0	1	0	1
rno-miR-328a-3p	Kcnh8	0	1	0	1
rno-miR-328a-3p	Kcnmb2	1	0	0	1
rno-miR-328a-3p	Kcnmb3_predi	0	1	0	1
rno-miR-328a-3p	Kcnmb4	0	1	0	1
rno-miR-328a-3p	Kctd1	0	1	0	1
rno-miR-328a-3p	Kif1c	0	1	0	1
rno-miR-328a-3p	Kif26a_predict	0	1	0	1
rno-miR-328a-3p	Kl	0	1	0	1
rno-miR-328a-3p	KLC1_RAT	0	1	0	1
rno-miR-328a-3p	Klc4	0	1	0	1
rno-miR-328a-3p	Klf15	1	0	0	1
rno-miR-328a-3p	Klhl20_predict	0	1	0	1
rno-miR-328a-3p	Klhl31	0	0	1	1
rno-miR-328a-3p	Klk12_predicte	0	1	0	1
rno-miR-328a-3p	Kremen1	0	1	0	1
rno-miR-328a-3p	Ky_predicted	0	1	0	1
rno-miR-328a-3p	Laptm5	1	0	0	1
rno-miR-328a-3p	Lass6	0	1	0	1
rno-miR-328a-3p	Lcn7	0	1	0	1
rno-miR-328a-3p	Lect2_predicte	0	1	0	1
rno-miR-328a-3p	Lim2	0	1	0	1
rno-miR-328a-3p	Limd1_predict	0	1	0	1

rno-miR-328a-3p	Limk1	0	1	0	1
rno-miR-328a-3p	Lipe<i><sup>n	0	1	0	1
rno-miR-328a-3p	Lmna	0	1	0	1
rno-miR-328a-3p	LOC10036221	0	0	1	1
rno-miR-328a-3p	LOC291967	0	1	0	1
rno-miR-328a-3p	LOC292801	0	1	0	1
rno-miR-328a-3p	LOC296165	0	1	0	1
rno-miR-328a-3p	LOC299282	0	1	0	1
rno-miR-328a-3p	LOC300768	0	1	0	1
rno-miR-328a-3p	LOC313672	0	1	0	1
rno-miR-328a-3p	LOC314655	0	1	0	1
rno-miR-328a-3p	LOC315712	0	1	0	1
rno-miR-328a-3p	LOC316326	0	1	0	1
rno-miR-328a-3p	LOC317416	0	1	0	1
rno-miR-328a-3p	LOC360570	0	1	0	1
rno-miR-328a-3p	LOC360910	0	1	0	1
rno-miR-328a-3p	LOC361399	0	1	0	1
rno-miR-328a-3p	LOC361420	0	1	0	1
rno-miR-328a-3p	LOC362012	0	1	0	1
rno-miR-328a-3p	LOC362901	0	1	0	1
rno-miR-328a-3p	LOC363351	0	1	0	1
rno-miR-328a-3p	LOC365238	0	1	0	1
rno-miR-328a-3p	LOC365778	1	0	0	1
rno-miR-328a-3p	LOC365839	0	1	0	1
rno-miR-328a-3p	LOC497873	0	1	0	1
rno-miR-328a-3p	LOC498459	0	1	0	1
rno-miR-328a-3p	LOC498685	0	1	0	1
rno-miR-328a-3p	LOC499306	1	0	0	1
rno-miR-328a-3p	LOC499418	1	0	0	1
rno-miR-328a-3p	LOC499749	1	0	0	1
rno-miR-328a-3p	LOC499754	0	1	0	1
rno-miR-328a-3p	LOC499768	0	1	0	1
rno-miR-328a-3p	LOC500442	0	1	0	1
rno-miR-328a-3p	LOC500573	0	1	0	1
rno-miR-328a-3p	LOC500591	0	1	0	1
rno-miR-328a-3p	LOC500901	0	1	0	1
rno-miR-328a-3p	LOC500908	0	1	0	1
rno-miR-328a-3p	LOC501110	1	0	0	1
rno-miR-328a-3p	LOC501718	0	1	0	1
rno-miR-328a-3p	LOC502758	0	1	0	1
rno-miR-328a-3p	LOC680317	0	1	0	1
rno-miR-328a-3p	LOC680434	0	1	0	1
rno-miR-328a-3p	LOC680448	0	1	0	1
rno-miR-328a-3p	LOC681012	0	1	0	1
rno-miR-328a-3p	LOC681287	0	1	0	1
rno-miR-328a-3p	LOC681457	0	1	0	1
rno-miR-328a-3p	LOC682142	0	1	0	1
rno-miR-328a-3p	LOC682185	0	1	0	1
rno-miR-328a-3p	LOC682929	0	1	0	1
rno-miR-328a-3p	LOC683786	0	1	0	1
rno-miR-328a-3p	LOC684528	0	1	0	1

rno-miR-328a-3p	LOC684629	0	1	0	1
rno-miR-328a-3p	LOC684802	0	1	0	1
rno-miR-328a-3p	LOC685385	0	1	0	1
rno-miR-328a-3p	LOC686428	0	0	1	1
rno-miR-328a-3p	LOC687072	0	1	0	1
rno-miR-328a-3p	LOC687139	0	1	0	1
rno-miR-328a-3p	LOC687237	0	1	0	1
rno-miR-328a-3p	LOC687948	0	1	0	1
rno-miR-328a-3p	LOC687964	0	1	0	1
rno-miR-328a-3p	LOC688223	0	1	0	1
rno-miR-328a-3p	LOC688339	0	1	0	1
rno-miR-328a-3p	LOC688510	0	1	0	1
rno-miR-328a-3p	LOC688708	0	1	0	1
rno-miR-328a-3p	LOC688777	0	1	0	1
rno-miR-328a-3p	LOC688990	0	1	0	1
rno-miR-328a-3p	LOC689039	0	1	0	1
rno-miR-328a-3p	LOC689142	0	1	0	1
rno-miR-328a-3p	LOC689199	0	1	0	1
rno-miR-328a-3p	LOC689755	0	1	0	1
rno-miR-328a-3p	LOC689765	0	1	0	1
rno-miR-328a-3p	LOC690911	0	1	0	1
rno-miR-328a-3p	LOC690955	0	1	0	1
rno-miR-328a-3p	LOC691143	0	1	0	1
rno-miR-328a-3p	Loxl4_predicte	0	1	0	1
rno-miR-328a-3p	Lphn2	1	0	0	1
rno-miR-328a-3p	Lrig1_predicte	0	1	0	1
rno-miR-328a-3p	Lrp1	0	1	0	1
rno-miR-328a-3p	Lrp3	1	0	0	1
rno-miR-328a-3p	Lrp4	1	0	0	1
rno-miR-328a-3p	Lrp5_predicted	0	1	0	1
rno-miR-328a-3p	Lrrc10_predict	0	1	0	1
rno-miR-328a-3p	Lrrc4	1	0	0	1
rno-miR-328a-3p	Lrrc42	0	1	0	1
rno-miR-328a-3p	Lrrc56	0	1	0	1
rno-miR-328a-3p	Lsm14a_predi	0	1	0	1
rno-miR-328a-3p	Lss	0	1	0	1
rno-miR-328a-3p	Lypla2	1	0	0	1
rno-miR-328a-3p	M6pr	1	0	0	1
rno-miR-328a-3p	Maf1	1	0	0	1
rno-miR-328a-3p	Mak16	1	0	0	1
rno-miR-328a-3p	Map2k7	0	1	0	1
rno-miR-328a-3p	Map3k1	0	1	0	1
rno-miR-328a-3p	Map3k7ip1	0	1	0	1
rno-miR-328a-3p	Map4k2_predi	0	1	0	1
rno-miR-328a-3p	Mapk13	1	0	0	1
rno-miR-328a-3p	Mapk8ip	0	1	0	1
rno-miR-328a-3p	Mapk8ip1	1	0	0	1
rno-miR-328a-3p	Mark2	0	1	0	1
rno-miR-328a-3p	Matk	0	1	0	1
rno-miR-328a-3p	Max	1	0	0	1
rno-miR-328a-3p	Me3_predicted	0	1	0	1

rno-miR-328a-3p	Med25_predict	0	1	0	1
rno-miR-328a-3p	Meis3_predicte	0	1	0	1
rno-miR-328a-3p	Mell1_predicte	0	1	0	1
rno-miR-328a-3p	Mesdc1	0	1	0	1
rno-miR-328a-3p	Mesdc2	1	0	0	1
rno-miR-328a-3p	Mettl3	0	1	0	1
rno-miR-328a-3p	Mettl7a	0	1	0	1
rno-miR-328a-3p	Mgat5	0	1	0	1
rno-miR-328a-3p	Mgat5b_predic	0	1	0	1
rno-miR-328a-3p	MGC109491	0	1	0	1
rno-miR-328a-3p	MGC112727	0	1	0	1
rno-miR-328a-3p	MGC72612	1	0	0	1
rno-miR-328a-3p	MGC94192	1	0	0	1
rno-miR-328a-3p	MGC94600	0	1	0	1
rno-miR-328a-3p	MGC94720	0	1	0	1
rno-miR-328a-3p	Mid1ip1	1	0	0	1
rno-miR-328a-3p	Mig12	0	1	0	1
rno-miR-328a-3p	Mip	0	1	0	1
rno-miR-328a-3p	Mlc3	0	1	0	1
rno-miR-328a-3p	Mlec	1	0	0	1
rno-miR-328a-3p	Mlph	1	0	0	1
rno-miR-328a-3p	Mmd2	0	0	1	1
rno-miR-328a-3p	Mmp23	0	1	0	1
rno-miR-328a-3p	Morn1	1	0	0	1
rno-miR-328a-3p	Mrfap1	1	0	0	1
rno-miR-328a-3p	Mrpl27_predict	0	1	0	1
rno-miR-328a-3p	Mrpl38	1	0	0	1
rno-miR-328a-3p	Mrpl46	0	1	0	1
rno-miR-328a-3p	Mrps6	0	1	0	1
rno-miR-328a-3p	Mtf1_predicted	0	1	0	1
rno-miR-328a-3p	Mtr	1	0	0	1
rno-miR-328a-3p	MUC4_RAT	0	1	0	1
rno-miR-328a-3p	Mx1	1	0	0	1
rno-miR-328a-3p	Myl9_predicted	0	1	0	1
rno-miR-328a-3p	Myo16	1	0	0	1
rno-miR-328a-3p	Myo1e	0	1	0	1
rno-miR-328a-3p	Myocd	1	0	0	1
rno-miR-328a-3p	Myom1	0	1	0	1
rno-miR-328a-3p	Myrip	0	1	0	1
rno-miR-328a-3p	N22.1	0	1	0	1
rno-miR-328a-3p	Naaladl1	0	1	0	1
rno-miR-328a-3p	Nanog	0	1	0	1
rno-miR-328a-3p	Nans_predicte	0	1	0	1
rno-miR-328a-3p	Narf	1	0	0	1
rno-miR-328a-3p	Ncam1	1	0	0	1
rno-miR-328a-3p	Ncaph2	1	0	0	1
rno-miR-328a-3p	Ncor2_predicte	0	1	0	1
rno-miR-328a-3p	Ndel1	1	0	0	1
rno-miR-328a-3p	Ndrg2	0	1	0	1
rno-miR-328a-3p	Ndufb7_predic	0	1	0	1
rno-miR-328a-3p	Ndufs2	0	1	0	1

rno-miR-328a-3p	Neb_predicted	0	1	0	1
rno-miR-328a-3p	Nebl_predicted	0	1	0	1
rno-miR-328a-3p	Nelf	1	0	0	1
rno-miR-328a-3p	Neud4	0	1	0	1
rno-miR-328a-3p	Neurod1	1	0	0	1
rno-miR-328a-3p	Neurod2	1	0	0	1
rno-miR-328a-3p	Nfasc	1	0	0	1
rno-miR-328a-3p	Ng3	0	1	0	1
rno-miR-328a-3p	Nkx2-3_predic	0	1	0	1
rno-miR-328a-3p	Nkx2-4_predic	0	1	0	1
rno-miR-328a-3p	Nkx2-5	1	0	0	1
rno-miR-328a-3p	Nkx3-1	1	0	0	1
rno-miR-328a-3p	Nlgn3	0	1	0	1
rno-miR-328a-3p	Nmur1	0	1	0	1
rno-miR-328a-3p	NP_00101394	0	1	0	1
rno-miR-328a-3p	NP_00107491	0	1	0	1
rno-miR-328a-3p	NP_110493.1	0	1	0	1
rno-miR-328a-3p	Npal3	0	1	0	1
rno-miR-328a-3p	Npas1_predict	0	1	0	1
rno-miR-328a-3p	Npdc1	1	0	0	1
rno-miR-328a-3p	Npffr1	0	1	0	1
rno-miR-328a-3p	Nr1h2	0	1	0	1
rno-miR-328a-3p	Nr5a1	0	1	0	1
rno-miR-328a-3p	Nr6a1	0	1	0	1
rno-miR-328a-3p	Nrap_predicted	0	1	0	1
rno-miR-328a-3p	Nt5c	0	1	0	1
rno-miR-328a-3p	Nt5dc1	0	0	1	1
rno-miR-328a-3p	Ntrk2	1	0	0	1
rno-miR-328a-3p	Nufip1	0	1	0	1
rno-miR-328a-3p	null	0	1	0	1
rno-miR-328a-3p	Nxph3	1	0	0	1
rno-miR-328a-3p	O55168_RAT	0	1	0	1
rno-miR-328a-3p	Oas1d	1	0	0	1
rno-miR-328a-3p	Oas1e	1	0	0	1
rno-miR-328a-3p	Odz4_predicte	0	1	0	1
rno-miR-328a-3p	Og9x	0	1	0	1
rno-miR-328a-3p	Okl38	0	1	0	1
rno-miR-328a-3p	Olfml1	1	0	0	1
rno-miR-328a-3p	Olfml2b_predic	0	1	0	1
rno-miR-328a-3p	Olr1366	1	0	0	1
rno-miR-328a-3p	Olr1433_predi	0	1	0	1
rno-miR-328a-3p	Olr1551_predi	0	1	0	1
rno-miR-328a-3p	Olr1572_predi	0	1	0	1
rno-miR-328a-3p	Olr288_predict	0	1	0	1
rno-miR-328a-3p	Olr709_predict	0	1	0	1
rno-miR-328a-3p	Olr747_predict	0	1	0	1
rno-miR-328a-3p	Olr956_predict	0	1	0	1
rno-miR-328a-3p	Oprd1	0	1	0	1
rno-miR-328a-3p	Oprm1	1	0	0	1
rno-miR-328a-3p	Optn	1	0	0	1
rno-miR-328a-3p	Osgin1	1	0	0	1

rno-miR-328a-3p	Otop3_predicte	0	1	0	1
rno-miR-328a-3p	Otud7a	0	1	0	1
rno-miR-328a-3p	P2rx3	1	0	0	1
rno-miR-328a-3p	Pak6_predicte	0	1	0	1
rno-miR-328a-3p	Parp16	1	0	0	1
rno-miR-328a-3p	Pcbp4_predict	0	1	0	1
rno-miR-328a-3p	Pcdhb18	0	1	0	1
rno-miR-328a-3p	Pcsk7	1	0	0	1
rno-miR-328a-3p	Pctk3	0	1	0	1
rno-miR-328a-3p	Pcyt1b	1	0	0	1
rno-miR-328a-3p	Pde1c	0	1	0	1
rno-miR-328a-3p	Pecr	0	1	0	1
rno-miR-328a-3p	Pex11a	1	0	0	1
rno-miR-328a-3p	Pfkfb2	1	0	0	1
rno-miR-328a-3p	Pfn1	1	0	0	1
rno-miR-328a-3p	Pgea1	0	1	0	1
rno-miR-328a-3p	Pgk1	1	0	0	1
rno-miR-328a-3p	Phf5a	1	0	0	1
rno-miR-328a-3p	Phf7	0	1	0	1
rno-miR-328a-3p	Phldb1	0	1	0	1
rno-miR-328a-3p	Phox2a	0	1	0	1
rno-miR-328a-3p	Phyhip	1	0	0	1
rno-miR-328a-3p	Pias3	1	0	0	1
rno-miR-328a-3p	Pitpnm1	0	1	0	1
rno-miR-328a-3p	Pkdrej_predict	0	1	0	1
rno-miR-328a-3p	Pkn3	0	1	0	1
rno-miR-328a-3p	Pkp3_predicte	0	1	0	1
rno-miR-328a-3p	Plcg1	1	0	0	1
rno-miR-328a-3p	Pllp	1	0	0	1
rno-miR-328a-3p	Plxnc1	0	1	0	1
rno-miR-328a-3p	Plxnd1_predict	0	1	0	1
rno-miR-328a-3p	Pnldc1	1	0	0	1
rno-miR-328a-3p	Pofut2_predict	0	1	0	1
rno-miR-328a-3p	Polr2f	1	0	0	1
rno-miR-328a-3p	Polr3b	0	1	0	1
rno-miR-328a-3p	Polrmt_predict	0	1	0	1
rno-miR-328a-3p	Pou1f1	1	0	0	1
rno-miR-328a-3p	Ppfia4	0	0	1	1
rno-miR-328a-3p	Ppm1f	1	0	0	1
rno-miR-328a-3p	Ppp2r5b	1	0	0	1
rno-miR-328a-3p	Ppp3r1	0	1	0	1
rno-miR-328a-3p	Ppp5c	0	1	0	1
rno-miR-328a-3p	Prdx5	1	0	0	1
rno-miR-328a-3p	Prg-2	0	1	0	1
rno-miR-328a-3p	Prkch	1	0	0	1
rno-miR-328a-3p	Prkd2	0	1	0	1
rno-miR-328a-3p	Prodh2	0	1	0	1
rno-miR-328a-3p	Prop1	0	1	0	1
rno-miR-328a-3p	Prrxl1	1	0	0	1
rno-miR-328a-3p	Prss23	1	0	0	1
rno-miR-328a-3p	Prss8	1	0	0	1

rno-miR-328a-3p	Psmb5	0	1	0	1
rno-miR-328a-3p	Psme3	1	0	0	1
rno-miR-328a-3p	Pstpip1_predic	0	1	0	1
rno-miR-328a-3p	Ptger2	0	1	0	1
rno-miR-328a-3p	Ptgs1	1	0	0	1
rno-miR-328a-3p	Ptk2b	1	0	0	1
rno-miR-328a-3p	Ptov1	0	1	0	1
rno-miR-328a-3p	Ptpn14_predic	0	1	0	1
rno-miR-328a-3p	Ptpn18	1	0	0	1
rno-miR-328a-3p	Ptpra	1	0	0	1
rno-miR-328a-3p	Ptpre	0	1	0	1
rno-miR-328a-3p	Pus10	1	0	0	1
rno-miR-328a-3p	PVR	1	0	0	1
rno-miR-328a-3p	Pxk	0	1	0	1
rno-miR-328a-3p	Pxn	0	1	0	1
rno-miR-328a-3p	Pycrl	0	1	0	1
rno-miR-328a-3p	Q4G039_RAT	0	1	0	1
rno-miR-328a-3p	Q5BJU4_RAT	0	1	0	1
rno-miR-328a-3p	Q5RK22_RAT	0	1	0	1
rno-miR-328a-3p	Q80YF3_RAT	0	1	0	1
rno-miR-328a-3p	Q8CFC4_RAT	0	1	0	1
rno-miR-328a-3p	Q8CIE4_MOU	0	1	0	1
rno-miR-328a-3p	Q8K3V5_RAT	0	1	0	1
rno-miR-328a-3p	Qprt	0	1	0	1
rno-miR-328a-3p	Rab28	0	1	0	1
rno-miR-328a-3p	Rab31	1	0	0	1
rno-miR-328a-3p	Rab40c	0	1	0	1
rno-miR-328a-3p	Rab4b	1	0	0	1
rno-miR-328a-3p	Rab8a	0	1	0	1
rno-miR-328a-3p	Rad1_predicte	0	1	0	1
rno-miR-328a-3p	Rad23b	1	0	0	1
rno-miR-328a-3p	Rapsn_predict	0	1	0	1
rno-miR-328a-3p	Rara	0	1	0	1
rno-miR-328a-3p	Rasgef1a	0	1	0	1
rno-miR-328a-3p	Rasgrp2_predi	0	1	0	1
rno-miR-328a-3p	Rasl10b_predi	0	1	0	1
rno-miR-328a-3p	Rassf1	0	1	0	1
rno-miR-328a-3p	Rassf2	1	0	0	1
rno-miR-328a-3p	Rbaf600	0	1	0	1
rno-miR-328a-3p	Rbfox1	0	0	1	1
rno-miR-328a-3p	Rbm7_predicte	0	1	0	1
rno-miR-328a-3p	Rdh5_predicte	0	1	0	1
rno-miR-328a-3p	Recc1	0	1	0	1
rno-miR-328a-3p	Rem2	0	1	0	1
rno-miR-328a-3p	Rer1	1	0	0	1
rno-miR-328a-3p	RGD1303066	0	1	0	1
rno-miR-328a-3p	RGD1303271	1	0	0	1
rno-miR-328a-3p	RGD1304595_	0	1	0	1
rno-miR-328a-3p	RGD1304688_	0	1	0	1
rno-miR-328a-3p	RGD1304694	0	1	0	1
rno-miR-328a-3p	RGD1304816_	0	1	0	1

rno-miR-328a-3p	RGD1304963_	0	1	0	1
rno-miR-328a-3p	RGD1305061	0	1	0	1
rno-miR-328a-3p	RGD1305157_	0	1	0	1
rno-miR-328a-3p	RGD1305254_	0	1	0	1
rno-miR-328a-3p	RGD1305298_	0	1	0	1
rno-miR-328a-3p	RGD1305350_	0	1	0	1
rno-miR-328a-3p	RGD1305614_	0	1	0	1
rno-miR-328a-3p	RGD1305833	0	1	0	1
rno-miR-328a-3p	RGD1305903_	0	1	0	1
rno-miR-328a-3p	RGD1306108	0	1	0	1
rno-miR-328a-3p	RGD1306233_	0	1	0	1
rno-miR-328a-3p	RGD1306502_	0	1	0	1
rno-miR-328a-3p	RGD1306582	0	1	0	1
rno-miR-328a-3p	RGD1306729_	0	1	0	1
rno-miR-328a-3p	RGD1306787_	0	1	0	1
rno-miR-328a-3p	RGD1307723_	0	1	0	1
rno-miR-328a-3p	RGD1307767_	0	1	0	1
rno-miR-328a-3p	RGD1307772	0	1	0	1
rno-miR-328a-3p	RGD1307822	0	1	0	1
rno-miR-328a-3p	RGD1308111_	0	1	0	1
rno-miR-328a-3p	RGD1308134_	0	1	0	1
rno-miR-328a-3p	RGD1308215_	0	1	0	1
rno-miR-328a-3p	RGD1308290_	0	1	0	1
rno-miR-328a-3p	RGD1308356	0	1	0	1
rno-miR-328a-3p	RGD1308396_	0	1	0	1
rno-miR-328a-3p	RGD1308787_	0	1	0	1
rno-miR-328a-3p	RGD1308836	0	1	0	1
rno-miR-328a-3p	RGD1308848_	0	1	0	1
rno-miR-328a-3p	RGD1308874	1	0	0	1
rno-miR-328a-3p	RGD1308876_	0	1	0	1
rno-miR-328a-3p	RGD1308915	0	1	0	1
rno-miR-328a-3p	RGD1308929_	0	1	0	1
rno-miR-328a-3p	RGD1309170_	0	1	0	1
rno-miR-328a-3p	RGD1309220	1	0	0	1
rno-miR-328a-3p	RGD1309414_	0	1	0	1
rno-miR-328a-3p	RGD1309594	1	0	0	1
rno-miR-328a-3p	RGD1309870_	0	1	0	1
rno-miR-328a-3p	RGD1310066	0	1	0	1
rno-miR-328a-3p	RGD1310090	0	1	0	1
rno-miR-328a-3p	RGD1310111	0	1	0	1
rno-miR-328a-3p	RGD1310127	1	0	0	1
rno-miR-328a-3p	RGD1310320	0	1	0	1
rno-miR-328a-3p	RGD1310348_	0	1	0	1
rno-miR-328a-3p	RGD1310357_	0	1	0	1
rno-miR-328a-3p	RGD1310490_	0	1	0	1
rno-miR-328a-3p	RGD1310536_	0	1	0	1
rno-miR-328a-3p	RGD1310708_	0	1	0	1
rno-miR-328a-3p	RGD1310875_	0	1	0	1
rno-miR-328a-3p	RGD1310887_	0	1	0	1
rno-miR-328a-3p	RGD1310964_	0	1	0	1
rno-miR-328a-3p	RGD1311091_	0	1	0	1

rno-miR-328a-3p	RGD1311249	1	0	0	1
rno-miR-328a-3p	RGD1311267	1	0	0	1
rno-miR-328a-3p	RGD1311298_	0	1	0	1
rno-miR-328a-3p	RGD1311307	1	0	0	1
rno-miR-328a-3p	RGD1311451_	0	1	0	1
rno-miR-328a-3p	RGD1311698_	0	1	0	1
rno-miR-328a-3p	RGD1311847	0	1	0	1
rno-miR-328a-3p	RGD1359242	0	1	0	1
rno-miR-328a-3p	RGD1559493_	0	1	0	1
rno-miR-328a-3p	RGD1559811_	0	1	0	1
rno-miR-328a-3p	RGD1559856_	0	1	0	1
rno-miR-328a-3p	RGD1559942_	0	1	0	1
rno-miR-328a-3p	RGD1560015_	0	1	0	1
rno-miR-328a-3p	RGD1560213_	0	1	0	1
rno-miR-328a-3p	RGD1560330_	0	1	0	1
rno-miR-328a-3p	RGD1560519_	0	1	0	1
rno-miR-328a-3p	RGD1560593_	0	1	0	1
rno-miR-328a-3p	RGD1560636_	0	1	0	1
rno-miR-328a-3p	RGD1560673_	0	1	0	1
rno-miR-328a-3p	RGD1560686_	0	1	0	1
rno-miR-328a-3p	RGD1560880_	0	1	0	1
rno-miR-328a-3p	RGD1560887_	0	1	0	1
rno-miR-328a-3p	RGD1561119_	0	1	0	1
rno-miR-328a-3p	RGD1561325_	0	1	0	1
rno-miR-328a-3p	RGD1561352_	0	1	0	1
rno-miR-328a-3p	RGD1561500_	0	1	0	1
rno-miR-328a-3p	RGD1561521_	0	1	0	1
rno-miR-328a-3p	RGD1561608_	0	1	0	1
rno-miR-328a-3p	RGD1561842_	0	1	0	1
rno-miR-328a-3p	RGD1561909_	0	1	0	1
rno-miR-328a-3p	RGD1562008_	0	1	0	1
rno-miR-328a-3p	RGD1562036_	0	1	0	1
rno-miR-328a-3p	RGD1562084_	0	1	0	1
rno-miR-328a-3p	RGD1562299_	0	1	0	1
rno-miR-328a-3p	RGD1562943_	0	1	0	1
rno-miR-328a-3p	RGD1562992_	0	1	0	1
rno-miR-328a-3p	RGD1563034_	0	1	0	1
rno-miR-328a-3p	RGD1563208_	0	1	0	1
rno-miR-328a-3p	RGD1563431_	0	1	0	1
rno-miR-328a-3p	RGD1563463_	0	1	0	1
rno-miR-328a-3p	RGD1563731_	0	1	0	1
rno-miR-328a-3p	RGD1563798_	0	1	0	1
rno-miR-328a-3p	RGD1564010_	0	1	0	1
rno-miR-328a-3p	RGD1564027_	0	1	0	1
rno-miR-328a-3p	RGD1564197_	0	1	0	1
rno-miR-328a-3p	RGD1564337_	0	1	0	1
rno-miR-328a-3p	RGD1564385_	0	1	0	1
rno-miR-328a-3p	RGD1564403_	0	1	0	1
rno-miR-328a-3p	RGD1564412_	0	1	0	1
rno-miR-328a-3p	RGD1564862_	0	1	0	1
rno-miR-328a-3p	RGD1564946_	0	1	0	1

rno-miR-328a-3p	RGD1564994_	0	1	0	1
rno-miR-328a-3p	RGD1564996_	0	1	0	1
rno-miR-328a-3p	RGD1565166_	0	1	0	1
rno-miR-328a-3p	RGD1565232_	0	1	0	1
rno-miR-328a-3p	RGD1565408_	0	1	0	1
rno-miR-328a-3p	RGD1565414_	0	1	0	1
rno-miR-328a-3p	RGD1565542_	0	1	0	1
rno-miR-328a-3p	RGD1565757_	0	1	0	1
rno-miR-328a-3p	RGD1565779_	0	1	0	1
rno-miR-328a-3p	RGD1565810_	0	1	0	1
rno-miR-328a-3p	RGD1565857_	0	1	0	1
rno-miR-328a-3p	RGD1565883_	0	1	0	1
rno-miR-328a-3p	RGD1565972_	0	1	0	1
rno-miR-328a-3p	RGD1565984_	0	1	0	1
rno-miR-328a-3p	RGD1566048_	0	1	0	1
rno-miR-328a-3p	RGD1566083_	0	1	0	1
rno-miR-328a-3p	RGD1566130_	0	1	0	1
rno-miR-328a-3p	RGD1566394_	0	1	0	1
rno-miR-328a-3p	RGD1566403_	0	1	0	1
rno-miR-328a-3p	RGD735112	1	0	0	1
rno-miR-328a-3p	RGD735194	0	1	0	1
rno-miR-328a-3p	Rhobtb2	1	0	0	1
rno-miR-328a-3p	Rhog	1	0	0	1
rno-miR-328a-3p	Rhoq	1	0	0	1
rno-miR-328a-3p	Rhpn1	0	1	0	1
rno-miR-328a-3p	Rin3_predicted	0	1	0	1
rno-miR-328a-3p	Rkhd3	0	1	0	1
rno-miR-328a-3p	RL39_RAT	0	1	0	1
rno-miR-328a-3p	Rnaseh2c	0	1	0	1
rno-miR-328a-3p	Rnd1	1	0	0	1
rno-miR-328a-3p	Rnf44	0	1	0	1
rno-miR-328a-3p	Ros1	1	0	0	1
rno-miR-328a-3p	Rpa2	1	0	0	1
rno-miR-328a-3p	Rpl28	1	0	0	1
rno-miR-328a-3p	Rpl31	1	0	0	1
rno-miR-328a-3p	Rpl3l_predicte	0	1	0	1
rno-miR-328a-3p	Rpo1-3	1	0	0	1
rno-miR-328a-3p	Rragd_predicte	0	1	0	1
rno-miR-328a-3p	Rsbn1l_predic	0	1	0	1
rno-miR-328a-3p	Rspo4	0	1	0	1
rno-miR-328a-3p	Rtn4	0	1	0	1
rno-miR-328a-3p	Rutbc2_predic	0	1	0	1
rno-miR-328a-3p	Rxrb	0	1	0	1
rno-miR-328a-3p	S100a13	0	1	0	1
rno-miR-328a-3p	S100b	1	0	0	1
rno-miR-328a-3p	Sbf1_predicted	0	1	0	1
rno-miR-328a-3p	Scamp4	1	0	0	1
rno-miR-328a-3p	Scamp5	0	1	0	1
rno-miR-328a-3p	Scnn1b	0	1	0	1
rno-miR-328a-3p	Scnn1g	1	0	0	1
rno-miR-328a-3p	Sdc3	1	0	0	1

rno-miR-328a-3p	Sdccag3	1	0	0	1
rno-miR-328a-3p	Sec14l2	1	0	0	1
rno-miR-328a-3p	Sec16b	1	0	0	1
rno-miR-328a-3p	Sectm1a	1	0	0	1
rno-miR-328a-3p	Sema4c_predi	0	1	0	1
rno-miR-328a-3p	Serpina4	0	1	0	1
rno-miR-328a-3p	Serpina9_pred	0	1	0	1
rno-miR-328a-3p	Setd7	0	0	1	1
rno-miR-328a-3p	Sftpa1	1	0	0	1
rno-miR-328a-3p	Sfxn2	0	1	0	1
rno-miR-328a-3p	Sh3bp2	0	1	0	1
rno-miR-328a-3p	Sh3bp5	1	0	0	1
rno-miR-328a-3p	Sh3glb2	0	1	0	1
rno-miR-328a-3p	Shisa3	0	0	1	1
rno-miR-328a-3p	Sidt2_predicte	0	1	0	1
rno-miR-328a-3p	Six3	0	1	0	1
rno-miR-328a-3p	Sla2	0	1	0	1
rno-miR-328a-3p	Slamf9_predic	0	1	0	1
rno-miR-328a-3p	Slc12a3	1	0	0	1
rno-miR-328a-3p	Slc24a4	0	0	1	1
rno-miR-328a-3p	Slc24a4_predi	0	1	0	1
rno-miR-328a-3p	Slc25a14	1	0	0	1
rno-miR-328a-3p	Slc25a25	1	0	0	1
rno-miR-328a-3p	Slc25a26_pred	0	1	0	1
rno-miR-328a-3p	Slc28a2	1	0	0	1
rno-miR-328a-3p	Slc29a1	0	1	0	1
rno-miR-328a-3p	Slc34a2	0	1	0	1
rno-miR-328a-3p	Slc35e4	1	0	0	1
rno-miR-328a-3p	Slc36a2	0	1	0	1
rno-miR-328a-3p	Slc37a4	1	0	0	1
rno-miR-328a-3p	Slc38a2	1	0	0	1
rno-miR-328a-3p	Slc39a5_predi	0	1	0	1
rno-miR-328a-3p	Slc39a7	1	0	0	1
rno-miR-328a-3p	Slc44a2	0	0	1	1
rno-miR-328a-3p	Slc45a3_predi	0	1	0	1
rno-miR-328a-3p	Slc47a1	1	0	0	1
rno-miR-328a-3p	Slc4a10	1	0	0	1
rno-miR-328a-3p	Slc4a11_predi	0	1	0	1
rno-miR-328a-3p	Slc5a11	0	1	0	1
rno-miR-328a-3p	Slc5a6	1	0	0	1
rno-miR-328a-3p	Slc7a13	1	0	0	1
rno-miR-328a-3p	Slc7a7	1	0	0	1
rno-miR-328a-3p	Slc7a8	1	0	0	1
rno-miR-328a-3p	Slc8a3	1	0	0	1
rno-miR-328a-3p	Slco1a4	1	0	0	1
rno-miR-328a-3p	Slco1c1	1	0	0	1
rno-miR-328a-3p	Slitrk3_predict	0	1	0	1
rno-miR-328a-3p	Smarcb1	0	1	0	1
rno-miR-328a-3p	Smarcd2	1	0	0	1
rno-miR-328a-3p	Smpd3	1	0	0	1
rno-miR-328a-3p	Smtn	0	1	0	1

rno-miR-328a-3p	Snai3_predicte	0	1	0	1
rno-miR-328a-3p	Snap47	1	0	0	1
rno-miR-328a-3p	Snrp70_predic	0	1	0	1
rno-miR-328a-3p	Snrpa1	0	1	0	1
rno-miR-328a-3p	Sord	1	0	0	1
rno-miR-328a-3p	Sox4	0	1	0	1
rno-miR-328a-3p	Sp1	0	1	0	1
rno-miR-328a-3p	Sp5_predicted	0	1	0	1
rno-miR-328a-3p	Spag7_predict	0	1	0	1
rno-miR-328a-3p	Spas1	0	1	0	1
rno-miR-328a-3p	Spdye4	1	0	0	1
rno-miR-328a-3p	Spert	1	0	0	1
rno-miR-328a-3p	Srcrb4d_predic	0	1	0	1
rno-miR-328a-3p	Srebf1	0	1	0	1
rno-miR-328a-3p	Srxn1	0	1	0	1
rno-miR-328a-3p	St3gal3	0	1	0	1
rno-miR-328a-3p	Stac3_predicte	0	1	0	1
rno-miR-328a-3p	Stat5a	1	0	0	1
rno-miR-328a-3p	Stbd1	1	0	0	1
rno-miR-328a-3p	Stk11_predicte	0	1	0	1
rno-miR-328a-3p	Stk19	1	0	0	1
rno-miR-328a-3p	Stk3	1	0	0	1
rno-miR-328a-3p	Stk32c_predic	0	1	0	1
rno-miR-328a-3p	Stmn4	0	1	0	1
rno-miR-328a-3p	Stoml2	0	1	0	1
rno-miR-328a-3p	Stox1_predicte	0	1	0	1
rno-miR-328a-3p	Strada	1	0	0	1
rno-miR-328a-3p	Stx5	1	0	0	1
rno-miR-328a-3p	Stx5a	0	1	0	1
rno-miR-328a-3p	Stxbp1	0	1	0	1
rno-miR-328a-3p	Sumo1	1	0	0	1
rno-miR-328a-3p	Supt6h	0	1	0	1
rno-miR-328a-3p	Svop	0	1	0	1
rno-miR-328a-3p	Sympk	0	1	0	1
rno-miR-328a-3p	Syt7	1	0	0	1
rno-miR-328a-3p	Syt7_v3	0	1	0	1
rno-miR-328a-3p	Tap2	0	1	0	1
rno-miR-328a-3p	Tbx1_predicted	0	1	0	1
rno-miR-328a-3p	Tceb2	0	1	0	1
rno-miR-328a-3p	Tcf1	0	1	0	1
rno-miR-328a-3p	Tcf7l2	0	0	1	1
rno-miR-328a-3p	Tcfap4_predic	0	1	0	1
rno-miR-328a-3p	Tcp11l2	1	0	0	1
rno-miR-328a-3p	Tctex1	0	1	0	1
rno-miR-328a-3p	Tekt1	0	1	0	1
rno-miR-328a-3p	Tert	1	0	0	1
rno-miR-328a-3p	Tesk2	1	0	0	1
rno-miR-328a-3p	Tex101	0	1	0	1
rno-miR-328a-3p	Tfpi	1	0	0	1
rno-miR-328a-3p	Tfpt	0	1	0	1
rno-miR-328a-3p	Thap3_predict	0	1	0	1

rno-miR-328a-3p	Thbs2	0	1	0	1
rno-miR-328a-3p	Thh_predicted	0	1	0	1
rno-miR-328a-3p	Thoc3_predict	0	1	0	1
rno-miR-328a-3p	Tk2_predicted	0	1	0	1
rno-miR-328a-3p	Tle3	1	0	0	1
rno-miR-328a-3p	Tm7sf3	1	0	0	1
rno-miR-328a-3p	Tmed1	0	1	0	1
rno-miR-328a-3p	Tmem101	0	1	0	1
rno-miR-328a-3p	Tmem176a	1	0	0	1
rno-miR-328a-3p	Tmem177	1	0	0	1
rno-miR-328a-3p	Tmem186	1	0	0	1
rno-miR-328a-3p	Tmem49	1	0	0	1
rno-miR-328a-3p	Tmem52	0	1	0	1
rno-miR-328a-3p	Tmem54	0	1	0	1
rno-miR-328a-3p	Tmem58_pred	0	1	0	1
rno-miR-328a-3p	Tmem97	1	0	0	1
rno-miR-328a-3p	Tmprss2	1	0	0	1
rno-miR-328a-3p	Tmprss6_pred	0	1	0	1
rno-miR-328a-3p	Tnip2	0	1	0	1
rno-miR-328a-3p	Tnk2	1	0	0	1
rno-miR-328a-3p	Tnrc4	0	1	0	1
rno-miR-328a-3p	Tph1	0	1	0	1
rno-miR-328a-3p	Tpm1	1	0	0	1
rno-miR-328a-3p	Tppp3	1	0	0	1
rno-miR-328a-3p	Tprn	0	0	1	1
rno-miR-328a-3p	Trak2	0	1	0	1
rno-miR-328a-3p	Trcg1	0	1	0	1
rno-miR-328a-3p	Trim54	0	1	0	1
rno-miR-328a-3p	Trit1	0	1	0	1
rno-miR-328a-3p	Trmu_predicte	0	1	0	1
rno-miR-328a-3p	Trpm8	1	0	0	1
rno-miR-328a-3p	Trpv3	1	0	0	1
rno-miR-328a-3p	Trpv5	1	0	0	1
rno-miR-328a-3p	Tsku	1	0	0	1
rno-miR-328a-3p	Tsn	0	1	0	1
rno-miR-328a-3p	Tspan2	1	0	0	1
rno-miR-328a-3p	Tspan4	0	1	0	1
rno-miR-328a-3p	Tssk2	0	1	0	1
rno-miR-328a-3p	Ttc5	1	0	0	1
rno-miR-328a-3p	Ttc8_predicted	0	1	0	1
rno-miR-328a-3p	Ttc9c	1	0	0	1
rno-miR-328a-3p	Tubb3	0	1	0	1
rno-miR-328a-3p	Tubb5	1	0	0	1
rno-miR-328a-3p	Tubb6	0	1	0	1
rno-miR-328a-3p	Ube2z	1	0	0	1
rno-miR-328a-3p	Unc5b	0	1	0	1
rno-miR-328a-3p	Uqcrc1	0	1	0	1
rno-miR-328a-3p	Usf2	0	1	0	1
rno-miR-328a-3p	Ushbp1	0	1	0	1
rno-miR-328a-3p	Usp19	1	0	0	1
rno-miR-328a-3p	Usp48	1	0	0	1

rno-miR-328a-3p	Usp5	0	1	0	1
rno-miR-328a-3p	Vamp2	0	1	0	1
rno-miR-328a-3p	Vat1	0	1	0	1
rno-miR-328a-3p	Vax2	0	1	0	1
rno-miR-328a-3p	Vcam1	1	0	0	1
rno-miR-328a-3p	Vcpip1	1	0	0	1
rno-miR-328a-3p	Vsig4	1	0	0	1
rno-miR-328a-3p	Vtcn1	1	0	0	1
rno-miR-328a-3p	Wasf1	1	0	0	1
rno-miR-328a-3p	Wtip_predicted	0	1	0	1
rno-miR-328a-3p	Xbp1	1	0	0	1
rno-miR-328a-3p	XR_009608.1	0	1	0	1
rno-miR-328a-3p	XR_032494.1	0	1	0	1
rno-miR-328a-3p	Ybx2_predicte	0	1	0	1
rno-miR-328a-3p	Yif1b	0	1	0	1
rno-miR-328a-3p	Yipf2	0	1	0	1
rno-miR-328a-3p	Zbtb22	1	0	0	1
rno-miR-328a-3p	Zbtb9	1	0	0	1
rno-miR-328a-3p	Zc3h7b	0	1	0	1
rno-miR-328a-3p	Zcwpw1_predi	0	1	0	1
rno-miR-328a-3p	Zdhhc7	1	0	0	1
rno-miR-328a-3p	Zfhx2	0	1	0	1
rno-miR-328a-3p	Zfp13_predicte	0	1	0	1
rno-miR-328a-3p	Zfp384	1	0	0	1
rno-miR-328a-3p	Zfp393_predic	0	1	0	1
rno-miR-328a-3p	Zfp423	1	0	0	1
rno-miR-328a-3p	Zfp444_predic	0	1	0	1
rno-miR-328a-3p	Zfp446	0	1	0	1
rno-miR-328a-3p	Zfp513	0	1	0	1
rno-miR-328a-3p	Zfp709	1	0	0	1
rno-miR-328a-3p	Zfp799	1	0	0	1
rno-miR-328a-3p	Zfyve28_predi	0	1	0	1
rno-miR-328a-3p	Zmynd10	0	1	0	1
rno-miR-328a-3p	Zmynd19	0	1	0	1
rno-miR-328a-3p	Znf14	0	1	0	1
rno-miR-331-3p	0710005M24R	0	1	0	1
rno-miR-331-3p	1200011O22R	0	1	0	1
rno-miR-331-3p	1700023F06R	0	1	0	1
rno-miR-331-3p	1700095B22R	0	1	0	1
rno-miR-331-3p	3110079O15R	0	1	0	1
rno-miR-331-3p	3830408D24R	0	1	0	1
rno-miR-331-3p	5730593N15R	0	1	0	1
rno-miR-331-3p	A530088E08R	0	1	0	1
rno-miR-331-3p	A930006D01R	0	1	0	1
rno-miR-331-3p	Abcd2	1	0	0	1
rno-miR-331-3p	Abo_predicted	0	1	0	1
rno-miR-331-3p	Abr_predicted	0	1	0	1
rno-miR-331-3p	Abtb2	0	1	0	1
rno-miR-331-3p	Accn4	0	1	0	1
rno-miR-331-3p	Acsf2	1	0	0	1
rno-miR-331-3p	Acsl1	1	0	0	1

rno-miR-331-3p	Acsl5	0	1	0	1
rno-miR-331-3p	Ada	0	1	0	1
rno-miR-331-3p	Adam17	1	0	0	1
rno-miR-331-3p	Adam33	0	1	0	1
rno-miR-331-3p	Adamts7	0	1	0	1
rno-miR-331-3p	Adck5	0	1	0	1
rno-miR-331-3p	Adcy5	0	1	0	1
rno-miR-331-3p	Adcy6	1	0	0	1
rno-miR-331-3p	Adcy9_predict	0	1	0	1
rno-miR-331-3p	Afap1	1	0	0	1
rno-miR-331-3p	Agpat2_predic	0	1	0	1
rno-miR-331-3p	Agpat7_predic	0	1	0	1
rno-miR-331-3p	Aim1	0	1	0	1
rno-miR-331-3p	Alox5	0	1	0	1
rno-miR-331-3p	Ankrd13b	0	0	1	1
rno-miR-331-3p	Ankrd34a	1	0	0	1
rno-miR-331-3p	Ap1g2	0	1	0	1
rno-miR-331-3p	Apaf1	0	1	0	1
rno-miR-331-3p	Apbb1	0	1	0	1
rno-miR-331-3p	Apobec2_pred	0	1	0	1
rno-miR-331-3p	Araf	1	0	0	1
rno-miR-331-3p	Arhgef18_pred	0	1	0	1
rno-miR-331-3p	Arl12_predicte	0	1	0	1
rno-miR-331-3p	Armc5	0	1	0	1
rno-miR-331-3p	Arrdc1	0	1	0	1
rno-miR-331-3p	Art5	0	1	0	1
rno-miR-331-3p	Atf5	1	0	0	1
rno-miR-331-3p	Atp5d	0	1	0	1
rno-miR-331-3p	Atpbd1c	0	1	0	1
rno-miR-331-3p	Axin1	1	0	0	1
rno-miR-331-3p	B3galt7_predic	0	1	0	1
rno-miR-331-3p	B3gat1	1	0	0	1
rno-miR-331-3p	B3gnt4_predic	0	1	0	1
rno-miR-331-3p	B4galt2	0	0	1	1
rno-miR-331-3p	B4galt3	0	1	0	1
rno-miR-331-3p	Bai2_predicted	0	1	0	1
rno-miR-331-3p	Baiap2	0	1	0	1
rno-miR-331-3p	Bat3	1	0	0	1
rno-miR-331-3p	Bbs7	1	0	0	1
rno-miR-331-3p	Bbs9	0	1	0	1
rno-miR-331-3p	BC024139	0	1	0	1
rno-miR-331-3p	BC039210	0	1	0	1
rno-miR-331-3p	Bckdha	0	1	0	1
rno-miR-331-3p	Bcl11a	0	1	0	1
rno-miR-331-3p	Bcl2l2	0	1	0	1
rno-miR-331-3p	Bcl7c_predicte	0	1	0	1
rno-miR-331-3p	Bcl9l_predicte	0	1	0	1
rno-miR-331-3p	Begain	0	1	0	1
rno-miR-331-3p	Bicd2	1	0	0	1
rno-miR-331-3p	Bin1	0	1	0	1
rno-miR-331-3p	Bin2	1	0	0	1

rno-miR-331-3p	Blcap	1	0	0	1
rno-miR-331-3p	Blvra	0	1	0	1
rno-miR-331-3p	Bmf	1	0	0	1
rno-miR-331-3p	Bnip1	1	0	0	1
rno-miR-331-3p	Boc_predicted	0	1	0	1
rno-miR-331-3p	Bok	1	0	0	1
rno-miR-331-3p	Boll_predicted	0	1	0	1
rno-miR-331-3p	Bpnt1	0	1	0	1
rno-miR-331-3p	Bre	1	0	0	1
rno-miR-331-3p	Brpf1	0	1	0	1
rno-miR-331-3p	Brunol6	0	1	0	1
rno-miR-331-3p	Bsdc1	0	1	0	1
rno-miR-331-3p	Btbd14a	0	1	0	1
rno-miR-331-3p	Btg1	1	0	0	1
rno-miR-331-3p	Bud31	1	0	0	1
rno-miR-331-3p	C1qtnf6	1	0	0	1
rno-miR-331-3p	Ca5a	0	1	0	1
rno-miR-331-3p	Cacna1a	0	1	0	1
rno-miR-331-3p	Cacnb4	0	0	1	1
rno-miR-331-3p	Cad	0	1	0	1
rno-miR-331-3p	Calml3	1	0	0	1
rno-miR-331-3p	Camk1d	0	1	0	1
rno-miR-331-3p	Camkk2	0	1	0	1
rno-miR-331-3p	Capn6	1	0	0	1
rno-miR-331-3p	Capn8	0	1	0	1
rno-miR-331-3p	Capon	0	1	0	1
rno-miR-331-3p	Carhsp1	1	0	0	1
rno-miR-331-3p	Caskin2_predi	0	1	0	1
rno-miR-331-3p	Ccdc19	0	1	0	1
rno-miR-331-3p	Ccdc22_predic	0	1	0	1
rno-miR-331-3p	Ccdc53_predic	0	1	0	1
rno-miR-331-3p	Ccl21b	0	1	0	1
rno-miR-331-3p	Ccl25	0	1	0	1
rno-miR-331-3p	Ccr5	1	0	0	1
rno-miR-331-3p	Ccr7	0	1	0	1
rno-miR-331-3p	Cd247	1	0	0	1
rno-miR-331-3p	Cd55	1	0	0	1
rno-miR-331-3p	Cd72	0	1	0	1
rno-miR-331-3p	CD9_RAT	0	1	0	1
rno-miR-331-3p	Cdc16	0	1	0	1
rno-miR-331-3p	Cdc34	1	0	0	1
rno-miR-331-3p	Cdc37	0	1	0	1
rno-miR-331-3p	Cdc40_predict	0	1	0	1
rno-miR-331-3p	Cdca2	0	1	0	1
rno-miR-331-3p	Cdh15	0	1	0	1
rno-miR-331-3p	Cdh24	0	1	0	1
rno-miR-331-3p	Cdk5	0	1	0	1
rno-miR-331-3p	Cdk8	0	1	0	1
rno-miR-331-3p	Cdkn2aipnl	1	0	0	1
rno-miR-331-3p	Cdkn2b	1	0	0	1
rno-miR-331-3p	Cep76	0	1	0	1

rno-miR-331-3p	Chac1_predict	0	1	0	1
rno-miR-331-3p	Chmp6_predic	0	1	0	1
rno-miR-331-3p	Chrng	0	1	0	1
rno-miR-331-3p	Chst8_predicte	0	1	0	1
rno-miR-331-3p	Cib2	1	0	0	1
rno-miR-331-3p	Cidea_predicte	0	1	0	1
rno-miR-331-3p	Cilp2_predicte	0	1	0	1
rno-miR-331-3p	Cldn19	0	1	0	1
rno-miR-331-3p	Clec4n	0	1	0	1
rno-miR-331-3p	Clic3	0	1	0	1
rno-miR-331-3p	Clptm1_predic	0	1	0	1
rno-miR-331-3p	Cnksr3	1	0	0	1
rno-miR-331-3p	Cnnm1_predic	0	1	0	1
rno-miR-331-3p	Cnnm4_predic	0	1	0	1
rno-miR-331-3p	Cnot3_predicte	0	1	0	1
rno-miR-331-3p	Cntnap2	0	1	0	1
rno-miR-331-3p	Cntnap4_predi	0	1	0	1
rno-miR-331-3p	Coasy	0	1	0	1
rno-miR-331-3p	Col11a2	1	0	0	1
rno-miR-331-3p	Col9a2_predic	0	1	0	1
rno-miR-331-3p	Coro1c_predic	0	1	0	1
rno-miR-331-3p	Cplx1	0	1	0	1
rno-miR-331-3p	Cpox	1	0	0	1
rno-miR-331-3p	Crb3	1	0	0	1
rno-miR-331-3p	Crcp	1	0	0	1
rno-miR-331-3p	Creb3l2	1	0	0	1
rno-miR-331-3p	Creb3l4	0	1	0	1
rno-miR-331-3p	Crisp1	1	0	0	1
rno-miR-331-3p	Crmp1	1	0	0	1
rno-miR-331-3p	Cs	0	1	0	1
rno-miR-331-3p	Cspg4	1	0	0	1
rno-miR-331-3p	Ctdspl_predict	0	1	0	1
rno-miR-331-3p	Ctrb	0	1	0	1
rno-miR-331-3p	Cxxc5	0	1	0	1
rno-miR-331-3p	Cygb	0	1	0	1
rno-miR-331-3p	Cyhr1	0	1	0	1
rno-miR-331-3p	D330017J20R	0	1	0	1
rno-miR-331-3p	Daam2	0	1	0	1
rno-miR-331-3p	Daf1	0	1	0	1
rno-miR-331-3p	Dapp1_predict	0	1	0	1
rno-miR-331-3p	Dazap1	1	0	0	1
rno-miR-331-3p	Dbn1	1	0	0	1
rno-miR-331-3p	Dclk1	1	0	0	1
rno-miR-331-3p	Dctn3_predicte	0	1	0	1
rno-miR-331-3p	Dctn4	1	0	0	1
rno-miR-331-3p	Dd25	0	1	0	1
rno-miR-331-3p	Ddc	1	0	0	1
rno-miR-331-3p	Ddr1	0	1	0	1
rno-miR-331-3p	Ddx23_predict	0	1	0	1
rno-miR-331-3p	Dffa	1	0	0	1
rno-miR-331-3p	DHB13_RAT	0	1	0	1

rno-miR-331-3p	Dhrs9	1	0	0	1
rno-miR-331-3p	Dlx3	0	0	1	1
rno-miR-331-3p	Dlx4	0	0	1	1
rno-miR-331-3p	Dnajb11	1	0	0	1
rno-miR-331-3p	Dnajb5	0	0	1	1
rno-miR-331-3p	Dnajb5_predic	0	1	0	1
rno-miR-331-3p	Dnajc15_predi	0	1	0	1
rno-miR-331-3p	Dok1	0	1	0	1
rno-miR-331-3p	Drd2	0	1	0	1
rno-miR-331-3p	Dscr2_predicte	0	1	0	1
rno-miR-331-3p	Dtnb	0	1	0	1
rno-miR-331-3p	Dullard	0	1	0	1
rno-miR-331-3p	DUOX1_RAT	0	1	0	1
rno-miR-331-3p	Dusp2	1	0	0	1
rno-miR-331-3p	Dynll2	1	0	0	1
rno-miR-331-3p	Dyt1	0	1	0	1
rno-miR-331-3p	E2f6	0	1	0	1
rno-miR-331-3p	E430025E21R	0	1	0	1
rno-miR-331-3p	Ece1	1	0	0	1
rno-miR-331-3p	Eefsec	0	0	1	1
rno-miR-331-3p	Efhd1	0	0	1	1
rno-miR-331-3p	Egfl9_predicte	0	1	0	1
rno-miR-331-3p	Eif4ebp3	0	1	0	1
rno-miR-331-3p	Elf2	1	0	0	1
rno-miR-331-3p	Emb	0	1	0	1
rno-miR-331-3p	ENSMUSG000	0	1	0	1
rno-miR-331-3p	ENSMUSG000	0	1	0	1
rno-miR-331-3p	ENSMUSG000	0	1	0	1
rno-miR-331-3p	Epb4.9_predic	0	1	0	1
rno-miR-331-3p	Erf	0	1	0	1
rno-miR-331-3p	Ern2_predicted	0	1	0	1
rno-miR-331-3p	Espl1_predicte	0	1	0	1
rno-miR-331-3p	Exoc2	1	0	0	1
rno-miR-331-3p	Exoc3	0	1	0	1
rno-miR-331-3p	F101B_RAT	0	1	0	1
rno-miR-331-3p	F12	0	1	0	1
rno-miR-331-3p	Fads3	0	1	0	1
rno-miR-331-3p	Fam100a	1	0	0	1
rno-miR-331-3p	Fbl	1	0	0	1
rno-miR-331-3p	Fbxl19_predict	0	1	0	1
rno-miR-331-3p	Fbxl20	0	1	0	1
rno-miR-331-3p	Fbxw2_predict	0	1	0	1
rno-miR-331-3p	Fbxw9	0	1	0	1
rno-miR-331-3p	Fdxr	0	1	0	1
rno-miR-331-3p	Fgd2_predicte	0	1	0	1
rno-miR-331-3p	Fgd3_predicte	0	1	0	1
rno-miR-331-3p	Fgf16	0	1	0	1
rno-miR-331-3p	Fgf21	0	1	0	1
rno-miR-331-3p	Fgfr4	0	1	0	1
rno-miR-331-3p	Filip1	1	0	0	1
rno-miR-331-3p	Fkbp11	0	1	0	1

rno-miR-331-3p	Fkbp4	0	1	0	1
rno-miR-331-3p	Fkbp8	0	1	0	1
rno-miR-331-3p	Flt4	1	0	0	1
rno-miR-331-3p	Fmo2	1	0	0	1
rno-miR-331-3p	Foxa3	0	1	0	1
rno-miR-331-3p	Foxr1_predicte	0	1	0	1
rno-miR-331-3p	Frem2_predict	0	1	0	1
rno-miR-331-3p	Fut4	1	0	0	1
rno-miR-331-3p	Fxr2h_predicte	0	1	0	1
rno-miR-331-3p	G10	0	1	0	1
rno-miR-331-3p	Gabbr1	1	0	0	1
rno-miR-331-3p	Gabrr2	0	1	0	1
rno-miR-331-3p	Gadd45gip1	0	0	1	1
rno-miR-331-3p	Gak	0	1	0	1
rno-miR-331-3p	Gale	0	1	0	1
rno-miR-331-3p	Galp	0	1	0	1
rno-miR-331-3p	Gar1	1	0	0	1
rno-miR-331-3p	Gclc	1	0	0	1
rno-miR-331-3p	Gcn5l2_predic	0	1	0	1
rno-miR-331-3p	Gfer	0	1	0	1
rno-miR-331-3p	Ggtl3	0	1	0	1
rno-miR-331-3p	Giyd2	1	0	0	1
rno-miR-331-3p	Gja4	1	0	0	1
rno-miR-331-3p	Glra1	1	0	0	1
rno-miR-331-3p	Gm1043	0	1	0	1
rno-miR-331-3p	Gpd1	1	0	0	1
rno-miR-331-3p	Gpr124	0	1	0	1
rno-miR-331-3p	Gpr179	0	1	0	1
rno-miR-331-3p	Gpr37	1	0	0	1
rno-miR-331-3p	Gpr68_predict	0	1	0	1
rno-miR-331-3p	Gpr89_predict	0	1	0	1
rno-miR-331-3p	Gramd1b_pred	0	1	0	1
rno-miR-331-3p	Grid2	1	0	0	1
rno-miR-331-3p	Grina	1	0	0	1
rno-miR-331-3p	Grk1	0	1	0	1
rno-miR-331-3p	Grk5	1	0	0	1
rno-miR-331-3p	Guca2a	0	1	0	1
rno-miR-331-3p	Gypc	1	0	0	1
rno-miR-331-3p	Hadhsc	0	1	0	1
rno-miR-331-3p	Has2	1	0	0	1
rno-miR-331-3p	Hbp1	1	0	0	1
rno-miR-331-3p	Hcn2	0	1	0	1
rno-miR-331-3p	Heg1	0	1	0	1
rno-miR-331-3p	Hemk1_predic	0	1	0	1
rno-miR-331-3p	Hepacam	0	1	0	1
rno-miR-331-3p	Hlx1_predicted	0	1	0	1
rno-miR-331-3p	Hmox1	1	0	0	1
rno-miR-331-3p	Homer3	0	1	0	1
rno-miR-331-3p	Hoxb13_predic	0	1	0	1
rno-miR-331-3p	Hoxc4	0	1	0	1
rno-miR-331-3p	Hr	1	0	0	1

rno-miR-331-3p	Hrmt1l2	0	1	0	1
rno-miR-331-3p	Hrsp12	1	0	0	1
rno-miR-331-3p	Hs3st2	0	1	0	1
rno-miR-331-3p	Hsd17b1	0	1	0	1
rno-miR-331-3p	Hsd17b7	1	0	0	1
rno-miR-331-3p	Hsd17b8	1	0	0	1
rno-miR-331-3p	Hspb7	0	1	0	1
rno-miR-331-3p	Hspb9_predict	0	1	0	1
rno-miR-331-3p	Htatip	0	1	0	1
rno-miR-331-3p	Ibrdc3_predict	0	1	0	1
rno-miR-331-3p	Icam5_predict	0	1	0	1
rno-miR-331-3p	Idh3a	1	0	0	1
rno-miR-331-3p	Idh3B	0	1	0	1
rno-miR-331-3p	Ier2	1	0	0	1
rno-miR-331-3p	Ifi27	1	0	0	1
rno-miR-331-3p	Ikbkg	0	1	0	1
rno-miR-331-3p	Il1rl1	1	0	0	1
rno-miR-331-3p	Impg1	0	1	0	1
rno-miR-331-3p	Ing5_predicted	0	1	0	1
rno-miR-331-3p	Inhbc	1	0	0	1
rno-miR-331-3p	Ino80c	1	0	0	1
rno-miR-331-3p	Ints1	0	1	0	1
rno-miR-331-3p	Ipo4_predicted	0	1	0	1
rno-miR-331-3p	Iqcb1_predicte	0	1	0	1
rno-miR-331-3p	Irgq_predicted	0	1	0	1
rno-miR-331-3p	isg12(a)	0	1	0	1
rno-miR-331-3p	Itga3_predicte	0	1	0	1
rno-miR-331-3p	Itgb1	1	0	0	1
rno-miR-331-3p	Jdp1	0	1	0	1
rno-miR-331-3p	Ka16	0	1	0	1
rno-miR-331-3p	Ka22	0	1	0	1
rno-miR-331-3p	Kcna4	1	0	0	1
rno-miR-331-3p	Kcnc1	0	1	0	1
rno-miR-331-3p	Kcnip2	1	0	0	1
rno-miR-331-3p	Kcnj9	0	1	0	1
rno-miR-331-3p	Kcnk6	1	0	0	1
rno-miR-331-3p	Kctd14_predic	0	1	0	1
rno-miR-331-3p	Kir3dl1	0	1	0	1
rno-miR-331-3p	Klhl18_predict	0	1	0	1
rno-miR-331-3p	Klk11_predicte	0	1	0	1
rno-miR-331-3p	Krt84	0	1	0	1
rno-miR-331-3p	L3mbtl3	0	1	0	1
rno-miR-331-3p	Laptm4b	1	0	0	1
rno-miR-331-3p	Lasp1	1	0	0	1
rno-miR-331-3p	Ldlrad2	0	1	0	1
rno-miR-331-3p	Leprot	1	0	0	1
rno-miR-331-3p	Lig1	0	1	0	1
rno-miR-331-3p	LOC10027184	0	0	1	1
rno-miR-331-3p	LOC10036024	0	0	1	1
rno-miR-331-3p	LOC10036569	0	0	1	1
rno-miR-331-3p	LOC286914	0	1	0	1

rno-miR-331-3p	LOC287992	0	1	0	1
rno-miR-331-3p	LOC288750	0	1	0	1
rno-miR-331-3p	LOC292069	0	1	0	1
rno-miR-331-3p	LOC296300	0	1	0	1
rno-miR-331-3p	LOC298346	0	1	0	1
rno-miR-331-3p	LOC301231	0	1	0	1
rno-miR-331-3p	LOC304396	1	0	0	1
rno-miR-331-3p	LOC304558	0	1	0	1
rno-miR-331-3p	LOC308266	0	1	0	1
rno-miR-331-3p	LOC308846	0	1	0	1
rno-miR-331-3p	LOC311984	0	1	0	1
rno-miR-331-3p	LOC312102	0	1	0	1
rno-miR-331-3p	LOC312831	0	1	0	1
rno-miR-331-3p	LOC314251	0	1	0	1
rno-miR-331-3p	LOC314655	0	1	0	1
rno-miR-331-3p	LOC360479	0	1	0	1
rno-miR-331-3p	LOC360912	0	1	0	1
rno-miR-331-3p	LOC361399	0	1	0	1
rno-miR-331-3p	LOC361571	0	1	0	1
rno-miR-331-3p	LOC361596	0	1	0	1
rno-miR-331-3p	LOC362477	0	1	0	1
rno-miR-331-3p	LOC363256	0	1	0	1
rno-miR-331-3p	LOC364534	0	1	0	1
rno-miR-331-3p	LOC367295	0	1	0	1
rno-miR-331-3p	LOC367994	0	1	0	1
rno-miR-331-3p	LOC497959	0	1	0	1
rno-miR-331-3p	LOC498396	0	1	0	1
rno-miR-331-3p	LOC498606	0	1	0	1
rno-miR-331-3p	LOC499418	0	1	0	1
rno-miR-331-3p	LOC499709	0	1	0	1
rno-miR-331-3p	LOC499768	0	1	0	1
rno-miR-331-3p	LOC499885	0	1	0	1
rno-miR-331-3p	LOC500110	0	1	0	1
rno-miR-331-3p	LOC500442	0	1	0	1
rno-miR-331-3p	LOC500591	0	1	0	1
rno-miR-331-3p	LOC500901	0	1	0	1
rno-miR-331-3p	LOC501738	0	1	0	1
rno-miR-331-3p	LOC619574	1	0	0	1
rno-miR-331-3p	LOC678868	0	1	0	1
rno-miR-331-3p	LOC679290	0	1	0	1
rno-miR-331-3p	LOC679552	0	1	0	1
rno-miR-331-3p	LOC680127	0	1	0	1
rno-miR-331-3p	LOC680404	0	1	0	1
rno-miR-331-3p	LOC680434	0	1	0	1
rno-miR-331-3p	LOC680899	0	1	0	1
rno-miR-331-3p	LOC681012	0	1	0	1
rno-miR-331-3p	LOC681300	0	1	0	1
rno-miR-331-3p	LOC681316	0	1	0	1
rno-miR-331-3p	LOC681501	0	1	0	1
rno-miR-331-3p	LOC682100	0	1	0	1
rno-miR-331-3p	LOC682709	0	1	0	1

rno-miR-331-3p	LOC684053	0	1	0	1
rno-miR-331-3p	LOC684112	0	0	1	1
rno-miR-331-3p	LOC685169	0	1	0	1
rno-miR-331-3p	LOC685269	0	1	0	1
rno-miR-331-3p	LOC685325	0	1	0	1
rno-miR-331-3p	LOC685587	0	1	0	1
rno-miR-331-3p	LOC686055	0	1	0	1
rno-miR-331-3p	LOC686106	0	1	0	1
rno-miR-331-3p	LOC686139	0	1	0	1
rno-miR-331-3p	LOC686210	0	1	0	1
rno-miR-331-3p	LOC686393	0	1	0	1
rno-miR-331-3p	LOC686457	0	1	0	1
rno-miR-331-3p	LOC686771	0	1	0	1
rno-miR-331-3p	LOC686799	0	1	0	1
rno-miR-331-3p	LOC686868	0	1	0	1
rno-miR-331-3p	LOC687099	0	1	0	1
rno-miR-331-3p	LOC687186	0	1	0	1
rno-miR-331-3p	LOC688142	0	1	0	1
rno-miR-331-3p	LOC688289	0	1	0	1
rno-miR-331-3p	LOC688757	0	1	0	1
rno-miR-331-3p	LOC688777	0	1	0	1
rno-miR-331-3p	LOC688966	0	1	0	1
rno-miR-331-3p	LOC689038	0	1	0	1
rno-miR-331-3p	LOC689844	0	1	0	1
rno-miR-331-3p	LOC690366	0	1	0	1
rno-miR-331-3p	LOC690407	0	1	0	1
rno-miR-331-3p	LOC690504	0	1	0	1
rno-miR-331-3p	LOC690810	0	1	0	1
rno-miR-331-3p	LOC691086	0	1	0	1
rno-miR-331-3p	LOC691478	0	1	0	1
rno-miR-331-3p	LOC691719	0	1	0	1
rno-miR-331-3p	Lphn1	0	1	0	1
rno-miR-331-3p	Lrig2_predicte	0	1	0	1
rno-miR-331-3p	Lrp2bp	1	0	0	1
rno-miR-331-3p	Lrp4	1	0	0	1
rno-miR-331-3p	Lrrc33	0	1	0	1
rno-miR-331-3p	Lrrc4b_predict	0	1	0	1
rno-miR-331-3p	Lsm7_predicte	0	1	0	1
rno-miR-331-3p	Ltb	0	1	0	1
rno-miR-331-3p	Ltf_predicted	0	1	0	1
rno-miR-331-3p	Ly6e	1	0	0	1
rno-miR-331-3p	Ly6h_predicte	0	1	0	1
rno-miR-331-3p	Madd<i><sup>	0	1	0	1
rno-miR-331-3p	Mafg	0	1	0	1
rno-miR-331-3p	Mal	0	1	0	1
rno-miR-331-3p	Mall	1	0	0	1
rno-miR-331-3p	Mamdc2	0	1	0	1
rno-miR-331-3p	Map1lc3b	1	0	0	1
rno-miR-331-3p	Map2k7	0	1	0	1
rno-miR-331-3p	Map3k11	0	1	0	1
rno-miR-331-3p	Mapk8ip3	0	1	0	1

rno-miR-331-3p	Mapre1	1	0	0	1
rno-miR-331-3p	Mblac1	1	0	0	1
rno-miR-331-3p	Mdga1_predict	0	1	0	1
rno-miR-331-3p	Mell1_predicte	0	1	0	1
rno-miR-331-3p	Mfge8	1	0	0	1
rno-miR-331-3p	MFGM_RAT	0	1	0	1
rno-miR-331-3p	Mgat5	0	1	0	1
rno-miR-331-3p	Mgat5b_predic	0	1	0	1
rno-miR-331-3p	MGC112682	0	1	0	1
rno-miR-331-3p	MGC116147	0	1	0	1
rno-miR-331-3p	MGC125213	0	1	0	1
rno-miR-331-3p	MGC94145	0	1	0	1
rno-miR-331-3p	Mia2	0	1	0	1
rno-miR-331-3p	Mllt10	1	0	0	1
rno-miR-331-3p	Mllt6	0	1	0	1
rno-miR-331-3p	Mmadhc	1	0	0	1
rno-miR-331-3p	Mmd2_predicte	0	1	0	1
rno-miR-331-3p	Mmp15	0	1	0	1
rno-miR-331-3p	Mobkl2b_predi	0	1	0	1
rno-miR-331-3p	Mpst	0	1	0	1
rno-miR-331-3p	Mrgpre	0	1	0	1
rno-miR-331-3p	Mrpl16	0	1	0	1
rno-miR-331-3p	mrpl24	1	0	0	1
rno-miR-331-3p	Mrpl38	0	1	0	1
rno-miR-331-3p	Mrpl45_predict	0	1	0	1
rno-miR-331-3p	Mrpl4_predicte	0	1	0	1
rno-miR-331-3p	Mrps12_predic	0	1	0	1
rno-miR-331-3p	Mrps18b	0	1	0	1
rno-miR-331-3p	Mrrf	1	0	0	1
rno-miR-331-3p	Mtnr1b	0	1	0	1
rno-miR-331-3p	Mtor	1	0	0	1
rno-miR-331-3p	Mtpn	1	0	0	1
rno-miR-331-3p	Mtvr2	0	1	0	1
rno-miR-331-3p	Muted_predicte	0	1	0	1
rno-miR-331-3p	Mxra8	1	0	0	1
rno-miR-331-3p	Myl3	0	1	0	1
rno-miR-331-3p	Mylk2	1	0	0	1
rno-miR-331-3p	Myo1d	1	0	0	1
rno-miR-331-3p	Myo1e	0	1	0	1
rno-miR-331-3p	Myst2	1	0	0	1
rno-miR-331-3p	N4bp3	1	0	0	1
rno-miR-331-3p	Nags_predicte	0	1	0	1
rno-miR-331-3p	Narfl	0	1	0	1
rno-miR-331-3p	Ncdn	0	1	0	1
rno-miR-331-3p	Ndor1_predicte	0	1	0	1
rno-miR-331-3p	Ndufa12_predi	0	1	0	1
rno-miR-331-3p	NEST_RAT	0	1	0	1
rno-miR-331-3p	Net1	1	0	0	1
rno-miR-331-3p	Nf2	0	1	0	1
rno-miR-331-3p	Nfatc2_predict	0	1	0	1
rno-miR-331-3p	Nolc1	1	0	0	1

rno-miR-331-3p	Nos1ap	1	0	0	1
rno-miR-331-3p	Nov	0	1	0	1
rno-miR-331-3p	Np	0	1	0	1
rno-miR-331-3p	NP_00100767	0	1	0	1
rno-miR-331-3p	NP_00100772	0	1	0	1
rno-miR-331-3p	NP_00102092	0	1	0	1
rno-miR-331-3p	NP_00107340	0	1	0	1
rno-miR-331-3p	NP_00107425	0	1	0	1
rno-miR-331-3p	NP_110493.1	0	1	0	1
rno-miR-331-3p	NP_113755.1	0	1	0	1
rno-miR-331-3p	NP_620211.2	0	1	0	1
rno-miR-331-3p	Npepl1	0	1	0	1
rno-miR-331-3p	Nr1d1	0	1	0	1
rno-miR-331-3p	Nr2c2	1	0	0	1
rno-miR-331-3p	Nr4a3	1	0	0	1
rno-miR-331-3p	Nrap_predicte	0	1	0	1
rno-miR-331-3p	Nrcam	1	0	0	1
rno-miR-331-3p	Nrg2	0	1	0	1
rno-miR-331-3p	Nt5c	0	1	0	1
rno-miR-331-3p	Nt5c3_predicte	0	1	0	1
rno-miR-331-3p	Ntng2_predicte	0	1	0	1
rno-miR-331-3p	Ntrk2	1	0	0	1
rno-miR-331-3p	Ntsr1_predicte	0	1	0	1
rno-miR-331-3p	Nuak2	1	0	0	1
rno-miR-331-3p	Nucb1	1	0	0	1
rno-miR-331-3p	Nudt6	1	0	0	1
rno-miR-331-3p	null	0	1	0	1
rno-miR-331-3p	Nup188	0	1	0	1
rno-miR-331-3p	Nup98	0	1	0	1
rno-miR-331-3p	O35282_RAT	0	1	0	1
rno-miR-331-3p	O88943-7	0	1	0	1
rno-miR-331-3p	O89037_RAT	0	1	0	1
rno-miR-331-3p	Odf2	1	0	0	1
rno-miR-331-3p	Ogg1	0	1	0	1
rno-miR-331-3p	Olig3_predicte	0	1	0	1
rno-miR-331-3p	Olr1096_predi	0	1	0	1
rno-miR-331-3p	Olr1366	1	0	0	1
rno-miR-331-3p	Olr1743_predi	0	1	0	1
rno-miR-331-3p	Olr748_predict	0	1	0	1
rno-miR-331-3p	Olr956_predict	0	1	0	1
rno-miR-331-3p	Optc_predicted	0	1	0	1
rno-miR-331-3p	Orc5l	1	0	0	1
rno-miR-331-3p	Os-9	0	1	0	1
rno-miR-331-3p	Ostf1	1	0	0	1
rno-miR-331-3p	Otof	0	1	0	1
rno-miR-331-3p	Ovol1_predicte	0	1	0	1
rno-miR-331-3p	Oxt	0	1	0	1
rno-miR-331-3p	P2rx7	1	0	0	1
rno-miR-331-3p	P2ry2	0	1	0	1
rno-miR-331-3p	P2ry6	1	0	0	1
rno-miR-331-3p	Pabpn1	0	0	1	1

rno-miR-331-3p	Padi3	1	0	0	1
rno-miR-331-3p	Pah	1	0	0	1
rno-miR-331-3p	Palmd	0	1	0	1
rno-miR-331-3p	Parp1	0	1	0	1
rno-miR-331-3p	Pcaf	1	0	0	1
rno-miR-331-3p	Pcgf6	1	0	0	1
rno-miR-331-3p	Pde5a	0	1	0	1
rno-miR-331-3p	Pde6h	0	1	0	1
rno-miR-331-3p	Pdgfrl	0	1	0	1
rno-miR-331-3p	Pdzk1ip1	0	1	0	1
rno-miR-331-3p	Pef1	1	0	0	1
rno-miR-331-3p	Peflin	0	1	0	1
rno-miR-331-3p	Pgrmc2	1	0	0	1
rno-miR-331-3p	Phactr1	1	0	0	1
rno-miR-331-3p	Phc2	0	0	1	1
rno-miR-331-3p	Phf7	0	1	0	1
rno-miR-331-3p	Phlda2_predic	0	1	0	1
rno-miR-331-3p	Phlpp1	1	0	0	1
rno-miR-331-3p	Pigt_predicted	0	1	0	1
rno-miR-331-3p	Pik3c3	1	0	0	1
rno-miR-331-3p	Pim1	0	1	0	1
rno-miR-331-3p	Pipox	1	0	0	1
rno-miR-331-3p	Pir	1	0	0	1
rno-miR-331-3p	Pkm2	1	0	0	1
rno-miR-331-3p	Pla2g4b_predi	0	1	0	1
rno-miR-331-3p	Plcd1	0	1	0	1
rno-miR-331-3p	Plcg1	1	0	0	1
rno-miR-331-3p	Plekhm2_pred	0	1	0	1
rno-miR-331-3p	Plk3	0	1	0	1
rno-miR-331-3p	Pnliprp1	0	1	0	1
rno-miR-331-3p	Por	0	1	0	1
rno-miR-331-3p	Ppox_predicte	0	1	0	1
rno-miR-331-3p	Ppp1r14b	0	1	0	1
rno-miR-331-3p	Ppp2r5b	0	1	0	1
rno-miR-331-3p	Prkcbp1	0	1	0	1
rno-miR-331-3p	Prmt5_predicte	0	1	0	1
rno-miR-331-3p	Prnpip1	0	1	0	1
rno-miR-331-3p	Prps2	1	0	0	1
rno-miR-331-3p	Prss32_predic	0	1	0	1
rno-miR-331-3p	Pscdbp	0	1	0	1
rno-miR-331-3p	Psen1	1	0	0	1
rno-miR-331-3p	Psma3	1	0	0	1
rno-miR-331-3p	Psmb1	0	1	0	1
rno-miR-331-3p	Psmb10	1	0	0	1
rno-miR-331-3p	Psmc3	0	1	0	1
rno-miR-331-3p	Psmd2	0	1	0	1
rno-miR-331-3p	Psme2	1	0	0	1
rno-miR-331-3p	Ptges2_predic	0	1	0	1
rno-miR-331-3p	Ptgis	0	1	0	1
rno-miR-331-3p	Pthr1	0	1	0	1
rno-miR-331-3p	Ptk7	0	1	0	1

rno-miR-331-3p	Ptpn18	1	0	0	1
rno-miR-331-3p	Ptpn2	1	0	0	1
rno-miR-331-3p	Ptpn7	1	0	0	1
rno-miR-331-3p	Ptpns1	0	1	0	1
rno-miR-331-3p	Ptprn2	1	0	0	1
rno-miR-331-3p	Pvrl1	0	1	0	1
rno-miR-331-3p	Pygm	0	1	0	1
rno-miR-331-3p	Q3TWM1_MO	0	1	0	1
rno-miR-331-3p	Q3TZG3_MOU	0	1	0	1
rno-miR-331-3p	Q4G025_RAT	0	1	0	1
rno-miR-331-3p	Q4G028_RAT	0	1	0	1
rno-miR-331-3p	Q5XHY1_RAT	0	1	0	1
rno-miR-331-3p	Q62916_RAT	0	1	0	1
rno-miR-331-3p	Q63717_RAT	0	1	0	1
rno-miR-331-3p	Q63991_RAT	0	1	0	1
rno-miR-331-3p	Q6MGB2_RAT	0	1	0	1
rno-miR-331-3p	Q6QI81_RAT	0	1	0	1
rno-miR-331-3p	Q7TSP3_RAT	0	1	0	1
rno-miR-331-3p	Q8CIE4_MOU	0	1	0	1
rno-miR-331-3p	Q8K484_RAT	0	1	0	1
rno-miR-331-3p	Rab31	1	0	0	1
rno-miR-331-3p	Rab37	0	1	0	1
rno-miR-331-3p	Rab3a	1	0	0	1
rno-miR-331-3p	Raf1	1	0	0	1
rno-miR-331-3p	Rai16_predicte	0	1	0	1
rno-miR-331-3p	Rap2ip	0	1	0	1
rno-miR-331-3p	Rasal1	0	1	0	1
rno-miR-331-3p	Rasgrp2_predi	0	1	0	1
rno-miR-331-3p	Rasl11a	0	1	0	1
rno-miR-331-3p	Rassf7_predic	0	1	0	1
rno-miR-331-3p	Rbm14	0	1	0	1
rno-miR-331-3p	Reep6	1	0	0	1
rno-miR-331-3p	Rell2	1	0	0	1
rno-miR-331-3p	RGD1303003	1	0	0	1
rno-miR-331-3p	RGD1303130	0	1	0	1
rno-miR-331-3p	RGD1304706	0	1	0	1
rno-miR-331-3p	RGD1304931	0	1	0	1
rno-miR-331-3p	RGD1304952	0	1	0	1
rno-miR-331-3p	RGD1305052_	0	1	0	1
rno-miR-331-3p	RGD1305094_	0	1	0	1
rno-miR-331-3p	RGD1305457	0	1	0	1
rno-miR-331-3p	RGD1305466	0	1	0	1
rno-miR-331-3p	RGD1305514_	0	1	0	1
rno-miR-331-3p	RGD1305553_	0	1	0	1
rno-miR-331-3p	RGD1305587_	0	1	0	1
rno-miR-331-3p	RGD1305593_	0	1	0	1
rno-miR-331-3p	RGD1305613	0	1	0	1
rno-miR-331-3p	RGD1305687_	0	1	0	1
rno-miR-331-3p	RGD1305821	0	1	0	1
rno-miR-331-3p	RGD1306074_	0	1	0	1
rno-miR-331-3p	RGD1306108	0	1	0	1

rno-miR-331-3p	RGD1306164_	0	1	0	1
rno-miR-331-3p	RGD1306404_	0	1	0	1
rno-miR-331-3p	RGD1306592_	0	1	0	1
rno-miR-331-3p	RGD1306729_	0	1	0	1
rno-miR-331-3p	RGD1306947	0	1	0	1
rno-miR-331-3p	RGD1307018	0	1	0	1
rno-miR-331-3p	RGD1307151_	0	1	0	1
rno-miR-331-3p	RGD1307160_	0	1	0	1
rno-miR-331-3p	RGD1307399_	0	1	0	1
rno-miR-331-3p	RGD1307703_	0	1	0	1
rno-miR-331-3p	RGD1307749_	0	1	0	1
rno-miR-331-3p	RGD1307753_	0	1	0	1
rno-miR-331-3p	RGD1307929	0	1	0	1
rno-miR-331-3p	RGD1307982	0	1	0	1
rno-miR-331-3p	RGD1308123	0	1	0	1
rno-miR-331-3p	RGD1308127	1	0	0	1
rno-miR-331-3p	RGD1308154_	0	1	0	1
rno-miR-331-3p	RGD1308356	0	1	0	1
rno-miR-331-3p	RGD1308384_	0	1	0	1
rno-miR-331-3p	RGD1308665	0	1	0	1
rno-miR-331-3p	RGD1308874	0	1	0	1
rno-miR-331-3p	RGD1309049	1	0	0	1
rno-miR-331-3p	RGD1309085_	0	1	0	1
rno-miR-331-3p	RGD1309228	1	0	0	1
rno-miR-331-3p	RGD1309313	0	1	0	1
rno-miR-331-3p	RGD1309341	0	1	0	1
rno-miR-331-3p	RGD1309400_	0	1	0	1
rno-miR-331-3p	RGD1309710_	0	1	0	1
rno-miR-331-3p	RGD1309871_	0	1	0	1
rno-miR-331-3p	RGD1310008	0	1	0	1
rno-miR-331-3p	RGD1310131_	0	1	0	1
rno-miR-331-3p	RGD1310149_	0	1	0	1
rno-miR-331-3p	RGD1310213_	0	1	0	1
rno-miR-331-3p	RGD1310552_	0	1	0	1
rno-miR-331-3p	RGD1310800_	0	1	0	1
rno-miR-331-3p	RGD1310819	0	0	1	1
rno-miR-331-3p	RGD1310922_	0	1	0	1
rno-miR-331-3p	RGD1311072	0	1	0	1
rno-miR-331-3p	RGD1311154_	0	1	0	1
rno-miR-331-3p	RGD1311334_	0	1	0	1
rno-miR-331-3p	RGD1311475_	0	1	0	1
rno-miR-331-3p	RGD1311698_	0	1	0	1
rno-miR-331-3p	RGD1311710	0	1	0	1
rno-miR-331-3p	RGD1311822_	0	1	0	1
rno-miR-331-3p	RGD1311900_	0	1	0	1
rno-miR-331-3p	RGD1311939_	0	1	0	1
rno-miR-331-3p	RGD1359443	0	1	0	1
rno-miR-331-3p	RGD1359682	0	1	0	1
rno-miR-331-3p	RGD1559497_	0	1	0	1
rno-miR-331-3p	RGD1559693_	0	1	0	1
rno-miR-331-3p	RGD1559716_	0	1	0	1

rno-miR-331-3p	RGD1559981_	0	1	0	1
rno-miR-331-3p	RGD1560076_	0	1	0	1
rno-miR-331-3p	RGD1560108_	0	1	0	1
rno-miR-331-3p	RGD1560139_	0	1	0	1
rno-miR-331-3p	RGD1560187_	0	1	0	1
rno-miR-331-3p	RGD1560286	0	0	1	1
rno-miR-331-3p	RGD1560293_	0	1	0	1
rno-miR-331-3p	RGD1560335_	0	1	0	1
rno-miR-331-3p	RGD1560410_	0	1	0	1
rno-miR-331-3p	RGD1560481_	0	1	0	1
rno-miR-331-3p	RGD1560552_	0	1	0	1
rno-miR-331-3p	RGD1560636_	0	1	0	1
rno-miR-331-3p	RGD1560672_	0	1	0	1
rno-miR-331-3p	RGD1560726_	0	1	0	1
rno-miR-331-3p	RGD1560731_	0	1	0	1
rno-miR-331-3p	RGD1560810_	0	1	0	1
rno-miR-331-3p	RGD1560873_	0	1	0	1
rno-miR-331-3p	RGD1561354_	0	1	0	1
rno-miR-331-3p	RGD1561521_	0	1	0	1
rno-miR-331-3p	RGD1561632_	0	1	0	1
rno-miR-331-3p	RGD1561734_	0	1	0	1
rno-miR-331-3p	RGD1562025_	0	1	0	1
rno-miR-331-3p	RGD1562074_	0	1	0	1
rno-miR-331-3p	RGD1562279_	0	1	0	1
rno-miR-331-3p	RGD1562316_	0	1	0	1
rno-miR-331-3p	RGD1562321_	0	1	0	1
rno-miR-331-3p	RGD1562464_	0	1	0	1
rno-miR-331-3p	RGD1562655_	0	1	0	1
rno-miR-331-3p	RGD1563020_	0	1	0	1
rno-miR-331-3p	RGD1563034_	0	1	0	1
rno-miR-331-3p	RGD1563048_	0	1	0	1
rno-miR-331-3p	RGD1563108_	0	1	0	1
rno-miR-331-3p	RGD1563195_	0	1	0	1
rno-miR-331-3p	RGD1563203_	0	1	0	1
rno-miR-331-3p	RGD1563268_	0	1	0	1
rno-miR-331-3p	RGD1563386_	0	1	0	1
rno-miR-331-3p	RGD1563532_	0	1	0	1
rno-miR-331-3p	RGD1563648_	0	1	0	1
rno-miR-331-3p	RGD1563714_	0	1	0	1
rno-miR-331-3p	RGD1564002_	0	1	0	1
rno-miR-331-3p	RGD1564086_	0	1	0	1
rno-miR-331-3p	RGD1564337_	0	1	0	1
rno-miR-331-3p	RGD1564360_	0	1	0	1
rno-miR-331-3p	RGD1564638_	0	1	0	1
rno-miR-331-3p	RGD1564779_	0	1	0	1
rno-miR-331-3p	RGD1564808_	0	1	0	1
rno-miR-331-3p	RGD1565031_	0	1	0	1
rno-miR-331-3p	RGD1565049_	0	1	0	1
rno-miR-331-3p	RGD1565079_	0	1	0	1
rno-miR-331-3p	RGD1565779_	0	1	0	1
rno-miR-331-3p	RGD1566083_	0	1	0	1

rno-miR-331-3p	RGD1566309_	0	1	0	1
rno-miR-331-3p	RGD1566320_	0	1	0	1
rno-miR-331-3p	RGD735106	0	1	0	1
rno-miR-331-3p	Rgs19	0	1	0	1
rno-miR-331-3p	Rgs4	1	0	0	1
rno-miR-331-3p	Rhbdl6_predic	0	1	0	1
rno-miR-331-3p	Rhcg	0	1	0	1
rno-miR-331-3p	Rhob	0	1	0	1
rno-miR-331-3p	Rhoc_predicte	0	1	0	1
rno-miR-331-3p	Rims4	0	1	0	1
rno-miR-331-3p	Ripx	0	1	0	1
rno-miR-331-3p	RL13_RAT	0	1	0	1
rno-miR-331-3p	RL36_RAT	0	1	0	1
rno-miR-331-3p	Rmnd5b	1	0	0	1
rno-miR-331-3p	Rnf122	0	1	0	1
rno-miR-331-3p	Rnf135	0	1	0	1
rno-miR-331-3p	Rnf150	0	0	1	1
rno-miR-331-3p	Rnf40	0	1	0	1
rno-miR-331-3p	Rnf41	1	0	0	1
rno-miR-331-3p	Rnf44	0	1	0	1
rno-miR-331-3p	Rnpc2	0	1	0	1
rno-miR-331-3p	Rpl28	1	0	0	1
rno-miR-331-3p	Rps11	0	1	0	1
rno-miR-331-3p	Rpusd1_predic	0	1	0	1
rno-miR-331-3p	RRMJ3_RAT	0	1	0	1
rno-miR-331-3p	Rs1	0	1	0	1
rno-miR-331-3p	RT1-A2	0	1	0	1
rno-miR-331-3p	RT1-Bb	0	1	0	1
rno-miR-331-3p	Rtn3	1	0	0	1
rno-miR-331-3p	Runx3	0	1	0	1
rno-miR-331-3p	Rutbc1_predic	0	1	0	1
rno-miR-331-3p	Rutbc3	0	1	0	1
rno-miR-331-3p	S100a16_pred	0	1	0	1
rno-miR-331-3p	S100vp	1	0	0	1
rno-miR-331-3p	Sars1	0	1	0	1
rno-miR-331-3p	Sbf1_predicted	0	1	0	1
rno-miR-331-3p	Scarf2_predict	0	1	0	1
rno-miR-331-3p	Scly	1	0	0	1
rno-miR-331-3p	Scn4a	1	0	0	1
rno-miR-331-3p	Sdc2	1	0	0	1
rno-miR-331-3p	Sec14l2	1	0	0	1
rno-miR-331-3p	Sele	1	0	0	1
rno-miR-331-3p	Sema3g	0	1	0	1
rno-miR-331-3p	Sema6b	1	0	0	1
rno-miR-331-3p	Sept5	1	0	0	1
rno-miR-331-3p	Serpine1	1	0	0	1
rno-miR-331-3p	Serpinf2	0	1	0	1
rno-miR-331-3p	Sertad1	0	1	0	1
rno-miR-331-3p	Sfpi1	0	1	0	1
rno-miR-331-3p	Sfrs10	1	0	0	1
rno-miR-331-3p	Sh2b2	1	0	0	1

rno-miR-331-3p	Sh2d3c_predic	0	1	0	1
rno-miR-331-3p	Sh3bp4	0	1	0	1
rno-miR-331-3p	Shank3	0	1	0	1
rno-miR-331-3p	Shisa5	1	0	0	1
rno-miR-331-3p	Shkbp1_predic	0	1	0	1
rno-miR-331-3p	Sil1	0	1	0	1
rno-miR-331-3p	Sirpa	1	0	0	1
rno-miR-331-3p	Ska1	0	0	1	1
rno-miR-331-3p	Skap1	1	0	0	1
rno-miR-331-3p	Slc12a7	0	1	0	1
rno-miR-331-3p	Slc16a13	0	1	0	1
rno-miR-331-3p	Slc17a5	1	0	0	1
rno-miR-331-3p	Slc17a8	1	0	0	1
rno-miR-331-3p	Slc25a1	1	0	0	1
rno-miR-331-3p	Slc25a10	0	1	0	1
rno-miR-331-3p	Slc25a12	0	1	0	1
rno-miR-331-3p	Slc25a29	1	0	0	1
rno-miR-331-3p	Slc27a3_predi	0	1	0	1
rno-miR-331-3p	Slc27a4	0	1	0	1
rno-miR-331-3p	Slc2a8	0	1	0	1
rno-miR-331-3p	Slc30a2	0	1	0	1
rno-miR-331-3p	Slc35a2	0	1	0	1
rno-miR-331-3p	Slc38a2	1	0	0	1
rno-miR-331-3p	Slc43a1_predi	0	1	0	1
rno-miR-331-3p	Slc43a2_predi	0	1	0	1
rno-miR-331-3p	Slc4a9	1	0	0	1
rno-miR-331-3p	Slc9a3r1	1	0	0	1
rno-miR-331-3p	Slc9a3r2	0	1	0	1
rno-miR-331-3p	Slc9a5	0	1	0	1
rno-miR-331-3p	Smarca2	1	0	0	1
rno-miR-331-3p	Smarcd3	0	1	0	1
rno-miR-331-3p	Snai1	0	1	0	1
rno-miR-331-3p	Snai3_predicte	0	1	0	1
rno-miR-331-3p	Snrp70_predic	0	1	0	1
rno-miR-331-3p	Snw1	0	0	1	1
rno-miR-331-3p	Snx15	0	1	0	1
rno-miR-331-3p	Snx17	0	1	0	1
rno-miR-331-3p	Snx8_predicte	0	1	0	1
rno-miR-331-3p	Sostdc1	1	0	0	1
rno-miR-331-3p	Sox4	0	1	0	1
rno-miR-331-3p	Spint1	0	1	0	1
rno-miR-331-3p	Sprn	0	1	0	1
rno-miR-331-3p	Srebf2	1	0	0	1
rno-miR-331-3p	Srpx2_predicte	0	1	0	1
rno-miR-331-3p	Sstr3	1	0	0	1
rno-miR-331-3p	St3gal6	1	0	0	1
rno-miR-331-3p	Stac3_predicte	0	1	0	1
rno-miR-331-3p	Stam2	1	0	0	1
rno-miR-331-3p	Stard10	0	1	0	1
rno-miR-331-3p	Stard5_predict	0	1	0	1
rno-miR-331-3p	Stat3	1	0	0	1

rno-miR-331-3p	Stat5a	1	0	0	1
rno-miR-331-3p	Stk22s1	0	1	0	1
rno-miR-331-3p	Stk39	1	0	0	1
rno-miR-331-3p	Stmn3	0	1	0	1
rno-miR-331-3p	Stra6	1	0	0	1
rno-miR-331-3p	Stx7	1	0	0	1
rno-miR-331-3p	Surf6	1	0	0	1
rno-miR-331-3p	Svop	1	0	0	1
rno-miR-331-3p	Syngr4	1	0	0	1
rno-miR-331-3p	Syt11	1	0	0	1
rno-miR-331-3p	Syt3	0	1	0	1
rno-miR-331-3p	Sytl1	0	1	0	1
rno-miR-331-3p	Tacr1	1	0	0	1
rno-miR-331-3p	Taok2	0	1	0	1
rno-miR-331-3p	Tbkbp1	1	0	0	1
rno-miR-331-3p	Tbx2_predicte	0	1	0	1
rno-miR-331-3p	Tceb1	1	0	0	1
rno-miR-331-3p	Tcfap2b_predi	0	1	0	1
rno-miR-331-3p	Tchp	0	1	0	1
rno-miR-331-3p	Tekt1	0	1	0	1
rno-miR-331-3p	Tex101	0	1	0	1
rno-miR-331-3p	Tex261	0	1	0	1
rno-miR-331-3p	Tfpi	1	0	0	1
rno-miR-331-3p	Tgfb1	0	1	0	1
rno-miR-331-3p	Tgfb2	1	0	0	1
rno-miR-331-3p	Tgm4	0	1	0	1
rno-miR-331-3p	Thbs4	0	1	0	1
rno-miR-331-3p	Them4	0	1	0	1
rno-miR-331-3p	Thop1	1	0	0	1
rno-miR-331-3p	Timm13	0	1	0	1
rno-miR-331-3p	Tinag	1	0	0	1
rno-miR-331-3p	Tlr10_predicte	0	1	0	1
rno-miR-331-3p	Tmem103_pre	0	1	0	1
rno-miR-331-3p	Tmem106a	0	1	0	1
rno-miR-331-3p	Tmem11_pred	0	1	0	1
rno-miR-331-3p	Tmem39b	0	1	0	1
rno-miR-331-3p	Tmem40	0	1	0	1
rno-miR-331-3p	Tmem52	0	1	0	1
rno-miR-331-3p	Tmem85	0	0	1	1
rno-miR-331-3p	Tmem93_pred	0	1	0	1
rno-miR-331-3p	Tmprss2	0	1	0	1
rno-miR-331-3p	Tmprss6_pred	0	1	0	1
rno-miR-331-3p	Tnfaip2	0	1	0	1
rno-miR-331-3p	Tnfrsf12a	0	1	0	1
rno-miR-331-3p	Tnfrsf1a	0	1	0	1
rno-miR-331-3p	Tnfrsf5	0	1	0	1
rno-miR-331-3p	Tnfrsf8	1	0	0	1
rno-miR-331-3p	Tnfsf12	0	1	0	1
rno-miR-331-3p	Tnfsf13	1	0	0	1
rno-miR-331-3p	Tnip2	0	1	0	1
rno-miR-331-3p	Tob2	1	0	0	1

rno-miR-331-3p	Tomm40	0	1	0	1
rno-miR-331-3p	Tomm70a	1	0	0	1
rno-miR-331-3p	Tpcn2_predict	0	1	0	1
rno-miR-331-3p	Tradd	0	1	0	1
rno-miR-331-3p	Trappc4	0	1	0	1
rno-miR-331-3p	Trpm2	0	1	0	1
rno-miR-331-3p	Tsku	1	0	0	1
rno-miR-331-3p	Tspan18_pred	0	1	0	1
rno-miR-331-3p	Tssc4	0	1	0	1
rno-miR-331-3p	Tssk1	0	1	0	1
rno-miR-331-3p	Ttc16	0	1	0	1
rno-miR-331-3p	Tubd1_predict	0	1	0	1
rno-miR-331-3p	Tubg1	0	1	0	1
rno-miR-331-3p	Txlnb	0	0	1	1
rno-miR-331-3p	Txn2	0	1	0	1
rno-miR-331-3p	Ubl3	1	0	0	1
rno-miR-331-3p	Unc119	0	1	0	1
rno-miR-331-3p	Unc13b	0	1	0	1
rno-miR-331-3p	Unc5d	0	1	0	1
rno-miR-331-3p	Usp39_predict	0	1	0	1
rno-miR-331-3p	Usp5	0	1	0	1
rno-miR-331-3p	Vat1	1	0	0	1
rno-miR-331-3p	Vcam1	1	0	0	1
rno-miR-331-3p	Vmd2l3_predic	0	1	0	1
rno-miR-331-3p	Vom2r27	1	0	0	1
rno-miR-331-3p	Vps54	1	0	0	1
rno-miR-331-3p	Wnt2	0	1	0	1
rno-miR-331-3p	Xylt2	0	1	0	1
rno-miR-331-3p	Ypel3	0	1	0	1
rno-miR-331-3p	Yrdc	1	0	0	1
rno-miR-331-3p	Zbtb7a	0	1	0	1
rno-miR-331-3p	Zcwpw2	0	1	0	1
rno-miR-331-3p	Zfp365	0	1	0	1
rno-miR-331-3p	Zfp410_predic	0	1	0	1
rno-miR-331-3p	Zfp46	0	0	1	1
rno-miR-331-3p	Zfp46_predicte	0	1	0	1
rno-miR-331-3p	Zfp523_predic	0	1	0	1
rno-miR-331-3p	Zfp598_predic	0	1	0	1
rno-miR-331-3p	Zfp641_predic	0	1	0	1
rno-miR-331-3p	Zfp652	0	0	1	1
rno-miR-331-3p	Zfyve19	1	0	0	1
rno-miR-331-3p	Znf394	1	0	0	1
rno-miR-331-3p	Znf467	1	0	0	1
rno-miR-331-3p	Znf655	1	0	0	1
rno-miR-34c-3p	1110001A16R	0	1	0	1
rno-miR-34c-3p	1110001D15R	0	1	0	1
rno-miR-34c-3p	1110006O17R	0	1	0	1
rno-miR-34c-3p	3110082D06R	0	1	0	1
rno-miR-34c-3p	4930513O06R	0	1	0	1
rno-miR-34c-3p	4930571C24R	0	1	0	1
rno-miR-34c-3p	4932416N17R	0	1	0	1

rno-miR-34c-3p	5730437N04R	0	1	0	1
rno-miR-34c-3p	6030443O07R	0	1	0	1
rno-miR-34c-3p	9630041N07R	0	1	0	1
rno-miR-34c-3p	A130038H09R	0	1	0	1
rno-miR-34c-3p	A2BHQ4_MOU	0	1	0	1
rno-miR-34c-3p	A430110N23R	0	1	0	1
rno-miR-34c-3p	Aasdhppt_pred	0	1	0	1
rno-miR-34c-3p	Abca15_predic	0	1	0	1
rno-miR-34c-3p	Abca4_predict	0	1	0	1
rno-miR-34c-3p	Abcb1a	1	0	0	1
rno-miR-34c-3p	Abhd14b	1	0	0	1
rno-miR-34c-3p	Acsl5	0	1	0	1
rno-miR-34c-3p	Actr10	0	1	0	1
rno-miR-34c-3p	Actr3	1	0	0	1
rno-miR-34c-3p	Adad2	0	1	0	1
rno-miR-34c-3p	Adam33	0	1	0	1
rno-miR-34c-3p	Adamts1	1	0	0	1
rno-miR-34c-3p	Adamts3_pred	0	1	0	1
rno-miR-34c-3p	Adamts6_pred	0	1	0	1
rno-miR-34c-3p	Adcyap1	1	0	0	1
rno-miR-34c-3p	Adi1	1	0	0	1
rno-miR-34c-3p	Admr	0	1	0	1
rno-miR-34c-3p	Adrb2	0	1	0	1
rno-miR-34c-3p	Adrm1	0	1	0	1
rno-miR-34c-3p	Af6	0	1	0	1
rno-miR-34c-3p	Akap12	0	1	0	1
rno-miR-34c-3p	Akap6	1	0	0	1
rno-miR-34c-3p	Akr1c12_predi	0	1	0	1
rno-miR-34c-3p	Akt2	0	1	0	1
rno-miR-34c-3p	Alg10	1	0	0	1
rno-miR-34c-3p	Alg9_predicted	0	1	0	1
rno-miR-34c-3p	Ambn	0	1	0	1
rno-miR-34c-3p	Amd1	1	0	0	1
rno-miR-34c-3p	Anxa6	0	1	0	1
rno-miR-34c-3p	Aqp11	1	0	0	1
rno-miR-34c-3p	Aqp2	1	0	0	1
rno-miR-34c-3p	Araf	1	0	0	1
rno-miR-34c-3p	Arhgef19_pred	0	1	0	1
rno-miR-34c-3p	Arhgef9	1	0	0	1
rno-miR-34c-3p	Arl4a	1	0	0	1
rno-miR-34c-3p	Arl5b	1	0	0	1
rno-miR-34c-3p	Arl8b	1	0	0	1
rno-miR-34c-3p	Armc4_predict	0	1	0	1
rno-miR-34c-3p	Arpc1a	0	1	0	1
rno-miR-34c-3p	Art3	0	1	0	1
rno-miR-34c-3p	Asb6	0	1	0	1
rno-miR-34c-3p	Atad1	1	0	0	1
rno-miR-34c-3p	Atf7ip2	1	0	0	1
rno-miR-34c-3p	Atn1	0	1	0	1
rno-miR-34c-3p	Atp1b1	0	1	0	1
rno-miR-34c-3p	Atp1b4	1	0	0	1

rno-miR-34c-3p	Atp2c1	0	1	0	1
rno-miR-34c-3p	Atp5sl	1	0	0	1
rno-miR-34c-3p	Atp8b4	0	1	0	1
rno-miR-34c-3p	B3galt1_predic	0	1	0	1
rno-miR-34c-3p	B3galt6_predic	0	1	0	1
rno-miR-34c-3p	B3gnt4_predic	0	1	0	1
rno-miR-34c-3p	B4galt6	1	0	0	1
rno-miR-34c-3p	Bace1	0	1	0	1
rno-miR-34c-3p	Bag3	0	1	0	1
rno-miR-34c-3p	Baiap2	0	1	0	1
rno-miR-34c-3p	Bat5	1	0	0	1
rno-miR-34c-3p	Bcap29	1	0	0	1
rno-miR-34c-3p	Bcl10	0	1	0	1
rno-miR-34c-3p	Bcl2a1	0	1	0	1
rno-miR-34c-3p	Bcl2l14	1	0	0	1
rno-miR-34c-3p	Bcorl1	0	1	0	1
rno-miR-34c-3p	Bcs1l	0	1	0	1
rno-miR-34c-3p	Bdnf	1	0	0	1
rno-miR-34c-3p	Bhlhb2	0	1	0	1
rno-miR-34c-3p	Bhlhe41	0	0	1	1
rno-miR-34c-3p	Bicc1_predicte	0	1	0	1
rno-miR-34c-3p	Bmp5_predicte	0	1	0	1
rno-miR-34c-3p	Bmpr2	0	1	0	1
rno-miR-34c-3p	Bnip3	1	0	0	1
rno-miR-34c-3p	Brf2	0	1	0	1
rno-miR-34c-3p	Brpf3_predicte	0	1	0	1
rno-miR-34c-3p	Btbd14a	0	1	0	1
rno-miR-34c-3p	Btbd15	0	1	0	1
rno-miR-34c-3p	Btbd4_predicte	0	1	0	1
rno-miR-34c-3p	Btbd5_predicte	0	1	0	1
rno-miR-34c-3p	Btg1	1	0	0	1
rno-miR-34c-3p	Btg4	1	0	0	1
rno-miR-34c-3p	C12orf10	0	1	0	1
rno-miR-34c-3p	Cadm1	1	0	0	1
rno-miR-34c-3p	Calb1	1	0	0	1
rno-miR-34c-3p	Cald1	1	0	0	1
rno-miR-34c-3p	Camkk1	1	0	0	1
rno-miR-34c-3p	Cand1	0	1	0	1
rno-miR-34c-3p	Caprin1	0	1	0	1
rno-miR-34c-3p	Car15_predicte	0	1	0	1
rno-miR-34c-3p	Car2	1	0	0	1
rno-miR-34c-3p	Car9_predicted	0	1	0	1
rno-miR-34c-3p	Casc5	0	1	0	1
rno-miR-34c-3p	Casp8ap2_pre	0	1	0	1
rno-miR-34c-3p	Cav2	0	0	1	1
rno-miR-34c-3p	Cbfa2t1_predic	0	1	0	1
rno-miR-34c-3p	Ccdc63	0	1	0	1
rno-miR-34c-3p	Ccna2	1	0	0	1
rno-miR-34c-3p	Cct2	0	1	0	1
rno-miR-34c-3p	Cct3	1	0	0	1
rno-miR-34c-3p	Cct6a	1	0	0	1

rno-miR-34c-3p	Cd209a_predic	0	1	0	1
rno-miR-34c-3p	Cd22_predicte	0	1	0	1
rno-miR-34c-3p	Cd302	0	1	0	1
rno-miR-34c-3p	Cd38	1	0	0	1
rno-miR-34c-3p	Cd3e_predicte	0	1	0	1
rno-miR-34c-3p	Cd3z	0	1	0	1
rno-miR-34c-3p	Cd53	1	0	0	1
rno-miR-34c-3p	Cdc27	0	1	0	1
rno-miR-34c-3p	Cdca1	0	1	0	1
rno-miR-34c-3p	Cdh1	1	0	0	1
rno-miR-34c-3p	Cdk17	0	0	1	1
rno-miR-34c-3p	Cdk5rap1	0	1	0	1
rno-miR-34c-3p	Cenpf	0	1	0	1
rno-miR-34c-3p	Ces7	0	1	0	1
rno-miR-34c-3p	Cetn1	0	1	0	1
rno-miR-34c-3p	Cfh	0	1	0	1
rno-miR-34c-3p	Chaf1b	0	1	0	1
rno-miR-34c-3p	Chd2_predicte	0	1	0	1
rno-miR-34c-3p	Chd3	0	1	0	1
rno-miR-34c-3p	Chi3l4_predict	0	1	0	1
rno-miR-34c-3p	Chmp7_predic	0	1	0	1
rno-miR-34c-3p	Chp	0	1	0	1
rno-miR-34c-3p	Chrm2	0	1	0	1
rno-miR-34c-3p	Chrng	0	1	0	1
rno-miR-34c-3p	Chst3	0	1	0	1
rno-miR-34c-3p	CK5P2_RAT	0	1	0	1
rno-miR-34c-3p	Clcn1	0	1	0	1
rno-miR-34c-3p	Clec4d	0	1	0	1
rno-miR-34c-3p	Clock	0	1	0	1
rno-miR-34c-3p	Clpx	1	0	0	1
rno-miR-34c-3p	Cmas	0	1	0	1
rno-miR-34c-3p	Cmbl	1	0	0	1
rno-miR-34c-3p	Cnnm3_predic	0	1	0	1
rno-miR-34c-3p	Cnr1	0	0	1	1
rno-miR-34c-3p	Cntn3	1	0	0	1
rno-miR-34c-3p	Coasy	0	1	0	1
rno-miR-34c-3p	Commd9	1	0	0	1
rno-miR-34c-3p	Cox8h	0	1	0	1
rno-miR-34c-3p	Cpne9	0	1	0	1
rno-miR-34c-3p	Crmp1	1	0	0	1
rno-miR-34c-3p	Crsp9	0	1	0	1
rno-miR-34c-3p	Csf3r_predicte	0	1	0	1
rno-miR-34c-3p	Csn2	1	0	0	1
rno-miR-34c-3p	Cttnbp2	0	1	0	1
rno-miR-34c-3p	Ctxn	0	1	0	1
rno-miR-34c-3p	Cuedc2	0	1	0	1
rno-miR-34c-3p	Cuzd1	0	1	0	1
rno-miR-34c-3p	Cxcl12	1	0	0	1
rno-miR-34c-3p	Cybasc3	1	0	0	1
rno-miR-34c-3p	Cyp2j4	1	0	0	1
rno-miR-34c-3p	Cyp2j9	0	1	0	1

rno-miR-34c-3p	Cyp2s1	0	1	0	1
rno-miR-34c-3p	Cyp4a14	0	1	0	1
rno-miR-34c-3p	Cysltr2	0	1	0	1
rno-miR-34c-3p	Cyyr1	1	0	0	1
rno-miR-34c-3p	D030022P06R	0	1	0	1
rno-miR-34c-3p	D630045J12R	0	1	0	1
rno-miR-34c-3p	Dao	1	0	0	1
rno-miR-34c-3p	Dao1	0	1	0	1
rno-miR-34c-3p	Dazap2	1	0	0	1
rno-miR-34c-3p	Dclk1	1	0	0	1
rno-miR-34c-3p	Ddc	0	1	0	1
rno-miR-34c-3p	Ddx19a	1	0	0	1
rno-miR-34c-3p	Ddx5	1	0	0	1
rno-miR-34c-3p	Defa-rs1	0	1	0	1
rno-miR-34c-3p	Defb29	0	1	0	1
rno-miR-34c-3p	Dennd4c	0	1	0	1
rno-miR-34c-3p	DHB13_RAT	0	1	0	1
rno-miR-34c-3p	Dhrs1	1	0	0	1
rno-miR-34c-3p	Dhrs13	0	1	0	1
rno-miR-34c-3p	Dhx9	0	0	1	1
rno-miR-34c-3p	Dhx9_predicte	0	1	0	1
rno-miR-34c-3p	Dio1	0	1	0	1
rno-miR-34c-3p	Dmd	0	1	0	1
rno-miR-34c-3p	Dmrt2_predict	0	1	0	1
rno-miR-34c-3p	Dmtf1	1	0	0	1
rno-miR-34c-3p	Dnahc5	0	1	0	1
rno-miR-34c-3p	Dnttip2	0	1	0	1
rno-miR-34c-3p	Dpys	1	0	0	1
rno-miR-34c-3p	Dr1	1	0	0	1
rno-miR-34c-3p	Drd1a	1	0	0	1
rno-miR-34c-3p	Dsc2	0	0	1	1
rno-miR-34c-3p	Dscr6_predicte	0	1	0	1
rno-miR-34c-3p	Dspp	0	1	0	1
rno-miR-34c-3p	Dtx3l	0	1	0	1
rno-miR-34c-3p	Dusp8_predict	0	1	0	1
rno-miR-34c-3p	Dut	1	0	0	1
rno-miR-34c-3p	Dynll1	1	0	0	1
rno-miR-34c-3p	E430025E21R	0	1	0	1
rno-miR-34c-3p	Edg8	0	1	0	1
rno-miR-34c-3p	Eed_predicted	0	1	0	1
rno-miR-34c-3p	Efna1	1	0	0	1
rno-miR-34c-3p	Eftud2	0	1	0	1
rno-miR-34c-3p	Eif3s8	0	1	0	1
rno-miR-34c-3p	Eif4a2	1	0	0	1
rno-miR-34c-3p	Elavl2	1	0	0	1
rno-miR-34c-3p	Elavl3	0	1	0	1
rno-miR-34c-3p	Elmo1_predict	0	1	0	1
rno-miR-34c-3p	Elp2	1	0	0	1
rno-miR-34c-3p	Eltd1	1	0	0	1
rno-miR-34c-3p	Eme1_predicte	0	1	0	1
rno-miR-34c-3p	ENSMUSG000	0	1	0	1

rno-miR-34c-3p	ENSMUSG000	0	1	0	1
rno-miR-34c-3p	ENSMUSG000	0	1	0	1
rno-miR-34c-3p	Entpd5	0	1	0	1
rno-miR-34c-3p	Epdr1	1	0	0	1
rno-miR-34c-3p	Epsti1	0	0	1	1
rno-miR-34c-3p	Erp29	1	0	0	1
rno-miR-34c-3p	Esm1	1	0	0	1
rno-miR-34c-3p	Evc	0	1	0	1
rno-miR-34c-3p	Exoc5	0	1	0	1
rno-miR-34c-3p	Eya2	0	1	0	1
rno-miR-34c-3p	F2r	0	1	0	1
rno-miR-34c-3p	Fabp9	1	0	0	1
rno-miR-34c-3p	FAKD3_RAT	0	1	0	1
rno-miR-34c-3p	Fam101b	1	0	0	1
rno-miR-34c-3p	Fam175b	0	0	1	1
rno-miR-34c-3p	Fam21c	1	0	0	1
rno-miR-34c-3p	Fam48a	1	0	0	1
rno-miR-34c-3p	Fam96a	1	0	0	1
rno-miR-34c-3p	Farsb	1	0	0	1
rno-miR-34c-3p	Farsla	0	1	0	1
rno-miR-34c-3p	Fbln5	1	0	0	1
rno-miR-34c-3p	Fbxo34_predic	0	1	0	1
rno-miR-34c-3p	Fbxo39	0	1	0	1
rno-miR-34c-3p	Fen1	1	0	0	1
rno-miR-34c-3p	Fgfr1op2	1	0	0	1
rno-miR-34c-3p	Fgfr2	0	1	0	1
rno-miR-34c-3p	Fh1	0	1	0	1
rno-miR-34c-3p	Figf	1	0	0	1
rno-miR-34c-3p	Flt3	0	1	0	1
rno-miR-34c-3p	Fmnl2	0	1	0	1
rno-miR-34c-3p	Fmr1	1	0	0	1
rno-miR-34c-3p	Foxg1	1	0	0	1
rno-miR-34c-3p	Fryl	0	1	0	1
rno-miR-34c-3p	Fuk_predicted	0	1	0	1
rno-miR-34c-3p	Gabra3	0	1	0	1
rno-miR-34c-3p	Gad1	1	0	0	1
rno-miR-34c-3p	Gata2	1	0	0	1
rno-miR-34c-3p	Gbp5_predicte	0	1	0	1
rno-miR-34c-3p	Gcg	0	1	0	1
rno-miR-34c-3p	Gdf10	1	0	0	1
rno-miR-34c-3p	Gdi1	1	0	0	1
rno-miR-34c-3p	Gfra3	0	1	0	1
rno-miR-34c-3p	Ggnbp2	1	0	0	1
rno-miR-34c-3p	Gimap9	1	0	0	1
rno-miR-34c-3p	Gja1	1	0	0	1
rno-miR-34c-3p	Gja5	1	0	0	1
rno-miR-34c-3p	Glod4	0	1	0	1
rno-miR-34c-3p	Glt6d1_predict	0	1	0	1
rno-miR-34c-3p	Gm904	0	1	0	1
rno-miR-34c-3p	Gnai3	1	0	0	1
rno-miR-34c-3p	Gng10	0	1	0	1

rno-miR-34c-3p	Golga7	1	0	0	1
rno-miR-34c-3p	Gpc4	0	1	0	1
rno-miR-34c-3p	Gpm6b	0	1	0	1
rno-miR-34c-3p	Gpr43	0	1	0	1
rno-miR-34c-3p	Gpt2	1	0	0	1
rno-miR-34c-3p	Grb7	1	0	0	1
rno-miR-34c-3p	Gria4	1	0	0	1
rno-miR-34c-3p	Gsr	0	1	0	1
rno-miR-34c-3p	Gtf3c1	0	1	0	1
rno-miR-34c-3p	Has2	1	0	0	1
rno-miR-34c-3p	Hcls1	0	1	0	1
rno-miR-34c-3p	Hebp1_predict	0	1	0	1
rno-miR-34c-3p	Herc4	0	1	0	1
rno-miR-34c-3p	Hfe	0	1	0	1
rno-miR-34c-3p	Hmg20a_predi	0	1	0	1
rno-miR-34c-3p	Hmha1_predic	0	1	0	1
rno-miR-34c-3p	Hnrnpa0	0	0	1	1
rno-miR-34c-3p	Hnrnpf	1	0	0	1
rno-miR-34c-3p	Hnrnpk	1	0	0	1
rno-miR-34c-3p	Hnrpa0	0	1	0	1
rno-miR-34c-3p	Hoxd13	0	0	1	1
rno-miR-34c-3p	Hrasls3	0	1	0	1
rno-miR-34c-3p	Hsdl1	1	0	0	1
rno-miR-34c-3p	Htatsf1	0	1	0	1
rno-miR-34c-3p	I830077J02Rik	0	1	0	1
rno-miR-34c-3p	IF4E_RAT	0	1	0	1
rno-miR-34c-3p	Ifih1	0	0	1	1
rno-miR-34c-3p	Ighmbp2	0	1	0	1
rno-miR-34c-3p	Igsf9	0	1	0	1
rno-miR-34c-3p	Il11ra1	1	0	0	1
rno-miR-34c-3p	Il16	0	1	0	1
rno-miR-34c-3p	Il17f	1	0	0	1
rno-miR-34c-3p	Il1f6_predicted	0	1	0	1
rno-miR-34c-3p	Il1r2	0	1	0	1
rno-miR-34c-3p	Il1rl1	1	0	0	1
rno-miR-34c-3p	Il23a	1	0	0	1
rno-miR-34c-3p	Ilf3	0	1	0	1
rno-miR-34c-3p	Immp2l	0	1	0	1
rno-miR-34c-3p	Impa2	1	0	0	1
rno-miR-34c-3p	Ing3	1	0	0	1
rno-miR-34c-3p	Inpp5a_predict	0	1	0	1
rno-miR-34c-3p	Inpp5f_predict	0	1	0	1
rno-miR-34c-3p	Ipo7_predicted	0	1	0	1
rno-miR-34c-3p	Irak2	1	0	0	1
rno-miR-34c-3p	Ireb2	1	0	0	1
rno-miR-34c-3p	Isg20l1_predic	0	1	0	1
rno-miR-34c-3p	Itga2	0	1	0	1
rno-miR-34c-3p	Itpr1	1	0	0	1
rno-miR-34c-3p	Itpr2	0	0	1	1
rno-miR-34c-3p	Jag1	1	0	0	1
rno-miR-34c-3p	Jmjd6	0	1	0	1

rno-miR-34c-3p	Ka40	0	1	0	1
rno-miR-34c-3p	Kcne1	1	0	0	1
rno-miR-34c-3p	Kcnk10	1	0	0	1
rno-miR-34c-3p	Kcnq3	1	0	0	1
rno-miR-34c-3p	Kcns2	0	1	0	1
rno-miR-34c-3p	Kif2	0	1	0	1
rno-miR-34c-3p	Kif9_predicted	0	1	0	1
rno-miR-34c-3p	Kir3dl1	0	1	0	1
rno-miR-34c-3p	Kitl	0	1	0	1
rno-miR-34c-3p	Klf9	1	0	0	1
rno-miR-34c-3p	Kpna1	1	0	0	1
rno-miR-34c-3p	Kpnb1	0	1	0	1
rno-miR-34c-3p	Lalba	0	1	0	1
rno-miR-34c-3p	Laptm4b	1	0	0	1
rno-miR-34c-3p	Las1l_predicte	0	1	0	1
rno-miR-34c-3p	Lass5_predicte	0	1	0	1
rno-miR-34c-3p	Lat2	0	1	0	1
rno-miR-34c-3p	Lct	0	1	0	1
rno-miR-34c-3p	Leng8	0	1	0	1
rno-miR-34c-3p	Lhfpl5	0	1	0	1
rno-miR-34c-3p	Lix1_predicted	0	1	0	1
rno-miR-34c-3p	Llgl1	0	1	0	1
rno-miR-34c-3p	Lmtk2	0	1	0	1
rno-miR-34c-3p	LOC10035986	0	0	1	1
rno-miR-34c-3p	LOC10036021	0	0	1	1
rno-miR-34c-3p	LOC10036282	0	0	1	1
rno-miR-34c-3p	LOC246120	0	1	0	1
rno-miR-34c-3p	LOC246295	0	1	0	1
rno-miR-34c-3p	LOC290396	0	1	0	1
rno-miR-34c-3p	LOC291840	0	1	0	1
rno-miR-34c-3p	LOC296235	0	1	0	1
rno-miR-34c-3p	LOC297530	0	1	0	1
rno-miR-34c-3p	LOC298062	0	1	0	1
rno-miR-34c-3p	LOC300024	0	1	0	1
rno-miR-34c-3p	LOC301334	0	1	0	1
rno-miR-34c-3p	LOC301893	0	1	0	1
rno-miR-34c-3p	LOC303215	0	1	0	1
rno-miR-34c-3p	LOC303448	1	0	0	1
rno-miR-34c-3p	LOC305166	0	1	0	1
rno-miR-34c-3p	LOC306792	0	1	0	1
rno-miR-34c-3p	LOC308240	0	1	0	1
rno-miR-34c-3p	LOC308954	1	0	0	1
rno-miR-34c-3p	LOC308990	1	0	0	1
rno-miR-34c-3p	LOC316457	0	1	0	1
rno-miR-34c-3p	LOC361377	0	1	0	1
rno-miR-34c-3p	LOC362154	0	1	0	1
rno-miR-34c-3p	LOC362526	0	1	0	1
rno-miR-34c-3p	LOC363458	0	1	0	1
rno-miR-34c-3p	LOC364773	0	1	0	1
rno-miR-34c-3p	LOC366093	0	1	0	1
rno-miR-34c-3p	LOC367153	0	1	0	1

rno-miR-34c-3p	LOC367398	0	1	0	1
rno-miR-34c-3p	LOC493865	0	1	0	1
rno-miR-34c-3p	LOC497859	0	1	0	1
rno-miR-34c-3p	LOC498749	0	1	0	1
rno-miR-34c-3p	LOC499300	0	1	0	1
rno-miR-34c-3p	LOC500378	0	1	0	1
rno-miR-34c-3p	LOC500476	0	1	0	1
rno-miR-34c-3p	LOC500598	0	1	0	1
rno-miR-34c-3p	LOC501110	1	0	0	1
rno-miR-34c-3p	LOC502908	0	1	0	1
rno-miR-34c-3p	LOC678760	0	1	0	1
rno-miR-34c-3p	LOC678973	0	1	0	1
rno-miR-34c-3p	LOC679158	0	1	0	1
rno-miR-34c-3p	LOC679469	0	1	0	1
rno-miR-34c-3p	LOC679478	0	1	0	1
rno-miR-34c-3p	LOC679774	0	1	0	1
rno-miR-34c-3p	LOC679802	0	1	0	1
rno-miR-34c-3p	LOC679906	0	1	0	1
rno-miR-34c-3p	LOC680104	0	1	0	1
rno-miR-34c-3p	LOC680214	0	1	0	1
rno-miR-34c-3p	LOC680683	0	1	0	1
rno-miR-34c-3p	LOC681067	0	1	0	1
rno-miR-34c-3p	LOC681221	0	1	0	1
rno-miR-34c-3p	LOC681338	0	1	0	1
rno-miR-34c-3p	LOC681499	0	1	0	1
rno-miR-34c-3p	LOC681501	0	1	0	1
rno-miR-34c-3p	LOC681715	0	1	0	1
rno-miR-34c-3p	LOC682070	0	1	0	1
rno-miR-34c-3p	LOC682072	0	1	0	1
rno-miR-34c-3p	LOC683259	0	1	0	1
rno-miR-34c-3p	LOC683501	0	1	0	1
rno-miR-34c-3p	LOC683844	0	0	1	1
rno-miR-34c-3p	LOC683863	0	1	0	1
rno-miR-34c-3p	LOC683968	0	1	0	1
rno-miR-34c-3p	LOC684096	0	1	0	1
rno-miR-34c-3p	LOC684202	0	1	0	1
rno-miR-34c-3p	LOC684322	0	1	0	1
rno-miR-34c-3p	LOC684447	0	1	0	1
rno-miR-34c-3p	LOC685226	0	1	0	1
rno-miR-34c-3p	LOC685233	0	1	0	1
rno-miR-34c-3p	LOC685552	0	1	0	1
rno-miR-34c-3p	LOC685580	0	1	0	1
rno-miR-34c-3p	LOC686212	0	1	0	1
rno-miR-34c-3p	LOC686213	0	1	0	1
rno-miR-34c-3p	LOC686457	0	1	0	1
rno-miR-34c-3p	LOC686480	0	1	0	1
rno-miR-34c-3p	LOC687029	0	0	1	1
rno-miR-34c-3p	LOC687236	0	1	0	1
rno-miR-34c-3p	LOC687719	0	1	0	1
rno-miR-34c-3p	LOC688209	0	1	0	1
rno-miR-34c-3p	LOC688691	0	1	0	1

rno-miR-34c-3p	LOC689232	0	1	0	1
rno-miR-34c-3p	LOC689373	0	1	0	1
rno-miR-34c-3p	LOC689414	0	1	0	1
rno-miR-34c-3p	LOC689460	0	1	0	1
rno-miR-34c-3p	LOC690507	0	1	0	1
rno-miR-34c-3p	LOC690593	0	1	0	1
rno-miR-34c-3p	LOC691030	0	1	0	1
rno-miR-34c-3p	LOC691167	0	1	0	1
rno-miR-34c-3p	LOC691515	0	1	0	1
rno-miR-34c-3p	Lphn1	0	1	0	1
rno-miR-34c-3p	Lpin1	0	1	0	1
rno-miR-34c-3p	Lrig1_predicte	0	1	0	1
rno-miR-34c-3p	Lrrc42	0	1	0	1
rno-miR-34c-3p	Ly6a_predicte	0	1	0	1
rno-miR-34c-3p	Ly6c	0	1	0	1
rno-miR-34c-3p	Maff_predicted	0	1	0	1
rno-miR-34c-3p	Magi2	0	1	0	1
rno-miR-34c-3p	Map3k2	0	1	0	1
rno-miR-34c-3p	Map3k5	0	1	0	1
rno-miR-34c-3p	Map3k8	1	0	0	1
rno-miR-34c-3p	Mbl1	1	0	0	1
rno-miR-34c-3p	Mds024	0	1	0	1
rno-miR-34c-3p	Med8	0	0	1	1
rno-miR-34c-3p	Med8_predicte	0	1	0	1
rno-miR-34c-3p	Mepe	0	1	0	1
rno-miR-34c-3p	Mesdc1	1	0	0	1
rno-miR-34c-3p	MGC105560	1	0	0	1
rno-miR-34c-3p	MGC109046	0	1	0	1
rno-miR-34c-3p	MGC114499	0	1	0	1
rno-miR-34c-3p	MGC125015	0	1	0	1
rno-miR-34c-3p	MGC72612	1	0	0	1
rno-miR-34c-3p	Mgea5	0	1	0	1
rno-miR-34c-3p	Mocs2	1	0	0	1
rno-miR-34c-3p	Mrap_predicte	0	1	0	1
rno-miR-34c-3p	Mrfap1	1	0	0	1
rno-miR-34c-3p	Mrgprg	0	1	0	1
rno-miR-34c-3p	Mrpl16	0	1	0	1
rno-miR-34c-3p	Mrpl40	1	0	0	1
rno-miR-34c-3p	Mrpl47	1	0	0	1
rno-miR-34c-3p	Mrps30_predic	0	1	0	1
rno-miR-34c-3p	Mrps31_predic	0	1	0	1
rno-miR-34c-3p	Ms4a11_predi	0	1	0	1
rno-miR-34c-3p	Ms4a1_predict	0	1	0	1
rno-miR-34c-3p	Msc_predicted	0	1	0	1
rno-miR-34c-3p	Mtf2	0	1	0	1
rno-miR-34c-3p	Mtm1	0	1	0	1
rno-miR-34c-3p	Mtpn	1	0	0	1
rno-miR-34c-3p	Mug1	0	1	0	1
rno-miR-34c-3p	Mx1	1	0	0	1
rno-miR-34c-3p	Myd116	0	1	0	1
rno-miR-34c-3p	Myh10	1	0	0	1

rno-miR-34c-3p	Myo1d	0	1	0	1
rno-miR-34c-3p	Myt1l	0	1	0	1
rno-miR-34c-3p	Naglt1	0	1	0	1
rno-miR-34c-3p	Nanp	1	0	0	1
rno-miR-34c-3p	Nat1	1	0	0	1
rno-miR-34c-3p	Ncam2	1	0	0	1
rno-miR-34c-3p	Ncan	1	0	0	1
rno-miR-34c-3p	Nckap1	0	0	1	1
rno-miR-34c-3p	Ncoa4	1	0	0	1
rno-miR-34c-3p	Ndufa10	1	0	0	1
rno-miR-34c-3p	Ndufaf1_predic	0	1	0	1
rno-miR-34c-3p	Ndufs5b	0	1	0	1
rno-miR-34c-3p	Nedd9	1	0	0	1
rno-miR-34c-3p	Nek4	0	1	0	1
rno-miR-34c-3p	Nf2	0	1	0	1
rno-miR-34c-3p	Nfatc1	0	1	0	1
rno-miR-34c-3p	Ngep	0	1	0	1
rno-miR-34c-3p	Nkx2-4_predic	0	1	0	1
rno-miR-34c-3p	Nkx3-1	1	0	0	1
rno-miR-34c-3p	Nkx6-1	1	0	0	1
rno-miR-34c-3p	Notch4	1	0	0	1
rno-miR-34c-3p	NP_00101477	0	1	0	1
rno-miR-34c-3p	NP_00107361	0	1	0	1
rno-miR-34c-3p	NP_620211.2	0	1	0	1
rno-miR-34c-3p	Npl	1	0	0	1
rno-miR-34c-3p	Nppa	0	1	0	1
rno-miR-34c-3p	Nptn	1	0	0	1
rno-miR-34c-3p	Npy5r	1	0	0	1
rno-miR-34c-3p	Ntng1	0	0	1	1
rno-miR-34c-3p	null	0	1	0	1
rno-miR-34c-3p	Nupl1	1	0	0	1
rno-miR-34c-3p	Nxf1	1	0	0	1
rno-miR-34c-3p	Nxnl2	0	0	1	1
rno-miR-34c-3p	O35770_RAT	0	1	0	1
rno-miR-34c-3p	Ociad1	0	1	0	1
rno-miR-34c-3p	Ogfr	0	1	0	1
rno-miR-34c-3p	Olfm3	1	0	0	1
rno-miR-34c-3p	Olr1	1	0	0	1
rno-miR-34c-3p	Olr1068_predi	0	1	0	1
rno-miR-34c-3p	Olr1069_predi	0	1	0	1
rno-miR-34c-3p	Olr1075_predi	0	1	0	1
rno-miR-34c-3p	Olr1076_predi	0	1	0	1
rno-miR-34c-3p	Olr12_predicte	0	1	0	1
rno-miR-34c-3p	Olr1401_predi	0	1	0	1
rno-miR-34c-3p	Olr1406_predi	0	1	0	1
rno-miR-34c-3p	Olr141_predict	0	1	0	1
rno-miR-34c-3p	Olr1425_predi	0	1	0	1
rno-miR-34c-3p	Olr1538_predi	0	1	0	1
rno-miR-34c-3p	Olr1590_predi	0	1	0	1
rno-miR-34c-3p	Olr1611_predi	0	1	0	1
rno-miR-34c-3p	Olr1617_predi	0	1	0	1

rno-miR-34c-3p	Olr1641_predi	0	1	0	1
rno-miR-34c-3p	Olr1657_predi	0	1	0	1
rno-miR-34c-3p	Olr188_predict	0	1	0	1
rno-miR-34c-3p	Olr194_predict	0	1	0	1
rno-miR-34c-3p	Olr233_predict	0	1	0	1
rno-miR-34c-3p	Olr281_predict	0	1	0	1
rno-miR-34c-3p	Olr35	0	1	0	1
rno-miR-34c-3p	Olr354_predict	0	1	0	1
rno-miR-34c-3p	Olr409_predict	0	1	0	1
rno-miR-34c-3p	Olr462_predict	0	1	0	1
rno-miR-34c-3p	Olr466_predict	0	1	0	1
rno-miR-34c-3p	Olr507_predict	0	1	0	1
rno-miR-34c-3p	Olr533_predict	0	1	0	1
rno-miR-34c-3p	Olr596_predict	0	1	0	1
rno-miR-34c-3p	Olr623_predict	0	1	0	1
rno-miR-34c-3p	Olr690_predict	0	1	0	1
rno-miR-34c-3p	Olr717_predict	0	1	0	1
rno-miR-34c-3p	Olr721_predict	0	1	0	1
rno-miR-34c-3p	Olr79	0	1	0	1
rno-miR-34c-3p	Olr85_predicte	0	1	0	1
rno-miR-34c-3p	Omd	0	1	0	1
rno-miR-34c-3p	Opcml	1	0	0	1
rno-miR-34c-3p	Oprk1	1	0	0	1
rno-miR-34c-3p	Oprm1	1	0	0	1
rno-miR-34c-3p	Osbpl6_predic	0	1	0	1
rno-miR-34c-3p	P2ry1	1	0	0	1
rno-miR-34c-3p	Pacsin2	1	0	0	1
rno-miR-34c-3p	Pafah1b1	0	0	1	1
rno-miR-34c-3p	Pak1ip1	1	0	0	1
rno-miR-34c-3p	Pcdhb14_pred	0	1	0	1
rno-miR-34c-3p	Pcdhgb4	0	1	0	1
rno-miR-34c-3p	Pcid2	0	1	0	1
rno-miR-34c-3p	Pcm1	0	1	0	1
rno-miR-34c-3p	Pctp	1	0	0	1
rno-miR-34c-3p	Pcyox1	1	0	0	1
rno-miR-34c-3p	Pdha1	1	0	0	1
rno-miR-34c-3p	Pdzk3	0	1	0	1
rno-miR-34c-3p	Pdzrn3_predic	0	1	0	1
rno-miR-34c-3p	Pex3	1	0	0	1
rno-miR-34c-3p	Pfkfb2	1	0	0	1
rno-miR-34c-3p	Phlpp1	1	0	0	1
rno-miR-34c-3p	Pi15_predicted	0	1	0	1
rno-miR-34c-3p	Pi4k2b	1	0	0	1
rno-miR-34c-3p	Pigp_predicted	0	1	0	1
rno-miR-34c-3p	Pik3r4_predict	0	1	0	1
rno-miR-34c-3p	Pip5k1a	0	1	0	1
rno-miR-34c-3p	Pkp3_predicte	0	1	0	1
rno-miR-34c-3p	Plat	1	0	0	1
rno-miR-34c-3p	Plcg2	0	1	0	1
rno-miR-34c-3p	Plcl1	1	0	0	1
rno-miR-34c-3p	Plekha3	0	1	0	1

rno-miR-34c-3p	Plpi	0	1	0	1
rno-miR-34c-3p	Plrg1	0	1	0	1
rno-miR-34c-3p	Pltp_predicted	0	1	0	1
rno-miR-34c-3p	Pola1	0	1	0	1
rno-miR-34c-3p	Polb	1	0	0	1
rno-miR-34c-3p	Pot1	1	0	0	1
rno-miR-34c-3p	Pou2af1	0	0	1	1
rno-miR-34c-3p	Ppl_predicted	0	1	0	1
rno-miR-34c-3p	Ppp1cc	1	0	0	1
rno-miR-34c-3p	Ppp1r15a	1	0	0	1
rno-miR-34c-3p	Ppp1r15b_pre	0	1	0	1
rno-miR-34c-3p	Ppp1r2	1	0	0	1
rno-miR-34c-3p	Ppp3ca	0	1	0	1
rno-miR-34c-3p	Ppp3cc	0	1	0	1
rno-miR-34c-3p	Pprf18	0	1	0	1
rno-miR-34c-3p	PQLC1_RAT	0	1	0	1
rno-miR-34c-3p	Prdm10	0	1	0	1
rno-miR-34c-3p	Prdx2	0	1	0	1
rno-miR-34c-3p	Prdx6	1	0	0	1
rno-miR-34c-3p	Prepl	1	0	0	1
rno-miR-34c-3p	Prickle1	0	1	0	1
rno-miR-34c-3p	Prkcd	0	1	0	1
rno-miR-34c-3p	Prkwnk1	0	1	0	1
rno-miR-34c-3p	Prl3c1	1	0	0	1
rno-miR-34c-3p	Prlr	0	1	0	1
rno-miR-34c-3p	Prpf4b	1	0	0	1
rno-miR-34c-3p	Prss34_predic	0	1	0	1
rno-miR-34c-3p	Psma1	0	1	0	1
rno-miR-34c-3p	Psmd13_predi	0	1	0	1
rno-miR-34c-3p	Pter	1	0	0	1
rno-miR-34c-3p	Ptk7	0	1	0	1
rno-miR-34c-3p	Ptplad2_predic	0	1	0	1
rno-miR-34c-3p	Ptpn14_predic	0	1	0	1
rno-miR-34c-3p	Purg	0	1	0	1
rno-miR-34c-3p	Pus1	0	1	0	1
rno-miR-34c-3p	Q32ZG4_RAT	0	1	0	1
rno-miR-34c-3p	Q3KRE5_RAT	0	1	0	1
rno-miR-34c-3p	Q4FZT7_RAT	0	1	0	1
rno-miR-34c-3p	Q5FVP9_RAT	0	1	0	1
rno-miR-34c-3p	Q5M928_RAT	0	1	0	1
rno-miR-34c-3p	Q5XHY1_RAT	0	1	0	1
rno-miR-34c-3p	Q64548-2	0	1	0	1
rno-miR-34c-3p	Q6AYP8_RAT	0	1	0	1
rno-miR-34c-3p	Q6TXI9_RAT	0	1	0	1
rno-miR-34c-3p	Q7TMB2_RAT	0	1	0	1
rno-miR-34c-3p	Q7TP73_RAT	0	1	0	1
rno-miR-34c-3p	Q8R4H5_RAT	0	1	0	1
rno-miR-34c-3p	Q91Y79_RAT	0	1	0	1
rno-miR-34c-3p	Q9JKC9-3	0	1	0	1
rno-miR-34c-3p	Rab1	1	0	0	1
rno-miR-34c-3p	Rab18	1	0	0	1

rno-miR-34c-3p	Rab2l	0	1	0	1
rno-miR-34c-3p	Rab3ip	0	1	0	1
rno-miR-34c-3p	Rab6ip2	0	1	0	1
rno-miR-34c-3p	Rad18	0	1	0	1
rno-miR-34c-3p	Rai14	1	0	0	1
rno-miR-34c-3p	Ramp1	0	1	0	1
rno-miR-34c-3p	Rap1b	1	0	0	1
rno-miR-34c-3p	Rassf3_predic	0	1	0	1
rno-miR-34c-3p	Rbks_predicte	0	1	0	1
rno-miR-34c-3p	Rbm16	1	0	0	1
rno-miR-34c-3p	Rbm24_predic	0	1	0	1
rno-miR-34c-3p	Rcan2	1	0	0	1
rno-miR-34c-3p	Rcn1_predicte	0	1	0	1
rno-miR-34c-3p	Rcor2	0	1	0	1
rno-miR-34c-3p	Rdh10	0	1	0	1
rno-miR-34c-3p	Rdx	0	1	0	1
rno-miR-34c-3p	Recc1	0	1	0	1
rno-miR-34c-3p	Rgag4	0	1	0	1
rno-miR-34c-3p	RGD1303127	1	0	0	1
rno-miR-34c-3p	RGD1304587	0	1	0	1
rno-miR-34c-3p	RGD1304704	0	1	0	1
rno-miR-34c-3p	RGD1304906_	0	1	0	1
rno-miR-34c-3p	RGD1305215	1	0	0	1
rno-miR-34c-3p	RGD1305350	0	0	1	1
rno-miR-34c-3p	RGD1305350_	0	1	0	1
rno-miR-34c-3p	RGD1305356	0	1	0	1
rno-miR-34c-3p	RGD1305455	0	1	0	1
rno-miR-34c-3p	RGD1305527_	0	1	0	1
rno-miR-34c-3p	RGD1305939	0	1	0	1
rno-miR-34c-3p	RGD1305961_	0	1	0	1
rno-miR-34c-3p	RGD1306423_	0	1	0	1
rno-miR-34c-3p	RGD1306519_	0	1	0	1
rno-miR-34c-3p	RGD1306568	0	1	0	1
rno-miR-34c-3p	RGD1306697_	0	1	0	1
rno-miR-34c-3p	RGD1306801_	0	1	0	1
rno-miR-34c-3p	RGD1306820_	0	1	0	1
rno-miR-34c-3p	RGD1307202	0	1	0	1
rno-miR-34c-3p	RGD1307704_	0	1	0	1
rno-miR-34c-3p	RGD1307749_	0	1	0	1
rno-miR-34c-3p	RGD1307832_	0	1	0	1
rno-miR-34c-3p	RGD1307890	1	0	0	1
rno-miR-34c-3p	RGD1308084	0	1	0	1
rno-miR-34c-3p	RGD1308317_	0	1	0	1
rno-miR-34c-3p	RGD1308430_	0	1	0	1
rno-miR-34c-3p	RGD1308470	1	0	0	1
rno-miR-34c-3p	RGD1308632	0	1	0	1
rno-miR-34c-3p	RGD1308773_	0	1	0	1
rno-miR-34c-3p	RGD1308782	0	1	0	1
rno-miR-34c-3p	RGD1309020	0	1	0	1
rno-miR-34c-3p	RGD1309362	1	0	0	1
rno-miR-34c-3p	RGD1309708	0	1	0	1

rno-miR-34c-3p	RGD1309765	0	0	1	1
rno-miR-34c-3p	RGD1309783_	0	1	0	1
rno-miR-34c-3p	RGD1309873_	0	1	0	1
rno-miR-34c-3p	RGD1309892	0	1	0	1
rno-miR-34c-3p	RGD1310137	0	1	0	1
rno-miR-34c-3p	RGD1310139_	0	1	0	1
rno-miR-34c-3p	RGD1310143	0	1	0	1
rno-miR-34c-3p	RGD1310316	0	1	0	1
rno-miR-34c-3p	RGD1310670_	0	1	0	1
rno-miR-34c-3p	RGD1311045_	0	1	0	1
rno-miR-34c-3p	RGD1311123	0	1	0	1
rno-miR-34c-3p	RGD1311283_	0	1	0	1
rno-miR-34c-3p	RGD1311547	0	1	0	1
rno-miR-34c-3p	RGD1312038_	0	1	0	1
rno-miR-34c-3p	RGD1359529	1	0	0	1
rno-miR-34c-3p	RGD1559527_	0	1	0	1
rno-miR-34c-3p	RGD1559531_	0	1	0	1
rno-miR-34c-3p	RGD1559904_	0	1	0	1
rno-miR-34c-3p	RGD1560224_	0	1	0	1
rno-miR-34c-3p	RGD1560394_	0	1	0	1
rno-miR-34c-3p	RGD1560456_	0	1	0	1
rno-miR-34c-3p	RGD1560459_	0	1	0	1
rno-miR-34c-3p	RGD1560496_	0	1	0	1
rno-miR-34c-3p	RGD1560636_	0	1	0	1
rno-miR-34c-3p	RGD1560707_	0	1	0	1
rno-miR-34c-3p	RGD1560717_	0	1	0	1
rno-miR-34c-3p	RGD1560948_	0	1	0	1
rno-miR-34c-3p	RGD1560953_	0	1	0	1
rno-miR-34c-3p	RGD1560978_	0	1	0	1
rno-miR-34c-3p	RGD1561023_	0	1	0	1
rno-miR-34c-3p	RGD1561196_	0	1	0	1
rno-miR-34c-3p	RGD1561833_	0	1	0	1
rno-miR-34c-3p	RGD1561873_	0	1	0	1
rno-miR-34c-3p	RGD1561940	0	0	1	1
rno-miR-34c-3p	RGD1561958_	0	1	0	1
rno-miR-34c-3p	RGD1562084_	0	1	0	1
rno-miR-34c-3p	RGD1562140_	0	1	0	1
rno-miR-34c-3p	RGD1562214_	0	1	0	1
rno-miR-34c-3p	RGD1562246_	0	1	0	1
rno-miR-34c-3p	RGD1562284_	0	1	0	1
rno-miR-34c-3p	RGD1562356_	0	1	0	1
rno-miR-34c-3p	RGD1562417_	0	1	0	1
rno-miR-34c-3p	RGD1562494_	0	1	0	1
rno-miR-34c-3p	RGD1562548_	0	1	0	1
rno-miR-34c-3p	RGD1563046_	0	1	0	1
rno-miR-34c-3p	RGD1563142_	0	1	0	1
rno-miR-34c-3p	RGD1563383_	0	1	0	1
rno-miR-34c-3p	RGD1563465_	0	1	0	1
rno-miR-34c-3p	RGD1563468_	0	1	0	1
rno-miR-34c-3p	RGD1563625_	0	1	0	1
rno-miR-34c-3p	RGD1564002_	0	1	0	1

rno-miR-34c-3p	RGD1564125_	0	1	0	1
rno-miR-34c-3p	RGD1564172_	0	1	0	1
rno-miR-34c-3p	RGD1564178_	0	1	0	1
rno-miR-34c-3p	RGD1564444_	0	1	0	1
rno-miR-34c-3p	RGD1564638_	0	1	0	1
rno-miR-34c-3p	RGD1564706_	0	1	0	1
rno-miR-34c-3p	RGD1564904_	0	1	0	1
rno-miR-34c-3p	RGD1564936_	0	1	0	1
rno-miR-34c-3p	RGD1565018_	0	1	0	1
rno-miR-34c-3p	RGD1565031_	0	1	0	1
rno-miR-34c-3p	RGD1565055_	0	1	0	1
rno-miR-34c-3p	RGD1565152_	0	1	0	1
rno-miR-34c-3p	RGD1565212_	0	1	0	1
rno-miR-34c-3p	RGD1565245_	0	1	0	1
rno-miR-34c-3p	RGD1565419_	0	1	0	1
rno-miR-34c-3p	RGD1565498_	0	1	0	1
rno-miR-34c-3p	RGD1565560_	0	1	0	1
rno-miR-34c-3p	RGD1566224_	0	1	0	1
rno-miR-34c-3p	RGD1566225_	0	1	0	1
rno-miR-34c-3p	RGD1566252_	0	1	0	1
rno-miR-34c-3p	RL12_RAT	0	1	0	1
rno-miR-34c-3p	Rlim	0	0	1	1
rno-miR-34c-3p	Rnd3	1	0	0	1
rno-miR-34c-3p	Rnf11	0	0	1	1
rno-miR-34c-3p	Rnmt	1	0	0	1
rno-miR-34c-3p	Rock2	0	1	0	1
rno-miR-34c-3p	Rpl11	0	1	0	1
rno-miR-34c-3p	Rpl8	1	0	0	1
rno-miR-34c-3p	Rps18	0	1	0	1
rno-miR-34c-3p	Rps21	1	0	0	1
rno-miR-34c-3p	Rps27	0	1	0	1
rno-miR-34c-3p	Rps27a	0	1	0	1
rno-miR-34c-3p	Rps6ka5	0	1	0	1
rno-miR-34c-3p	Rreb1_predicte	0	1	0	1
rno-miR-34c-3p	Rsrc2	1	0	0	1
rno-miR-34c-3p	RT1-Ke4	0	1	0	1
rno-miR-34c-3p	RT1-M6-2	0	1	0	1
rno-miR-34c-3p	RT1-S3	0	1	0	1
rno-miR-34c-3p	Rtn3	1	0	0	1
rno-miR-34c-3p	Rtn4rl1	0	1	0	1
rno-miR-34c-3p	S100a4	0	1	0	1
rno-miR-34c-3p	S1pr5	1	0	0	1
rno-miR-34c-3p	Sall2_predicte	0	1	0	1
rno-miR-34c-3p	Samhd1_predi	0	1	0	1
rno-miR-34c-3p	Sat1	1	0	0	1
rno-miR-34c-3p	Scamp1	0	0	1	1
rno-miR-34c-3p	Sccpdh	0	1	0	1
rno-miR-34c-3p	Scn2a1	1	0	0	1
rno-miR-34c-3p	Sdccag10	0	1	0	1
rno-miR-34c-3p	Sdha	1	0	0	1
rno-miR-34c-3p	Sdpr	1	0	0	1

rno-miR-34c-3p	Sec61a1	1	0	0	1
rno-miR-34c-3p	Secisbp2	1	0	0	1
rno-miR-34c-3p	Set	1	0	0	1
rno-miR-34c-3p	Sfrs15	0	1	0	1
rno-miR-34c-3p	Sfrs2	1	0	0	1
rno-miR-34c-3p	Sfrs5	1	0	0	1
rno-miR-34c-3p	Sft2d2	1	0	0	1
rno-miR-34c-3p	Sft2d3	0	0	1	1
rno-miR-34c-3p	Sgk2	0	1	0	1
rno-miR-34c-3p	Sigmar1	1	0	0	1
rno-miR-34c-3p	Sil1	0	1	0	1
rno-miR-34c-3p	Sirt5	0	1	0	1
rno-miR-34c-3p	Slc10a2	1	0	0	1
rno-miR-34c-3p	Slc12a7	0	1	0	1
rno-miR-34c-3p	Slc23a1	1	0	0	1
rno-miR-34c-3p	Slc23a2	0	1	0	1
rno-miR-34c-3p	Slc24a2	1	0	0	1
rno-miR-34c-3p	Slc25a27	1	0	0	1
rno-miR-34c-3p	Slc33a1	1	0	0	1
rno-miR-34c-3p	Slc35a1	0	0	1	1
rno-miR-34c-3p	Slc39a7	1	0	0	1
rno-miR-34c-3p	Slc6a15	1	0	0	1
rno-miR-34c-3p	Slfn3	1	0	0	1
rno-miR-34c-3p	Smarca2	1	0	0	1
rno-miR-34c-3p	Smc5l1_predic	0	1	0	1
rno-miR-34c-3p	Snd1	0	1	0	1
rno-miR-34c-3p	Snrpa	0	1	0	1
rno-miR-34c-3p	Snx1	1	0	0	1
rno-miR-34c-3p	Snx15	1	0	0	1
rno-miR-34c-3p	Snx16	1	0	0	1
rno-miR-34c-3p	Snx24	1	0	0	1
rno-miR-34c-3p	Soat1	0	1	0	1
rno-miR-34c-3p	Sorbs2	1	0	0	1
rno-miR-34c-3p	Sorl1_predicte	0	1	0	1
rno-miR-34c-3p	Sox8_predicte	0	1	0	1
rno-miR-34c-3p	Spa17	0	1	0	1
rno-miR-34c-3p	Spast_predicte	0	1	0	1
rno-miR-34c-3p	Spint2	1	0	0	1
rno-miR-34c-3p	Srgn	1	0	0	1
rno-miR-34c-3p	Srp54a	1	0	0	1
rno-miR-34c-3p	St18	1	0	0	1
rno-miR-34c-3p	Stat5b	0	1	0	1
rno-miR-34c-3p	Statip1	0	1	0	1
rno-miR-34c-3p	Stch	0	1	0	1
rno-miR-34c-3p	Stim2_predicte	0	1	0	1
rno-miR-34c-3p	Stoml3_predic	0	1	0	1
rno-miR-34c-3p	Strn3	1	0	0	1
rno-miR-34c-3p	Sts	0	1	0	1
rno-miR-34c-3p	Stx12	1	0	0	1
rno-miR-34c-3p	Stx5a	0	1	0	1
rno-miR-34c-3p	Sufu	0	1	0	1

rno-miR-34c-3p	Sult1c2	1	0	0	1
rno-miR-34c-3p	Sv2b	1	0	0	1
rno-miR-34c-3p	Syt1	1	0	0	1
rno-miR-34c-3p	Syt6	0	1	0	1
rno-miR-34c-3p	Tacc2	0	1	0	1
rno-miR-34c-3p	Tanc1	0	1	0	1
rno-miR-34c-3p	Tbx19_predict	0	1	0	1
rno-miR-34c-3p	Tcea1	1	0	0	1
rno-miR-34c-3p	Tcfdp2_predic	0	1	0	1
rno-miR-34c-3p	Tcirg1	1	0	0	1
rno-miR-34c-3p	Tdp1	0	1	0	1
rno-miR-34c-3p	TECT2_RAT	0	1	0	1
rno-miR-34c-3p	Tex14_predict	0	1	0	1
rno-miR-34c-3p	Tfb2m	1	0	0	1
rno-miR-34c-3p	tGap1	1	0	0	1
rno-miR-34c-3p	Tgfb2	1	0	0	1
rno-miR-34c-3p	Tgfbr1	1	0	0	1
rno-miR-34c-3p	Tgoln1	1	0	0	1
rno-miR-34c-3p	Tigd2	0	1	0	1
rno-miR-34c-3p	Timm17a	1	0	0	1
rno-miR-34c-3p	Tle1_predicted	0	1	0	1
rno-miR-34c-3p	Tmc1_predicte	0	1	0	1
rno-miR-34c-3p	Tmem106c	1	0	0	1
rno-miR-34c-3p	Tmem16h	0	1	0	1
rno-miR-34c-3p	Tmem183a	1	0	0	1
rno-miR-34c-3p	Tmem41b	1	0	0	1
rno-miR-34c-3p	Tmem43	1	0	0	1
rno-miR-34c-3p	Tmem47	0	0	1	1
rno-miR-34c-3p	Tmem77	1	0	0	1
rno-miR-34c-3p	Tmpo	1	0	0	1
rno-miR-34c-3p	Tnip2	1	0	0	1
rno-miR-34c-3p	Tpp2	1	0	0	1
rno-miR-34c-3p	Tradd	0	1	0	1
rno-miR-34c-3p	TRAV2	0	1	0	1
rno-miR-34c-3p	Trhr2	0	1	0	1
rno-miR-34c-3p	Trim34_predic	0	1	0	1
rno-miR-34c-3p	Trim40	0	1	0	1
rno-miR-34c-3p	Trmu_predicte	0	1	0	1
rno-miR-34c-3p	Trpm8	1	0	0	1
rno-miR-34c-3p	Trps1_predicte	0	1	0	1
rno-miR-34c-3p	Tsc22d3	1	0	0	1
rno-miR-34c-3p	Tsn	0	1	0	1
rno-miR-34c-3p	Tsnax	1	0	0	1
rno-miR-34c-3p	Tssc1	0	1	0	1
rno-miR-34c-3p	Ttll7	0	1	0	1
rno-miR-34c-3p	Ttrap	1	0	0	1
rno-miR-34c-3p	Tuba6	0	1	0	1
rno-miR-34c-3p	Tubgcp5_pred	0	1	0	1
rno-miR-34c-3p	Twistnb_predi	0	1	0	1
rno-miR-34c-3p	Txndc11_pred	0	1	0	1
rno-miR-34c-3p	Tyr	0	1	0	1

rno-miR-34c-3p	Ube2d3	1	0	0	1
rno-miR-34c-3p	Ube2v2	1	0	0	1
rno-miR-34c-3p	Ube2z	0	1	0	1
rno-miR-34c-3p	Ubfd1	1	0	0	1
rno-miR-34c-3p	Ubiad1_predic	0	1	0	1
rno-miR-34c-3p	Ubl4	0	0	1	1
rno-miR-34c-3p	Ubl4a_predicte	0	1	0	1
rno-miR-34c-3p	Uchl5	1	0	0	1
rno-miR-34c-3p	Ugdh	0	1	0	1
rno-miR-34c-3p	Unc5d	0	1	0	1
rno-miR-34c-3p	Usp1	1	0	0	1
rno-miR-34c-3p	Usp9x_predict	0	1	0	1
rno-miR-34c-3p	Vamp1	1	0	0	1
rno-miR-34c-3p	Vcp	1	0	0	1
rno-miR-34c-3p	Vgll2_predicte	0	1	0	1
rno-miR-34c-3p	Visa	1	0	0	1
rno-miR-34c-3p	Vwa5a	1	0	0	1
rno-miR-34c-3p	Vwf	0	1	0	1
rno-miR-34c-3p	Wdr20	0	1	0	1
rno-miR-34c-3p	Wdr27_predict	0	1	0	1
rno-miR-34c-3p	Wdr31	0	1	0	1
rno-miR-34c-3p	Wdr44	0	1	0	1
rno-miR-34c-3p	Wdr46	0	1	0	1
rno-miR-34c-3p	Wdr77	0	1	0	1
rno-miR-34c-3p	Wee1	1	0	0	1
rno-miR-34c-3p	Wipf1	1	0	0	1
rno-miR-34c-3p	Xiap	1	0	0	1
rno-miR-34c-3p	XR_005455.1	0	1	0	1
rno-miR-34c-3p	XR_005456.1	0	1	0	1
rno-miR-34c-3p	Yars2	1	0	0	1
rno-miR-34c-3p	Ybx2_predicte	0	1	0	1
rno-miR-34c-3p	Yeats4_predic	0	1	0	1
rno-miR-34c-3p	Yme1l1	0	1	0	1
rno-miR-34c-3p	Ywhaq	1	0	0	1
rno-miR-34c-3p	Zbtb39_predic	0	1	0	1
rno-miR-34c-3p	Zbtb44	1	0	0	1
rno-miR-34c-3p	Zbtb9	1	0	0	1
rno-miR-34c-3p	Zc3h8	0	1	0	1
rno-miR-34c-3p	Zdhhc13	0	1	0	1
rno-miR-34c-3p	Zfp110	0	1	0	1
rno-miR-34c-3p	Zfp187	0	0	1	1
rno-miR-34c-3p	Zfp2	0	1	0	1
rno-miR-34c-3p	Zfp296	0	1	0	1
rno-miR-34c-3p	Zfp334_predic	0	1	0	1
rno-miR-34c-3p	Zfp339_predic	0	1	0	1
rno-miR-34c-3p	Zfp358_predic	0	1	0	1
rno-miR-34c-3p	Zfp367	1	0	0	1
rno-miR-34c-3p	Zfp423	1	0	0	1
rno-miR-34c-3p	Zfp52	1	0	0	1
rno-miR-34c-3p	Zfp868	0	0	1	1
rno-miR-34c-3p	Zmat2	0	1	0	1

rno-miR-34c-3p	Zmym1_predic	0	1	0	1
rno-miR-34c-3p	Znf511_predic	0	1	0	1
rno-miR-34c-3p	Znf655	1	0	0	1
rno-miR-34c-3p	Zscan22	0	1	0	1
rno-miR-374-3p	Acsl1	0	0	1	1
rno-miR-374-3p	Agl	0	0	1	1
rno-miR-374-3p	Ankrd29	0	0	1	1
rno-miR-374-3p	Arpc1a	0	0	1	1
rno-miR-374-3p	Asb10	0	0	1	1
rno-miR-374-3p	Atad1	0	0	1	1
rno-miR-374-3p	Cbfb	0	0	1	1
rno-miR-374-3p	Cyp2c11	0	0	1	1
rno-miR-374-3p	Dgkb	0	0	1	1
rno-miR-374-3p	Dpysl5	0	0	1	1
rno-miR-374-3p	Dynlt1	0	0	1	1
rno-miR-374-3p	Eif1ax	0	0	1	1
rno-miR-374-3p	Eif4a2	0	0	1	1
rno-miR-374-3p	Fzd6	0	0	1	1
rno-miR-374-3p	Golga7	0	0	1	1
rno-miR-374-3p	Gpr160	0	0	1	1
rno-miR-374-3p	Gpr85	0	0	1	1
rno-miR-374-3p	Grip1	0	0	1	1
rno-miR-374-3p	Hace1	0	0	1	1
rno-miR-374-3p	Has3	0	0	1	1
rno-miR-374-3p	Hmgcr	0	0	1	1
rno-miR-374-3p	Ifi30	0	0	1	1
rno-miR-374-3p	Il17a	0	0	1	1
rno-miR-374-3p	Jazf1	0	0	1	1
rno-miR-374-3p	Kdm2a	0	0	1	1
rno-miR-374-3p	Lgalsl	0	0	1	1
rno-miR-374-3p	LOC679651	0	0	1	1
rno-miR-374-3p	LOC684800	0	0	1	1
rno-miR-374-3p	Lpgat1	0	0	1	1
rno-miR-374-3p	Lrrn1	0	0	1	1
rno-miR-374-3p	Mageh1	0	0	1	1
rno-miR-374-3p	Msl2	0	0	1	1
rno-miR-374-3p	Msx2	0	0	1	1
rno-miR-374-3p	Nfat5	0	0	1	1
rno-miR-374-3p	Nxt2	0	0	1	1
rno-miR-374-3p	Olig2	0	0	1	1
rno-miR-374-3p	Pip4k2a	0	0	1	1
rno-miR-374-3p	Pld3	0	0	1	1
rno-miR-374-3p	Plekha8	0	0	1	1
rno-miR-374-3p	Prpf38a	0	0	1	1
rno-miR-374-3p	Rab21	0	0	1	1
rno-miR-374-3p	Rab9a	0	0	1	1
rno-miR-374-3p	Rad17	0	0	1	1
rno-miR-374-3p	Rasgrp1	0	0	1	1
rno-miR-374-3p	Rbm8a	0	0	1	1
rno-miR-374-3p	Ret	0	0	1	1
rno-miR-374-3p	RGD1305721	0	0	1	1

rno-miR-374-3p	RGD1308874	0	0	1	1
rno-miR-374-3p	RGD1359108	0	0	1	1
rno-miR-374-3p	RGD1563701	0	0	1	1
rno-miR-374-3p	Rnf34	0	0	1	1
rno-miR-374-3p	Rqcd1	0	0	1	1
rno-miR-374-3p	Sec63	0	0	1	1
rno-miR-374-3p	Sgms1	0	0	1	1
rno-miR-374-3p	Sh3kbp1	0	0	1	1
rno-miR-374-3p	Ska1	0	0	1	1
rno-miR-374-3p	Slc18a1	0	0	1	1
rno-miR-374-3p	Slc20a1	0	0	1	1
rno-miR-374-3p	Slc35f5	0	0	1	1
rno-miR-374-3p	Slc6a8	0	0	1	1
rno-miR-374-3p	Smtnl2	0	0	1	1
rno-miR-374-3p	Sparc	0	0	1	1
rno-miR-374-3p	Taf9b	0	0	1	1
rno-miR-374-3p	Tcea1	0	0	1	1
rno-miR-374-3p	Tek	0	0	1	1
rno-miR-374-3p	Tfrc	0	0	1	1
rno-miR-374-3p	Tmem134	0	0	1	1
rno-miR-374-3p	Trib2	0	0	1	1
rno-miR-374-3p	Ublcp1	0	0	1	1
rno-miR-374-3p	Ugt2b1	0	0	1	1
rno-miR-374-3p	Vldlr	0	0	1	1
rno-miR-374-3p	Zfp280d	0	0	1	1
rno-miR-374-3p	Zfp53	0	0	1	1
rno-miR-375-3p	0610010E21R	0	1	0	1
rno-miR-375-3p	1700001P01R	0	1	0	1
rno-miR-375-3p	1700010C24R	0	1	0	1
rno-miR-375-3p	2410187C16R	0	1	0	1
rno-miR-375-3p	2700049A03R	0	1	0	1
rno-miR-375-3p	4930426D05R	0	1	0	1
rno-miR-375-3p	4930485B16R	0	1	0	1
rno-miR-375-3p	4932416N17R	0	1	0	1
rno-miR-375-3p	A230050P20R	0	1	0	1
rno-miR-375-3p	A530088E08R	0	1	0	1
rno-miR-375-3p	Abca9_predict	0	1	0	1
rno-miR-375-3p	Abcb1a	1	0	0	1
rno-miR-375-3p	Abcd3	0	1	0	1
rno-miR-375-3p	Abi3	0	1	0	1
rno-miR-375-3p	Abo	0	1	0	1
rno-miR-375-3p	Ace2	0	1	0	1
rno-miR-375-3p	Acn9	0	1	0	1
rno-miR-375-3p	Acsl3	1	0	0	1
rno-miR-375-3p	Actr10	0	1	0	1
rno-miR-375-3p	Adamts6_pred	0	1	0	1
rno-miR-375-3p	Adap2	1	0	0	1
rno-miR-375-3p	Adcy10	1	0	0	1
rno-miR-375-3p	Adfp	0	1	0	1
rno-miR-375-3p	Adprhl1	0	1	0	1
rno-miR-375-3p	Agmat	0	1	0	1

rno-miR-375-3p	Agrp	0	1	0	1
rno-miR-375-3p	Aifm1	1	0	0	1
rno-miR-375-3p	Aim1l_predicte	0	1	0	1
rno-miR-375-3p	Akap2	0	1	0	1
rno-miR-375-3p	Aldh3b1	0	1	0	1
rno-miR-375-3p	Aldh5a1	0	1	0	1
rno-miR-375-3p	Alg1_predicted	0	1	0	1
rno-miR-375-3p	Amacr	0	1	0	1
rno-miR-375-3p	Ank1	0	1	0	1
rno-miR-375-3p	Anxa2	0	1	0	1
rno-miR-375-3p	Apeh	0	1	0	1
rno-miR-375-3p	Arf4l_predicted	0	1	0	1
rno-miR-375-3p	Arg1	0	1	0	1
rno-miR-375-3p	Arhgap15	1	0	0	1
rno-miR-375-3p	Arhgdig_predic	0	1	0	1
rno-miR-375-3p	Arhgef5	0	1	0	1
rno-miR-375-3p	Arid4b	1	0	0	1
rno-miR-375-3p	Arl13a	1	0	0	1
rno-miR-375-3p	Arl3	0	1	0	1
rno-miR-375-3p	Arl6ip6	1	0	0	1
rno-miR-375-3p	Asah1	1	0	0	1
rno-miR-375-3p	Asahl_predicte	0	1	0	1
rno-miR-375-3p	Asb-4	0	1	0	1
rno-miR-375-3p	Atp1b1	1	0	0	1
rno-miR-375-3p	Atp5i	0	1	0	1
rno-miR-375-3p	Atp6v0d2	0	1	0	1
rno-miR-375-3p	Atp8b1_predic	0	1	0	1
rno-miR-375-3p	Atp8b3_predic	0	1	0	1
rno-miR-375-3p	Atpbd1c	0	1	0	1
rno-miR-375-3p	B3gnt4_predic	0	1	0	1
rno-miR-375-3p	B4galt2_predic	0	1	0	1
rno-miR-375-3p	Bbs7	1	0	0	1
rno-miR-375-3p	Bche	0	1	0	1
rno-miR-375-3p	Bcl2a1	0	1	0	1
rno-miR-375-3p	Bdh1	0	1	0	1
rno-miR-375-3p	Bin1	0	1	0	1
rno-miR-375-3p	Birc2	0	1	0	1
rno-miR-375-3p	Bloc1s1_predic	0	1	0	1
rno-miR-375-3p	Bmp2	0	1	0	1
rno-miR-375-3p	Bola1_predicte	0	1	0	1
rno-miR-375-3p	Bpnt1	0	1	0	1
rno-miR-375-3p	Brd2	0	1	0	1
rno-miR-375-3p	Btbd4_predicte	0	1	0	1
rno-miR-375-3p	Btg3	0	1	0	1
rno-miR-375-3p	Btrc	0	1	0	1
rno-miR-375-3p	Bxdc5	0	1	0	1
rno-miR-375-3p	Bzrap1	0	1	0	1
rno-miR-375-3p	C030046E11R	0	1	0	1
rno-miR-375-3p	C6	0	1	0	1
rno-miR-375-3p	Cacna1g	0	1	0	1
rno-miR-375-3p	Calm1	0	1	0	1

rno-miR-375-3p	Camlg	0	1	0	1
rno-miR-375-3p	Capn2	0	1	0	1
rno-miR-375-3p	Capzb	1	0	0	1
rno-miR-375-3p	Car3	1	0	0	1
rno-miR-375-3p	Casp3	1	0	0	1
rno-miR-375-3p	Cbfa2t2_predic	0	1	0	1
rno-miR-375-3p	Ccdc50	1	0	0	1
rno-miR-375-3p	Ccl2	0	1	0	1
rno-miR-375-3p	Cct6a	0	1	0	1
rno-miR-375-3p	Cd109_predict	0	1	0	1
rno-miR-375-3p	Cd302	0	1	0	1
rno-miR-375-3p	Cd36	1	0	0	1
rno-miR-375-3p	Cd5	0	1	0	1
rno-miR-375-3p	Cd6	0	1	0	1
rno-miR-375-3p	Cd68	1	0	0	1
rno-miR-375-3p	Cdc42bpb	1	0	0	1
rno-miR-375-3p	Cdc7_predicte	0	1	0	1
rno-miR-375-3p	Cdc91l1	0	1	0	1
rno-miR-375-3p	Cdh10	0	1	0	1
rno-miR-375-3p	Cdh22	0	1	0	1
rno-miR-375-3p	Cdk10	0	1	0	1
rno-miR-375-3p	Cdk2	1	0	0	1
rno-miR-375-3p	Cdk7	0	1	0	1
rno-miR-375-3p	Cdkal1_predic	0	1	0	1
rno-miR-375-3p	CDX1_RAT	0	1	0	1
rno-miR-375-3p	Ceacam10	0	1	0	1
rno-miR-375-3p	Cenpc1	1	0	0	1
rno-miR-375-3p	Cept1	1	0	0	1
rno-miR-375-3p	Chst4_predicte	0	1	0	1
rno-miR-375-3p	Chsy1_predict	0	1	0	1
rno-miR-375-3p	Clcn3	0	1	0	1
rno-miR-375-3p	Clec14a	1	0	0	1
rno-miR-375-3p	Clic6	0	1	0	1
rno-miR-375-3p	Clybl	0	1	0	1
rno-miR-375-3p	Cnbp	1	0	0	1
rno-miR-375-3p	Cnot2	0	1	0	1
rno-miR-375-3p	Cntf	0	1	0	1
rno-miR-375-3p	Col11a2	1	0	0	1
rno-miR-375-3p	Col18a1	0	1	0	1
rno-miR-375-3p	Commd8_pred	0	1	0	1
rno-miR-375-3p	Cops4	0	1	0	1
rno-miR-375-3p	Coq3	0	1	0	1
rno-miR-375-3p	Corin	0	1	0	1
rno-miR-375-3p	Cox5a	1	0	0	1
rno-miR-375-3p	Cpne4_predict	0	1	0	1
rno-miR-375-3p	Cpox	1	0	0	1
rno-miR-375-3p	Cpped1	1	0	0	1
rno-miR-375-3p	Cpt1a	1	0	0	1
rno-miR-375-3p	Cry1	1	0	0	1
rno-miR-375-3p	Cryab	0	1	0	1
rno-miR-375-3p	Csf2rb1	0	1	0	1

rno-miR-375-3p	Csprs_predicte	0	1	0	1
rno-miR-375-3p	Ctbp2	1	0	0	1
rno-miR-375-3p	Ctcf	1	0	0	1
rno-miR-375-3p	Cttnbp2	0	1	0	1
rno-miR-375-3p	Cul2	0	0	1	1
rno-miR-375-3p	Cuzd1	0	1	0	1
rno-miR-375-3p	Cxcl3	1	0	0	1
rno-miR-375-3p	Cyc1_predicte	0	1	0	1
rno-miR-375-3p	Cycs	1	0	0	1
rno-miR-375-3p	Cyfip1_predict	0	1	0	1
rno-miR-375-3p	Cyp24a1	1	0	0	1
rno-miR-375-3p	Cyp2a2	1	0	0	1
rno-miR-375-3p	Cyp4f2	0	1	0	1
rno-miR-375-3p	Cytor4	0	1	0	1
rno-miR-375-3p	D330050I23Ri	0	1	0	1
rno-miR-375-3p	Dbh	0	1	0	1
rno-miR-375-3p	Dcn	1	0	0	1
rno-miR-375-3p	Dctn5	1	0	0	1
rno-miR-375-3p	Dcun1d5	1	0	0	1
rno-miR-375-3p	Ddt	0	1	0	1
rno-miR-375-3p	Ddx59	0	1	0	1
rno-miR-375-3p	Deaf1	0	1	0	1
rno-miR-375-3p	Dffa	1	0	0	1
rno-miR-375-3p	Dgka	0	1	0	1
rno-miR-375-3p	DHB13_RAT	0	1	0	1
rno-miR-375-3p	Dhh	0	1	0	1
rno-miR-375-3p	Dhrs3	0	1	0	1
rno-miR-375-3p	Dhrsx_predicte	0	1	0	1
rno-miR-375-3p	Dirc2	0	1	0	1
rno-miR-375-3p	Dlat	0	1	0	1
rno-miR-375-3p	Dlg2	1	0	0	1
rno-miR-375-3p	Dlx5	0	1	0	1
rno-miR-375-3p	Dmgdh	0	1	0	1
rno-miR-375-3p	Dmrtc1c	1	0	0	1
rno-miR-375-3p	Dnase2a	1	0	0	1
rno-miR-375-3p	Dnm3	0	1	0	1
rno-miR-375-3p	Dnmt3b	0	1	0	1
rno-miR-375-3p	Dnmt3l	0	1	0	1
rno-miR-375-3p	Dock5_predict	0	1	0	1
rno-miR-375-3p	Dok3_predicte	0	1	0	1
rno-miR-375-3p	Dpm1_predicte	0	1	0	1
rno-miR-375-3p	Dppa5_predict	0	1	0	1
rno-miR-375-3p	Dpysl3	0	1	0	1
rno-miR-375-3p	Dram	0	0	1	1
rno-miR-375-3p	Drb1	0	1	0	1
rno-miR-375-3p	Dtymk_predict	0	1	0	1
rno-miR-375-3p	Dusp6	1	0	0	1
rno-miR-375-3p	E030019B06R	0	1	0	1
rno-miR-375-3p	E2f6	0	1	0	1
rno-miR-375-3p	Echdc1	1	0	0	1
rno-miR-375-3p	Eftud2	0	1	0	1

rno-miR-375-3p	Egfl3	0	1	0	1
rno-miR-375-3p	Egln1	0	1	0	1
rno-miR-375-3p	Egr4	0	1	0	1
rno-miR-375-3p	Elk4_predicted	0	1	0	1
rno-miR-375-3p	Eml3	0	1	0	1
rno-miR-375-3p	ENSMUSG000	0	1	0	1
rno-miR-375-3p	ENSMUSG000	0	1	0	1
rno-miR-375-3p	ENSMUSG000	0	1	0	1
rno-miR-375-3p	ENSMUSG000	0	1	0	1
rno-miR-375-3p	ENSMUSG000	0	1	0	1
rno-miR-375-3p	ENSMUSG000	0	1	0	1
rno-miR-375-3p	Epb4.1l3	1	0	0	1
rno-miR-375-3p	Epha5	0	1	0	1
rno-miR-375-3p	Eps8l3	0	1	0	1
rno-miR-375-3p	Eraf_predicted	0	1	0	1
rno-miR-375-3p	Ercc6_predicte	0	1	0	1
rno-miR-375-3p	Evl	0	1	0	1
rno-miR-375-3p	Exosc5_predic	0	1	0	1
rno-miR-375-3p	F101B_RAT	0	1	0	1
rno-miR-375-3p	F5	0	1	0	1
rno-miR-375-3p	Faf2	1	0	0	1
rno-miR-375-3p	Fah	0	1	0	1
rno-miR-375-3p	Fam48a	1	0	0	1
rno-miR-375-3p	Fbxo24	0	1	0	1
rno-miR-375-3p	Fbxo39	0	1	0	1
rno-miR-375-3p	Fcgrt	0	1	0	1
rno-miR-375-3p	Fez1	0	1	0	1
rno-miR-375-3p	Fgr	0	1	0	1
rno-miR-375-3p	Fip1l1	0	1	0	1
rno-miR-375-3p	Foxg1	1	0	0	1
rno-miR-375-3p	Frap1	0	1	0	1
rno-miR-375-3p	Fstl1	1	0	0	1
rno-miR-375-3p	Fuz	1	0	0	1
rno-miR-375-3p	Fzd4	0	1	0	1
rno-miR-375-3p	Gabra1	1	0	0	1
rno-miR-375-3p	Galnt7	0	1	0	1
rno-miR-375-3p	Gap43	1	0	0	1
rno-miR-375-3p	Gcm2_predicte	0	1	0	1
rno-miR-375-3p	Gjb5	0	1	0	1
rno-miR-375-3p	Glb1	0	1	0	1
rno-miR-375-3p	Glis2_predicte	0	1	0	1
rno-miR-375-3p	Glt6d1_predict	0	1	0	1
rno-miR-375-3p	Gm1960	0	1	0	1
rno-miR-375-3p	Gm484	0	1	0	1
rno-miR-375-3p	Gmfb	0	1	0	1
rno-miR-375-3p	Gna12	0	1	0	1
rno-miR-375-3p	Golgb1	0	1	0	1
rno-miR-375-3p	Golph3	1	0	0	1
rno-miR-375-3p	GPR34_predic	0	1	0	1
rno-miR-375-3p	Gpr41_predict	0	1	0	1
rno-miR-375-3p	Gpsm3	0	1	0	1

rno-miR-375-3p	Gpx7_predicte	0	1	0	1
rno-miR-375-3p	Gramd1a	0	1	0	1
rno-miR-375-3p	Gria4	1	0	0	1
rno-miR-375-3p	Grip1	1	0	0	1
rno-miR-375-3p	Grip2_v2	0	1	0	1
rno-miR-375-3p	Grk1	0	1	0	1
rno-miR-375-3p	Gsc	0	1	0	1
rno-miR-375-3p	Gsta5	1	0	0	1
rno-miR-375-3p	Gtf2h4	0	1	0	1
rno-miR-375-3p	Gtl3	0	1	0	1
rno-miR-375-3p	Gtpbp8	0	1	0	1
rno-miR-375-3p	Hace1_predict	0	1	0	1
rno-miR-375-3p	Hcfc1r1	0	1	0	1
rno-miR-375-3p	Hddc2_predict	0	1	0	1
rno-miR-375-3p	Hexdc	0	1	0	1
rno-miR-375-3p	Hirip3	0	1	0	1
rno-miR-375-3p	Hist1h2ai_pred	0	1	0	1
rno-miR-375-3p	Hmgn3	1	0	0	1
rno-miR-375-3p	Hoxb13_predic	0	1	0	1
rno-miR-375-3p	Hoxd1_predict	0	1	0	1
rno-miR-375-3p	Hpd	0	1	0	1
rno-miR-375-3p	Hps1	0	1	0	1
rno-miR-375-3p	Hrasls_predict	0	1	0	1
rno-miR-375-3p	Hrh1	0	1	0	1
rno-miR-375-3p	Hrh4	0	1	0	1
rno-miR-375-3p	Hsbp1	1	0	0	1
rno-miR-375-3p	Hsd3b6	1	0	0	1
rno-miR-375-3p	Hsfy2	1	0	0	1
rno-miR-375-3p	Hspb6	0	1	0	1
rno-miR-375-3p	Htatip	0	1	0	1
rno-miR-375-3p	Htatip2_predic	0	1	0	1
rno-miR-375-3p	Htr2b	1	0	0	1
rno-miR-375-3p	Htr4	0	1	0	1
rno-miR-375-3p	Htr5a	0	1	0	1
rno-miR-375-3p	Iars2	0	1	0	1
rno-miR-375-3p	Ibsp	1	0	0	1
rno-miR-375-3p	Idh1	0	1	0	1
rno-miR-375-3p	Ier3	0	1	0	1
rno-miR-375-3p	Igf2bp1	0	1	0	1
rno-miR-375-3p	Igsf6	1	0	0	1
rno-miR-375-3p	Il17ra	0	1	0	1
rno-miR-375-3p	Il22ra2	1	0	0	1
rno-miR-375-3p	Il2ra	0	1	0	1
rno-miR-375-3p	Il4ra	0	1	0	1
rno-miR-375-3p	Inpp4b	1	0	0	1
rno-miR-375-3p	Ins1	0	1	0	1
rno-miR-375-3p	Insig2	1	0	0	1
rno-miR-375-3p	Irak2	0	1	0	1
rno-miR-375-3p	Irak4_predicte	0	1	0	1
rno-miR-375-3p	Irf2_predicted	0	1	0	1
rno-miR-375-3p	Isl2	0	1	0	1

rno-miR-375-3p	Itch	0	1	0	1
rno-miR-375-3p	Itgb3bp	0	1	0	1
rno-miR-375-3p	Itgb8_predicted	0	1	0	1
rno-miR-375-3p	Itpa	0	1	0	1
rno-miR-375-3p	Itpr2	0	1	0	1
rno-miR-375-3p	Jak3	0	1	0	1
rno-miR-375-3p	Jph4	0	1	0	1
rno-miR-375-3p	Jtb	0	1	0	1
rno-miR-375-3p	Kb1	0	1	0	1
rno-miR-375-3p	Kb35	0	1	0	1
rno-miR-375-3p	Kbtbd2_predic	0	1	0	1
rno-miR-375-3p	KCC2B_RAT	0	1	0	1
rno-miR-375-3p	Kcmf1	0	1	0	1
rno-miR-375-3p	Kcnab1	1	0	0	1
rno-miR-375-3p	Kcnab3	0	1	0	1
rno-miR-375-3p	Kcnj15_v2	0	1	0	1
rno-miR-375-3p	Kcnk1	1	0	0	1
rno-miR-375-3p	Kcns1	0	1	0	1
rno-miR-375-3p	Kif27	0	1	0	1
rno-miR-375-3p	Kiss1	0	1	0	1
rno-miR-375-3p	KLC1_RAT	0	1	0	1
rno-miR-375-3p	Klc3	0	1	0	1
rno-miR-375-3p	Klf4	1	0	0	1
rno-miR-375-3p	Klf7_predicted	0	1	0	1
rno-miR-375-3p	Klra5	0	1	0	1
rno-miR-375-3p	Klrd1	1	0	0	1
rno-miR-375-3p	Kprp	1	0	0	1
rno-miR-375-3p	Lactb2	1	0	0	1
rno-miR-375-3p	Lamb3	0	1	0	1
rno-miR-375-3p	Larp1_predicte	0	1	0	1
rno-miR-375-3p	Lass4_predicte	0	1	0	1
rno-miR-375-3p	Lass6	0	1	0	1
rno-miR-375-3p	Lbp	1	0	0	1
rno-miR-375-3p	Ldhb	0	1	0	1
rno-miR-375-3p	Lgals7	0	1	0	1
rno-miR-375-3p	Lhfp	0	1	0	1
rno-miR-375-3p	Lhfpl5	0	1	0	1
rno-miR-375-3p	Lhx2_predicted	0	1	0	1
rno-miR-375-3p	Lhx9	0	1	0	1
rno-miR-375-3p	Lif	0	1	0	1
rno-miR-375-3p	Limk2	0	1	0	1
rno-miR-375-3p	Lims2	1	0	0	1
rno-miR-375-3p	Lipa	1	0	0	1
rno-miR-375-3p	LOC266771	0	1	0	1
rno-miR-375-3p	LOC288526	0	1	0	1
rno-miR-375-3p	LOC290396	0	1	0	1
rno-miR-375-3p	LOC291944	0	1	0	1
rno-miR-375-3p	LOC298643	0	1	0	1
rno-miR-375-3p	LOC305076	0	1	0	1
rno-miR-375-3p	LOC306079	0	1	0	1
rno-miR-375-3p	LOC312299	0	1	0	1

rno-miR-375-3p	LOC313067	0	1	0	1
rno-miR-375-3p	LOC361414	0	1	0	1
rno-miR-375-3p	LOC362056	0	1	0	1
rno-miR-375-3p	LOC362261	0	1	0	1
rno-miR-375-3p	LOC363437	0	1	0	1
rno-miR-375-3p	LOC365778	0	1	0	1
rno-miR-375-3p	LOC367191	0	1	0	1
rno-miR-375-3p	LOC367311	0	1	0	1
rno-miR-375-3p	LOC497940	0	1	0	1
rno-miR-375-3p	LOC497941	0	1	0	1
rno-miR-375-3p	LOC498131	0	1	0	1
rno-miR-375-3p	LOC498145	1	0	0	1
rno-miR-375-3p	LOC498330	1	0	0	1
rno-miR-375-3p	LOC498331	0	1	0	1
rno-miR-375-3p	LOC499022	0	1	0	1
rno-miR-375-3p	LOC499331	1	0	0	1
rno-miR-375-3p	LOC499339	0	1	0	1
rno-miR-375-3p	LOC499719	0	1	0	1
rno-miR-375-3p	LOC499900	0	1	0	1
rno-miR-375-3p	LOC499994	0	1	0	1
rno-miR-375-3p	LOC500292	0	1	0	1
rno-miR-375-3p	LOC500659	0	1	0	1
rno-miR-375-3p	LOC500761	0	1	0	1
rno-miR-375-3p	LOC500827	1	0	0	1
rno-miR-375-3p	LOC501069	0	1	0	1
rno-miR-375-3p	LOC501126	0	1	0	1
rno-miR-375-3p	LOC501617	0	1	0	1
rno-miR-375-3p	LOC606294	0	1	0	1
rno-miR-375-3p	Loc65050	0	1	0	1
rno-miR-375-3p	LOC679102	0	1	0	1
rno-miR-375-3p	LOC679116	0	1	0	1
rno-miR-375-3p	LOC679258	0	1	0	1
rno-miR-375-3p	LOC679341	0	1	0	1
rno-miR-375-3p	LOC679839	0	1	0	1
rno-miR-375-3p	LOC679937	0	1	0	1
rno-miR-375-3p	LOC680478	0	1	0	1
rno-miR-375-3p	LOC680542	0	1	0	1
rno-miR-375-3p	LOC680711	0	1	0	1
rno-miR-375-3p	LOC680913	0	1	0	1
rno-miR-375-3p	LOC681221	0	1	0	1
rno-miR-375-3p	LOC681354	0	1	0	1
rno-miR-375-3p	LOC681446	0	1	0	1
rno-miR-375-3p	LOC681522	0	1	0	1
rno-miR-375-3p	LOC681910	0	1	0	1
rno-miR-375-3p	LOC683510	0	1	0	1
rno-miR-375-3p	LOC683661	0	1	0	1
rno-miR-375-3p	LOC683674	0	1	0	1
rno-miR-375-3p	LOC683863	0	1	0	1
rno-miR-375-3p	LOC683868	0	1	0	1
rno-miR-375-3p	LOC684096	0	1	0	1
rno-miR-375-3p	LOC684204	0	1	0	1

rno-miR-375-3p	LOC684227	0	1	0	1
rno-miR-375-3p	LOC684383	0	1	0	1
rno-miR-375-3p	LOC684889	0	1	0	1
rno-miR-375-3p	LOC685479	0	1	0	1
rno-miR-375-3p	LOC685587	0	1	0	1
rno-miR-375-3p	LOC686449	0	1	0	1
rno-miR-375-3p	LOC686809	0	1	0	1
rno-miR-375-3p	LOC687554	0	1	0	1
rno-miR-375-3p	LOC687849	0	1	0	1
rno-miR-375-3p	LOC688130	0	1	0	1
rno-miR-375-3p	LOC688335	0	1	0	1
rno-miR-375-3p	LOC688422	0	1	0	1
rno-miR-375-3p	LOC688531	0	1	0	1
rno-miR-375-3p	LOC688885	0	1	0	1
rno-miR-375-3p	LOC688887	0	1	0	1
rno-miR-375-3p	LOC689039	0	1	0	1
rno-miR-375-3p	LOC689962	0	1	0	1
rno-miR-375-3p	LOC690057	0	1	0	1
rno-miR-375-3p	LOC690211	0	1	0	1
rno-miR-375-3p	LOC690745	0	1	0	1
rno-miR-375-3p	LOC690911	0	1	0	1
rno-miR-375-3p	LOC690919	0	1	0	1
rno-miR-375-3p	LOC691002	0	1	0	1
rno-miR-375-3p	LOC691098	0	1	0	1
rno-miR-375-3p	LOC691145	0	1	0	1
rno-miR-375-3p	LOC691286	0	1	0	1
rno-miR-375-3p	LOC691835	0	1	0	1
rno-miR-375-3p	Lphn2	1	0	0	1
rno-miR-375-3p	Lphn3	0	1	0	1
rno-miR-375-3p	Lpxn	1	0	0	1
rno-miR-375-3p	Lrfn1_predicte	0	1	0	1
rno-miR-375-3p	Lsg1	0	1	0	1
rno-miR-375-3p	Ltbp4	0	1	0	1
rno-miR-375-3p	Ltf_predicted	0	1	0	1
rno-miR-375-3p	Lypd1	1	0	0	1
rno-miR-375-3p	Lzts2	0	1	0	1
rno-miR-375-3p	Mafg	0	1	0	1
rno-miR-375-3p	Magi2	0	1	0	1
rno-miR-375-3p	Maoa	0	1	0	1
rno-miR-375-3p	Map3k6_predi	0	1	0	1
rno-miR-375-3p	Mapk14	1	0	0	1
rno-miR-375-3p	Mapk6	0	1	0	1
rno-miR-375-3p	Mapkap1	1	0	0	1
rno-miR-375-3p	Mdga2	1	0	0	1
rno-miR-375-3p	Metrn	0	1	0	1
rno-miR-375-3p	Mfap5_predict	0	1	0	1
rno-miR-375-3p	MGC105508	0	1	0	1
rno-miR-375-3p	MGC114439	0	1	0	1
rno-miR-375-3p	MGC114464	0	1	0	1
rno-miR-375-3p	MGC94881	0	1	0	1
rno-miR-375-3p	Mip	0	1	0	1

rno-miR-375-3p	Mitd1	1	0	0	1
rno-miR-375-3p	MITF_RAT	0	1	0	1
rno-miR-375-3p	Mk1	0	1	0	1
rno-miR-375-3p	Mll	0	1	0	1
rno-miR-375-3p	Mlycd	1	0	0	1
rno-miR-375-3p	Mmp7	0	1	0	1
rno-miR-375-3p	Mnda	0	1	0	1
rno-miR-375-3p	Mobp	1	0	0	1
rno-miR-375-3p	Mrgprb1	0	1	0	1
rno-miR-375-3p	Mrpl44	0	1	0	1
rno-miR-375-3p	Mrps25	0	1	0	1
rno-miR-375-3p	Mrps34_predic	0	1	0	1
rno-miR-375-3p	Ms4a10_predi	0	1	0	1
rno-miR-375-3p	Ms4a6b	1	0	0	1
rno-miR-375-3p	Msi1	0	1	0	1
rno-miR-375-3p	Msra	0	1	0	1
rno-miR-375-3p	Msx3	0	1	0	1
rno-miR-375-3p	Mtm1	0	1	0	1
rno-miR-375-3p	Mtmr7_predict	0	1	0	1
rno-miR-375-3p	Myct1_predicte	0	1	0	1
rno-miR-375-3p	MYH8_RAT	0	1	0	1
rno-miR-375-3p	Myh9	0	1	0	1
rno-miR-375-3p	Myl3	0	1	0	1
rno-miR-375-3p	Myo9b	0	1	0	1
rno-miR-375-3p	Myoz2_predict	0	1	0	1
rno-miR-375-3p	Ndufa2_predic	0	1	0	1
rno-miR-375-3p	Ndufaf4	1	0	0	1
rno-miR-375-3p	Nelf	0	1	0	1
rno-miR-375-3p	Nell1	0	1	0	1
rno-miR-375-3p	Net1	0	1	0	1
rno-miR-375-3p	Neu4_predicte	0	1	0	1
rno-miR-375-3p	Nfatc4	0	1	0	1
rno-miR-375-3p	Nkx2-3_predic	0	1	0	1
rno-miR-375-3p	NP_00101315	0	1	0	1
rno-miR-375-3p	NP_00101403	0	1	0	1
rno-miR-375-3p	Npc2	1	0	0	1
rno-miR-375-3p	Nppa	0	1	0	1
rno-miR-375-3p	Nr2c2	1	0	0	1
rno-miR-375-3p	Nr2f1	1	0	0	1
rno-miR-375-3p	Nrg2	0	0	1	1
rno-miR-375-3p	Nrp1	0	1	0	1
rno-miR-375-3p	Nsd1_predicte	0	1	0	1
rno-miR-375-3p	Ntrk2	1	0	0	1
rno-miR-375-3p	Ntrk3	0	1	0	1
rno-miR-375-3p	Nucks1	1	0	0	1
rno-miR-375-3p	Nudt2	1	0	0	1
rno-miR-375-3p	Nudt21	1	0	0	1
rno-miR-375-3p	Nudt7_predicte	0	1	0	1
rno-miR-375-3p	null	0	1	0	1
rno-miR-375-3p	Nxt2_predicted	0	1	0	1
rno-miR-375-3p	Oas1b	1	0	0	1

rno-miR-375-3p	Obfc2a	1	0	0	1
rno-miR-375-3p	Odz2	0	1	0	1
rno-miR-375-3p	Olfm1	1	0	0	1
rno-miR-375-3p	Olig2_predicte	0	1	0	1
rno-miR-375-3p	Olr1000_predi	0	1	0	1
rno-miR-375-3p	Olr1202_predi	0	1	0	1
rno-miR-375-3p	Olr1265_predi	0	1	0	1
rno-miR-375-3p	Olr127_predict	0	1	0	1
rno-miR-375-3p	Olr1404	0	1	0	1
rno-miR-375-3p	Olr142_predict	0	1	0	1
rno-miR-375-3p	Olr143_predict	0	1	0	1
rno-miR-375-3p	Olr1440_predi	0	1	0	1
rno-miR-375-3p	Olr185_predict	0	1	0	1
rno-miR-375-3p	Olr229_predict	0	1	0	1
rno-miR-375-3p	Olr288_predict	0	1	0	1
rno-miR-375-3p	Olr298_predict	0	1	0	1
rno-miR-375-3p	Olr338_predict	0	1	0	1
rno-miR-375-3p	Olr41_predicte	0	1	0	1
rno-miR-375-3p	Olr566_predict	0	1	0	1
rno-miR-375-3p	Olr598_predict	0	1	0	1
rno-miR-375-3p	Olr649_predict	0	1	0	1
rno-miR-375-3p	Olr741_predict	0	1	0	1
rno-miR-375-3p	Olr749_predict	0	1	0	1
rno-miR-375-3p	Olr809_predict	0	1	0	1
rno-miR-375-3p	Olr837	0	1	0	1
rno-miR-375-3p	Olr959_predict	0	1	0	1
rno-miR-375-3p	Oma1_predicte	0	1	0	1
rno-miR-375-3p	Otud6b_predic	0	1	0	1
rno-miR-375-3p	Oxr1	0	1	0	1
rno-miR-375-3p	P11506-10	0	1	0	1
rno-miR-375-3p	P2rx2	1	0	0	1
rno-miR-375-3p	P2ry10_predic	0	1	0	1
rno-miR-375-3p	P97879-2	0	1	0	1
rno-miR-375-3p	Papd4	1	0	0	1
rno-miR-375-3p	Pax6	1	0	0	1
rno-miR-375-3p	Pax6<em><su	0	1	0	1
rno-miR-375-3p	Pcdh10	0	1	0	1
rno-miR-375-3p	Pcdh12	0	1	0	1
rno-miR-375-3p	Pcdh18_predic	0	1	0	1
rno-miR-375-3p	Pcdh7	0	1	0	1
rno-miR-375-3p	Pcdha3	0	1	0	1
rno-miR-375-3p	Pcid2	0	1	0	1
rno-miR-375-3p	Pcp4	0	1	0	1
rno-miR-375-3p	Pcsk5	0	1	0	1
rno-miR-375-3p	Pcsk6	0	1	0	1
rno-miR-375-3p	Pctk3	0	1	0	1
rno-miR-375-3p	Pdcd8	0	1	0	1
rno-miR-375-3p	Pde4a	1	0	0	1
rno-miR-375-3p	Pde6h	0	1	0	1
rno-miR-375-3p	Pde8a	0	1	0	1
rno-miR-375-3p	Pdgfc	0	1	0	1

rno-miR-375-3p	Pdgfrl	0	1	0	1
rno-miR-375-3p	Pdlim3	0	1	0	1
rno-miR-375-3p	Pdp1	1	0	0	1
rno-miR-375-3p	Peo1_predicte	0	1	0	1
rno-miR-375-3p	Pex13_predict	0	1	0	1
rno-miR-375-3p	Pfn1	0	1	0	1
rno-miR-375-3p	Pgsg	0	1	0	1
rno-miR-375-3p	Phf7	0	1	0	1
rno-miR-375-3p	Phlpp1	1	0	0	1
rno-miR-375-3p	Phyhd1	0	1	0	1
rno-miR-375-3p	Phyhipl	0	1	0	1
rno-miR-375-3p	Pib5pa	0	1	0	1
rno-miR-375-3p	Pik3c2b_predi	0	1	0	1
rno-miR-375-3p	Pik3c2g	0	1	0	1
rno-miR-375-3p	Pitpna	1	0	0	1
rno-miR-375-3p	Pkig	0	1	0	1
rno-miR-375-3p	Pla2g10	0	1	0	1
rno-miR-375-3p	Plagl2_predict	0	1	0	1
rno-miR-375-3p	Plekhj1	0	1	0	1
rno-miR-375-3p	Plk2	0	1	0	1
rno-miR-375-3p	Plod3	0	1	0	1
rno-miR-375-3p	Pmpca	0	1	0	1
rno-miR-375-3p	Polb	1	0	0	1
rno-miR-375-3p	Poldip2_predic	0	1	0	1
rno-miR-375-3p	Polr2a	0	1	0	1
rno-miR-375-3p	Polr2h_predict	0	1	0	1
rno-miR-375-3p	Pot1	1	0	0	1
rno-miR-375-3p	Pou2f2	0	1	0	1
rno-miR-375-3p	Ppargc1a	0	1	0	1
rno-miR-375-3p	Ppcs	0	1	0	1
rno-miR-375-3p	Ppm1b	1	0	0	1
rno-miR-375-3p	Ppp2cb	1	0	0	1
rno-miR-375-3p	Pqbp1	0	1	0	1
rno-miR-375-3p	Prb1	1	0	0	1
rno-miR-375-3p	Prg-2	0	1	0	1
rno-miR-375-3p	Prom1	0	1	0	1
rno-miR-375-3p	Prpg2	0	1	0	1
rno-miR-375-3p	Prr7	0	1	0	1
rno-miR-375-3p	Prrx1	1	0	0	1
rno-miR-375-3p	Prx	0	1	0	1
rno-miR-375-3p	Psat1	0	1	0	1
rno-miR-375-3p	Psmb10	1	0	0	1
rno-miR-375-3p	Psmd2	0	1	0	1
rno-miR-375-3p	Psmd7_predic	0	1	0	1
rno-miR-375-3p	Psme1	0	1	0	1
rno-miR-375-3p	Ptprb_predicte	0	1	0	1
rno-miR-375-3p	Pyroxd1	1	0	0	1
rno-miR-375-3p	Q3KR91_RAT	0	1	0	1
rno-miR-375-3p	Q56A21_RAT	0	1	0	1
rno-miR-375-3p	Q5RJK4_RAT	0	1	0	1
rno-miR-375-3p	Q5XFW6_RAT	0	1	0	1

rno-miR-375-3p	Q7TN00_RAT	0	1	0	1
rno-miR-375-3p	Q7TP26_RAT	0	1	0	1
rno-miR-375-3p	Q8C7P0_MOU	0	1	0	1
rno-miR-375-3p	Q8K3P7_RAT	0	1	0	1
rno-miR-375-3p	Q8R1E8_MOU	0	1	0	1
rno-miR-375-3p	Q91ZP2_RAT	0	1	0	1
rno-miR-375-3p	Q99PC0_RAT	0	1	0	1
rno-miR-375-3p	Rab3il1	0	1	0	1
rno-miR-375-3p	Rap1b	1	0	0	1
rno-miR-375-3p	Rasd1	0	1	0	1
rno-miR-375-3p	Rasgrf1	0	1	0	1
rno-miR-375-3p	Rasip1_predic	0	1	0	1
rno-miR-375-3p	Rbclca2	0	1	0	1
rno-miR-375-3p	Rbm17	0	1	0	1
rno-miR-375-3p	Rbm45	1	0	0	1
rno-miR-375-3p	Rbms1	0	1	0	1
rno-miR-375-3p	Rcbtb2	0	1	0	1
rno-miR-375-3p	Recql5_predic	0	1	0	1
rno-miR-375-3p	Rfx3	0	1	0	1
rno-miR-375-3p	Rfx4	0	1	0	1
rno-miR-375-3p	RGD1304783	0	1	0	1
rno-miR-375-3p	RGD1304963_	0	1	0	1
rno-miR-375-3p	RGD1305239_	0	1	0	1
rno-miR-375-3p	RGD1305240_	0	1	0	1
rno-miR-375-3p	RGD1305350_	0	1	0	1
rno-miR-375-3p	RGD1305450_	0	1	0	1
rno-miR-375-3p	RGD1305622	0	1	0	1
rno-miR-375-3p	RGD1305633_	0	1	0	1
rno-miR-375-3p	RGD1305903_	0	1	0	1
rno-miR-375-3p	RGD1305928_	0	1	0	1
rno-miR-375-3p	RGD1306126	0	1	0	1
rno-miR-375-3p	RGD1306212_	0	1	0	1
rno-miR-375-3p	RGD1306702	0	1	0	1
rno-miR-375-3p	RGD1307201_	0	1	0	1
rno-miR-375-3p	RGD1307336	0	1	0	1
rno-miR-375-3p	RGD1307506_	0	1	0	1
rno-miR-375-3p	RGD1307735_	0	1	0	1
rno-miR-375-3p	RGD1307814_	0	1	0	1
rno-miR-375-3p	RGD1307851	0	1	0	1
rno-miR-375-3p	RGD1308064_	0	1	0	1
rno-miR-375-3p	RGD1308084	0	1	0	1
rno-miR-375-3p	RGD1308101_	0	1	0	1
rno-miR-375-3p	RGD1308454	0	1	0	1
rno-miR-375-3p	RGD1308958_	0	1	0	1
rno-miR-375-3p	RGD1309025_	0	1	0	1
rno-miR-375-3p	RGD1309065_	0	1	0	1
rno-miR-375-3p	RGD1309189_	0	1	0	1
rno-miR-375-3p	RGD1309313	0	1	0	1
rno-miR-375-3p	RGD1309403_	0	1	0	1
rno-miR-375-3p	RGD1309540	0	1	0	1
rno-miR-375-3p	RGD1309543_	0	1	0	1

rno-miR-375-3p	RGD1309550	0	1	0	1
rno-miR-375-3p	RGD1309567_	0	1	0	1
rno-miR-375-3p	RGD1309863	0	1	0	1
rno-miR-375-3p	RGD1310039	0	1	0	1
rno-miR-375-3p	RGD1310111	0	1	0	1
rno-miR-375-3p	RGD1310132	0	1	0	1
rno-miR-375-3p	RGD1310137	0	1	0	1
rno-miR-375-3p	RGD1310376_	0	1	0	1
rno-miR-375-3p	RGD1310450	0	1	0	1
rno-miR-375-3p	RGD1310481_	0	1	0	1
rno-miR-375-3p	RGD1310861	1	0	0	1
rno-miR-375-3p	RGD1311122	0	1	0	1
rno-miR-375-3p	RGD1311123	0	1	0	1
rno-miR-375-3p	RGD1311283_	0	1	0	1
rno-miR-375-3p	RGD1311424_	0	1	0	1
rno-miR-375-3p	RGD1311457_	0	1	0	1
rno-miR-375-3p	RGD1311584	0	1	0	1
rno-miR-375-3p	RGD1311752_	0	1	0	1
rno-miR-375-3p	RGD1311848_	0	1	0	1
rno-miR-375-3p	RGD1311914	0	1	0	1
rno-miR-375-3p	RGD1359460	1	0	0	1
rno-miR-375-3p	RGD1359508	0	1	0	1
rno-miR-375-3p	RGD1359509	0	1	0	1
rno-miR-375-3p	RGD1359600	1	0	0	1
rno-miR-375-3p	RGD1359616	0	1	0	1
rno-miR-375-3p	RGD1559457_	0	1	0	1
rno-miR-375-3p	RGD1559522_	0	1	0	1
rno-miR-375-3p	RGD1559740_	0	1	0	1
rno-miR-375-3p	RGD1559783_	0	1	0	1
rno-miR-375-3p	RGD1559882_	0	1	0	1
rno-miR-375-3p	RGD1559899_	0	1	0	1
rno-miR-375-3p	RGD1559926_	0	1	0	1
rno-miR-375-3p	RGD1559931_	0	1	0	1
rno-miR-375-3p	RGD1559961_	0	1	0	1
rno-miR-375-3p	RGD1559995_	0	1	0	1
rno-miR-375-3p	RGD1560084_	0	1	0	1
rno-miR-375-3p	RGD1560290_	0	1	0	1
rno-miR-375-3p	RGD1560307_	0	1	0	1
rno-miR-375-3p	RGD1560424_	0	1	0	1
rno-miR-375-3p	RGD1560459_	0	1	0	1
rno-miR-375-3p	RGD1560572_	0	1	0	1
rno-miR-375-3p	RGD1560603_	0	1	0	1
rno-miR-375-3p	RGD1560748_	0	1	0	1
rno-miR-375-3p	RGD1560788_	0	1	0	1
rno-miR-375-3p	RGD1560958_	0	1	0	1
rno-miR-375-3p	RGD1561203_	0	1	0	1
rno-miR-375-3p	RGD1561521_	0	1	0	1
rno-miR-375-3p	RGD1561652_	0	1	0	1
rno-miR-375-3p	RGD1561842_	0	1	0	1
rno-miR-375-3p	RGD1562029_	0	1	0	1
rno-miR-375-3p	RGD1562236_	0	1	0	1

rno-miR-375-3p	RGD1562252_	0	1	0	1
rno-miR-375-3p	RGD1562289_	0	1	0	1
rno-miR-375-3p	RGD1562331_	0	1	0	1
rno-miR-375-3p	RGD1562438_	0	1	0	1
rno-miR-375-3p	RGD1562622_	0	1	0	1
rno-miR-375-3p	RGD1562629_	0	1	0	1
rno-miR-375-3p	RGD1562646_	0	1	0	1
rno-miR-375-3p	RGD1562653_	0	1	0	1
rno-miR-375-3p	RGD1562922_	0	1	0	1
rno-miR-375-3p	RGD1563019_	0	1	0	1
rno-miR-375-3p	RGD1563047_	0	1	0	1
rno-miR-375-3p	RGD1563106_	0	1	0	1
rno-miR-375-3p	RGD1563422_	0	1	0	1
rno-miR-375-3p	RGD1563429_	0	1	0	1
rno-miR-375-3p	RGD1563615_	0	1	0	1
rno-miR-375-3p	RGD1563619_	0	1	0	1
rno-miR-375-3p	RGD1563657_	0	1	0	1
rno-miR-375-3p	RGD1563986_	0	1	0	1
rno-miR-375-3p	RGD1564051_	0	1	0	1
rno-miR-375-3p	RGD1564306_	0	1	0	1
rno-miR-375-3p	RGD1564316_	0	1	0	1
rno-miR-375-3p	RGD1564385_	0	1	0	1
rno-miR-375-3p	RGD1564396_	0	1	0	1
rno-miR-375-3p	RGD1564634_	0	1	0	1
rno-miR-375-3p	RGD1564894_	0	1	0	1
rno-miR-375-3p	RGD1564950_	0	1	0	1
rno-miR-375-3p	RGD1564994_	0	1	0	1
rno-miR-375-3p	RGD1565073_	0	1	0	1
rno-miR-375-3p	RGD1565091_	0	1	0	1
rno-miR-375-3p	RGD1565159_	0	1	0	1
rno-miR-375-3p	RGD1565212_	0	1	0	1
rno-miR-375-3p	RGD1565222_	0	1	0	1
rno-miR-375-3p	RGD1565385_	0	1	0	1
rno-miR-375-3p	RGD1565555_	0	1	0	1
rno-miR-375-3p	RGD1565557_	0	1	0	1
rno-miR-375-3p	RGD1565820_	0	1	0	1
rno-miR-375-3p	RGD1565845_	0	1	0	1
rno-miR-375-3p	RGD1565856_	0	1	0	1
rno-miR-375-3p	RGD1565857_	0	1	0	1
rno-miR-375-3p	RGD1566083_	0	1	0	1
rno-miR-375-3p	RGD1566224_	0	1	0	1
rno-miR-375-3p	RGD1566232_	0	1	0	1
rno-miR-375-3p	RGD1566413_	0	1	0	1
rno-miR-375-3p	RGD727788	0	1	0	1
rno-miR-375-3p	RGD735029	1	0	0	1
rno-miR-375-3p	Rgs10	0	1	0	1
rno-miR-375-3p	Rhbdf1	0	1	0	1
rno-miR-375-3p	Rhbdl1	0	1	0	1
rno-miR-375-3p	Rimbp2	0	1	0	1
rno-miR-375-3p	Rims1	1	0	0	1
rno-miR-375-3p	Ring1	0	1	0	1

rno-miR-375-3p	Rnase11	0	1	0	1
rno-miR-375-3p	Rnf135	0	1	0	1
rno-miR-375-3p	Rnf213	0	1	0	1
rno-miR-375-3p	Rnf4	0	1	0	1
rno-miR-375-3p	Rnls	1	0	0	1
rno-miR-375-3p	Rock2	0	1	0	1
rno-miR-375-3p	Rom1	0	1	0	1
rno-miR-375-3p	Rora_predicted	0	1	0	1
rno-miR-375-3p	Rpl10a	0	1	0	1
rno-miR-375-3p	Rpo1-2	0	1	0	1
rno-miR-375-3p	Rprm	0	1	0	1
rno-miR-375-3p	Rps11	1	0	0	1
rno-miR-375-3p	Rps2	0	1	0	1
rno-miR-375-3p	Rps3	1	0	0	1
rno-miR-375-3p	Rps6kc1	0	1	0	1
rno-miR-375-3p	RT1-DOb	0	1	0	1
rno-miR-375-3p	Saa4	0	1	0	1
rno-miR-375-3p	Scap1	0	1	0	1
rno-miR-375-3p	Scgb1c1_pred	0	1	0	1
rno-miR-375-3p	Scgb2a2	1	0	0	1
rno-miR-375-3p	Scn11a	0	1	0	1
rno-miR-375-3p	Scn1a	0	1	0	1
rno-miR-375-3p	Sdccag3	0	1	0	1
rno-miR-375-3p	Sdfr1	0	1	0	1
rno-miR-375-3p	Sdsl_predicted	0	1	0	1
rno-miR-375-3p	Sec13	1	0	0	1
rno-miR-375-3p	Sec24a_predic	0	1	0	1
rno-miR-375-3p	Sec24b	0	1	0	1
rno-miR-375-3p	Sectm1a	1	0	0	1
rno-miR-375-3p	Sell	1	0	0	1
rno-miR-375-3p	Serpini1	1	0	0	1
rno-miR-375-3p	Sfrs10	1	0	0	1
rno-miR-375-3p	Sfxn4_predicte	0	1	0	1
rno-miR-375-3p	Sh2d4a	1	0	0	1
rno-miR-375-3p	Shb	0	1	0	1
rno-miR-375-3p	Shox2	1	0	0	1
rno-miR-375-3p	Sirt2	1	0	0	1
rno-miR-375-3p	Siva_predicted	0	1	0	1
rno-miR-375-3p	Slc18a1	0	1	0	1
rno-miR-375-3p	Slc1a7_predict	0	1	0	1
rno-miR-375-3p	Slc26a7_predi	0	1	0	1
rno-miR-375-3p	Slc35a3	1	0	0	1
rno-miR-375-3p	Slc35b2	1	0	0	1
rno-miR-375-3p	Slc5a5	0	1	0	1
rno-miR-375-3p	Slc8a1	1	0	0	1
rno-miR-375-3p	Slc9a5	0	1	0	1
rno-miR-375-3p	Slco1b2	0	1	0	1
rno-miR-375-3p	Slco4a1	0	1	0	1
rno-miR-375-3p	Slitrk5	0	0	1	1
rno-miR-375-3p	Smc5l1_predic	0	1	0	1
rno-miR-375-3p	Smgb	0	1	0	1

rno-miR-375-3p	Smn1	0	1	0	1
rno-miR-375-3p	Snip	0	1	0	1
rno-miR-375-3p	Snx24	0	1	0	1
rno-miR-375-3p	Sorl1_predicte	0	1	0	1
rno-miR-375-3p	Sp5_predicted	0	1	0	1
rno-miR-375-3p	Spcs1	0	1	0	1
rno-miR-375-3p	Spire2_predict	0	1	0	1
rno-miR-375-3p	Srrm2_predict	0	1	0	1
rno-miR-375-3p	Sstr1	1	0	0	1
rno-miR-375-3p	ST2A1_RAT	0	1	0	1
rno-miR-375-3p	St7l	0	1	0	1
rno-miR-375-3p	St8sia3	0	1	0	1
rno-miR-375-3p	Stard6	0	1	0	1
rno-miR-375-3p	Stc2	0	1	0	1
rno-miR-375-3p	Stk11_predicte	0	1	0	1
rno-miR-375-3p	Stk17b	1	0	0	1
rno-miR-375-3p	Strada	1	0	0	1
rno-miR-375-3p	Strbp	1	0	0	1
rno-miR-375-3p	Sts	0	1	0	1
rno-miR-375-3p	Sub1	1	0	0	1
rno-miR-375-3p	Sult1c1	0	1	0	1
rno-miR-375-3p	Sumo2	1	0	0	1
rno-miR-375-3p	Surb7_predicte	0	1	0	1
rno-miR-375-3p	Svil_predicted	0	1	0	1
rno-miR-375-3p	Syncrip	0	1	0	1
rno-miR-375-3p	Sytl4	0	1	0	1
rno-miR-375-3p	Taf1_predicted	0	1	0	1
rno-miR-375-3p	Taldo1	0	1	0	1
rno-miR-375-3p	Tas1r1	0	1	0	1
rno-miR-375-3p	Tcea3	1	0	0	1
rno-miR-375-3p	Tcf2	0	1	0	1
rno-miR-375-3p	Tex101	0	1	0	1
rno-miR-375-3p	Tfip11	0	1	0	1
rno-miR-375-3p	Tgfb1	0	1	0	1
rno-miR-375-3p	Tgif	0	1	0	1
rno-miR-375-3p	Tgm1	0	1	0	1
rno-miR-375-3p	Timm8a	0	1	0	1
rno-miR-375-3p	Timp2	0	1	0	1
rno-miR-375-3p	Tmco1	0	1	0	1
rno-miR-375-3p	Tmed5	1	0	0	1
rno-miR-375-3p	Tmeff2_predic	0	1	0	1
rno-miR-375-3p	Tmem110	0	1	0	1
rno-miR-375-3p	Tmem119_pre	0	1	0	1
rno-miR-375-3p	Tmem16f_pre	0	1	0	1
rno-miR-375-3p	Tmem23	0	1	0	1
rno-miR-375-3p	Tmem40	0	1	0	1
rno-miR-375-3p	Tmem41b	1	0	0	1
rno-miR-375-3p	Tmem53_pred	0	1	0	1
rno-miR-375-3p	Tmem63c_pre	0	1	0	1
rno-miR-375-3p	Tmem67	0	0	1	1
rno-miR-375-3p	Tmem93	0	0	1	1

rno-miR-375-3p	Tmlhe	1	0	0	1
rno-miR-375-3p	Tmprss11d	0	1	0	1
rno-miR-375-3p	Tns4	0	1	0	1
rno-miR-375-3p	Tpm3	1	0	0	1
rno-miR-375-3p	Tpo	0	1	0	1
rno-miR-375-3p	Tpp2	1	0	0	1
rno-miR-375-3p	TRAV13-5	0	1	0	1
rno-miR-375-3p	TRAV19	0	1	0	1
rno-miR-375-3p	Trim23	0	1	0	1
rno-miR-375-3p	Trim3	0	1	0	1
rno-miR-375-3p	Trim39	1	0	0	1
rno-miR-375-3p	Trim55	1	0	0	1
rno-miR-375-3p	Trip12	1	0	0	1
rno-miR-375-3p	Trpc4	1	0	0	1
rno-miR-375-3p	Trpc5	0	1	0	1
rno-miR-375-3p	Trps1_predicte	0	1	0	1
rno-miR-375-3p	Tspan12	1	0	0	1
rno-miR-375-3p	Ttc18_predicte	0	1	0	1
rno-miR-375-3p	Ttc24	0	1	0	1
rno-miR-375-3p	Ttc5	1	0	0	1
rno-miR-375-3p	Twist2	0	1	0	1
rno-miR-375-3p	Txndc3	1	0	0	1
rno-miR-375-3p	Uba3	1	0	0	1
rno-miR-375-3p	Ube3a_predict	0	1	0	1
rno-miR-375-3p	Ubqln2	0	1	0	1
rno-miR-375-3p	Ucp1	0	1	0	1
rno-miR-375-3p	Unc84a	0	1	0	1
rno-miR-375-3p	Usp32_predict	0	1	0	1
rno-miR-375-3p	V1ra8	0	1	0	1
rno-miR-375-3p	V1re16	0	1	0	1
rno-miR-375-3p	V1re17	0	1	0	1
rno-miR-375-3p	V1rm2	0	1	0	1
rno-miR-375-3p	Vcsa1	1	0	0	1
rno-miR-375-3p	Vegfa	1	0	0	1
rno-miR-375-3p	Veph1	0	1	0	1
rno-miR-375-3p	Vkorc1	1	0	0	1
rno-miR-375-3p	Vsig1	0	1	0	1
rno-miR-375-3p	Wbscr17	0	1	0	1
rno-miR-375-3p	Wdhd1	0	1	0	1
rno-miR-375-3p	Wnt2b	0	1	0	1
rno-miR-375-3p	Wwox	0	1	0	1
rno-miR-375-3p	Xlkd1_predicte	0	1	0	1
rno-miR-375-3p	XR_008291.1	0	1	0	1
rno-miR-375-3p	Xrcc3	0	1	0	1
rno-miR-375-3p	XRG9	0	1	0	1
rno-miR-375-3p	Ywhaz	1	0	0	1
rno-miR-375-3p	Zan_predicted	0	1	0	1
rno-miR-375-3p	Zar1	0	1	0	1
rno-miR-375-3p	Zbtb16	1	0	0	1
rno-miR-375-3p	Zc3h15	1	0	0	1
rno-miR-375-3p	Zcchc12	0	1	0	1

rno-miR-375-3p	Zdhhc20	1	0	0	1
rno-miR-375-3p	Zfp276	0	1	0	1
rno-miR-375-3p	Zfp329_predic	0	1	0	1
rno-miR-375-3p	Zfp334	0	1	0	1
rno-miR-375-3p	Zfp339_predic	0	1	0	1
rno-miR-375-3p	Zfp39	0	0	1	1
rno-miR-375-3p	Zhx1	1	0	0	1
rno-miR-375-3p	Znf251_predic	0	1	0	1
rno-miR-375-3p	Znf579_predic	0	1	0	1
rno-miR-873-5p	1600002H07R	0	1	0	1
rno-miR-873-5p	2310073E15R	0	1	0	1
rno-miR-873-5p	2600006K01R	0	1	0	1
rno-miR-873-5p	2700049A03R	0	1	0	1
rno-miR-873-5p	2810002I04Rik	0	1	0	1
rno-miR-873-5p	4930519N13R	0	1	0	1
rno-miR-873-5p	4933425L03Ri	0	1	0	1
rno-miR-873-5p	4933439F18Ri	0	1	0	1
rno-miR-873-5p	A2NXR7_RAT	0	1	0	1
rno-miR-873-5p	A530016L24R	0	1	0	1
rno-miR-873-5p	A630023P12R	0	1	0	1
rno-miR-873-5p	A930003A15R	0	1	0	1
rno-miR-873-5p	Abca13	0	1	0	1
rno-miR-873-5p	Abca2	1	0	0	1
rno-miR-873-5p	Abcc4	0	1	0	1
rno-miR-873-5p	Abcc5	1	0	0	1
rno-miR-873-5p	Abcf3	0	1	0	1
rno-miR-873-5p	Abcg3l2	1	0	0	1
rno-miR-873-5p	Abhd1	1	0	0	1
rno-miR-873-5p	Abt1	0	1	0	1
rno-miR-873-5p	Abtb1	0	1	0	1
rno-miR-873-5p	Acacb	0	1	0	1
rno-miR-873-5p	Acads	1	0	0	1
rno-miR-873-5p	Acot1	1	0	0	1
rno-miR-873-5p	Acr	0	1	0	1
rno-miR-873-5p	Actb	0	1	0	1
rno-miR-873-5p	Actg1	0	1	0	1
rno-miR-873-5p	Actg2	0	1	0	1
rno-miR-873-5p	Actl6b_predict	0	1	0	1
rno-miR-873-5p	Actr1b	1	0	0	1
rno-miR-873-5p	Adam17	1	0	0	1
rno-miR-873-5p	Adam7	1	0	0	1
rno-miR-873-5p	Adamts1	1	0	0	1
rno-miR-873-5p	Adamts14_pre	0	1	0	1
rno-miR-873-5p	Adamtsl5_pred	0	1	0	1
rno-miR-873-5p	Adcy6	1	0	0	1
rno-miR-873-5p	Adh7	1	0	0	1
rno-miR-873-5p	Adk	1	0	0	1
rno-miR-873-5p	Adrb2	0	1	0	1
rno-miR-873-5p	Adrb3	0	1	0	1
rno-miR-873-5p	Aga	0	1	0	1
rno-miR-873-5p	Agtr1b	1	0	0	1

rno-miR-873-5p	Ahi1	0	1	0	1
rno-miR-873-5p	Ahrr	1	0	0	1
rno-miR-873-5p	Aim1l_predicte	0	1	0	1
rno-miR-873-5p	Ak2	1	0	0	1
rno-miR-873-5p	Akap3	1	0	0	1
rno-miR-873-5p	Akap6	1	0	0	1
rno-miR-873-5p	Akap9	0	1	0	1
rno-miR-873-5p	Akr1c18	1	0	0	1
rno-miR-873-5p	Akt2	0	1	0	1
rno-miR-873-5p	Aldh3a2	1	0	0	1
rno-miR-873-5p	Ampd3	1	0	0	1
rno-miR-873-5p	Anapc2	0	1	0	1
rno-miR-873-5p	Ankrd34a	1	0	0	1
rno-miR-873-5p	Ankrd41	0	1	0	1
rno-miR-873-5p	Aoc3	1	0	0	1
rno-miR-873-5p	Ap2m1	1	0	0	1
rno-miR-873-5p	Ap3m1	1	0	0	1
rno-miR-873-5p	Apom	1	0	0	1
rno-miR-873-5p	Appl2	0	0	1	1
rno-miR-873-5p	Arf3	1	0	0	1
rno-miR-873-5p	Arfgap2	1	0	0	1
rno-miR-873-5p	Arglu1	1	0	0	1
rno-miR-873-5p	Arhgef17	0	1	0	1
rno-miR-873-5p	Arhgef19_pred	0	1	0	1
rno-miR-873-5p	Arhgef9	1	0	0	1
rno-miR-873-5p	Arih1	1	0	0	1
rno-miR-873-5p	Arl5b	1	0	0	1
rno-miR-873-5p	Armet_predicte	0	1	0	1
rno-miR-873-5p	Arnt	0	1	0	1
rno-miR-873-5p	Arpc5	1	0	0	1
rno-miR-873-5p	Arts1	0	1	0	1
rno-miR-873-5p	Asah1	1	0	0	1
rno-miR-873-5p	Aste1	0	1	0	1
rno-miR-873-5p	Asxl2_predicte	0	1	0	1
rno-miR-873-5p	Atad1	1	0	0	1
rno-miR-873-5p	Atcay_predicte	0	1	0	1
rno-miR-873-5p	Atf6b	1	0	0	1
rno-miR-873-5p	Atg9a	1	0	0	1
rno-miR-873-5p	Atp5l	1	0	0	1
rno-miR-873-5p	Atp6ap1	0	1	0	1
rno-miR-873-5p	Atp6v1e1	1	0	0	1
rno-miR-873-5p	Atxn1	1	0	0	1
rno-miR-873-5p	Atxn10	0	1	0	1
rno-miR-873-5p	Atxn2l_predict	0	1	0	1
rno-miR-873-5p	Aurkb	1	0	0	1
rno-miR-873-5p	Axl	0	1	0	1
rno-miR-873-5p	Azi2	1	0	0	1
rno-miR-873-5p	B3gat1	1	0	0	1
rno-miR-873-5p	B3gnt1_predic	0	1	0	1
rno-miR-873-5p	B3gntl1	1	0	0	1
rno-miR-873-5p	B4galt3	0	0	1	1

rno-miR-873-5p	Baalc	1	0	0	1
rno-miR-873-5p	Barx1_predicte	0	1	0	1
rno-miR-873-5p	Bax	1	0	0	1
rno-miR-873-5p	Bbs2	0	1	0	1
rno-miR-873-5p	Bbs7	0	1	0	1
rno-miR-873-5p	Bcl2a1d	1	0	0	1
rno-miR-873-5p	Bcl2l1	1	0	0	1
rno-miR-873-5p	Bcl2l2	1	0	0	1
rno-miR-873-5p	Bex1	1	0	0	1
rno-miR-873-5p	Bnip3	1	0	0	1
rno-miR-873-5p	Bod1	1	0	0	1
rno-miR-873-5p	Bpgm	1	0	0	1
rno-miR-873-5p	Bsnd	1	0	0	1
rno-miR-873-5p	Btbd10	1	0	0	1
rno-miR-873-5p	Btg3	1	0	0	1
rno-miR-873-5p	Btnl1	1	0	0	1
rno-miR-873-5p	Bwk1	1	0	0	1
rno-miR-873-5p	Bzw1	1	0	0	1
rno-miR-873-5p	C1qc	1	0	0	1
rno-miR-873-5p	C1qg	0	1	0	1
rno-miR-873-5p	C1ql1	0	1	0	1
rno-miR-873-5p	C1qtnf5	0	1	0	1
rno-miR-873-5p	Cables1_predi	0	1	0	1
rno-miR-873-5p	Cacna1a	0	1	0	1
rno-miR-873-5p	Cacna1b	1	0	0	1
rno-miR-873-5p	Cacna1c	1	0	0	1
rno-miR-873-5p	Cacna2d1	1	0	0	1
rno-miR-873-5p	Cacnb3	1	0	0	1
rno-miR-873-5p	Cacng4	0	1	0	1
rno-miR-873-5p	Camlg	1	0	0	1
rno-miR-873-5p	Car13	0	0	1	1
rno-miR-873-5p	Car3	1	0	0	1
rno-miR-873-5p	Casp2	1	0	0	1
rno-miR-873-5p	Casp3	1	0	0	1
rno-miR-873-5p	Casp7	1	0	0	1
rno-miR-873-5p	Cblb	1	0	0	1
rno-miR-873-5p	Cbx6	1	0	0	1
rno-miR-873-5p	Ccbl1	1	0	0	1
rno-miR-873-5p	Ccdc117	1	0	0	1
rno-miR-873-5p	Ccdc19	0	1	0	1
rno-miR-873-5p	Ccdc32	1	0	0	1
rno-miR-873-5p	Ccdc42	0	0	1	1
rno-miR-873-5p	Ccdc42_predic	0	1	0	1
rno-miR-873-5p	Ccdc53_predic	0	1	0	1
rno-miR-873-5p	Ccnb1ip1	1	0	0	1
rno-miR-873-5p	Ccr5	1	0	0	1
rno-miR-873-5p	Cct6a	1	0	0	1
rno-miR-873-5p	Cd14	0	1	0	1
rno-miR-873-5p	Cd200	1	0	0	1
rno-miR-873-5p	Cd22_predicte	0	1	0	1
rno-miR-873-5p	Cd34_predicte	0	1	0	1

rno-miR-873-5p	Cd36	1	0	0	1
rno-miR-873-5p	Cd44	0	1	0	1
rno-miR-873-5p	Cd5l	1	0	0	1
rno-miR-873-5p	Cd79b	1	0	0	1
rno-miR-873-5p	Cd96	0	1	0	1
rno-miR-873-5p	Cdc16	0	1	0	1
rno-miR-873-5p	Cdc26	1	0	0	1
rno-miR-873-5p	Cdc27	1	0	0	1
rno-miR-873-5p	Cdc42ep4	0	0	1	1
rno-miR-873-5p	Cdca3	1	0	0	1
rno-miR-873-5p	Cdk10	0	1	0	1
rno-miR-873-5p	Cdk12	0	0	1	1
rno-miR-873-5p	Cdkn1c	1	0	0	1
rno-miR-873-5p	Cdkn2b	1	0	0	1
rno-miR-873-5p	Cdrt4	0	1	0	1
rno-miR-873-5p	Ceacam1	1	0	0	1
rno-miR-873-5p	Cend1	1	0	0	1
rno-miR-873-5p	Cep63	1	0	0	1
rno-miR-873-5p	Cercam	1	0	0	1
rno-miR-873-5p	Cers6	0	0	1	1
rno-miR-873-5p	Cflar	1	0	0	1
rno-miR-873-5p	Cgrrf1	1	0	0	1
rno-miR-873-5p	Ch25h	1	0	0	1
rno-miR-873-5p	Chchd4	1	0	0	1
rno-miR-873-5p	Chd1l_predicte	0	1	0	1
rno-miR-873-5p	Chgb	1	0	0	1
rno-miR-873-5p	Chic2_predicte	0	1	0	1
rno-miR-873-5p	Chm	1	0	0	1
rno-miR-873-5p	Chn1	1	0	0	1
rno-miR-873-5p	Chp	1	0	0	1
rno-miR-873-5p	Chrdl1	1	0	0	1
rno-miR-873-5p	Chrnb3	1	0	0	1
rno-miR-873-5p	Chst3	0	1	0	1
rno-miR-873-5p	Cir1	1	0	0	1
rno-miR-873-5p	Cited4	0	1	0	1
rno-miR-873-5p	Clcn1	0	1	0	1
rno-miR-873-5p	Clcn6_predicte	0	1	0	1
rno-miR-873-5p	Cldn11	1	0	0	1
rno-miR-873-5p	Cldn6	0	0	1	1
rno-miR-873-5p	Cldn6_predicte	0	1	0	1
rno-miR-873-5p	Clec2d	1	0	0	1
rno-miR-873-5p	Clic2	1	0	0	1
rno-miR-873-5p	Clic3	1	0	0	1
rno-miR-873-5p	Clns1a	1	0	0	1
rno-miR-873-5p	Clpx	0	1	0	1
rno-miR-873-5p	Cma1	0	1	0	1
rno-miR-873-5p	Cmpk1	1	0	0	1
rno-miR-873-5p	Cmtm6	1	0	0	1
rno-miR-873-5p	Cnga4	0	1	0	1
rno-miR-873-5p	Cnih3	0	1	0	1
rno-miR-873-5p	Cnot4	1	0	0	1

rno-miR-873-5p	Cntf	1	0	0	1
rno-miR-873-5p	Col23a1	0	1	0	1
rno-miR-873-5p	Col6a1_predic	0	1	0	1
rno-miR-873-5p	Cox5a	1	0	0	1
rno-miR-873-5p	Cox6c	1	0	0	1
rno-miR-873-5p	Cox6c1	1	0	0	1
rno-miR-873-5p	Cpg1	1	0	0	1
rno-miR-873-5p	Cplx2	1	0	0	1
rno-miR-873-5p	Cpne5_predict	0	1	0	1
rno-miR-873-5p	Cpped1	1	0	0	1
rno-miR-873-5p	Cpt1a	1	0	0	1
rno-miR-873-5p	Cr1l	1	0	0	1
rno-miR-873-5p	Creb3l1	1	0	0	1
rno-miR-873-5p	Crebl1	0	1	0	1
rno-miR-873-5p	Crebl2	1	0	0	1
rno-miR-873-5p	Crisp2	1	0	0	1
rno-miR-873-5p	Crkrs	1	0	0	1
rno-miR-873-5p	Crot	1	0	0	1
rno-miR-873-5p	Crtc1	0	1	0	1
rno-miR-873-5p	Cryge	1	0	0	1
rno-miR-873-5p	Crygs	0	1	0	1
rno-miR-873-5p	Cryz	1	0	0	1
rno-miR-873-5p	Csda	1	0	0	1
rno-miR-873-5p	Csf3r_predicte	0	1	0	1
rno-miR-873-5p	Ctbp1	1	0	0	1
rno-miR-873-5p	Cte1	0	1	0	1
rno-miR-873-5p	Ctns_predicted	0	1	0	1
rno-miR-873-5p	Cwf19l2_predi	0	1	0	1
rno-miR-873-5p	Cxcl12	1	0	0	1
rno-miR-873-5p	Cxcr4	1	0	0	1
rno-miR-873-5p	Cygb	1	0	0	1
rno-miR-873-5p	Cyld	0	0	1	1
rno-miR-873-5p	Cyp11b1	1	0	0	1
rno-miR-873-5p	Cyp1a1	1	0	0	1
rno-miR-873-5p	Cyp21a1	0	1	0	1
rno-miR-873-5p	Cyp27a1	0	1	0	1
rno-miR-873-5p	Cyp2c13	1	0	0	1
rno-miR-873-5p	Cyp2c7	0	1	0	1
rno-miR-873-5p	Cyp4b1	1	0	0	1
rno-miR-873-5p	Cyp4f4	0	1	0	1
rno-miR-873-5p	Cyp51	1	0	0	1
rno-miR-873-5p	Cyyr1	1	0	0	1
rno-miR-873-5p	D16Bwg1494e	0	1	0	1
rno-miR-873-5p	Dars2	0	1	0	1
rno-miR-873-5p	Dazap2	1	0	0	1
rno-miR-873-5p	Dctn5	1	0	0	1
rno-miR-873-5p	Dd5	0	1	0	1
rno-miR-873-5p	Ddb2	0	1	0	1
rno-miR-873-5p	Ddc	1	0	0	1
rno-miR-873-5p	Ddr1	0	1	0	1
rno-miR-873-5p	Ddx21	1	0	0	1

rno-miR-873-5p	Ddx5	1	0	0	1
rno-miR-873-5p	Def8	1	0	0	1
rno-miR-873-5p	Defb21	0	1	0	1
rno-miR-873-5p	Defb52	1	0	0	1
rno-miR-873-5p	Dennd4b	0	1	0	1
rno-miR-873-5p	Derl1	1	0	0	1
rno-miR-873-5p	Dffa	1	0	0	1
rno-miR-873-5p	Dgcr6_predicte	0	1	0	1
rno-miR-873-5p	Dgka	0	1	0	1
rno-miR-873-5p	Dhrs1	0	1	0	1
rno-miR-873-5p	Dhrs9	0	1	0	1
rno-miR-873-5p	Dhx38_predict	0	1	0	1
rno-miR-873-5p	Dicer1	0	1	0	1
rno-miR-873-5p	DISC1_RAT	0	1	0	1
rno-miR-873-5p	Dixdc1	1	0	0	1
rno-miR-873-5p	Dlat	1	0	0	1
rno-miR-873-5p	Dlgh2	0	1	0	1
rno-miR-873-5p	DLP2	0	1	0	1
rno-miR-873-5p	Dmpk_predicte	0	1	0	1
rno-miR-873-5p	Dnajb9	1	0	0	1
rno-miR-873-5p	Dnajc12	1	0	0	1
rno-miR-873-5p	Dnajc9_predic	0	1	0	1
rno-miR-873-5p	Dnali1	1	0	0	1
rno-miR-873-5p	Dnd1	0	1	0	1
rno-miR-873-5p	Dnm1l	1	0	0	1
rno-miR-873-5p	Dock6	0	1	0	1
rno-miR-873-5p	Dpf2	0	0	1	1
rno-miR-873-5p	Dph2	0	1	0	1
rno-miR-873-5p	Dpysl5	0	1	0	1
rno-miR-873-5p	Dr1	1	0	0	1
rno-miR-873-5p	Drg2	0	1	0	1
rno-miR-873-5p	Dsg3	0	1	0	1
rno-miR-873-5p	Dtnb	1	0	0	1
rno-miR-873-5p	Dusp9	1	0	0	1
rno-miR-873-5p	Dync1li2	1	0	0	1
rno-miR-873-5p	Dync2li1	1	0	0	1
rno-miR-873-5p	Dynll2	1	0	0	1
rno-miR-873-5p	Dyt1	0	1	0	1
rno-miR-873-5p	Ece1	1	0	0	1
rno-miR-873-5p	Edc4	1	0	0	1
rno-miR-873-5p	Edn2	1	0	0	1
rno-miR-873-5p	Efna4_predicte	0	1	0	1
rno-miR-873-5p	Efnb2	0	1	0	1
rno-miR-873-5p	Efs	0	0	1	1
rno-miR-873-5p	Eftud2	0	1	0	1
rno-miR-873-5p	EG244911	0	1	0	1
rno-miR-873-5p	Egln3	1	0	0	1
rno-miR-873-5p	Ehd4	1	0	0	1
rno-miR-873-5p	Eif1a	1	0	0	1
rno-miR-873-5p	Eif2ak3	1	0	0	1
rno-miR-873-5p	Eif2b5	1	0	0	1

rno-miR-873-5p	Eif3b	1	0	0	1
rno-miR-873-5p	Eif3f	0	0	1	1
rno-miR-873-5p	Eif3h	1	0	0	1
rno-miR-873-5p	Eif3s9	0	1	0	1
rno-miR-873-5p	Eif4a2	1	0	0	1
rno-miR-873-5p	Eif4e	1	0	0	1
rno-miR-873-5p	Eif4g1	0	1	0	1
rno-miR-873-5p	Eif5	1	0	0	1
rno-miR-873-5p	Elf1	1	0	0	1
rno-miR-873-5p	Ell3	0	1	0	1
rno-miR-873-5p	Elovl3_predict	0	1	0	1
rno-miR-873-5p	Elovl6	1	0	0	1
rno-miR-873-5p	Emcn	1	0	0	1
rno-miR-873-5p	Ensa	1	0	0	1
rno-miR-873-5p	ENSMUSG000	0	1	0	1
rno-miR-873-5p	ENSMUSG000	0	1	0	1
rno-miR-873-5p	ENSMUSG000	0	1	0	1
rno-miR-873-5p	Entpd6	1	0	0	1
rno-miR-873-5p	Epc1	0	1	0	1
rno-miR-873-5p	Epha5	0	1	0	1
rno-miR-873-5p	Ephb6	0	1	0	1
rno-miR-873-5p	Ergic2	1	0	0	1
rno-miR-873-5p	Ermp1	1	0	0	1
rno-miR-873-5p	Erv3	0	1	0	1
rno-miR-873-5p	Ets1	1	0	0	1
rno-miR-873-5p	Evpl_predicted	0	1	0	1
rno-miR-873-5p	F13a1	1	0	0	1
rno-miR-873-5p	Faah	0	1	0	1
rno-miR-873-5p	Fam101b	1	0	0	1
rno-miR-873-5p	Fam107b	1	0	0	1
rno-miR-873-5p	Fam122a	1	0	0	1
rno-miR-873-5p	Fam12b	1	0	0	1
rno-miR-873-5p	Fam149b1	1	0	0	1
rno-miR-873-5p	Fam26e	1	0	0	1
rno-miR-873-5p	Fam82a1	1	0	0	1
rno-miR-873-5p	Farslb	0	1	0	1
rno-miR-873-5p	Faslg	1	0	0	1
rno-miR-873-5p	Fastk	1	0	0	1
rno-miR-873-5p	Fbf1	0	1	0	1
rno-miR-873-5p	Fbxo17	1	0	0	1
rno-miR-873-5p	Fbxo28_predic	0	1	0	1
rno-miR-873-5p	Fbxo4_predict	0	1	0	1
rno-miR-873-5p	Fcgr3a	1	0	0	1
rno-miR-873-5p	Fgd1	0	1	0	1
rno-miR-873-5p	Fhl1	1	0	0	1
rno-miR-873-5p	Fhl4	1	0	0	1
rno-miR-873-5p	Fkbp5	1	0	0	1
rno-miR-873-5p	Fkbp9	1	0	0	1
rno-miR-873-5p	Fliih	0	1	0	1
rno-miR-873-5p	Flot1	1	0	0	1
rno-miR-873-5p	Fmip	0	1	0	1

rno-miR-873-5p	Fn1	0	1	0	1
rno-miR-873-5p	Fos	1	0	0	1
rno-miR-873-5p	Foxk2	0	0	1	1
rno-miR-873-5p	Foxo3	0	1	0	1
rno-miR-873-5p	Frmd8	1	0	0	1
rno-miR-873-5p	Fuk_predicted	0	1	0	1
rno-miR-873-5p	Fut1	1	0	0	1
rno-miR-873-5p	Fut2	1	0	0	1
rno-miR-873-5p	Fut7	1	0	0	1
rno-miR-873-5p	Gabpb1	1	0	0	1
rno-miR-873-5p	Gabpb2	0	1	0	1
rno-miR-873-5p	Gabrb2	0	1	0	1
rno-miR-873-5p	Galnt11	0	1	0	1
rno-miR-873-5p	Galnt3	1	0	0	1
rno-miR-873-5p	Gata5	1	0	0	1
rno-miR-873-5p	Gatad2a	1	0	0	1
rno-miR-873-5p	Gdi1	1	0	0	1
rno-miR-873-5p	Gdpd1_predict	0	1	0	1
rno-miR-873-5p	Gdpd2_predict	0	1	0	1
rno-miR-873-5p	Geft	1	0	0	1
rno-miR-873-5p	Gfra1	1	0	0	1
rno-miR-873-5p	Gga1	1	0	0	1
rno-miR-873-5p	Ggnbp2	1	0	0	1
rno-miR-873-5p	Ghrh	0	1	0	1
rno-miR-873-5p	Giot1	1	0	0	1
rno-miR-873-5p	Giyd2	0	1	0	1
rno-miR-873-5p	Gjb3	0	1	0	1
rno-miR-873-5p	Glud1	1	0	0	1
rno-miR-873-5p	Gm317	0	1	0	1
rno-miR-873-5p	Gmcl1	1	0	0	1
rno-miR-873-5p	Gmpr2	1	0	0	1
rno-miR-873-5p	Gnrhr	0	1	0	1
rno-miR-873-5p	Gns	1	0	0	1
rno-miR-873-5p	Golph3	1	0	0	1
rno-miR-873-5p	Golph3l	1	0	0	1
rno-miR-873-5p	Got1	1	0	0	1
rno-miR-873-5p	Gpr107_predic	0	1	0	1
rno-miR-873-5p	Gpr126	0	1	0	1
rno-miR-873-5p	Gpr139	0	1	0	1
rno-miR-873-5p	Gpr157	0	1	0	1
rno-miR-873-5p	Gpr31_predicte	0	1	0	1
rno-miR-873-5p	Gpr45_predicte	0	1	0	1
rno-miR-873-5p	Gpr83	1	0	0	1
rno-miR-873-5p	Gprc5c	0	0	1	1
rno-miR-873-5p	Gramd1a	0	1	0	1
rno-miR-873-5p	Grcc10	0	1	0	1
rno-miR-873-5p	Grinl1a	1	0	0	1
rno-miR-873-5p	Grm8	0	1	0	1
rno-miR-873-5p	Gsta5	1	0	0	1
rno-miR-873-5p	Gstm1	0	1	0	1
rno-miR-873-5p	Gtf2i	1	0	0	1

rno-miR-873-5p	Gtf2ird1	1	0	0	1
rno-miR-873-5p	Gzmb	1	0	0	1
rno-miR-873-5p	Gzmc	1	0	0	1
rno-miR-873-5p	Hadh	1	0	0	1
rno-miR-873-5p	Hadhb	0	1	0	1
rno-miR-873-5p	Havcr2	0	0	1	1
rno-miR-873-5p	Hbxap_predict	0	1	0	1
rno-miR-873-5p	Hdac1	1	0	0	1
rno-miR-873-5p	Hdhd2	1	0	0	1
rno-miR-873-5p	Helz_predicted	0	1	0	1
rno-miR-873-5p	Heph	1	0	0	1
rno-miR-873-5p	Hes2	1	0	0	1
rno-miR-873-5p	Hes6	1	0	0	1
rno-miR-873-5p	Hfe	1	0	0	1
rno-miR-873-5p	Hibch	1	0	0	1
rno-miR-873-5p	Hist2h2be	0	0	1	1
rno-miR-873-5p	Hivep2	1	0	0	1
rno-miR-873-5p	Hmgcr	1	0	0	1
rno-miR-873-5p	Hmgcs1	1	0	0	1
rno-miR-873-5p	Hn1	1	0	0	1
rno-miR-873-5p	Hn1l	1	0	0	1
rno-miR-873-5p	Hnrnpab	1	0	0	1
rno-miR-873-5p	Hnrnpc	1	0	0	1
rno-miR-873-5p	Hnrnpk	1	0	0	1
rno-miR-873-5p	Hnrpd	1	0	0	1
rno-miR-873-5p	Hnrpul1_predic	0	1	0	1
rno-miR-873-5p	Hopx	1	0	0	1
rno-miR-873-5p	Hoxa2	1	0	0	1
rno-miR-873-5p	Hoxd1_predict	0	1	0	1
rno-miR-873-5p	Hpca	1	0	0	1
rno-miR-873-5p	Hpgd	1	0	0	1
rno-miR-873-5p	Hrasls5	0	1	0	1
rno-miR-873-5p	Hrbl_predicted	0	1	0	1
rno-miR-873-5p	Hsd17b8	1	0	0	1
rno-miR-873-5p	Hspa12a_pred	0	1	0	1
rno-miR-873-5p	Hspa4l	0	0	1	1
rno-miR-873-5p	Hspb3	0	1	0	1
rno-miR-873-5p	Htr2c	1	0	0	1
rno-miR-873-5p	Htr6	1	0	0	1
rno-miR-873-5p	Hyal3	0	1	0	1
rno-miR-873-5p	Hyou1	1	0	0	1
rno-miR-873-5p	Ict1_predicted	0	1	0	1
rno-miR-873-5p	Ide	1	0	0	1
rno-miR-873-5p	Ift57	0	1	0	1
rno-miR-873-5p	Igsf1	1	0	0	1
rno-miR-873-5p	Igsf8	0	1	0	1
rno-miR-873-5p	Ikbkap	1	0	0	1
rno-miR-873-5p	Il13ra1	1	0	0	1
rno-miR-873-5p	Il1rn	1	0	0	1
rno-miR-873-5p	Il21r	1	0	0	1
rno-miR-873-5p	Il24	1	0	0	1

rno-miR-873-5p	Il33	1	0	0	1
rno-miR-873-5p	Il34	1	0	0	1
rno-miR-873-5p	Il9r	1	0	0	1
rno-miR-873-5p	Inhbe	1	0	0	1
rno-miR-873-5p	Ino80e	1	0	0	1
rno-miR-873-5p	Inpp1	1	0	0	1
rno-miR-873-5p	Inpp4a	0	1	0	1
rno-miR-873-5p	Inpp4b	1	0	0	1
rno-miR-873-5p	Insr	1	0	0	1
rno-miR-873-5p	Iqcb1_predicte	0	1	0	1
rno-miR-873-5p	Iqsec3	1	0	0	1
rno-miR-873-5p	Irf2bp1_predic	0	1	0	1
rno-miR-873-5p	Irs1	1	0	0	1
rno-miR-873-5p	Itgb6	1	0	0	1
rno-miR-873-5p	Itpkb	0	1	0	1
rno-miR-873-5p	Jag1	1	0	0	1
rno-miR-873-5p	Jak3	1	0	0	1
rno-miR-873-5p	Jmjd2a_predic	0	1	0	1
rno-miR-873-5p	Jmjd8	1	0	0	1
rno-miR-873-5p	Jun	1	0	0	1
rno-miR-873-5p	Kalrn	0	1	0	1
rno-miR-873-5p	Kars	0	1	0	1
rno-miR-873-5p	Kb4	0	1	0	1
rno-miR-873-5p	Kb40	0	1	0	1
rno-miR-873-5p	Kcna4	1	0	0	1
rno-miR-873-5p	Kcna5	0	1	0	1
rno-miR-873-5p	Kcnd2	1	0	0	1
rno-miR-873-5p	Kcne2	1	0	0	1
rno-miR-873-5p	Kcne4	0	1	0	1
rno-miR-873-5p	Kcnip2	1	0	0	1
rno-miR-873-5p	Kcnj11	1	0	0	1
rno-miR-873-5p	Kcnmb1	1	0	0	1
rno-miR-873-5p	Kcnmb2	0	1	0	1
rno-miR-873-5p	Kcns1	0	1	0	1
rno-miR-873-5p	Kctd4	0	0	1	1
rno-miR-873-5p	Kdelc2	1	0	0	1
rno-miR-873-5p	Kdelr1	1	0	0	1
rno-miR-873-5p	Keg1	1	0	0	1
rno-miR-873-5p	Khdrbs2	1	0	0	1
rno-miR-873-5p	Kif2c	0	1	0	1
rno-miR-873-5p	Kif3a	0	1	0	1
rno-miR-873-5p	Kif3b_predicte	0	1	0	1
rno-miR-873-5p	Klf4	1	0	0	1
rno-miR-873-5p	Klrc1	1	0	0	1
rno-miR-873-5p	Kpna1	1	0	0	1
rno-miR-873-5p	Krt1-4	0	1	0	1
rno-miR-873-5p	Krt15	1	0	0	1
rno-miR-873-5p	Ksr1_predicted	0	1	0	1
rno-miR-873-5p	Lass5_predicte	0	1	0	1
rno-miR-873-5p	Lck	0	1	0	1
rno-miR-873-5p	Lef1	0	1	0	1

rno-miR-873-5p	Leprot	1	0	0	1
rno-miR-873-5p	Lgals2	0	1	0	1
rno-miR-873-5p	Lgals3bp	0	1	0	1
rno-miR-873-5p	Limd1_predicte	0	1	0	1
rno-miR-873-5p	Lin7a	0	1	0	1
rno-miR-873-5p	Lipa	1	0	0	1
rno-miR-873-5p	Lix1l	0	0	1	1
rno-miR-873-5p	Lkap	1	0	0	1
rno-miR-873-5p	Lmbr1l	0	1	0	1
rno-miR-873-5p	LMO7	0	1	0	1
rno-miR-873-5p	Lmod3	0	0	1	1
rno-miR-873-5p	LOC10035951	0	0	1	1
rno-miR-873-5p	LOC10036272	0	0	1	1
rno-miR-873-5p	LOC10036464	0	0	1	1
rno-miR-873-5p	LOC297481	0	1	0	1
rno-miR-873-5p	LOC298116	0	1	0	1
rno-miR-873-5p	LOC298139	1	0	0	1
rno-miR-873-5p	LOC299920	0	1	0	1
rno-miR-873-5p	LOC300024	0	1	0	1
rno-miR-873-5p	LOC301165	0	0	1	1
rno-miR-873-5p	LOC302827	0	1	0	1
rno-miR-873-5p	LOC303448	1	0	0	1
rno-miR-873-5p	LOC305111	0	1	0	1
rno-miR-873-5p	LOC306766	0	0	1	1
rno-miR-873-5p	LOC311123	0	1	0	1
rno-miR-873-5p	LOC312102	0	1	0	1
rno-miR-873-5p	LOC313558	0	1	0	1
rno-miR-873-5p	LOC361237	0	1	0	1
rno-miR-873-5p	LOC361571	0	1	0	1
rno-miR-873-5p	LOC497933	0	1	0	1
rno-miR-873-5p	LOC497967	0	1	0	1
rno-miR-873-5p	LOC498145	1	0	0	1
rno-miR-873-5p	LOC498330	0	1	0	1
rno-miR-873-5p	LOC498350	1	0	0	1
rno-miR-873-5p	LOC498647	0	1	0	1
rno-miR-873-5p	LOC499300	0	1	0	1
rno-miR-873-5p	LOC499330	1	0	0	1
rno-miR-873-5p	LOC499331	1	0	0	1
rno-miR-873-5p	LOC499465	1	0	0	1
rno-miR-873-5p	LOC499653	0	1	0	1
rno-miR-873-5p	LOC499781	0	1	0	1
rno-miR-873-5p	LOC499886	1	0	0	1
rno-miR-873-5p	LOC499924	0	1	0	1
rno-miR-873-5p	LOC500118	1	0	0	1
rno-miR-873-5p	LOC500270	0	1	0	1
rno-miR-873-5p	LOC500319	0	1	0	1
rno-miR-873-5p	LOC500476	0	1	0	1
rno-miR-873-5p	LOC501296	0	1	0	1
rno-miR-873-5p	LOC501422	0	1	0	1
rno-miR-873-5p	LOC501617	0	1	0	1
rno-miR-873-5p	LOC595134	0	1	0	1

rno-miR-873-5p	LOC606294	1	0	0	1
rno-miR-873-5p	LOC678714	0	1	0	1
rno-miR-873-5p	LOC678768	0	1	0	1
rno-miR-873-5p	LOC679472	0	1	0	1
rno-miR-873-5p	LOC679552	0	1	0	1
rno-miR-873-5p	LOC679839	0	1	0	1
rno-miR-873-5p	LOC680367	0	1	0	1
rno-miR-873-5p	LOC680400	0	1	0	1
rno-miR-873-5p	LOC680542	0	1	0	1
rno-miR-873-5p	LOC680602	0	1	0	1
rno-miR-873-5p	LOC680812	0	1	0	1
rno-miR-873-5p	LOC680815	0	1	0	1
rno-miR-873-5p	LOC680991	0	1	0	1
rno-miR-873-5p	LOC681023	0	1	0	1
rno-miR-873-5p	LOC681300	0	1	0	1
rno-miR-873-5p	LOC681380	0	1	0	1
rno-miR-873-5p	LOC681787	0	1	0	1
rno-miR-873-5p	LOC682225	0	1	0	1
rno-miR-873-5p	LOC682558	0	1	0	1
rno-miR-873-5p	LOC682630	0	1	0	1
rno-miR-873-5p	LOC682859	0	1	0	1
rno-miR-873-5p	LOC682940	0	1	0	1
rno-miR-873-5p	LOC683050	0	1	0	1
rno-miR-873-5p	LOC683118	0	1	0	1
rno-miR-873-5p	LOC683842	0	1	0	1
rno-miR-873-5p	LOC684227	0	1	0	1
rno-miR-873-5p	LOC684233	0	0	1	1
rno-miR-873-5p	LOC684822	0	1	0	1
rno-miR-873-5p	LOC685197	0	1	0	1
rno-miR-873-5p	LOC685271	0	1	0	1
rno-miR-873-5p	LOC685463	0	1	0	1
rno-miR-873-5p	LOC685557	0	1	0	1
rno-miR-873-5p	LOC686055	0	1	0	1
rno-miR-873-5p	LOC686442	0	1	0	1
rno-miR-873-5p	LOC686671	0	1	0	1
rno-miR-873-5p	LOC686931	0	1	0	1
rno-miR-873-5p	LOC686988	0	1	0	1
rno-miR-873-5p	LOC687049	0	1	0	1
rno-miR-873-5p	LOC687145	0	0	1	1
rno-miR-873-5p	LOC687398	0	1	0	1
rno-miR-873-5p	LOC687912	0	1	0	1
rno-miR-873-5p	LOC688252	0	1	0	1
rno-miR-873-5p	LOC688531	0	1	0	1
rno-miR-873-5p	LOC688599	0	1	0	1
rno-miR-873-5p	LOC688811	0	1	0	1
rno-miR-873-5p	LOC689524	0	1	0	1
rno-miR-873-5p	LOC690211	0	1	0	1
rno-miR-873-5p	LOC690428	0	1	0	1
rno-miR-873-5p	LOC691222	0	1	0	1
rno-miR-873-5p	LOC691223	0	1	0	1
rno-miR-873-5p	LOC691616	0	1	0	1

rno-miR-873-5p	LOC691981	0	1	0	1
rno-miR-873-5p	Lrba_predicted	0	1	0	1
rno-miR-873-5p	Lrg1	1	0	0	1
rno-miR-873-5p	Lrrc14	1	0	0	1
rno-miR-873-5p	Lrrc18	1	0	0	1
rno-miR-873-5p	Lrrc2	1	0	0	1
rno-miR-873-5p	Lrrc30_predict	0	1	0	1
rno-miR-873-5p	Lrrc3b_predict	0	1	0	1
rno-miR-873-5p	Lrrc4	1	0	0	1
rno-miR-873-5p	Lrrc56	0	1	0	1
rno-miR-873-5p	Lrrc59	1	0	0	1
rno-miR-873-5p	Lrrc8d	1	0	0	1
rno-miR-873-5p	Lrrc8e	0	1	0	1
rno-miR-873-5p	Lsg1	1	0	0	1
rno-miR-873-5p	Ltb	1	0	0	1
rno-miR-873-5p	Ly49i4	1	0	0	1
rno-miR-873-5p	Ly49i5	1	0	0	1
rno-miR-873-5p	Ly49s3	1	0	0	1
rno-miR-873-5p	Ly49s6	1	0	0	1
rno-miR-873-5p	Ly6h_predicted	0	1	0	1
rno-miR-873-5p	Lyar	1	0	0	1
rno-miR-873-5p	Lyn	1	0	0	1
rno-miR-873-5p	Mad2l1_predic	0	1	0	1
rno-miR-873-5p	Mafb	1	0	0	1
rno-miR-873-5p	Mafk	1	0	0	1
rno-miR-873-5p	Mageh1	1	0	0	1
rno-miR-873-5p	Magi2	0	1	0	1
rno-miR-873-5p	Map3k12	1	0	0	1
rno-miR-873-5p	Map3k14_pred	0	1	0	1
rno-miR-873-5p	Map4k3	0	0	1	1
rno-miR-873-5p	Mapk1	0	1	0	1
rno-miR-873-5p	Mapk11	0	1	0	1
rno-miR-873-5p	Mapk6	1	0	0	1
rno-miR-873-5p	Mapk9	1	0	0	1
rno-miR-873-5p	Mare	1	0	0	1
rno-miR-873-5p	Matn1	1	0	0	1
rno-miR-873-5p	Mb	1	0	0	1
rno-miR-873-5p	Mdk	0	1	0	1
rno-miR-873-5p	Med29	0	0	1	1
rno-miR-873-5p	Meox2	1	0	0	1
rno-miR-873-5p	Metap2	1	0	0	1
rno-miR-873-5p	Mettl7a	1	0	0	1
rno-miR-873-5p	Mga	0	1	0	1
rno-miR-873-5p	Mgat1	1	0	0	1
rno-miR-873-5p	MGC105560	1	0	0	1
rno-miR-873-5p	MGC112682	0	1	0	1
rno-miR-873-5p	MGC112830	1	0	0	1
rno-miR-873-5p	MGC114483	1	0	0	1
rno-miR-873-5p	MGC114520	1	0	0	1
rno-miR-873-5p	MGC156825	0	1	0	1
rno-miR-873-5p	MGC93975	1	0	0	1

rno-miR-873-5p	MGC94142	0	1	0	1
rno-miR-873-5p	MGC94335	0	1	0	1
rno-miR-873-5p	MGC94954	0	1	0	1
rno-miR-873-5p	MGC95152	1	0	0	1
rno-miR-873-5p	MGC95210	1	0	0	1
rno-miR-873-5p	Mgmt	1	0	0	1
rno-miR-873-5p	Mia	1	0	0	1
rno-miR-873-5p	Mia1	0	1	0	1
rno-miR-873-5p	Mkln1	1	0	0	1
rno-miR-873-5p	Mllt11	1	0	0	1
rno-miR-873-5p	Mlx	0	1	0	1
rno-miR-873-5p	Mmp12	0	1	0	1
rno-miR-873-5p	Mmp16	0	1	0	1
rno-miR-873-5p	Mmp7	0	1	0	1
rno-miR-873-5p	Morf4l2	1	0	0	1
rno-miR-873-5p	Mos	1	0	0	1
rno-miR-873-5p	Mpg	0	1	0	1
rno-miR-873-5p	mrpl11	0	1	0	1
rno-miR-873-5p	Mrpl16	0	1	0	1
rno-miR-873-5p	Mrpl2	1	0	0	1
rno-miR-873-5p	Mrpl21_predict	0	1	0	1
rno-miR-873-5p	Mrpl36_predict	0	1	0	1
rno-miR-873-5p	Mrpl38	1	0	0	1
rno-miR-873-5p	Mrps18b	0	1	0	1
rno-miR-873-5p	Mrps18c_pred	0	1	0	1
rno-miR-873-5p	Mrps23_predic	0	1	0	1
rno-miR-873-5p	Mrps26	1	0	0	1
rno-miR-873-5p	Ms4a10_predi	0	1	0	1
rno-miR-873-5p	Msl3	1	0	0	1
rno-miR-873-5p	Msrb2	1	0	0	1
rno-miR-873-5p	Mtf1_predicted	0	1	0	1
rno-miR-873-5p	Mtvr2	1	0	0	1
rno-miR-873-5p	Mum1_predict	0	1	0	1
rno-miR-873-5p	Myo9b	0	1	0	1
rno-miR-873-5p	Myt1_predicted	0	1	0	1
rno-miR-873-5p	N-pac	1	0	0	1
rno-miR-873-5p	Naa15	0	0	1	1
rno-miR-873-5p	Naaa	1	0	0	1
rno-miR-873-5p	Nap1l2	1	0	0	1
rno-miR-873-5p	Narfl	1	0	0	1
rno-miR-873-5p	Narg1_predicte	0	1	0	1
rno-miR-873-5p	Nbl1	1	0	0	1
rno-miR-873-5p	Ncf1	1	0	0	1
rno-miR-873-5p	Ncr1	1	0	0	1
rno-miR-873-5p	Ndfip1	1	0	0	1
rno-miR-873-5p	Ndufa1_predic	0	1	0	1
rno-miR-873-5p	Ndufaf4	1	0	0	1
rno-miR-873-5p	Ndufb5_predic	0	1	0	1
rno-miR-873-5p	Ndufv2	0	1	0	1
rno-miR-873-5p	Neil3_predicte	0	1	0	1
rno-miR-873-5p	Nek7_predicte	0	1	0	1

rno-miR-873-5p	Nelf	1	0	0	1
rno-miR-873-5p	Nell2	1	0	0	1
rno-miR-873-5p	Neto1	0	0	1	1
rno-miR-873-5p	Neurog3	0	1	0	1
rno-miR-873-5p	Nfatc2_predict	0	1	0	1
rno-miR-873-5p	Nfya	1	0	0	1
rno-miR-873-5p	Nfyb	1	0	0	1
rno-miR-873-5p	Ng35	0	1	0	1
rno-miR-873-5p	Ngrn	1	0	0	1
rno-miR-873-5p	Nip30	1	0	0	1
rno-miR-873-5p	Nkx6-1	1	0	0	1
rno-miR-873-5p	Nmb_predicted	0	1	0	1
rno-miR-873-5p	Nmnat3	1	0	0	1
rno-miR-873-5p	NP_00101412	0	1	0	1
rno-miR-873-5p	NP_00102907	0	1	0	1
rno-miR-873-5p	NP_00107425	0	1	0	1
rno-miR-873-5p	NP_00107491	0	1	0	1
rno-miR-873-5p	NP_00107709	0	1	0	1
rno-miR-873-5p	Npepps	0	1	0	1
rno-miR-873-5p	Nppb	1	0	0	1
rno-miR-873-5p	Npr1	1	0	0	1
rno-miR-873-5p	Nptn	1	0	0	1
rno-miR-873-5p	Nptx1	1	0	0	1
rno-miR-873-5p	Nr1d1	1	0	0	1
rno-miR-873-5p	Nr1i2	1	0	0	1
rno-miR-873-5p	Nr2f1	1	0	0	1
rno-miR-873-5p	Nr4a1	1	0	0	1
rno-miR-873-5p	Nr4a2	1	0	0	1
rno-miR-873-5p	Nr4a3	1	0	0	1
rno-miR-873-5p	Nrcam	1	0	0	1
rno-miR-873-5p	Nsfl1c	1	0	0	1
rno-miR-873-5p	Nsg1	1	0	0	1
rno-miR-873-5p	Nt5dc1_predic	0	1	0	1
rno-miR-873-5p	Nub1	1	0	0	1
rno-miR-873-5p	Nufip2	0	1	0	1
rno-miR-873-5p	null	0	1	0	1
rno-miR-873-5p	Nutf2	1	0	0	1
rno-miR-873-5p	Nyx_predicted	0	1	0	1
rno-miR-873-5p	O08661_RAT	0	1	0	1
rno-miR-873-5p	O88596_RAT	0	1	0	1
rno-miR-873-5p	O89037_RAT	0	1	0	1
rno-miR-873-5p	Oasl1	0	1	0	1
rno-miR-873-5p	Obp3	0	1	0	1
rno-miR-873-5p	Olfr1102	0	1	0	1
rno-miR-873-5p	Olr1095_predi	0	1	0	1
rno-miR-873-5p	Olr1121_predi	0	1	0	1
rno-miR-873-5p	Olr1213_predi	0	1	0	1
rno-miR-873-5p	Olr1437_predi	0	1	0	1
rno-miR-873-5p	Olr1528_predi	0	1	0	1
rno-miR-873-5p	Olr154_predict	0	1	0	1
rno-miR-873-5p	Olr1585_predi	0	1	0	1

rno-miR-873-5p	Olr1610_predi	0	1	0	1
rno-miR-873-5p	Olr1629_predi	0	1	0	1
rno-miR-873-5p	Olr1684_predi	0	1	0	1
rno-miR-873-5p	Olr1750_predi	0	1	0	1
rno-miR-873-5p	Olr234_predict	0	1	0	1
rno-miR-873-5p	Olr295_predict	0	1	0	1
rno-miR-873-5p	Olr311_predict	0	1	0	1
rno-miR-873-5p	Olr312_predict	0	1	0	1
rno-miR-873-5p	Olr313_predict	0	1	0	1
rno-miR-873-5p	Olr315_predict	0	1	0	1
rno-miR-873-5p	Olr357_predict	0	1	0	1
rno-miR-873-5p	Olr367_predict	0	1	0	1
rno-miR-873-5p	Olr419_predict	0	1	0	1
rno-miR-873-5p	Olr427_predict	0	1	0	1
rno-miR-873-5p	Olr458_predict	0	1	0	1
rno-miR-873-5p	Olr47_predicte	0	1	0	1
rno-miR-873-5p	Olr57_predicte	0	1	0	1
rno-miR-873-5p	Olr726_predict	0	1	0	1
rno-miR-873-5p	Olr749_predict	0	1	0	1
rno-miR-873-5p	Olr760_predict	0	1	0	1
rno-miR-873-5p	Olr804_predict	0	1	0	1
rno-miR-873-5p	Olr812_predict	0	1	0	1
rno-miR-873-5p	Omg	1	0	0	1
rno-miR-873-5p	Opalin	1	0	0	1
rno-miR-873-5p	Oprk1	1	0	0	1
rno-miR-873-5p	Oprl1	0	1	0	1
rno-miR-873-5p	Oprs1	0	1	0	1
rno-miR-873-5p	Orai3	1	0	0	1
rno-miR-873-5p	Ormdl2_predic	0	1	0	1
rno-miR-873-5p	Osbpl6_predic	0	1	0	1
rno-miR-873-5p	Ostf1	1	0	0	1
rno-miR-873-5p	P2rx4	1	0	0	1
rno-miR-873-5p	P2rx5	1	0	0	1
rno-miR-873-5p	P2rx7	1	0	0	1
rno-miR-873-5p	P4ha3	0	1	0	1
rno-miR-873-5p	Padi2	1	0	0	1
rno-miR-873-5p	Pafah1b2	1	0	0	1
rno-miR-873-5p	Pah	1	0	0	1
rno-miR-873-5p	Panx3	1	0	0	1
rno-miR-873-5p	Papln_predicte	0	1	0	1
rno-miR-873-5p	Pbsn	1	0	0	1
rno-miR-873-5p	Pbx1_predicte	0	1	0	1
rno-miR-873-5p	Pcbd1	1	0	0	1
rno-miR-873-5p	Pcdha1	1	0	0	1
rno-miR-873-5p	Pcdha10	1	0	0	1
rno-miR-873-5p	Pcdha11	1	0	0	1
rno-miR-873-5p	Pcdha12	1	0	0	1
rno-miR-873-5p	Pcdha13	1	0	0	1
rno-miR-873-5p	Pcdha2	1	0	0	1
rno-miR-873-5p	Pcdha3	1	0	0	1
rno-miR-873-5p	Pcdha4	1	0	0	1

rno-miR-873-5p	Pcdha5	1	0	0	1
rno-miR-873-5p	Pcdha6	1	0	0	1
rno-miR-873-5p	Pcdha7	1	0	0	1
rno-miR-873-5p	Pcdha8	1	0	0	1
rno-miR-873-5p	Pcdhac1	1	0	0	1
rno-miR-873-5p	Pcdhac2	1	0	0	1
rno-miR-873-5p	Pcdhb21	0	0	1	1
rno-miR-873-5p	Pcdhb22	0	1	0	1
rno-miR-873-5p	Pcdhb9	0	0	1	1
rno-miR-873-5p	Pcp4	0	1	0	1
rno-miR-873-5p	Pcsk1	1	0	0	1
rno-miR-873-5p	Pcsk5	0	1	0	1
rno-miR-873-5p	Pcsk9	1	0	0	1
rno-miR-873-5p	Pdpk1	0	1	0	1
rno-miR-873-5p	Pdpn	0	1	0	1
rno-miR-873-5p	Pdyn	1	0	0	1
rno-miR-873-5p	Pef1	1	0	0	1
rno-miR-873-5p	Per2	1	0	0	1
rno-miR-873-5p	Pfkfb1	1	0	0	1
rno-miR-873-5p	Pfn4	1	0	0	1
rno-miR-873-5p	Pgk1	1	0	0	1
rno-miR-873-5p	Pgpep1	1	0	0	1
rno-miR-873-5p	Phf5a	0	1	0	1
rno-miR-873-5p	Phf7	0	1	0	1
rno-miR-873-5p	Phlda3	1	0	0	1
rno-miR-873-5p	Phyhip	1	0	0	1
rno-miR-873-5p	Pigm	1	0	0	1
rno-miR-873-5p	Pir	1	0	0	1
rno-miR-873-5p	Pitpnb	1	0	0	1
rno-miR-873-5p	Pja2	1	0	0	1
rno-miR-873-5p	Pkib	1	0	0	1
rno-miR-873-5p	Pkn1	0	1	0	1
rno-miR-873-5p	Pla2g2a	0	1	0	1
rno-miR-873-5p	Pla2g2c	1	0	0	1
rno-miR-873-5p	Plcb2	0	1	0	1
rno-miR-873-5p	Plekhb1	1	0	0	1
rno-miR-873-5p	Plekhb2_predi	0	1	0	1
rno-miR-873-5p	Plk2	1	0	0	1
rno-miR-873-5p	Plod1	0	1	0	1
rno-miR-873-5p	Plxnb3_predict	0	1	0	1
rno-miR-873-5p	Pmm2_predict	0	1	0	1
rno-miR-873-5p	Pnldc1	1	0	0	1
rno-miR-873-5p	Pofut2_predict	0	1	0	1
rno-miR-873-5p	Polb	1	0	0	1
rno-miR-873-5p	Polr2a	0	1	0	1
rno-miR-873-5p	Polr2c	0	1	0	1
rno-miR-873-5p	Pomc	1	0	0	1
rno-miR-873-5p	Por	0	1	0	1
rno-miR-873-5p	Pou2f1	0	1	0	1
rno-miR-873-5p	Pparg	1	0	0	1
rno-miR-873-5p	Ppie	0	1	0	1

rno-miR-873-5p	Ppil4_predicte	0	1	0	1
rno-miR-873-5p	Ppm1b	1	0	0	1
rno-miR-873-5p	Ppm1k_predic	0	1	0	1
rno-miR-873-5p	Ppp1r1b	1	0	0	1
rno-miR-873-5p	Ppp1r2	1	0	0	1
rno-miR-873-5p	Ppp1r3c	1	0	0	1
rno-miR-873-5p	Ppp2cb	1	0	0	1
rno-miR-873-5p	Ppp2r1b	1	0	0	1
rno-miR-873-5p	Ppp2r4	0	0	1	1
rno-miR-873-5p	Ppp2r4_predic	0	1	0	1
rno-miR-873-5p	Ppp3ca	1	0	0	1
rno-miR-873-5p	PREB_RAT	0	1	0	1
rno-miR-873-5p	Prickle2	0	0	1	1
rno-miR-873-5p	Prkab1	1	0	0	1
rno-miR-873-5p	Prkcd	1	0	0	1
rno-miR-873-5p	Prkci	0	1	0	1
rno-miR-873-5p	Prkra	1	0	0	1
rno-miR-873-5p	Prkx	1	0	0	1
rno-miR-873-5p	Prl8a7	1	0	0	1
rno-miR-873-5p	Prpf19	0	1	0	1
rno-miR-873-5p	Prps1	1	0	0	1
rno-miR-873-5p	Psap	0	1	0	1
rno-miR-873-5p	Psat1	1	0	0	1
rno-miR-873-5p	Psd	0	1	0	1
rno-miR-873-5p	Psg29	1	0	0	1
rno-miR-873-5p	Psme1	0	1	0	1
rno-miR-873-5p	Pspc1	1	0	0	1
rno-miR-873-5p	Pter	1	0	0	1
rno-miR-873-5p	Ptges	0	1	0	1
rno-miR-873-5p	Ptges3l1	1	0	0	1
rno-miR-873-5p	Ptma	1	0	0	1
rno-miR-873-5p	Ptp4a3_predic	0	1	0	1
rno-miR-873-5p	Ptpn7	1	0	0	1
rno-miR-873-5p	Ptprc	0	1	0	1
rno-miR-873-5p	Ptprq	0	1	0	1
rno-miR-873-5p	Pttg1ip	1	0	0	1
rno-miR-873-5p	Pwp1_predicte	0	1	0	1
rno-miR-873-5p	Pycard	0	1	0	1
rno-miR-873-5p	Q38PG1_RAT	0	1	0	1
rno-miR-873-5p	Q3ZAV2_RAT	0	1	0	1
rno-miR-873-5p	Q45N69_RAT	0	1	0	1
rno-miR-873-5p	Q499U7_RAT	0	1	0	1
rno-miR-873-5p	Q4G039_RAT	0	1	0	1
rno-miR-873-5p	Q4KLH1_RAT	0	1	0	1
rno-miR-873-5p	Q5BJR9_RAT	0	1	0	1
rno-miR-873-5p	Q5BJV4_RAT	0	1	0	1
rno-miR-873-5p	Q5BKB3_RAT	0	1	0	1
rno-miR-873-5p	Q5M928_RAT	0	1	0	1
rno-miR-873-5p	Q5XFW6_RAT	0	1	0	1
rno-miR-873-5p	Q5XID9_RAT	0	1	0	1
rno-miR-873-5p	Q63773_RAT	0	1	0	1

rno-miR-873-5p	Q80VL0_RAT	0	1	0	1
rno-miR-873-5p	Q9EPS1_RAT	0	1	0	1
rno-miR-873-5p	Qsox1	1	0	0	1
rno-miR-873-5p	Rab1	1	0	0	1
rno-miR-873-5p	Rab18	1	0	0	1
rno-miR-873-5p	Rab26	0	1	0	1
rno-miR-873-5p	Rab2l	0	1	0	1
rno-miR-873-5p	Rab33b_predic	0	1	0	1
rno-miR-873-5p	Rab35	1	0	0	1
rno-miR-873-5p	Rab38	1	0	0	1
rno-miR-873-5p	Rab3a	1	0	0	1
rno-miR-873-5p	Rab5a	1	0	0	1
rno-miR-873-5p	Rabl2b	1	0	0	1
rno-miR-873-5p	Rad17	1	0	0	1
rno-miR-873-5p	Raf1	1	0	0	1
rno-miR-873-5p	Rai14	1	0	0	1
rno-miR-873-5p	Ran	1	0	0	1
rno-miR-873-5p	Rasgrf1	0	1	0	1
rno-miR-873-5p	Rassf4	1	0	0	1
rno-miR-873-5p	Rassf6	1	0	0	1
rno-miR-873-5p	Rbbp9	1	0	0	1
rno-miR-873-5p	Rbm16	1	0	0	1
rno-miR-873-5p	Rbm34	1	0	0	1
rno-miR-873-5p	Rbm4b	1	0	0	1
rno-miR-873-5p	Rbms1	0	1	0	1
rno-miR-873-5p	Rbmx	1	0	0	1
rno-miR-873-5p	Rbp1	0	1	0	1
rno-miR-873-5p	Rbx1	1	0	0	1
rno-miR-873-5p	Rcan1	1	0	0	1
rno-miR-873-5p	Recql4_predic	0	1	0	1
rno-miR-873-5p	Rell2	1	0	0	1
rno-miR-873-5p	Ren1	0	1	0	1
rno-miR-873-5p	Retnlb	1	0	0	1
rno-miR-873-5p	Rfc2	1	0	0	1
rno-miR-873-5p	Rfk	1	0	0	1
rno-miR-873-5p	Rg9mtd3	1	0	0	1
rno-miR-873-5p	RGD1304644	1	0	0	1
rno-miR-873-5p	RGD1304688_	0	1	0	1
rno-miR-873-5p	RGD1304902_	0	1	0	1
rno-miR-873-5p	RGD1304952	1	0	0	1
rno-miR-873-5p	RGD1304999	0	1	0	1
rno-miR-873-5p	RGD1305038_	0	1	0	1
rno-miR-873-5p	RGD1305077_	0	1	0	1
rno-miR-873-5p	RGD1305162_	0	1	0	1
rno-miR-873-5p	RGD1305455	0	0	1	1
rno-miR-873-5p	RGD1305801_	0	1	0	1
rno-miR-873-5p	RGD1305846_	0	1	0	1
rno-miR-873-5p	RGD1305899_	0	1	0	1
rno-miR-873-5p	RGD1306164_	0	1	0	1
rno-miR-873-5p	RGD1306227	0	1	0	1
rno-miR-873-5p	RGD1306371_	0	1	0	1

rno-miR-873-5p	RGD1306538	0	1	0	1
rno-miR-873-5p	RGD1306583	1	0	0	1
rno-miR-873-5p	RGD1306674_	0	1	0	1
rno-miR-873-5p	RGD1306880	0	0	1	1
rno-miR-873-5p	RGD1306908	0	1	0	1
rno-miR-873-5p	RGD1306926_	0	1	0	1
rno-miR-873-5p	RGD1306938_	0	1	0	1
rno-miR-873-5p	RGD1306939	0	1	0	1
rno-miR-873-5p	RGD1307047	0	1	0	1
rno-miR-873-5p	RGD1307222_	0	1	0	1
rno-miR-873-5p	RGD1307284_	0	1	0	1
rno-miR-873-5p	RGD1307414_	0	1	0	1
rno-miR-873-5p	RGD1307700	0	1	0	1
rno-miR-873-5p	RGD1307830_	0	1	0	1
rno-miR-873-5p	RGD1308665	0	1	0	1
rno-miR-873-5p	RGD1308759_	0	1	0	1
rno-miR-873-5p	RGD1308775	0	0	1	1
rno-miR-873-5p	RGD1308775_	0	1	0	1
rno-miR-873-5p	RGD1308836	0	1	0	1
rno-miR-873-5p	RGD1308907	0	0	1	1
rno-miR-873-5p	RGD1309065_	0	1	0	1
rno-miR-873-5p	RGD1309077_	0	1	0	1
rno-miR-873-5p	RGD1309362	1	0	0	1
rno-miR-873-5p	RGD1309443_	0	1	0	1
rno-miR-873-5p	RGD1309452_	0	1	0	1
rno-miR-873-5p	RGD1309546_	0	1	0	1
rno-miR-873-5p	RGD1309892	0	1	0	1
rno-miR-873-5p	RGD1310061	0	1	0	1
rno-miR-873-5p	RGD1310316	0	1	0	1
rno-miR-873-5p	RGD1310405	0	1	0	1
rno-miR-873-5p	RGD1310450	0	1	0	1
rno-miR-873-5p	RGD1310553	1	0	0	1
rno-miR-873-5p	RGD1310571	1	0	0	1
rno-miR-873-5p	RGD1310686	1	0	0	1
rno-miR-873-5p	RGD1310710_	0	1	0	1
rno-miR-873-5p	RGD1310722_	0	1	0	1
rno-miR-873-5p	RGD1310736_	0	1	0	1
rno-miR-873-5p	RGD1310868_	0	1	0	1
rno-miR-873-5p	RGD1311053_	0	1	0	1
rno-miR-873-5p	RGD1311154_	0	1	0	1
rno-miR-873-5p	RGD1311234_	0	1	0	1
rno-miR-873-5p	RGD1311300	0	1	0	1
rno-miR-873-5p	RGD1311481_	0	1	0	1
rno-miR-873-5p	RGD1311634_	0	1	0	1
rno-miR-873-5p	RGD1311662_	0	1	0	1
rno-miR-873-5p	RGD1311703	1	0	0	1
rno-miR-873-5p	RGD1311914	0	1	0	1
rno-miR-873-5p	RGD1312003	0	1	0	1
rno-miR-873-5p	RGD1359310	0	1	0	1
rno-miR-873-5p	RGD1559561_	0	1	0	1
rno-miR-873-5p	RGD1559628_	0	1	0	1

rno-miR-873-5p	RGD1559651_	0	1	0	1
rno-miR-873-5p	RGD1559751_	0	1	0	1
rno-miR-873-5p	RGD1560011_	0	1	0	1
rno-miR-873-5p	RGD1560538_	0	1	0	1
rno-miR-873-5p	RGD1560638_	0	1	0	1
rno-miR-873-5p	RGD1560849_	0	1	0	1
rno-miR-873-5p	RGD1560871_	0	1	0	1
rno-miR-873-5p	RGD1560880_	0	1	0	1
rno-miR-873-5p	RGD1560891_	0	1	0	1
rno-miR-873-5p	RGD1561176_	0	1	0	1
rno-miR-873-5p	RGD1561211_	0	1	0	1
rno-miR-873-5p	RGD1561338_	0	1	0	1
rno-miR-873-5p	RGD1561420_	0	1	0	1
rno-miR-873-5p	RGD1561492_	0	1	0	1
rno-miR-873-5p	RGD1561781_	0	1	0	1
rno-miR-873-5p	RGD1561808_	0	1	0	1
rno-miR-873-5p	RGD1561852_	0	1	0	1
rno-miR-873-5p	RGD1561926_	0	1	0	1
rno-miR-873-5p	RGD1561956_	0	1	0	1
rno-miR-873-5p	RGD1562232_	0	1	0	1
rno-miR-873-5p	RGD1562312_	0	1	0	1
rno-miR-873-5p	RGD1562527_	0	1	0	1
rno-miR-873-5p	RGD1562580_	0	1	0	1
rno-miR-873-5p	RGD1562652_	0	1	0	1
rno-miR-873-5p	RGD1562693_	0	1	0	1
rno-miR-873-5p	RGD1562705_	0	1	0	1
rno-miR-873-5p	RGD1562767_	0	1	0	1
rno-miR-873-5p	RGD1562793_	0	1	0	1
rno-miR-873-5p	RGD1563028_	0	1	0	1
rno-miR-873-5p	RGD1563072_	0	1	0	1
rno-miR-873-5p	RGD1563104_	0	1	0	1
rno-miR-873-5p	RGD1563142_	0	1	0	1
rno-miR-873-5p	RGD1563673_	0	1	0	1
rno-miR-873-5p	RGD1563680_	0	1	0	1
rno-miR-873-5p	RGD1563853_	0	1	0	1
rno-miR-873-5p	RGD1564010_	0	1	0	1
rno-miR-873-5p	RGD1564013_	0	1	0	1
rno-miR-873-5p	RGD1564091_	0	1	0	1
rno-miR-873-5p	RGD1564103_	0	1	0	1
rno-miR-873-5p	RGD1564164_	0	1	0	1
rno-miR-873-5p	RGD1564595_	0	1	0	1
rno-miR-873-5p	RGD1564975_	0	1	0	1
rno-miR-873-5p	RGD1565016_	0	1	0	1
rno-miR-873-5p	RGD1565031_	0	1	0	1
rno-miR-873-5p	RGD1565037_	0	1	0	1
rno-miR-873-5p	RGD1565088_	0	1	0	1
rno-miR-873-5p	RGD1565120_	0	1	0	1
rno-miR-873-5p	RGD1565192_	0	1	0	1
rno-miR-873-5p	RGD1565406_	0	1	0	1
rno-miR-873-5p	RGD1565542_	0	1	0	1
rno-miR-873-5p	RGD1565601_	0	1	0	1

rno-miR-873-5p	RGD1565710_	0	1	0	1
rno-miR-873-5p	RGD1566001_	0	1	0	1
rno-miR-873-5p	RGD1566036_	0	1	0	1
rno-miR-873-5p	RGD1566076_	0	1	0	1
rno-miR-873-5p	RGD1566153_	0	1	0	1
rno-miR-873-5p	RGD1566260_	0	1	0	1
rno-miR-873-5p	RGD69425	1	0	0	1
rno-miR-873-5p	RGD727788	0	1	0	1
rno-miR-873-5p	Rgs11	0	1	0	1
rno-miR-873-5p	Rgs12	1	0	0	1
rno-miR-873-5p	Rgs17	0	0	1	1
rno-miR-873-5p	Rgs17_predict	0	1	0	1
rno-miR-873-5p	Rgs7	1	0	0	1
rno-miR-873-5p	Rhbdl6_predic	0	1	0	1
rno-miR-873-5p	Rhcg	1	0	0	1
rno-miR-873-5p	Rhod_predicte	0	1	0	1
rno-miR-873-5p	Rilpl2	1	0	0	1
rno-miR-873-5p	Rims1	0	1	0	1
rno-miR-873-5p	Rint1	0	1	0	1
rno-miR-873-5p	Ripk3	0	1	0	1
rno-miR-873-5p	Ripk5	1	0	0	1
rno-miR-873-5p	Ris2_predicted	0	1	0	1
rno-miR-873-5p	Rit2	1	0	0	1
rno-miR-873-5p	RL30_RAT	0	1	0	1
rno-miR-873-5p	Rlf_predicted	0	1	0	1
rno-miR-873-5p	Rmnd5b	1	0	0	1
rno-miR-873-5p	Rnf181	1	0	0	1
rno-miR-873-5p	Rnf217	0	0	1	1
rno-miR-873-5p	Rnf38	1	0	0	1
rno-miR-873-5p	Rnf6_predicted	0	1	0	1
rno-miR-873-5p	Robo2	0	0	1	1
rno-miR-873-5p	Rod1	1	0	0	1
rno-miR-873-5p	Ror2_predicted	0	1	0	1
rno-miR-873-5p	Ros1	1	0	0	1
rno-miR-873-5p	Rpa2	1	0	0	1
rno-miR-873-5p	Rpl5	0	1	0	1
rno-miR-873-5p	Rrad	1	0	0	1
rno-miR-873-5p	RS29_RAT	0	1	0	1
rno-miR-873-5p	Rsrc2	1	0	0	1
rno-miR-873-5p	RT1-A2	1	0	0	1
rno-miR-873-5p	RT1-T24-4	1	0	0	1
rno-miR-873-5p	Runx1	0	1	0	1
rno-miR-873-5p	Rwdd2_predict	0	1	0	1
rno-miR-873-5p	S1pr1	1	0	0	1
rno-miR-873-5p	Safb	0	0	1	1
rno-miR-873-5p	Samd3	0	1	0	1
rno-miR-873-5p	Samd8	0	1	0	1
rno-miR-873-5p	Sap1	0	1	0	1
rno-miR-873-5p	Sar1a	1	0	0	1
rno-miR-873-5p	Sass6_predict	0	1	0	1
rno-miR-873-5p	Scarb2	1	0	0	1

rno-miR-873-5p	Sclt1	1	0	0	1
rno-miR-873-5p	Scly	1	0	0	1
rno-miR-873-5p	Scyl2_predicte	0	1	0	1
rno-miR-873-5p	Sdfr1	0	1	0	1
rno-miR-873-5p	Sdk2	0	1	0	1
rno-miR-873-5p	Sec11c	1	0	0	1
rno-miR-873-5p	Sec14l4	0	0	1	1
rno-miR-873-5p	Sec24a_predic	0	1	0	1
rno-miR-873-5p	Sectm1a	1	0	0	1
rno-miR-873-5p	Sele	1	0	0	1
rno-miR-873-5p	Sema5a_predi	0	1	0	1
rno-miR-873-5p	Senp8	1	0	0	1
rno-miR-873-5p	Sept9	1	0	0	1
rno-miR-873-5p	Sepw1	0	1	0	1
rno-miR-873-5p	Serbp1	1	0	0	1
rno-miR-873-5p	Serpina3k	0	1	0	1
rno-miR-873-5p	Serpina3m	0	1	0	1
rno-miR-873-5p	Sez6l2_predict	0	1	0	1
rno-miR-873-5p	Sfrp4	1	0	0	1
rno-miR-873-5p	Sfrs8	0	1	0	1
rno-miR-873-5p	Sgk1	1	0	0	1
rno-miR-873-5p	Sgms1	1	0	0	1
rno-miR-873-5p	Sh2d2a	1	0	0	1
rno-miR-873-5p	SH3G2_RAT	0	1	0	1
rno-miR-873-5p	Shank3	0	1	0	1
rno-miR-873-5p	Siae	0	1	0	1
rno-miR-873-5p	Sigirr	1	0	0	1
rno-miR-873-5p	Sigmar1	1	0	0	1
rno-miR-873-5p	Slain2	0	0	1	1
rno-miR-873-5p	Slc12a1	1	0	0	1
rno-miR-873-5p	Slc12a7	0	1	0	1
rno-miR-873-5p	Slc15a2	1	0	0	1
rno-miR-873-5p	Slc17a3	0	0	1	1
rno-miR-873-5p	Slc17a6	1	0	0	1
rno-miR-873-5p	Slc22a17	0	1	0	1
rno-miR-873-5p	Slc23a2	0	1	0	1
rno-miR-873-5p	Slc25a14	1	0	0	1
rno-miR-873-5p	Slc25a15	0	1	0	1
rno-miR-873-5p	Slc25a41	0	0	1	1
rno-miR-873-5p	Slc26a4	1	0	0	1
rno-miR-873-5p	Slc35e1	0	0	1	1
rno-miR-873-5p	Slc38a2	1	0	0	1
rno-miR-873-5p	Slc38a3	1	0	0	1
rno-miR-873-5p	Slc39a7	1	0	0	1
rno-miR-873-5p	Slc45a3_predi	0	1	0	1
rno-miR-873-5p	Slc5a6	1	0	0	1
rno-miR-873-5p	Slc6a8	1	0	0	1
rno-miR-873-5p	Slc6a9	1	0	0	1
rno-miR-873-5p	Slc7a11_predi	0	1	0	1
rno-miR-873-5p	Slc7a9	1	0	0	1
rno-miR-873-5p	Slc8a2<i><sup	0	1	0	1

rno-miR-873-5p	Slc8a3	1	0	0	1
rno-miR-873-5p	Slc9a8	1	0	0	1
rno-miR-873-5p	Slco1a1	1	0	0	1
rno-miR-873-5p	Slco1b3	1	0	0	1
rno-miR-873-5p	Slfn8	1	0	0	1
rno-miR-873-5p	Smad3	1	0	0	1
rno-miR-873-5p	Smagp	1	0	0	1
rno-miR-873-5p	Smp2a	0	1	0	1
rno-miR-873-5p	Smpx	1	0	0	1
rno-miR-873-5p	Snap29	1	0	0	1
rno-miR-873-5p	Snx16	1	0	0	1
rno-miR-873-5p	Snx24	1	0	0	1
rno-miR-873-5p	Snx27	1	0	0	1
rno-miR-873-5p	Sox18	1	0	0	1
rno-miR-873-5p	Sox6	1	0	0	1
rno-miR-873-5p	Sp140	1	0	0	1
rno-miR-873-5p	Spaca3_predic	0	1	0	1
rno-miR-873-5p	Spata1	1	0	0	1
rno-miR-873-5p	Spats2_predic	0	1	0	1
rno-miR-873-5p	Spc25	1	0	0	1
rno-miR-873-5p	Spcs2_predict	0	1	0	1
rno-miR-873-5p	Spdya	1	0	0	1
rno-miR-873-5p	Spg7	0	1	0	1
rno-miR-873-5p	Spint4	0	1	0	1
rno-miR-873-5p	Spt1	1	0	0	1
rno-miR-873-5p	Srd5a3	1	0	0	1
rno-miR-873-5p	Srebf1	0	1	0	1
rno-miR-873-5p	Srpk1	0	1	0	1
rno-miR-873-5p	Srr	1	0	0	1
rno-miR-873-5p	Ssh2_predicte	0	1	0	1
rno-miR-873-5p	Sstr1	1	0	0	1
rno-miR-873-5p	Sstr2	1	0	0	1
rno-miR-873-5p	Stc1	1	0	0	1
rno-miR-873-5p	Stil	0	1	0	1
rno-miR-873-5p	Stk25	1	0	0	1
rno-miR-873-5p	Stk32b_predic	0	1	0	1
rno-miR-873-5p	Strn4	0	1	0	1
rno-miR-873-5p	Stxbp4_predic	0	1	0	1
rno-miR-873-5p	Sumo3	1	0	0	1
rno-miR-873-5p	Surf1	1	0	0	1
rno-miR-873-5p	Surf6_predicte	0	1	0	1
rno-miR-873-5p	Susd2_predict	0	1	0	1
rno-miR-873-5p	Syn1	0	1	0	1
rno-miR-873-5p	Syn3	1	0	0	1
rno-miR-873-5p	Syt1	1	0	0	1
rno-miR-873-5p	Syt7	1	0	0	1
rno-miR-873-5p	Tac4	1	0	0	1
rno-miR-873-5p	Tacr1	1	0	0	1
rno-miR-873-5p	Taf9b	1	0	0	1
rno-miR-873-5p	Tagln3	0	1	0	1
rno-miR-873-5p	Tbc1d20	0	1	0	1

rno-miR-873-5p	TBRG1_RAT	0	1	0	1
rno-miR-873-5p	Tbx4_predicte	0	1	0	1
rno-miR-873-5p	Tcea1	1	0	0	1
rno-miR-873-5p	Tcfcp2_predict	0	1	0	1
rno-miR-873-5p	Tdg	1	0	0	1
rno-miR-873-5p	Tdo2	1	0	0	1
rno-miR-873-5p	Tep1	0	1	0	1
rno-miR-873-5p	Tert	1	0	0	1
rno-miR-873-5p	Tes_predicted	0	1	0	1
rno-miR-873-5p	Tessp5	0	1	0	1
rno-miR-873-5p	Tex19.1	1	0	0	1
rno-miR-873-5p	Tfb1m	1	0	0	1
rno-miR-873-5p	Tfip11	1	0	0	1
rno-miR-873-5p	Tfpi	1	0	0	1
rno-miR-873-5p	Tfpt	0	1	0	1
rno-miR-873-5p	Tgfb3	1	0	0	1
rno-miR-873-5p	Tgfbrap1_pred	0	1	0	1
rno-miR-873-5p	Tgif1	1	0	0	1
rno-miR-873-5p	Thap4	0	1	0	1
rno-miR-873-5p	Thbs4	0	1	0	1
rno-miR-873-5p	Thex1	1	0	0	1
rno-miR-873-5p	Thoc6	1	0	0	1
rno-miR-873-5p	Thumpd1	1	0	0	1
rno-miR-873-5p	Timm17a	1	0	0	1
rno-miR-873-5p	Tinf2	1	0	0	1
rno-miR-873-5p	Tle1_predicted	0	1	0	1
rno-miR-873-5p	Tlr3	1	0	0	1
rno-miR-873-5p	Tmem106a	1	0	0	1
rno-miR-873-5p	Tmem106b	1	0	0	1
rno-miR-873-5p	Tmem130	0	0	1	1
rno-miR-873-5p	Tmem183a	1	0	0	1
rno-miR-873-5p	Tmem186	1	0	0	1
rno-miR-873-5p	Tmem19	1	0	0	1
rno-miR-873-5p	Tmem38b	1	0	0	1
rno-miR-873-5p	Tmem42	0	0	1	1
rno-miR-873-5p	Tmem42_pred	0	1	0	1
rno-miR-873-5p	Tmem54	0	1	0	1
rno-miR-873-5p	Tmem55a	1	0	0	1
rno-miR-873-5p	Tmem58_pred	0	1	0	1
rno-miR-873-5p	Tmem63c_pre	0	1	0	1
rno-miR-873-5p	Tmprss2	0	1	0	1
rno-miR-873-5p	Tnfaip1	1	0	0	1
rno-miR-873-5p	Tnfrsf8	1	0	0	1
rno-miR-873-5p	Tnni1	0	1	0	1
rno-miR-873-5p	Tnpo2	0	1	0	1
rno-miR-873-5p	Tnrc6b	0	0	1	1
rno-miR-873-5p	Topbp1	0	0	1	1
rno-miR-873-5p	Tp53	0	1	0	1
rno-miR-873-5p	Tp53i11_predi	0	1	0	1
rno-miR-873-5p	Tpp1	1	0	0	1
rno-miR-873-5p	Tra1	1	0	0	1

rno-miR-873-5p	Tram1	1	0	0	1
rno-miR-873-5p	Trappc1	0	1	0	1
rno-miR-873-5p	Trdn	1	0	0	1
rno-miR-873-5p	Trhr2	1	0	0	1
rno-miR-873-5p	Trim11	0	1	0	1
rno-miR-873-5p	Trim14	0	0	1	1
rno-miR-873-5p	Trim14_predic	0	1	0	1
rno-miR-873-5p	Trim26	1	0	0	1
rno-miR-873-5p	Trim35	1	0	0	1
rno-miR-873-5p	Trio	0	1	0	1
rno-miR-873-5p	Trpc4	1	0	0	1
rno-miR-873-5p	Trpv3	1	0	0	1
rno-miR-873-5p	Trpv5	1	0	0	1
rno-miR-873-5p	Tsen34	1	0	0	1
rno-miR-873-5p	Tsga10	0	1	0	1
rno-miR-873-5p	Tsga14	0	1	0	1
rno-miR-873-5p	Tsga2	0	1	0	1
rno-miR-873-5p	Tshz1	0	0	1	1
rno-miR-873-5p	Tspan11	1	0	0	1
rno-miR-873-5p	Tspan18	0	0	1	1
rno-miR-873-5p	Tspan3	1	0	0	1
rno-miR-873-5p	Tspan9	0	0	1	1
rno-miR-873-5p	Tspan9_predic	0	1	0	1
rno-miR-873-5p	Tspyl4	1	0	0	1
rno-miR-873-5p	Ttc1	0	1	0	1
rno-miR-873-5p	Ttc4	1	0	0	1
rno-miR-873-5p	Ttl	0	1	0	1
rno-miR-873-5p	Ttr	0	1	0	1
rno-miR-873-5p	Tuba4	0	1	0	1
rno-miR-873-5p	Txndc11_predi	0	1	0	1
rno-miR-873-5p	Txnl5_predicte	0	1	0	1
rno-miR-873-5p	Txnrd1	0	1	0	1
rno-miR-873-5p	Tyro3	1	0	0	1
rno-miR-873-5p	U2af1l4	0	1	0	1
rno-miR-873-5p	Ubc	0	1	0	1
rno-miR-873-5p	Ube2d3	1	0	0	1
rno-miR-873-5p	Ube2f	1	0	0	1
rno-miR-873-5p	Ube2v2	1	0	0	1
rno-miR-873-5p	Ubl3	1	0	0	1
rno-miR-873-5p	Ubr4	1	0	0	1
rno-miR-873-5p	Uchl1	0	1	0	1
rno-miR-873-5p	Ugcgl2	0	1	0	1
rno-miR-873-5p	Ugt2b37	1	0	0	1
rno-miR-873-5p	Ugt2b5	1	0	0	1
rno-miR-873-5p	Uhmk1	1	0	0	1
rno-miR-873-5p	Unc45b_predic	0	1	0	1
rno-miR-873-5p	Unc50	0	1	0	1
rno-miR-873-5p	UP1_RAT	0	1	0	1
rno-miR-873-5p	Upk1b	1	0	0	1
rno-miR-873-5p	Uqcrh	0	1	0	1
rno-miR-873-5p	Usp10	0	1	0	1

rno-miR-873-5p	V1rb5	1	0	0	1
rno-miR-873-5p	V1re24	0	1	0	1
rno-miR-873-5p	V1rf3	0	1	0	1
rno-miR-873-5p	Vamp1	1	0	0	1
rno-miR-873-5p	Vapb	1	0	0	1
rno-miR-873-5p	Vax1	0	1	0	1
rno-miR-873-5p	Vcpip1	1	0	0	1
rno-miR-873-5p	Vdac1	1	0	0	1
rno-miR-873-5p	Vdac3	1	0	0	1
rno-miR-873-5p	Vegfa	1	0	0	1
rno-miR-873-5p	Vim	1	0	0	1
rno-miR-873-5p	Visa	1	0	0	1
rno-miR-873-5p	Vps37a	1	0	0	1
rno-miR-873-5p	Vwa1	1	0	0	1
rno-miR-873-5p	Vwa5a	1	0	0	1
rno-miR-873-5p	Wac	0	1	0	1
rno-miR-873-5p	Wbp11	1	0	0	1
rno-miR-873-5p	Wbscr16_pred	0	1	0	1
rno-miR-873-5p	Wdfy1	1	0	0	1
rno-miR-873-5p	Wdr1	1	0	0	1
rno-miR-873-5p	Wdr44	0	1	0	1
rno-miR-873-5p	Wdr46	0	1	0	1
rno-miR-873-5p	Wdr5	1	0	0	1
rno-miR-873-5p	Wdr61	0	0	1	1
rno-miR-873-5p	Wfdc1	0	1	0	1
rno-miR-873-5p	Wfdcl1	0	1	0	1
rno-miR-873-5p	Whsc2	1	0	0	1
rno-miR-873-5p	Wrb	1	0	0	1
rno-miR-873-5p	Xkr7	1	0	0	1
rno-miR-873-5p	Xkr8	1	0	0	1
rno-miR-873-5p	Xkrx	1	0	0	1
rno-miR-873-5p	Xpo7	0	1	0	1
rno-miR-873-5p	Yrdc	1	0	0	1
rno-miR-873-5p	Ythdf1	1	0	0	1
rno-miR-873-5p	Ywhab	1	0	0	1
rno-miR-873-5p	Ywhaq	1	0	0	1
rno-miR-873-5p	Zbed3	1	0	0	1
rno-miR-873-5p	Zbtb1	1	0	0	1
rno-miR-873-5p	Zbtb9	1	0	0	1
rno-miR-873-5p	Zc3hav1	0	1	0	1
rno-miR-873-5p	Zcchc12	1	0	0	1
rno-miR-873-5p	Zcchc24	0	0	1	1
rno-miR-873-5p	Zdhhc20	1	0	0	1
rno-miR-873-5p	Zfp161	0	1	0	1
rno-miR-873-5p	Zfp207	1	0	0	1
rno-miR-873-5p	Zfp219	0	1	0	1
rno-miR-873-5p	Zfp322a	0	0	1	1
rno-miR-873-5p	Zfp346_predic	0	1	0	1
rno-miR-873-5p	Zfp366	0	1	0	1
rno-miR-873-5p	Zfp422	1	0	0	1
rno-miR-873-5p	Zfp423	1	0	0	1

rno-miR-873-5p	Zfp426l	0	0	1	1
rno-miR-873-5p	Zfp444_predic	0	1	0	1
rno-miR-873-5p	Zfp469	0	0	1	1
rno-miR-873-5p	Zfp472	1	0	0	1
rno-miR-873-5p	Zfp532	0	0	1	1
rno-miR-873-5p	Zfp535	0	1	0	1
rno-miR-873-5p	Zfp653_predic	0	1	0	1
rno-miR-873-5p	Zfyve27	1	0	0	1
rno-miR-873-5p	Zkscan1	1	0	0	1
rno-miR-873-5p	Zmym4	0	1	0	1
rno-miR-873-5p	Zmynd10	0	1	0	1
rno-miR-873-5p	Zmynd11	1	0	0	1
rno-miR-873-5p	Znf18	1	0	0	1
rno-miR-873-5p	Znf672	1	0	0	1
rno-miR-873-5p	Zp3	0	1	0	1
